# Effects of *Lactiplantibacillus plantarum* and *Lacticaseibacillus paracasei* supplementation on the single-cell fecal parasitome in children with celiac disease autoimmunity: a randomized, double-blind placebo-controlled clinical trial

**DOI:** 10.1186/s13071-023-06027-1

**Published:** 2023-11-09

**Authors:** Jakub Hurych, Elin Oscarsson, Åsa Håkanson, Kateřina Jirků-Pomajbíková, Milan Jirků, Carin Andrén Aronson, Ondřej Cinek, Daniel Agardh, the HEDIMED Investigator Group

**Affiliations:** 1 Department of Medical Microbiology, 2nd Faculty of Medicine, Charles University, Prague, Czechia; 2 Department of Paediatrics, 2nd Faculty of Medicine, Charles University, Prague, Czechia; 3 Department of Food Technology, Engineering and Nutrition, Lund University, Lund, Sweden; 4 Institute of Parasitology, Biology Centre, Czech Academy of Sciences, České Budějovice, Czechia; 5 Celiac Disease and Diabetes Unit, Department of Clinical Sciences, Lund University, Malmö, Sweden

**Keywords:** *Blastocystis*, *Dientamoeba fragilis*, Celiac disease, Probiotics, Gut microbiome

## Abstract

**Background:**

*Lactiplantibacillus plantarum* HEAL9 and Lacticaseibacillus *paracasei* 8700:2 positively affect the fecal bacteriome in children with celiac disease autoimmunity after 6 months of supplementation. The aim of the present investigation was to study the effects of *Lactiplantibacillus plantarum* HEAL9 and *Lacticaseibacillus paracasei* 8700:2 on the single-cell parasitome, with a primary focus on *Blastocystis*.

**Methods:**

Stool samples were collected from 78 Swedish children with celiac disease autoimmunity participating in a randomized, double-blind, placebo-controlled clinical trial to either receive a mixture of supplementation with *L. plantarum* HEAL9 and *L. paracasei* 8700:2 (*n* = 38) or placebo (*n* = 40). A total of 227 stool samples collected at baseline and after 3 and 6 months of intervention, respectively, were retrospectively analyzed for *Blastocystis* by quantitative real-time PCR and subtyped by massively parallel amplicon sequencing. Other single-cell parasites were detected by untargeted 18S rDNA amplicon sequencing and verified by real-time PCR. The relation between the parasites and the bacteriome community was characterized by using 16S rDNA profiling of the V3-V4 region.

**Results:**

Three different single-cell protists were identified, of which the highest prevalence was found for *Dientamoeba fragilis* (23.1%, 18/78 children), followed by *Blastocystis* (15.4%, 12/78) and *Entamoeba* spp. (2.6%, 2/78). The quantity of the protists was stable over time and not affected by probiotic intervention (*P* = 0.14 for *Blastocystis*, *P* = 0.10 for *D. fragilis*). The positivity of the protists was associated with increased bacteriome diversity (measured by multiple indices, *P* < 0.03). Bacterial composition was influenced by the presence of the protists: positivity of *Blastocystis* was inversely associated with *Akkermansia* (at the levels of the genus as well as its family, order, class and phylum); *P* < 0.002), *Faecalibacterium* (*P* = 0.003) and *Romboutsia* (*P* = 0.029); positivity of *D. fragilis* was inversely associated with families Enterobacteriaceae (*P* = 0.016) and Coriobacteriaceae (*P* = 0.022) and genera *Flavonifractor* (*P* < 0.001), *Faecalibacterium* (*P* = 0.009), *Lachnoclostridium* (*P* = 0.029), *Ruminococcus* (*P* < 0.001) and *Granulicatella* (*P* = 0.018).

**Conclusions:**

The prevalence of single-cell protists is low in children with celiac disease autoimmunity. The colonization was stable regardless of the probiotic intervention and associated with increased diversity of the fecal bacteriome but inversely associated with some beneficial bacteria.

**Graphical Abstract:**

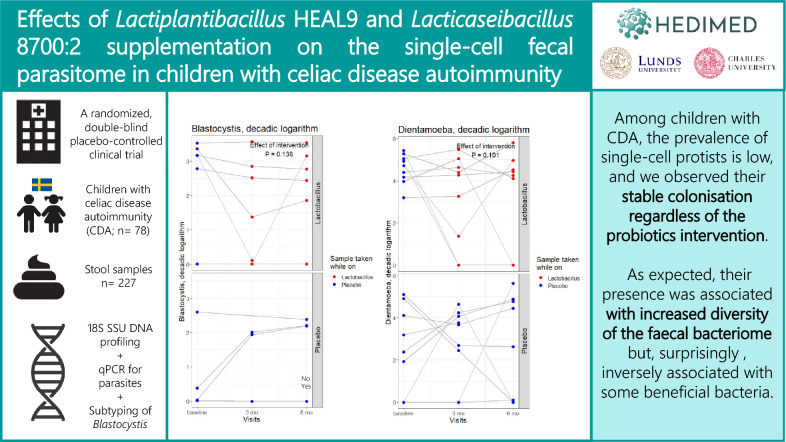

**Supplementary Information:**

The online version contains supplementary material available at 10.1186/s13071-023-06027-1.

## Background

The main protist of the human gut is *Blastocystis* sp. (hereafter referred to by its genus name *Blastocystis*). It is an anaerobic, non-flagellated single-cell and highly polymorphic organism with multiple forms. *Blastocystis* is proposed to be a marker of a balanced microbiome [1–3] and is believed to be the most common single-cell eukaryote in the gut of both adults [4] and children [5]. Being mainly transferred by the fecal-oral route, *Blastocystis* has a higher prevalence in developing countries with lower hygiene standards, usually between 40 and 84% [6–8], compared with high-income countries in Europe and the USA, where it commonly ranges between 7 and 56% [1, 9–15]. Among the genus *Blastocystis*, 26 genetic subtypes (ST) were identified [16] and proposed to be differing in biological properties. Of these subtypes, STs 1–17 are widely recognized as valid, whereas others may include molecular detection artifacts [17]. Of the ten subtypes in humans, ST1 to ST4 are the most prevalent [5, 18], from which ST4 is unique for Europe and ST3 is the most prevalent worldwide [10]. Another frequent protist is *Dientamoeba fragilis*, a flagellated trichomonad, surrounded by controversy about whether it is more a pathogen or a benign gut inhabitant [19–21]. Its prevalence varies between 0.4 and 71%, depending on the studied cohort and methods used [21–24]. Interestingly, unlike *Blastocystis*, its prevalence is lower in low-income countries, e.g. [25, 26], and higher in high-income countries, e.g. [22, 27].

Celiac disease is a chronic enteropathy due to an immune-mediated response to dietary gluten from wheat, rye and barley, arising in a small proportion of individuals who are genetically susceptible because carrying human leukocyte antigens (HLA) haplotypes DQ2 and/or DQ8 [28]. Although genetics and gluten are necessary for celiac disease development, it is most likely triggered by environmental factors such as gastrointestinal infectious episodes triggered by different microorganisms [29], 29. On the other hand, the prevalence of celiac disease has been reported to be significantly lower in populations of Russian Karelia with lower hygienic and socioeconomic standards with high exposures to microbes compared to populations living in Finland, despite the populations from the two geographically neighboring regions sharing the same genetic risk [31].

In contrast to the above study, recent prospective birth cohort studies on gastrointestinal infections have shown that patients who develop celiac disease have more frequent enterovirus [32] and parechovirus [33] infections prior to seroconversion of tissue transglutaminase autoantibodies (tTGA), a marker of celiac disease autoimmunity (CDA) [32].

The present study builds on the probiotic intervention trial in children with CDA [34], showing a moderate effect of probiotics on the gut bacteriome [35]. This study focuses on the fecal parasitome. The aim was to assess the effect of the intervention with two lactobacilli, probiotic strains *Lactiplantibacillus plantarum* HEAL9 and *Lacticaseibacillus paracasei* 8700:2, on the single-cell parasitome with specific emphasis on *Blastocystis* in a stool sample set of Swedish children with CDA. Our hypothesis was that the presumably beneficial probiotic bacteria might change the bacteriome composition, leading to an increased prevalence of *Blastocystis*.

## Materials and methods

### Study population

**Fig. 1:**
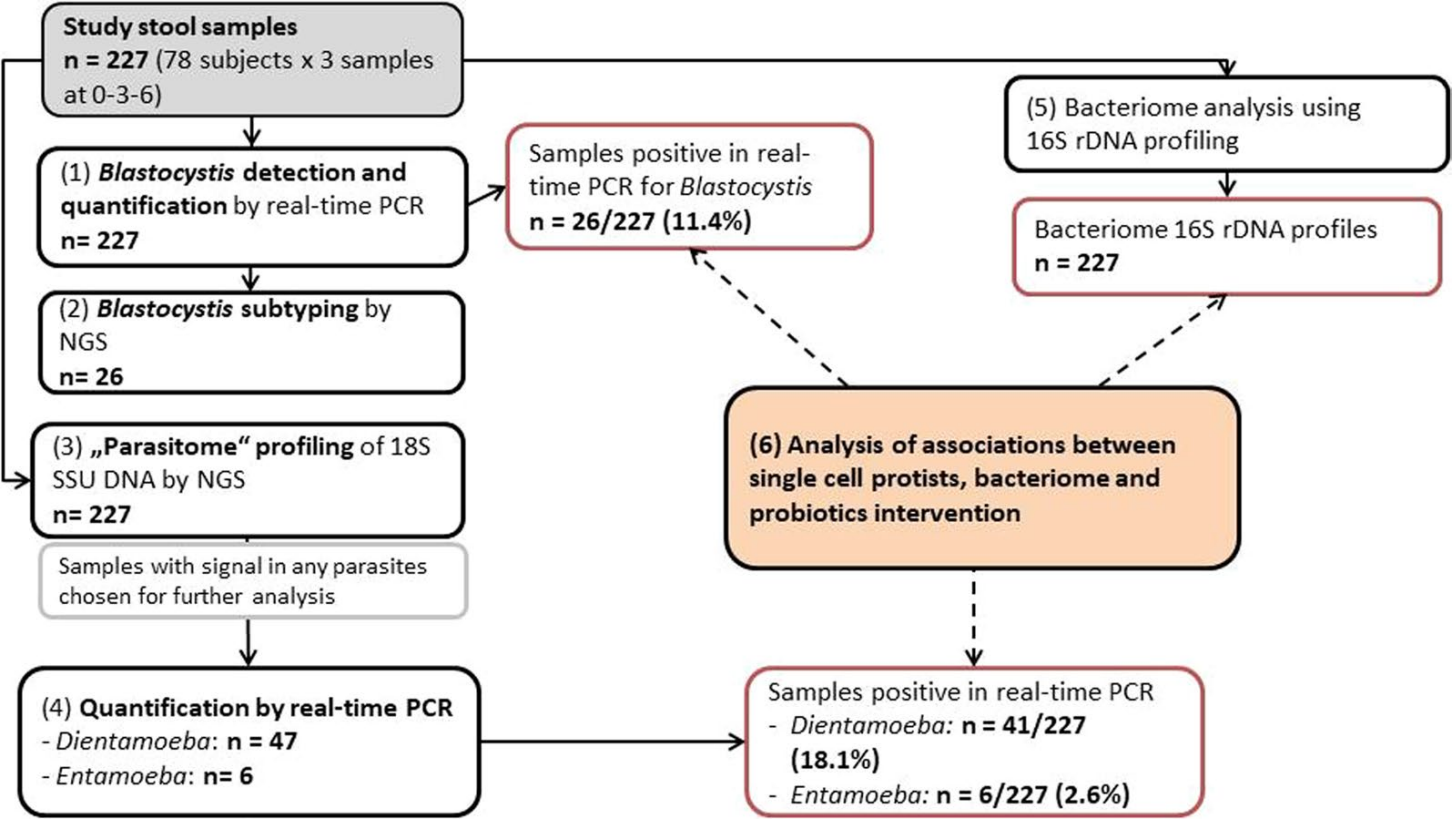
Workflow of the study. *NGS* next-generation sequencing, *SSU* small subunit of the ribosome

The Celiac Disease Prevention with Probiotics (CiPP) study, a double-blind placebo-controlled randomized clinical trial, was performed at the Department of Clinical Sciences, Unit of Celiac Disease and Diabetes, Lund University, Malmö, Sweden [34]. The trial was carried out between March 12, 2012, and August 25, 2015. A total of 118 children with CDA identified by screening were invited to participate in the CiPP study, of whom 89 accepted participation and 78 completed a 6-month follow-up of daily receiving either a mixture of *L. plantarum* HEAL9 and *L. paracasei 8700:2* (probiotic group, *n* = 40) or 1 g maltodextrin and yeast peptone (placebo group, *n* = 38) (the probiotic mixture is described in more detail in [34, 35]). Study participants were instructed to continue a gluten-containing diet and exclude other food products containing probiotics. During the intervention, six children in the probiotic group and four in the placebo group reported taking antibiotics (*P* = 0.561). Stool samples were collected at baseline and then 3 and 6 months after the intervention. Processing of the stool samples for DNA extraction was described earlier [35]. Briefly, 50 mg of thawed stool sample was used for the DNA extraction using the DNA tissue kit (Qiagen, Germany) and run on the EZ1 DNA extraction robot (Qiagen, Germany). The study was approved by the Ethics Committee of the Medical Faculty, Lund University (Dnr 2011/335; Dnr 2021–04470) and registered at ClinicalTrials.gov (NCT03176095); 227 samples collected from the 78 children with CDA participating in the (CiPP) study were available for this retrospective analysis (Fig. 1).

### Molecular methods

*Blastocystis* was tested and quantified by specific real-time PCR as the most common gut protist [18]. All positive samples for *Blastocystis* were then classified into genetic subtypes using massively parallel amplicon sequencing of a unique region of the 18S rDNA gene. The total parasitome was profiled by multiplex massively parallel amplicon sequencing of several amplicons of the 18S rDNA gene. Positive parasites from the 18S rDNA profiling (other than *Blastocystis*) were then confirmed and quantified by parasite-specific real-time PCR. After determining the single-cell parasitome content, the parasites were related to the intervention with studied probiotics and analyzed in the context of previously obtained bacteriome profiles [35].

#### Detection and quantification of blastocystis by specific real-time quantitative PCR

A real-time quantitative PCR assay with a specific probe for *Blastocystis* designed by Stensvold et al. [36] was used to test and quantify *Blastocystis*. For the calibration curve, DNA from a microscopically quantified xenic culture was used [37]. Platinum Taq Polymerase (Invitrogen, USA) from the Stensvold’s original protocol was substituted by the HotStar Taq polymerase chemistry (Qiagen, Germany), and the PCR program was corrected accordingly with 15 min of initial denaturation at 95 °C followed by 45 cycles of 15 s denaturation at 94 °C and 60 s of combined annealing and synthesis at 60 °C [5]. Reactions were performed in duplicates throughout the study, and negative controls were included in the extraction as well as in the detection reactions. PCR tubes containing real-time PCR products were always discarded unopened. The detection PCR fragments do not overlap with those used for *Blastocystis* subtyping.

#### Subtyping of blastocystis by massively parallel amplicon sequencing

Samples positive for *Blastocystis* in the above-mentioned real-time PCR assay were thereafter subtyped by massively parallel amplicon sequencing using the protocol by Maloney et al. [38] with primers developed by Santin et al. [39] extended by Nextera tails. The amplicons were then purified and indexed by Nextera XT Index Kit v2 Set A and D (Illumina, USA) by a short eight-cycle PCR, enabling multiplex sequencing. Indexed products were purified and equalized using a SequalPrep Normalization plate (Invitrogen, USA) and pooled. The pool of libraries was sequenced on a MiSeq instrument using Reagent Kit v2, 2 × 250 bp (Illumina, USA), with the addition of 20% PhiX control to balance the amplicon signal.

#### Parasitome survey using massively parallel amplicon profiling of the 18S rDNA gene

The parasitome survey was performed using massively parallel sequencing with five different PCR amplicons of the 18S rDNA gene, as reported in detail previously [5]. The procedure identified 3546 unique sequence variants and zero-radius operational taxonomic units (ZOTUs); those with a relative frequency > 0.1% (*n* = 103) were manually classified by BLAST search to GenBank. Signals of the single-cell protists were expressed as the number of reads aligned to a particular organism divided by the count of rarefied reads per reaction.

#### Quantification of *D. fragilis* and *Entamoeba* spp. by quantitative real-time PCR

The single-cell protists with a signal in the parasitome survey (*D. fragilis* and *Entamoeba* spp. apart from already quantified *Blastocystis*) were then quantified by a specific probe-based real-time quantitative PCR assay. For *D. fragilis*, the primer and probe sequences came from the work of Verweij et al. [40]. A dilution series of DNA from a xenic culture of *D. fragilis* was used as a control in qPCR reactions, but its exact protist genomic equivalent content could not be established because of problems with the microscopic quantification of the culture. The PCR conditions for *D. fragilis* detection were published in [41]. As to *Entamoeba* spp. (*E. histolytica, dispar, moshkovskii, hartmanni, coli*), the primer and probe sequences were designed in the Geneious Prime software (version 2020) based on the 18S SSU rRNA gene sequence references (X64142, AB197936, Z49256, AF149906, AF149907, AF149915) retrieved from the GenBank database (see Additional file 2: Table S1 and Figure S1). The PCR program for *Entamoeba spp.* detection consisted of 15 min of initial denaturation at 95 °C followed by 35 cycles of 15 s denaturation at 94 °C and 60 s of combined annealing and synthesis at 60 °C. For a calibration curve, DNA from an axenic *Entamoeba* culture was used, and cell counts from the culture were calculated using a Bürker chamber and then serially diluted to obtain aliquots containing 1, 10, 10^2^, 10^3^, 10^4^ and 10^5^ cells per microliter, which were subsequently subjected to DNA extraction according to Lhotská et al. [15]. Negative controls were included in every PCR run, and the tubes with PCR products were discarded unopened.

#### Bacteriome analysis

The processing, DNA library preparation and sequencing had been done previously by massively parallel amplicon sequencing of the V3-V4 region of the bacterial 16S rRNA gene [35]. For this study, the previously generated *fastq* files with sequencing reads were downloaded and reprocessed using the DADA2 pipeline (version 1.22) [42], with taxonomic classification using the SILVA database (version 138) [43] instead of the now slightly outdated Greengenes database version 13.5 and the Qiime2 suite. Amplicon sequence variants (ASVs, analogous to operational taxonomic units, OTUs) were then further analyzed using vegan [44] and phyloseq [45] in the R programming language [46].

### Statistical analysis

The prevalence of single-cell protists was determined by counting any PCR positivity at any level. Their presence and quantity were then modeled using generalized estimating equations (GEE) with the subject as the grouping variable, time point (factor with three levels—0, 3 and 6 months) and a first-order autoregressive covariance structure; the predictors were the study allocation (placebo or probiotics) and whether the sample was taken while on intervention with the live mixture.

Bacteriome alpha (within-sample) diversity was assessed from the unfiltered rarefied dataset, agglomerated at the genus level, using the observed counts, Chao1, ACE, Shannon, Simpson, inverse Simpson and Fisher indices. The association of alpha diversity indices with the presence or quantity of *Blastocystis* or *D. fragilis* was assessed in GEE models with the index as the dependent variable where predictors were the positivity or quantity of the parasites, and the study allocation (placebo or probiotics).

For the beta diversity (between samples) analysis, Bray-Curtis distance was calculated on Hellinger-transformed abundance data agglomerated at the taxonomic level of the genus, and ordination was performed using metric multi-dimensional scaling (MDS) and visually inspected. Associations of *Blastocystis* and/or *D. fragilis* positivity with fecal bacteriome composition were tested using constrained ordination, the redundancy analysis (RDA) on the Hellinger-transformed abundance data.

Using GEE, individual bacterial taxa were tested for associations with the positivity of the two abundant protists (*Blastocystis* or *D. fragilis*). Taxa having more than 20/15000 reads in at least two samples were considered. In this model, the outcome was the relative abundance of a bacterium, and the predictors were positivity for *Blastocystis* and/or *Dientamoeba*, time point (factor with three levels—0, 3 and 6 months) and intervention (on probiotics vs. on placebo). An autoregressive correlation matrix was used. The models were built for every taxonomic unit at the genus, family, order, class and phylum levels. Ensuing nominal *P* values were adjusted using the Bonferroni method for the number of taxonomic entities tested at the given level. In an additional model, the dependent variable was the fold difference of bacterium quantity from the ASV table after the centered log-ratio transformation (CLR) with a small pseudo-count replacing zero values.

The commented R Markdown code and output of the statistical analysis are shown, along with the session information, as Additional file 1: Statistical analysis—R Markdown.

## Results

### Fecal parasitome analysis

The molecular survey using 18S rDNA parasitome profiling and/or specific quantitative PCR for the single-cell protists revealed positivity in widely varying quantities: in *Blastocystis* the threshold cycles (Ct) ranged from 17.9 to 41.2, corresponding to quantities of < 1/100 to > 10,000,000 genomic equivalents (g.e.) per µl DNA. In *D. fragilis*, the threshold cycles ranged from 19.6 to 42.6; the abundance in genomic equivalents is not known since the standard culture could not be microscopically quantified. In *Entamoeba*, the threshold cycles ranged from 23.2 to 30.3, corresponding to quantities of 1 to > 100 genomic equivalents per µl DNA.

The subject-wise prevalence of these three protists, calculated as at least once positive among the three time points, was highest in *D. fragilis* (18/78, 23.1%), slightly lower in *Blastocystis* (12/78, 15.4%) and the lowest in *Entamoeba* spp. (2/78, 2.6%) (Table 1 and Additional file 2: Fig. S2). *Dientamoeba fragilis* was more frequent in *Blastocystis*-positive samples (*P* = 0.015) but not the other way around (*P* = 0.057, both from GEE models). *Entamoeba* was only present in stable loads in one subject in each intervention group.

**Table 1:** Sample- and subject-wise prevalence of the protist among CiPP study participants

Protist species positivity	*Blastocystis*	*Dientamoeba fragilis*	*Entamoeba* spp.
By samples (*n* = 227)	26 (11.5%)	41 (18.1%)	6 (2.6%)
By subjects (*n* = 78)			
Ever positive	12 (15.4%)	18 (23.1%)	2 (2.6%)
Persistent positivity	8	10	2
Newly acquired positivity	1	2	0
Converted into negativity	3	5	0
Other pattern	0	1	0

**Fig. 2:**
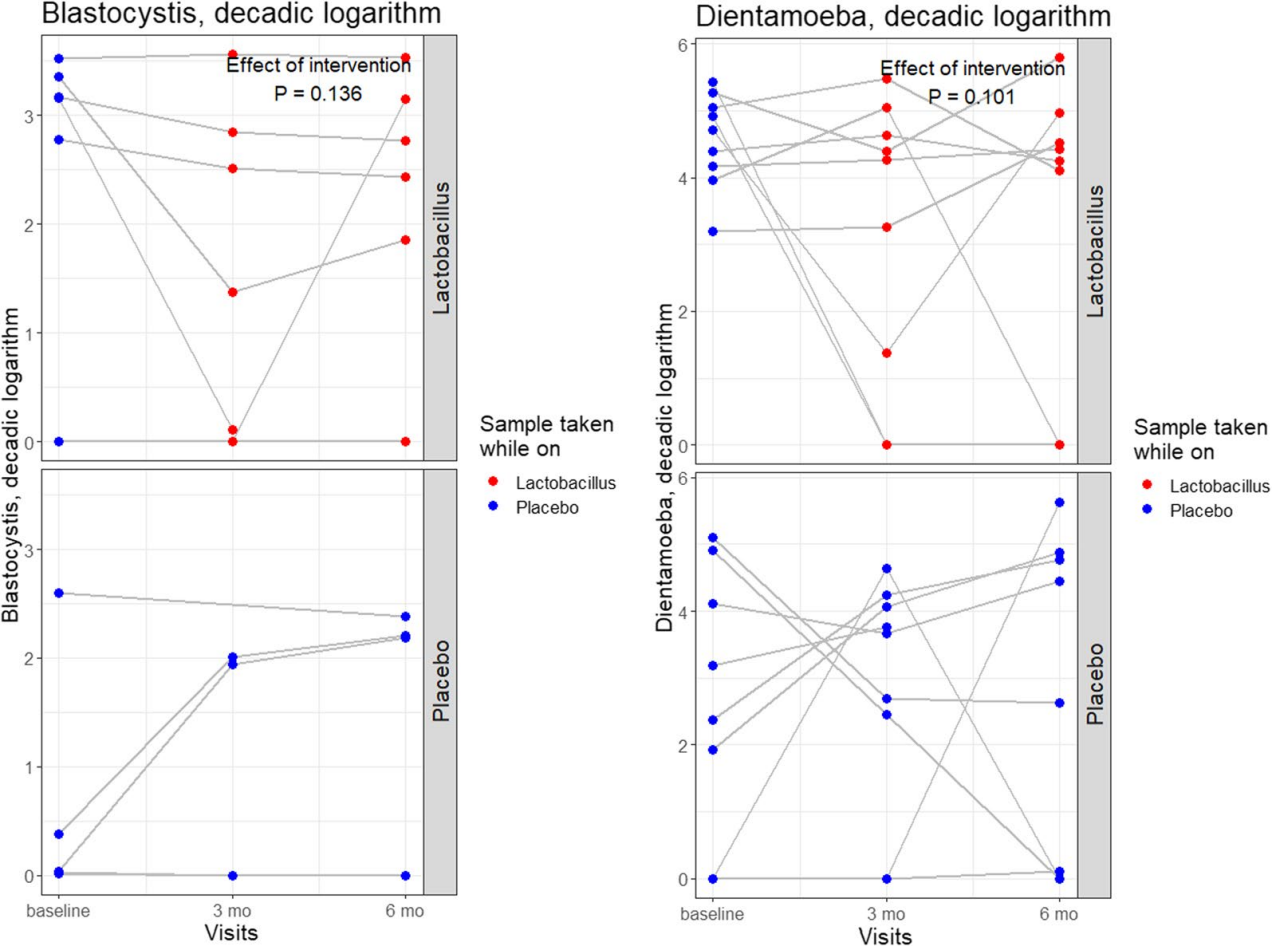
Quantity of *Blastocystis* and *Dientamoeba fragilis* in children with celiac disease autoimmunity receiving *Lactobacillus* (*Lactobacillus* group) or placebo (Placebo group) over 6 months of intervention

The protist frequency did not differ between the treatment groups (intervention vs. placebo) at baseline, and no significant effect on protist frequency was noted upon receiving the probiotic intervention (GEE with terms for the allocated treatment group, studied predictor being the ongoing intervention with lactobacilli: *P* = 0.34 for positivity of *Blastocystis*; *P* = 0.14 for *D. fragilis*; *P* = 0.31 for any of the three protists. Similarly, no influence of the intervention was noted on protist quantity—this was assessed in GEE models with the logarithm of quantity as the outcome (Fig. 2).

### Blastocystis subtypes

**Fig. 3:**
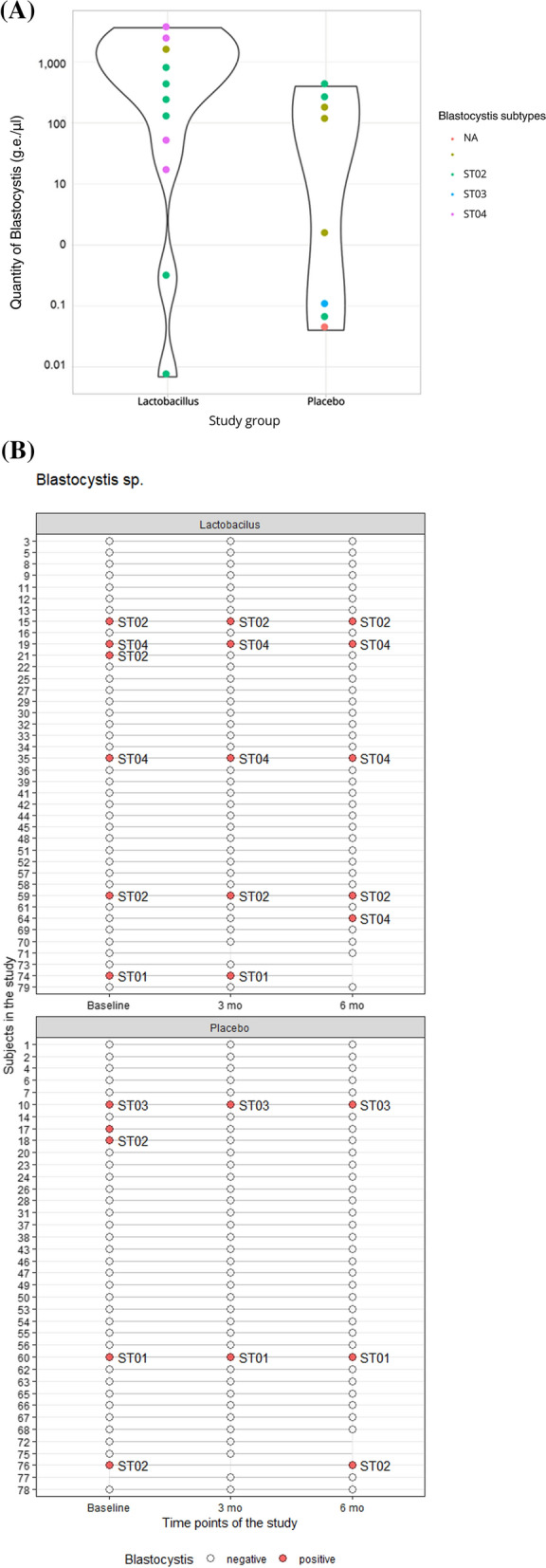
Blastocystis subtypes. (**A**) *Blastocystis* quantity and subtype distribution; (**B**) stability of *Blastocystis* subtypes

Of 26 *Blastocystis*-positive samples, 25 had their subtype identified, while one failed in the sequencing (likely because of very low quantity, Ct 41.2). Four *Blastocystis* subtypes were identified: the most prevalent one was ST2 (10/25 identified, 40%), followed by ST4 (7/25, 28%), ST1 (5/25, 20%) and ST3 (3/25, 12%). None of the samples was positive for more than one subtype (Fig. 3A and B). Quantity did not differ by *Blastocystis* sequence type (*P* = 0.46), and there was no difference in the subject-wise prevalence of the four observed subtypes. The overview of each sample’s results (qPCR, subtyping and parasitome survey) is summarized in the Additional file 3: Table—Parasites detection.

### Protists and the fecal bacteriome

**Fig. 4:**
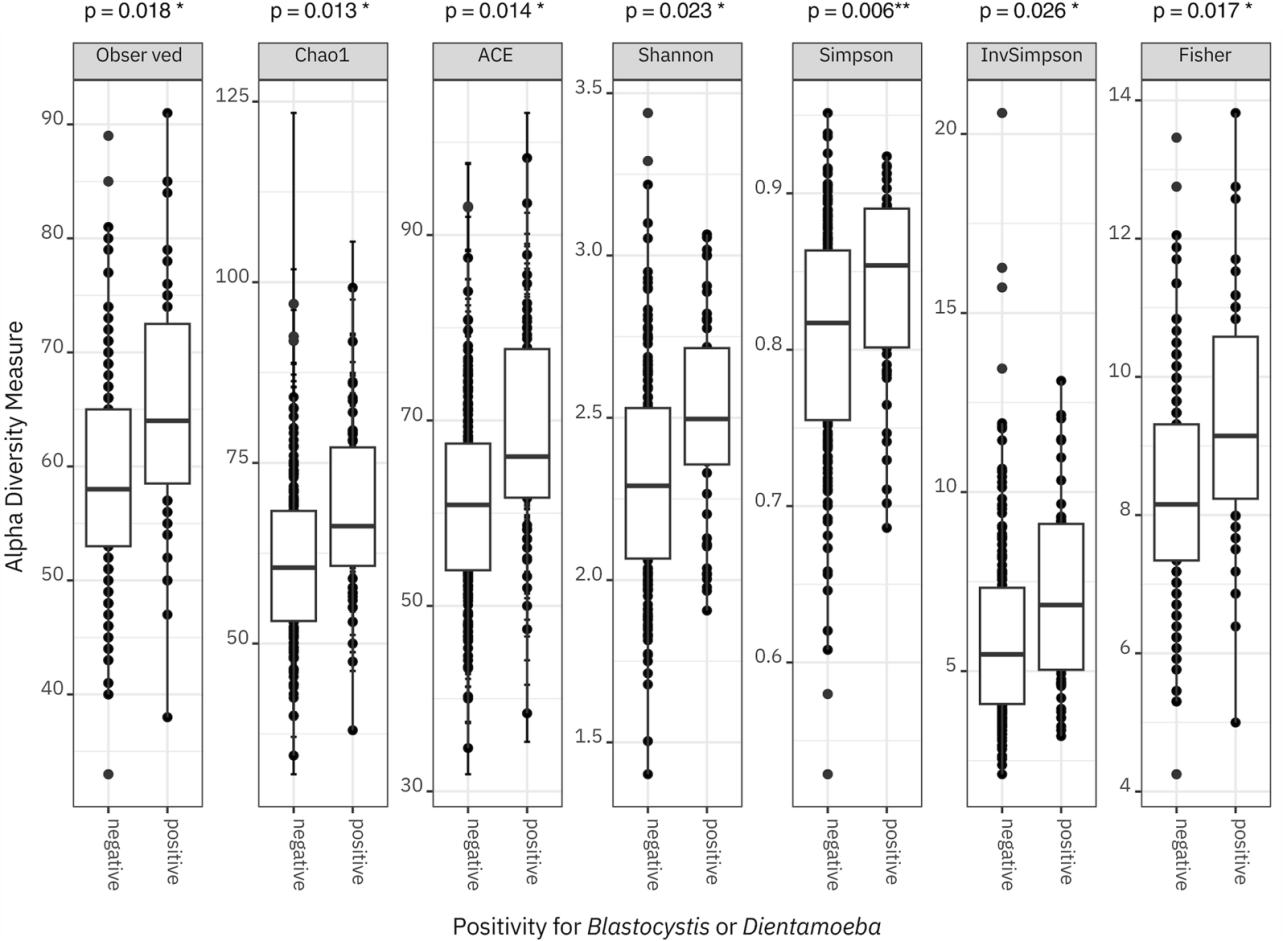
Alpha diversity of the faecal bacteriome and associations with positivity for *Blastocystis* or *Dientamoeba fragilis*

The presence of *Blastocystis* and/or *D. fragilis* was associated with a higher alpha diversity of the fecal bacteriome. This association was significant in a GEE model for the count of observed genera (*P* = 0.018), the Chao1 (*P* = 0.013), ACE (*P* = 0.014), Shannon (*P* = 0.023) and Fischer (*P* = 0.017) indices as well as for the Simpson (*P* = 0.0057) and inversed Simpson (*P* = 0.026) indices (Fig. 4). The difference in alpha diversity between protist-positive and -negative stools was most prominent in those collected at the second time point. Alpha diversity neither changed over time nor differed between treatment groups at baseline and was not associated with the probiotic intervention.

Redundancy analysis performed on Hellinger-transformed bacterial abundance data indicated no effect of the intervention (*P* = 0.591), but the bacteriome community composition was associated with the presence of *Blastocystis* (*P* = 0.006) and *D. fragilis* (*P* = 0.001) (Additional file 2: Figure S3). The proportion of overall community variance explained by the two protists was low (1.0% for *Blastocystis* and 1.95% for *D. fragilis*). The effects of the two protozoa were nearly orthogonal, i.e. the associations with individual microbes of the bacteriome differed. Also, upon an inspection of ordination plots of Bray–Curtis distance at the genus level, samples positive for either *Blastocystis* and/or *D. fragilis* showed a tendency towards moderate separation from those being negative for the parasites (Additional file 2: Figure S4). As the spread of the two categories significantly differed (function *vegan::betadi*sp*er*, *P* < 0.001), testing by permutational analysis of variance (PERMANOVA) was not meaningful—its significance (*P* < 0.001) reflected either the difference in centroid position or the above-demonstrated difference in the spread, or both.

When studying the bacterial taxa associated with positivity for parasites using adjusted GEE (Table 2), *Blastocystis* was inversely associated with the genera *Akkermansia* (and its taxonomic categories up to the level of phylum Verrucomicrobia), *Faecalibacterium* and *Romboutsia*, both from class Clostridia. The presence of *D. fragilis* was inversely associated with the genera *Flavonifractor, Faecalibacterium, Lachnoclostridium, Ruminococcus* and *Granulicatella*. Most of the above associations were apparent in both the linear and fold-difference models, except for *Akkermansia*, whose association was detectable only when linear quantity was used as the outcome.

**Table 2:**
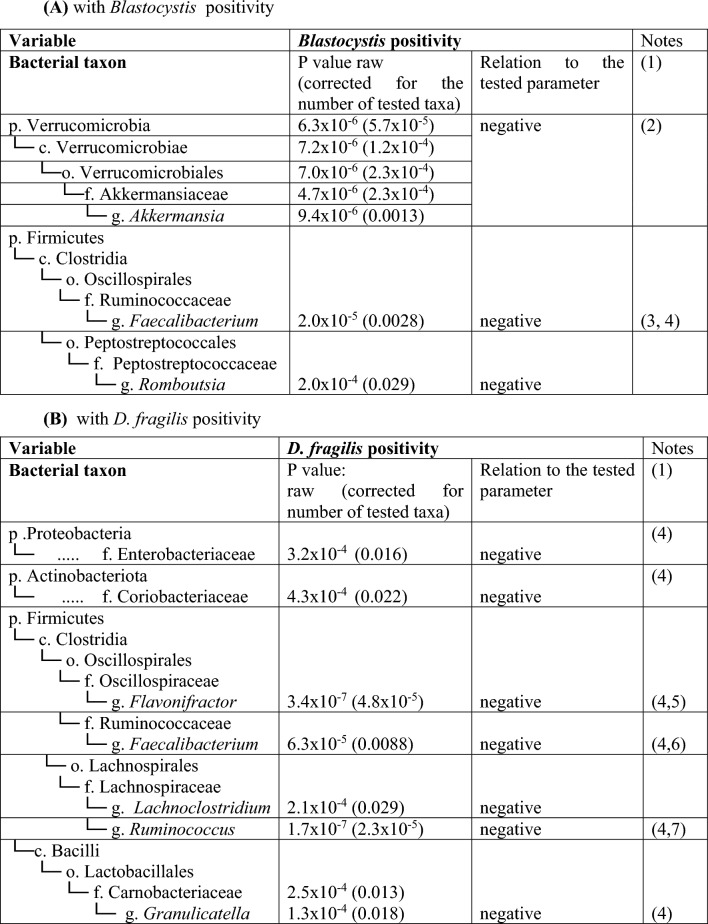
Bacterial taxa showing association with protist positivity: (A) with *Blastocystis* positivity, (B) with *D. fragilis* positivity

(1) Negative means an inverse correlation (and thus negative coefficient in the gee model) and vice versa

(2) *Akkermansia* was the only tested genus showing significance up to the level of phylum.

(3) Classified as *Faecalibacterium* sp. UBA1819 by SILVA database v. 138

(4) Significance noted also in the fold-change model with data after centered log-ratio transformation (adjusted *P* < 0.05)

(5) Significant only at genus level

(6) Classified as [*Ruminococcus*] torques group by SILVA database v. 138

Raw *P*-values were corrected using Bonferroni correction for each taxonomic level (9 phyla, 16 classes, 33 orders, 50 families and 119 genera)

## Discussion

This study analyzed the single-cell parasitome in a randomized clinical trial of probiotic strain intervention in CDA and found a relatively stable protist colonization (*Blastocystis* and/or *D. fragilis*) regardless of the probiotic intervention. The intervention with the two lactobacilli caused moderate changes in the bacteriome [35]; however, we observed no effects on the parasitome composition. Rather, the opposite occurred: positivity for a parasite was stable over the observation period and associated with an increased richness of the bacteriome but inversely associated with some presumably beneficial bacteria.

By targeted molecular testing using quantitative real-time PCR, we first looked at the prevalence of *Blastocystis* as the prime representative of the fecal parasitome [4, 5]. Then, we explored the whole parasitome by 18S rDNA profiling and assessed the quantities of the positive ones by specific real-time PCR assays for each of the protists. The subject-wise prevalence of the single-cell protists among children with CDA was low (23.1% for *D. fragilis*, 15.4% for *Blastocystis* and only 2.6% for *Entamoeba* spp.), in accordance with the hygiene hypothesis [47] and also to a recent report in celiac disease patients (regarding *Blastocystis*) [48].

The prevalence of *Blastocystis* is reported to be higher in lower-income countries and vice versa. Among the high-income countries, the lowest prevalence in a healthy population of only 7% was reported in Colorado, USA [13]. In continental Europe, it ranges around 20–30% (France 18%, The Netherlands 24%, Czechia 24%, Belgium 30%) [1, 11, 12, 15] and surprisingly higher prevalence of 56% was reported in Ireland [14]. On the other hand, two studies from rural populations of Nigeria [6] and Senegal [49] report a very high prevalence of 84% or 100%, respectively. Similarly, in underprivileged areas of Malaysia and Brazil, a higher prevalence of 41% and 47%, respectively, was reported [7, 8]. Thus, our results are in accord with the *Blastocystis* geographical gradient in healthy adults and children.

Although *Blastocystis* is often reported as the most common gut eukaryote [4], D. fragilis was more prevalent in the present dataset. This might be due to its higher prevalence in developed, urbanized countries [22, 27], but perhaps the location in the south of Sweden played a more prominent role, as supported by Jokelainen, who reported high prevalence in asymptomatic children under the age of 6 in day-care centeres in Copenhagen [20]. Additionally, a high prevalence of *D. fragilis* (compared to other intestinal parasites) was reported in urban areas of Copenhagen, Denmark [22, 50] (in adults) and Jönköping, Sweden [51] (in children) among patients with gastrointestinal issues suspected of parasitosis. Thus, the higher rates of *D. fragilis* than *Blastocystis* in our study seem meaningful regarding the geographic proximity to Copenhagen. Still, the high prevalence of *D. fragilis* in the region raises the question of its origin. It might be an endemic issue, but it might also be a simple reporting bias caused by the existence of several well-established parasite research groups in Denmark and Sweden. Similar isolated cases of a surprisingly high prevalence of *Dientamoeba* have been recently reported, e.g. in The Netherlands [52, 53] and Czechia [21], where groups studying single-cell protists also utilized molecular detection.

The colonization by these protists was stable over time and not influenced by the intervention of lactobacilli strains. The temporal stability in children was well described for *D. fragilis* [20]; however, for *Blastocystis* there is only one study in adults with a small number of subjects (*n* = 10) [14], making our study the first to report such stability of the parasitome among children.

The mixture of two lactobacilli strains was found to modulate the immune response [34] and change the fecal bacteriome towards a healthier composition [35]. However, it showed no detectable effect in modulating the fecal parasitome in presumably asymptomatic CDA patients. In contrast, probiotics may modulate parasite positivity in symptomatic patients: two studies investigated the effects of probiotics in treating symptomatic *Blastocystis hominis* infection. Dinleyici et al. showed a significant effect of *Saccharomyces boulardii* probiotics on *Blastocystis* eradication similar to metronidazole in vivo [54]; Lepczynska et al. reported an effect of a mixture of probiotics (*Lactobacillus rhamnosus, Lactococcus lactis* and *Enterococcus faecium*) on *Blastocystis* ST3 eradication in vitro [55].

In our study, ST2 was the most often identified subtype among study samples, albeit ST3 is reported as the most frequent worldwide [18]. ST3 was, on the contrary, found to be the least frequent. The occurrence of ST4, even as the second most common, is consistent with its strict European predilection [4]. Otherwise, the subtype distribution showed no association with the intervention.

The fecal bacteriome reaction to the mixture of *L. plantarum* HEAL9 and *L. paracasei* 8700:2 in our RCT was described earlier[35]. Briefly, the 6-month intervention with probiotics led to a shift in bacteriome composition in a healthier direction, most notably to an increase in *Akkermansia*, however, without any changes in alpha diversity indices. Celiac disease itself has been associated with changes in the gut bacteriome [56–60]. More specifically, it was associated with an increase in pro-inflammatory bacteria Enterobacteriaceae[56], *Bacteroides* [59] and *Fusobacterium* [56] and a decrease in *Akkermansia* [56], a beneficial bacterium for the gut epithelium [61]. Moreover, potential predictors of celiac disease were suggested, including an increase in *Porhyromonas*, *Dialister* and *Parabacteroides* and a decreased abundance of anti-inflammatory species [60] or decreased capacity to degrade gluten [57]. Importantly, 3-month probiotic supplementation shifted the bacteriome towards a healthier composition [58]. In contrast, recent work, with low patient count but innovative design, provided evidence that celiac disease might not be consistently associated with dysbiotic microbiome[62].

The probiotics did not influence the bacteriome’s alpha diversity; however, we found evidence of protist positivity increasing the alpha diversity similar to what has previously been described in adults, e.g. [2], and children [5]. As speculated in our previous work, the relation between protist positivity and rich bacteriome ecosystem might be in the thriving of *Blastocystis* in distinct communities [5] or the protist itself modifying the ecosystem [63]. Moreover, based on our current findings, *D. fragilis*, not just *Blastocystis*, is associated with fecal bacteriome diversity.

The positivity of *Blastocystis* and/or *D. fragilis* was associated with a difference in bacterial community composition. Even though the protist occurrence explained only a small part of the bacteriome beta diversity, the association was highly significant. Of particular taxa associated with *Blastocystis*, a negative association with *Akkermansia* and its upward taxonomic categories stands out. *Akkermansia* is a known mucin degrader and producer of short-chain fatty acids (SCFA) [64] and is thus generally considered beneficial to the human gut ecosystem [61]. It was found to be depleted in celiac disease [56] and, on the contrary, enriched after receiving probiotics in the CiPP study [35]. Of note, here, the *Akkermansia* association was only significant in the linear model, and the absolute difference is not high. We speculate that the bacterium does not thrive in an ecosystem engineered by *Blastocystis*. Another beneficial bacterium, *Faecalibacterium*, a known producer of SCFA [65] having anti-inflammatory properties [66], was also inversely associated with *Blastocystis* positivity. *Romboutsia* has been previously associated with an increased risk of nonalcoholic fatty liver disease [67] and neurodevelopmental disorders in children [68]; however, recent rigorous work found its beneficial role in cardiometabolic health [69]. All this combined makes its weak inverse association with *Blastocystis* ambiguous to interpret.

The presence of *D. fragilis* was, among others, inversely associated with *Enterobacteriaceae*, an inflammation-promoting bacteria known to be enriched in the bacteriomes of celiac disease patients [56]. On the other hand, its presence was inversely associated with many beneficial bacteria producing beneficial SCFA (including *Flavonifractor*, *Faecalibacterium* or Lachnospiraceae family), leaving the interpretation of these associations also complicated.

### Strengths and limitations

Our study provides a unique insight into a novel topic in celiac disease research as no study has yet investigated the gut parasitome by molecular techniques. The samples were taken longitudinally, which helps decrease the effects of short-term fluctuations in the abundance of individual microbial taxa [70]. Analysis was performed by several complementary methods: *Blastocystis* detection by real-time PCR assay was followed by its subtyping using amplicon sequencing of another rDNA region; in remaining parasites the positivity in the parasitome survey by massive parallel sequencing was then confirmed using real-time PCR. This, along with the inclusion of multiple negative controls, safeguarded against false positivity. Furthermore, the present study extended previous investigations to explore the whole parasitome by using multiple primer pairs [5]. Moreover, we reported the quantity of the protists, not only dichotomous positivity in analyses of subtypes but also in association with the intervention. Although absolute quantification from stool is cumbersome (as there is no internal standard for reference), we still believe that this quantitative aspect adds confidence to our findings, even though we did not find any association with the protist quantity.

The main limitation is the unavailability of a fresh fecal sample needed for direct morphological assessment by microscopy as only frozen samples were used for DNA extraction. Thus, we do not know what stages of the protist were present and whether morphology relates to the quantity of *Blastocystis* subtypes. However, such an interpretation of the microscopy result might still be difficult, given that *Blastocystis* possesses one or two nuclei [71]. Another limitation is that no background population without CDA was investigated for protist prevalence comparison as the trial was designed far earlier than this parasitome study. Thus, no control group of healthy children without CDA was used for establishing protist prevalence in the background population.

## Conclusions

The prevalence of *Blastocystis* and *D. fragilis* in children with CDA is rather low, with *D. fragilis* being the more prevalent of the two protists. Their positivity or quantity did not appreciably change upon the probiotic intervention. The presence of *Blastocystis* and *D. fragilis* was linked with an increased bacteriome diversity, although inversely associated with the abundance of some beneficial bacteria, like e.g. *Akkermansia muciniphila*. Even though the probiotics may help children with CDA to modulate the immune response and positively affect the fecal bacteriome, the single-cell parasitome remains unaffected.

### Supplementary Information

**Additional file 1.** Statistical analysis—R markdown.

**Additional file 2**: **Figure S1.** The detection primers and hydrolysis probe annealing to the consensus of several sequences of *Entamoeba* sp. **Figure S2**. Positivity of *Blastocystis *sp., *Dieantamoeba fragilis *and *Entamoeba *sp. in individual study samples. **Figure S3. **Constrained ordination (redundancy analysis) of the bacteriome community composition by *Blastocystis *sp. positivity, *Dieantamoeba fragilis* positivity and intervention with lactobacilli. **Figure S4**. The dispersion of samples negative for *Blastocystis* sp. or *Dieantamoeba fragilis *is significantly higher than that of their positive counterparts (*P* < 0.001), so testing by Permutational Multivariate Analysis of Variance would not be meaningful—its significant result may reflect not only the significant difference in centroid position but also the difference in spread. **Table S1**. Specific primers and probes for *Entamoeba *sp. quantitative PCR.

**Additional file 3.** Table—Parasites detection.

## Data Availability

Data are available upon reasonable request.

## Abbreviations

CDA: Celiac disease autoimmunity
CiPP: Celiac disease Prevention with Probiotics
qPCR: quantitative real-time PCR
PCR: Polymerase chain reaction
SCFA: Short-chain fatty acids
