# Supplementary material for: Effects of Lactiplantibacillus plantarum and Lacticaseibacillus paracasei supplementation on the single-cell fecal parasitome in children with celiac disease autoimmunity: a randomized, double-blind placebo-controlled clinical trial
Source: Parasit Vectors. 2023 Nov 9;16:411. doi: 10.1186/s13071-023-06027-1 (PMC10636941; doi:10.1186/s13071-023-06027-1)
Supplement: Supplementary file 1 — Additional file 1. Statistical analysis—R markdown. [file 13071_2023_6027_MOESM1_ESM.pdf]

CIPP bacteria and parasites

ONDREJ CINEK

The data import

The DADA2 result of the bacteriome

This is a reanalysis with a more recent taxonomic database, in order to refine the identification of the two intervention bacteria

```
in_object_fn_silva  <- "./SOURCE_DATA/dada_silva138/PS_SILVA_ASV_ARE_NUMBERED_object_WITH_TREE_final.RData"
load(in_object_fn_silva)
PS <- PS_SILVA_TREE
rm(PS_SILVA_TREE)
```

Remove non-bacterial reads

```
taxa_to_retain <- which(tax_table(PS)[, "Kingdom"] == "Bacteria")
PS <- prune_taxa(taxa = taxa_names(PS)[taxa_to_retain] , PS)
```

Add parasite data and study data to the bacteriome data, make a joint object of the Phyloseq class

```
s_data <- data.frame(sample_data(PS), stringsAsFactors = F)
s_data$fnfs <- NULL
s_data$fnrs <- NULL
s_data$filtfs <- NULL
s_data$filtrs <- NULL
s_data$original_sample_name <- sample_names(PS)
s_data$subject_no <- as.numeric(str_extract(s_data$original_sample_name, "\\d+"))
s_data$subject_f <- factor(s_data$subject_no)
s_data$time_abc <-  str_extract(s_data$original_sample_name, "[ABC]")
s_data$time_123 <- case_when(
  s_data$time_abc == "A" ~ 1,
  s_data$time_abc == "B" ~ 2,
  s_data$time_abc == "C" ~ 3
)
s_data$baseline <- factor(ifelse(s_data$time_123 == 1, "y", "n"))
s_data$sname <- paste0(sprintf("%02d", s_data$subject_no), "_", s_data$time_123)
s_data$sample <- s_data$ReactionName #for graphs - sample must be also the row name

unblinding <-read.xlsx(file = file.path(bact_dir, "SOURCE_DATA", "Unblinding_v3.xlsx"), sheetIndex = 1)
s_data <- left_join(s_data, unblinding, by = c("subject_no" = "patient_id"))
s_data$int_and_time <- paste0(s_data$study_supplement, s_data$time_123)
xtabs(~ subject_no + study_supplement, data = s_data)
```

```
##           study_supplement
## subject_no L P
##         1  0 3
##         2  0 3
##         3  3 0
##         4  0 3
##         5  3 0
##         6  0 3
##         7  0 3
##         8  3 0
##         9  3 0
##         10 0 3
##         11 3 0
##         12 3 0
##         13 3 0
##         14 0 3
##         15 3 0
##         16 3 0
##         17 0 3
##         18 0 3
##         19 3 0
##         20 0 3
##         21 3 0
##         22 3 0
##         23 0 3
```

```
##         24 0 3
##         25 3 0
##         26 0 3
##         27 3 0
##         28 0 3
##         29 3 0
##         30 3 0
##         31 0 3
##         32 3 0
##         33 3 0
##         34 3 0
##         35 3 0
##         36 3 0
##         37 0 3
##         38 0 3
##         39 3 0
##         41 3 0
##         42 3 0
##         43 0 3
##         44 3 0
##         45 3 0
##         46 0 3
##         47 0 3
##         48 3 0
##         49 0 3
##         50 0 3
##         51 3 0
##         52 3 0
##         53 0 3
##         54 0 3
##         55 0 3
##         56 0 3
##         57 3 0
##         58 3 0
##         59 3 0
##         60 0 3
##         61 3 0
##         62 0 3
##         63 0 3
##         64 3 0
##         65 0 3
##         66 0 3
##         67 0 3
##         68 0 3
##         69 3 0
##         70 3 0
##         71 2 0
##         72 0 2
##         73 2 0
##         74 2 0
##         75 0 2
##         76 0 2
##         77 0 2
##         78 0 3
##         79 3 0
```

```
parasites <- read.xlsx(file = file.path(bact_dir, "SOURCE_DATA", "cipp_parasites_metadata_final.xlsx"), sheetIndex = 1)
s_data <- left_join(
    s_data,
    select(parasites, sample_id, blastocystis_posneg, dientamoeba_posneg, entamoeba_posneg, blastocystis_pcr_concentration,
           blastocystis_subtype_result, dientamoeba_pcr_concentration),
    by = c("sample" = "sample_id")
)

s_data <- s_data %>%
    mutate(
        on_intervention = factor(if_else(study_supplement == "L" & time_123 != 1, 1, 0)),
        int_subject = paste0(study_supplement, sprintf("%02d", subject_no)),
```

```
        time_1 = if_else(time_123 == 1, 1, 0),
           time_2 = if_else(time_123 == 2, 1, 0),
           time_3 = if_else(time_123 == 3, 1, 0),
           time_23 = if_else(time_123 == 1, 0, 1),
        int_group = if_else(study_supplement == "P", 0, 1),
        int_subject_time = paste0(int_subject, ":", time_123)
    )

s_data[s_data$blastocystis_posneg == "negative", "blastocystis_pcr_concentration"] <- "0"
s_data[s_data$dientamoeba_posneg == "negative", "dientamoeba_pcr_concentration"] <- "0"

s_data <- s_data %>%
    mutate( blasto_q = as.numeric(blastocystis_pcr_concentration),
            dient_q = as.numeric(dientamoeba_pcr_concentration),
            blastocystis_pcr_concentration = as.numeric(blastocystis_pcr_concentration),
            dientamoeba_pcr_concentration  = as.numeric(dientamoeba_pcr_concentration),
            log_blastocystis_pcr = log10(blastocystis_pcr_concentration + 1),
            log_dientamoeba_pcr  = log10(dientamoeba_pcr_concentration + 1),
            bl_10 = ifelse(blastocystis_pcr_concentration>0, 1, 0),
            di_10 = ifelse(dientamoeba_pcr_concentration>0, 1, 0),
            en_10 = ifelse(dientamoeba_posneg == "positive", 1, 0),
            blasto_or_dientamoeba_pos = if_else(
                    blastocystis_posneg == "positive" | dientamoeba_posneg == "positive",
                    "positive",
                    "negative"
            ),
            blastocystis_or_dientamoeba = case_when(
                bl_10 == 1 & di_10 == 1 ~ "both positive",
                bl_10 == 1 & di_10 == 0 ~ "blasto positive",
                bl_10 == 0 & di_10 == 1 ~ "dient positive",
                bl_10 == 0 & di_10 == 0 ~ "NEG"
            ),
            any_parasite_pos = if_else(
                blastocystis_posneg == "positive" | dientamoeba_posneg == "positive" | entamoeba_posneg == "positive",
                "positive",
                "negative"
            ),

            any_10 = ifelse(bl_10 + di_10 + en_10 > 0, 1,0)
           )



s_data <- s_data %>%
    select(-any_of(c("basename", "position_beh_sname", "ntc",
                     "standards", "reads_in_fastq", "reads_after_filtering",
                     "analyse_sample", "dada_f", "dada_r", "merged",
                     "nonchim", "our_sample_names", "number",
                     "abc", "original_sample_name")))



row.names(s_data) <- s_data$ReactionName
sample_data(PS) <- sample_data(s_data)

# check for internal consistency
xtabs(~ intervention + time_123 + on_intervention, data = s_data)
```

```
## , , on_intervention = 0
##
##                time_123
## intervention    1  2  3
##   lactobacilus 40  0  0
##   placebo      37 37 36
##
## , , on_intervention = 1
##
##                time_123
```

```
## intervention    1  2  3
##   lactobacilus  0 39 38
##   placebo       0  0  0
```

Rarefaction

```
rarecurve(otu_table(PS), step=500, cex=0.5)
```

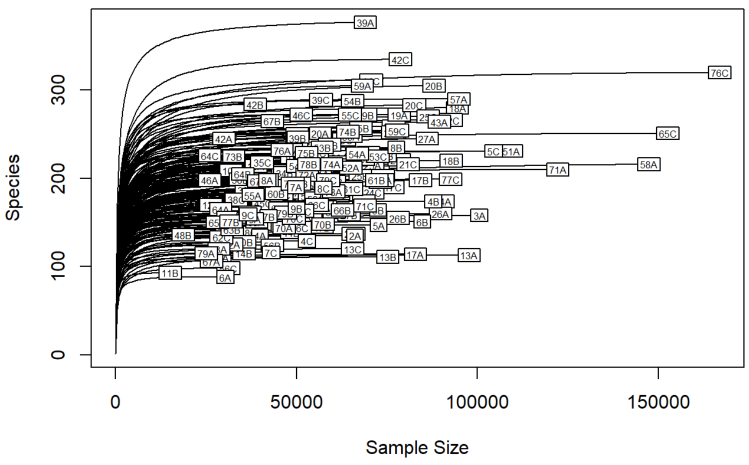


```
graph_of_reads_frequency(PS, "before rarefaction")
```

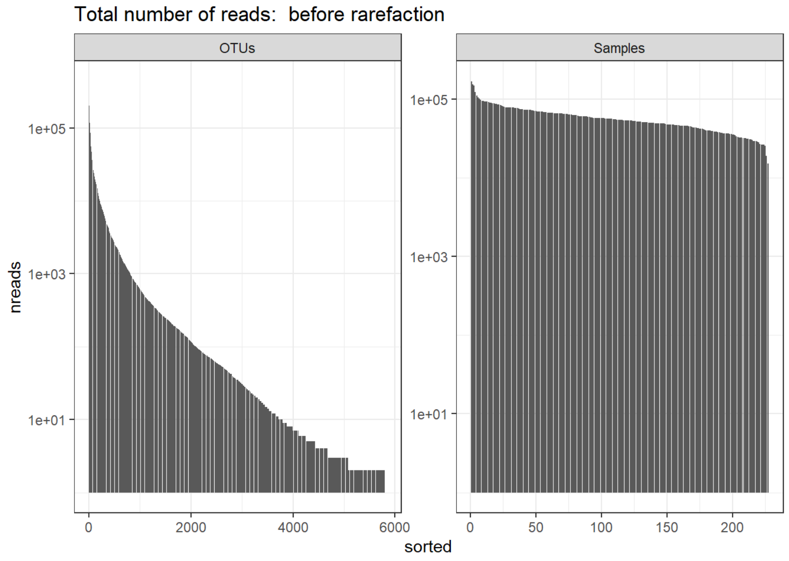


```
hist(log(sample_sums(PS),10))
```

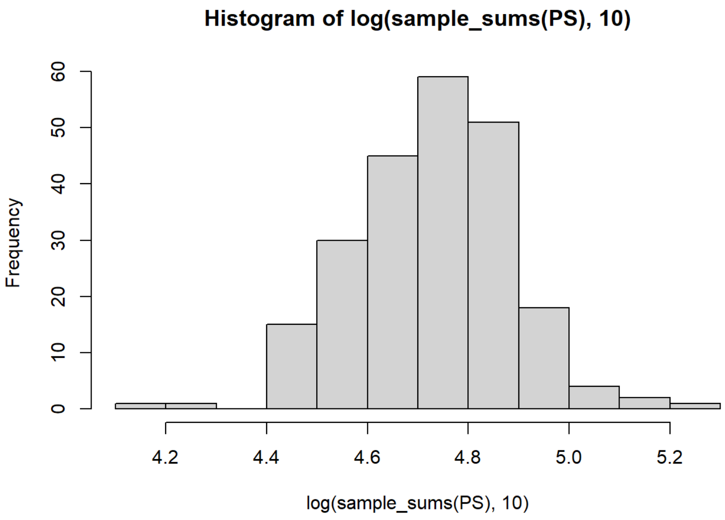

```
sort(sample_sums(PS))[1:40]
```

```
##   11B   48B   79A   64C   46A   12B   67A   65B   63A   11A   64A   62C   42A
## 15175 18813 25135 26080 26133 26402 26614 28175 28617 28972 29125 29284 30005
##    6A   22A   65A   56C   37C   77B   10B   62A   63B   63C   73B   38C   38B
## 30410 30736 31095 31227 31727 31957 31961 32074 32279 32384 32710 33429 34535
##   11C   64B   14B   50B   33C   37B    3B   68B    9C   48C   73A   68C   62B
## 35104 35266 35574 35680 36200 36395 36476 36594 36654 36728 37156 37767 37891
##   55A
## 38050
```

Count of reads: at the ASV level, rarefy and make a version of object with a abundance filter

```
min_reads <- 20
min_samples <- 2

RAR <- rarefy_even_depth(PS, sample.size = 15000, rngseed = 1000, replace = F)
```

```
## `set.seed(1000)` was used to initialize repeatable random subsampling.
```

```
## Please record this for your records so others can reproduce.
```

```
## Try `set.seed(1000); .Random.seed` for the full vector
```

```
## ...
```

```
## 791OTUs were removed because they are no longer
## present in any sample after random subsampling
```

```
## ...
```

```
graph_of_reads_frequency(RAR, "rarefied to 15,000")
```

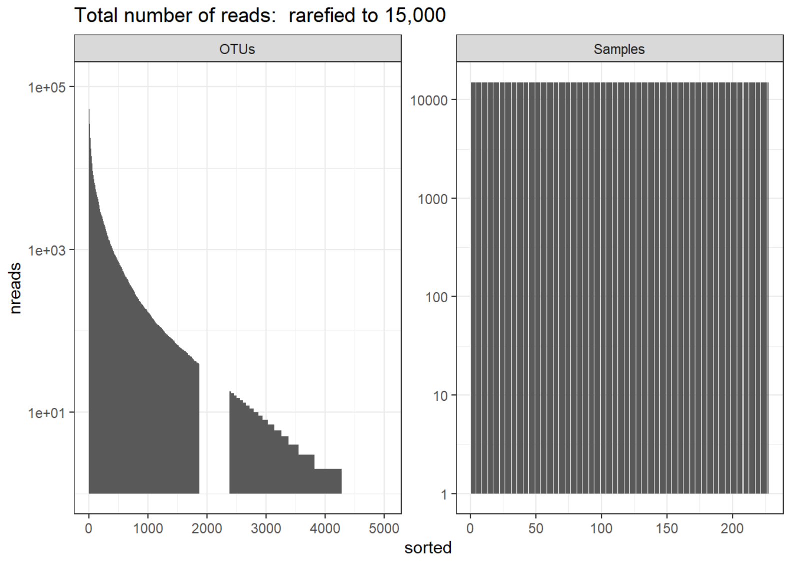


```
RAR_FILT <- filter_taxa(RAR, function(x) sum(x > min_reads) > min_samples, TRUE)
RAR_FILT <- rarefy_even_depth(RAR_FILT, sample.size = 10000, rngseed = 1000, replace = F)
```

```
## `set.seed(1000)` was used to initialize repeatable random subsampling.
```

```
## Please record this for your records so others can reproduce.
```

```
## Try `set.seed(1000); .Random.seed` for the full vector
```

```
## ...
```

Also tax-glom to phylum and down to genus + filter for abundance

```
PSMR_P <- tax_glom(PS, taxrank = "Phylum")
PSMR_P <- filter_taxa(PSMR_P, function(x) sum(x > min_reads) > floor(min_samples), TRUE)
sort(sample_sums(PSMR_P))[1:10]
```

```
##   11B   48B   79A   64C   46A   12B   67A   65B   63A   11A
## 15175 18813 25135 26080 26133 26402 26614 28175 28617 28972
```

```
PSMR_P <- rarefy_even_depth(PSMR_P, sample.size = 15000, rngseed = 7000, replace = F)
```

```
## `set.seed(7000)` was used to initialize repeatable random subsampling.
```

```
## Please record this for your records so others can reproduce.
```

```
## Try `set.seed(7000); .Random.seed` for the full vector
```

```
## ...
```

```
PSMR_C <- tax_glom(PS, taxrank = "Class")
PSMR_C <- filter_taxa(PSMR_C, function(x) sum(x > min_reads) > floor(min_samples), TRUE)
sort(sample_sums(PSMR_C))[1:10]
```

```
##   11B   48B   79A   64C   46A   12B   67A   65B   63A   11A
## 15175 18813 25135 26080 26133 26402 26614 28175 28617 28972
```

```
PSMR_C <- rarefy_even_depth(PSMR_C, sample.size = 15000, rngseed = 7000, replace = F)
```

```
## `set.seed(7000)` was used to initialize repeatable random subsampling.
```

```
## Please record this for your records so others can reproduce.
```

```
## Try `set.seed(7000); .Random.seed` for the full vector
```

```
## ...
```

```
PSMR_O <- tax_glom(PS, taxrank = "Order")
PSMR_O <- filter_taxa(PSMR_O, function(x) sum(x > min_reads) > floor(min_samples), TRUE)
sort(sample_sums(PSMR_O))[1:10]
```

```
##   11B   48B   79A   64C   46A   12B   67A   65B   63A   11A
## 15175 18785 25135 26080 26133 26402 26614 28175 28617 28972
```

```
PSMR_O <- rarefy_even_depth(PSMR_O, sample.size = 15000, rngseed = 7000, replace = F)
```

```
## `set.seed(7000)` was used to initialize repeatable random subsampling.
```

```
## Please record this for your records so others can reproduce.
```

```
## Try `set.seed(7000); .Random.seed` for the full vector
```

```
## ...
```

```
PSMR_F <- tax_glom(PS, taxrank = "Family")
PSMR_F <- filter_taxa(PSMR_F, function(x) sum(x > min_reads) > floor(min_samples), TRUE)
sort(sample_sums(PSMR_F))[1:10]
```

```
##   11B   48B   79A   46A   64C   12B   67A   65B   63A   11A
## 15025 18760 25133 25820 25824 26185 26614 28175 28304 28588
```

```
PSMR_F <- rarefy_even_depth(PSMR_F, sample.size = 15000, rngseed = 7000, replace = F)
```

```
## `set.seed(7000)` was used to initialize repeatable random subsampling.
```

```
## Please record this for your records so others can reproduce.
```

```
## Try `set.seed(7000); .Random.seed` for the full vector
```

```
## ...
```

```
PSMR_G <- tax_glom(PS, taxrank = "Genus")
PSMR_G <- filter_taxa(PSMR_G, function(x) sum(x > min_reads) > floor(min_samples), TRUE)
sort(sample_sums(PSMR_G))[1:10]
```

```
##   11B   48B   79A   64C   46A   12B   67A   65B   42A   63A
## 14844 18137 25081 25476 25521 25856 26583 27946 28045 28245
```

```
PSMR_G <- rarefy_even_depth(PSMR_G, sample.size = 10000 , rngseed = 7000, replace = F)
```

```
## `set.seed(7000)` was used to initialize repeatable random subsampling.
```

```
## Please record this for your records so others can reproduce.
```

```
## Try `set.seed(7000); .Random.seed` for the full vector
```

```
## ...
```

Some exploration

Are the intervention microbes there?

```
ttable <- data.frame(tax_table(RAR))
ttable$asv <- rownames(ttable)
print("Lacti something ASVs:")
```

```
## [1] "Lacti something ASVs:"
```

```
(lacti_something_genera <- ttable[!is.na(ttable$Genus) & str_detect(ttable$Genus, "Lacti.*bacillus"),])
```

```
##            Kingdom     Phylum   Class           Order           Family
## ASV00796 Bacteria Firmicutes Bacilli Lactobacillales Lactobacillaceae
## ASV01726 Bacteria Firmicutes Bacilli Lactobacillales Lactobacillaceae
## ASV04262 Bacteria Firmicutes Bacilli Lactobacillales Lactobacillaceae
```

```
## ASV04590 Bacteria Firmicutes Bacilli Lactobacillales Lactobacillaceae
## ASV00855 Bacteria Firmicutes Bacilli Lactobacillales Lactobacillaceae
## ASV02150 Bacteria Firmicutes Bacilli Lactobacillales Lactobacillaceae
## ASV02832 Bacteria Firmicutes Bacilli Lactobacillales Lactobacillaceae
## ASV02903 Bacteria Firmicutes Bacilli Lactobacillales Lactobacillaceae
## ASV01874 Bacteria Firmicutes Bacilli Lactobacillales Lactobacillaceae
##                        Genus Species      asv
## ASV00796 Lactiplantibacillus    <NA> ASV00796
## ASV01726 Lactiplantibacillus    <NA> ASV01726
## ASV04262 Lactiplantibacillus    <NA> ASV04262
## ASV04590  Lacticaseibacillus    <NA> ASV04590
## ASV00855  Lacticaseibacillus    <NA> ASV00855
## ASV02150  Lacticaseibacillus    <NA> ASV02150
## ASV02832  Lacticaseibacillus    <NA> ASV02832
## ASV02903  Lacticaseibacillus    <NA> ASV02903
## ASV01874  Lacticaseibacillus    <NA> ASV01874
```

```
lacti_something_bacillus_asv <- rownames(lacti_something_genera)

print("Lactiplantibacillus ASVs:")
```

```
## [1] "Lactiplantibacillus ASVs:"
```

```
(lactiplantibaciuulus_genera <- ttable[!is.na(ttable$Genus) & str_detect(ttable$Genus, "Lactiplantibacillus"),])
```

```
##           Kingdom     Phylum   Class           Order           Family
## ASV00796 Bacteria Firmicutes Bacilli Lactobacillales Lactobacillaceae
## ASV01726 Bacteria Firmicutes Bacilli Lactobacillales Lactobacillaceae
## ASV04262 Bacteria Firmicutes Bacilli Lactobacillales Lactobacillaceae
##                        Genus Species      asv
## ASV00796 Lactiplantibacillus    <NA> ASV00796
## ASV01726 Lactiplantibacillus    <NA> ASV01726
## ASV04262 Lactiplantibacillus    <NA> ASV04262
```

```
lactiplantibacillus_asv <- rownames(lactiplantibaciuulus_genera)

print("casei something ASVs:")
```

```
## [1] "casei something ASVs:"
```

```
(lacticasei_genera <- ttable[(!is.na(ttable$Species) & str_detect(ttable$Species, "casei")) |
                             (!is.na(ttable$Genus) & str_detect(ttable$Genus, "casei")) , ])
```

```
##           Kingdom     Phylum   Class           Order           Family
## ASV04590 Bacteria Firmicutes Bacilli Lactobacillales Lactobacillaceae
## ASV00855 Bacteria Firmicutes Bacilli Lactobacillales Lactobacillaceae
## ASV02150 Bacteria Firmicutes Bacilli Lactobacillales Lactobacillaceae
## ASV02832 Bacteria Firmicutes Bacilli Lactobacillales Lactobacillaceae
## ASV02903 Bacteria Firmicutes Bacilli Lactobacillales Lactobacillaceae
## ASV01874 Bacteria Firmicutes Bacilli Lactobacillales Lactobacillaceae
##                       Genus Species      asv
## ASV04590 Lacticaseibacillus    <NA> ASV04590
## ASV00855 Lacticaseibacillus    <NA> ASV00855
## ASV02150 Lacticaseibacillus    <NA> ASV02150
## ASV02832 Lacticaseibacillus    <NA> ASV02832
## ASV02903 Lacticaseibacillus    <NA> ASV02903
## ASV01874 Lacticaseibacillus    <NA> ASV01874
```

```
lacticasei_asv <- rownames(lacticasei_genera)
```

Find and characterize the most abundant otus

```
taxa_sums_by_otu <- sort(taxa_sums(RAR), decreasing = TRUE)
plot(log10(taxa_sums_by_otu))
```

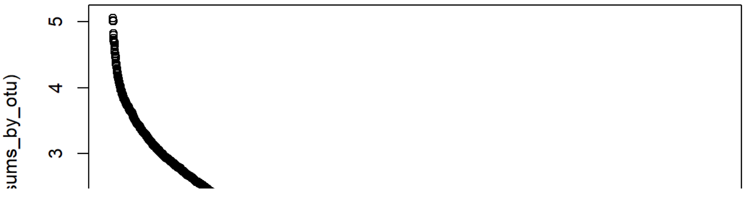

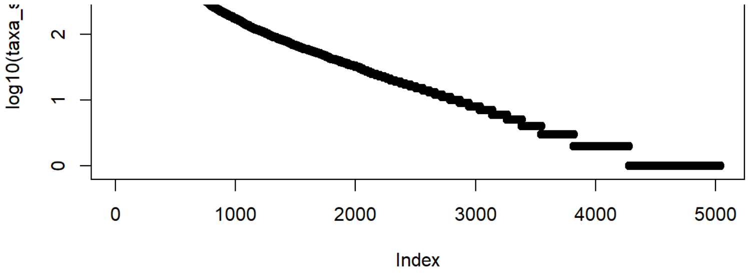


```
otus_over_10_reads <- taxa_sums_by_otu[taxa_sums_by_otu > 10]
taxonomic_assignation_otus_over_10_reads <- tax_table(RAR)[names(otus_over_10_reads), 2:7]
otu_sequences <- as.data.frame(refseq(RAR))
taxonomic_assignation_otus_over_10_reads <- cbind(taxonomic_assignation_otus_over_10_reads, quantity = floor(otus_over_10_reads),
sequence = otu_sequences[names(otus_over_10_reads), ])

sequences_of_otus_over_10_reads <- otu_sequences[names(otus_over_10_reads), ]

xlsx::write.xlsx(
    taxonomic_assignation_otus_over_10_reads,
    file = "graphs_and_outputs/taxonomic_assignation_otus_over_10_rr.xlsx"
)

taxa_sums_by_genus <- sort(taxa_sums(PSMR_G), decreasing = TRUE)
plot(log10(taxa_sums_by_genus))
```

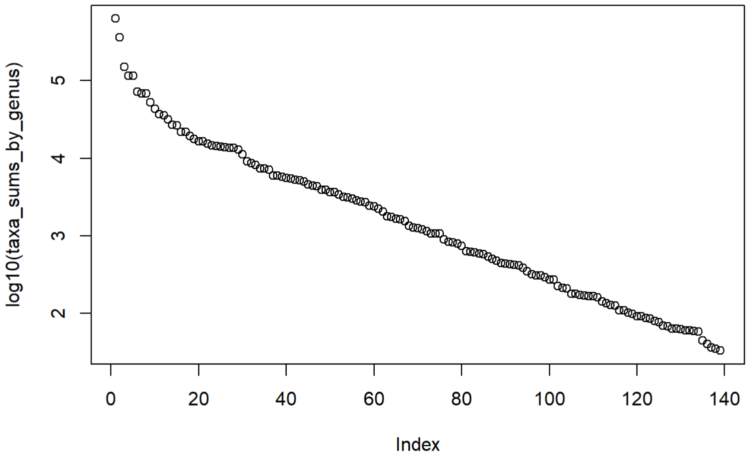


```
taxonomic_assignation_genera <- tax_table(PSMR_G)[names(taxa_sums_by_genus), 2:6]
genus_sequences <- as.data.frame(refseq(PSMR_G))
taxonomic_assignation_genera <- cbind(taxonomic_assignation_genera, quantity = floor(taxa_sums_by_genus), sequence =
genus_sequences[names(taxonomic_assignation_genera), ])

xlsx::write.xlsx(
    taxonomic_assignation_genera,
    file = "graphs_and_outputs/taxonomic_assignation_genera.xlsx"
)
```

Some abundance plots by the intervention and time point

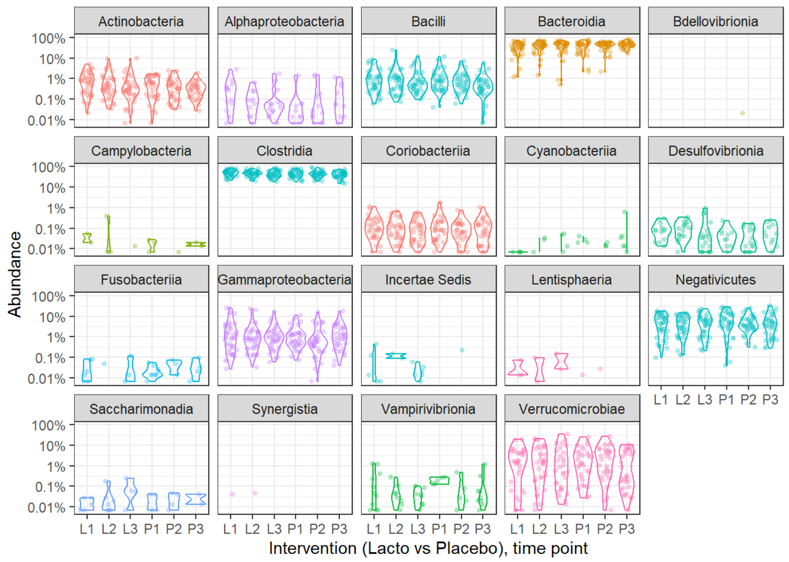

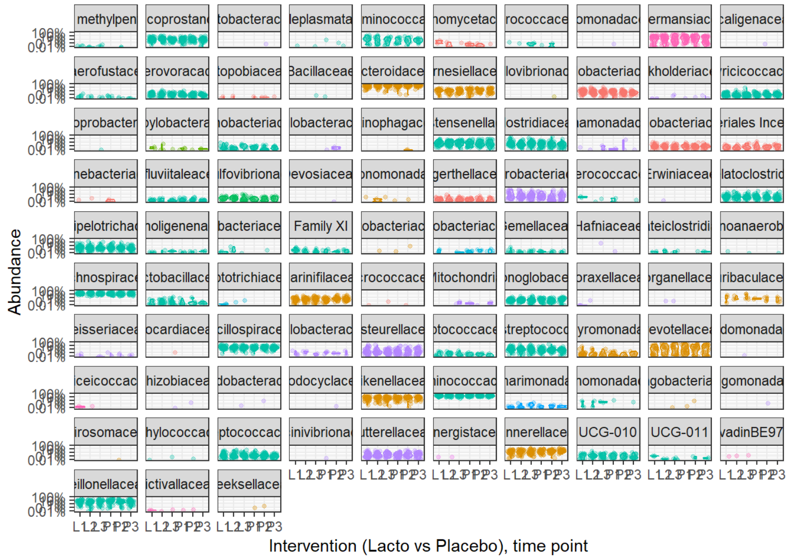


```
## no taxon ordering[1] "Abundance ~ Sample + int_and_time + Phylum"
```

```
## Scale for y is already present.
## Adding another scale for y, which will replace the existing scale.
```

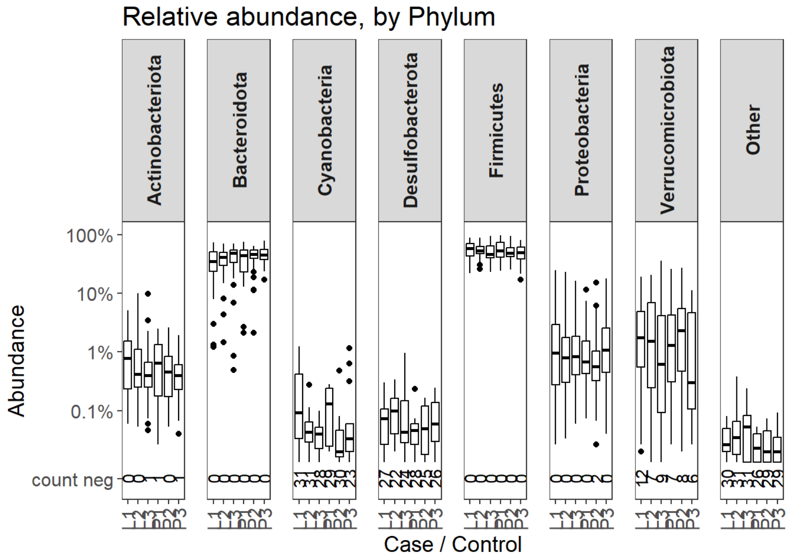

Plotting samples from the same individuals

save-load point

```
save.image(file = "./saved_data/cipp_intermediate01.RData")
#load(file = "./saved_data/cipp_intermediate01.RData")
```

ALPHA DIVERSITY

Alpha diversity by time and intervention, graphs. Following the advice by Reviewer 1, this is done at the genus level get an untrimmed rarefied data set agglomerated at the genus level, then make a data frame with the indices.

```
UNTR_G <- rarefy_even_depth(
  tax_glom(PS, taxrank = "Genus"),
  sample.size = 10000 ,
  rngseed = 7000,
  replace = F
)
```

```
## `set.seed(7000)` was used to initialize repeatable random subsampling.
```

```
## Please record this for your records so others can reproduce.
```

```
## Try `set.seed(7000); .Random.seed` for the full vector
```

```
## ...
```

```
## 14OTUs were removed because they are no longer
## present in any sample after random subsampling
```

```
## ...
```

```
rich_estim <- phyloseq::estimate_richness(UNTR_G)
rich_estim$sample <- substr(row.names(rich_estim), 2,99)
rich_estim <- left_join(data.frame(sample_data(UNTR_G), stringsAsFactors = F), rich_estim, by = "sample")
```

Alpha diversity indices that will be tested

```
alpha_diversity_measures = c("Observed", "Chao1", "ACE", "Shannon", "Simpson", "InvSimpson", "Fisher")
```

As reported previously from this study, no difference by intervention & time point (a rough test not respecting the repeated sampling, i.e. more prone to false positivity - yet still no association)

```
plot_richness(UNTR_G, "int_and_time", measures = alpha_diversity_measures) + geom_boxplot( aes(color = NULL)  )
```

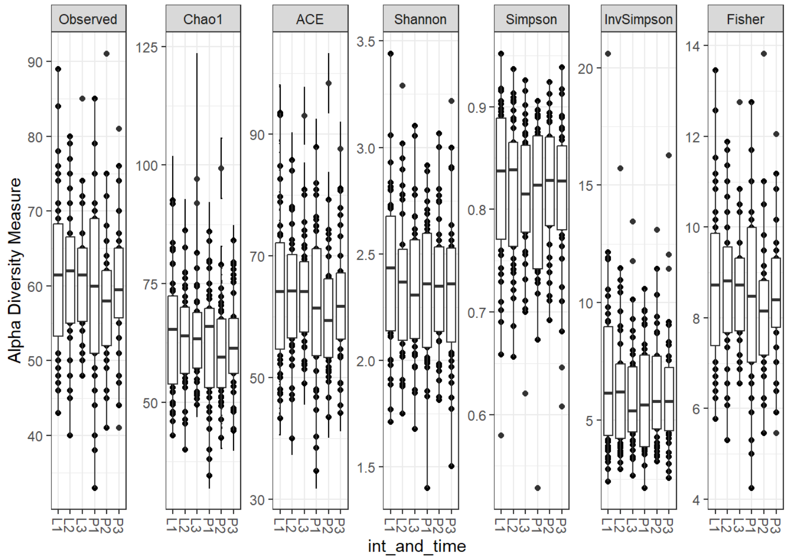


```
summary( aov(Chao1   ~int_and_time, data = rich_estim))
```

```
##               Df Sum Sq Mean Sq F value Pr(>F)
## int_and_time   5    397   79.36   0.616  0.688
## Residuals    221  28469  128.82
```

```
TukeyHSD(aov(Chao1   ~int_and_time, data = rich_estim), "int_and_time", ordered = F)
```

```
##   Tukey multiple comparisons of means
##     95% family-wise confidence level
##
## Fit: aov(formula = Chao1 ~ int_and_time, data = rich_estim)
##
## $int_and_time
##             diff        lwr      upr     p adj
## L2-L1 -1.0461040  -8.388874 6.296666 0.9985069
## L3-L1 -0.2457414  -7.637269 7.145786 0.9999989
## P1-L1 -2.6209599 -10.063535 4.821615 0.9134535
## P2-L1 -3.5862323 -11.028807 3.856343 0.7358690
## P3-L1 -2.4881056  -9.984186 5.007975 0.9316237
```

```
## L3-L2  0.8003626  -6.637189 8.237914 0.9996177
## P1-L2 -1.5748559  -9.063140 5.913429 0.9906246
## P2-L2 -2.5401283 -10.028413 4.948156 0.9253861
## P3-L2 -1.4420016  -8.983468 6.099464 0.9939676
## P1-L3 -2.3752185  -9.911319 5.160882 0.9446440
## P2-L3 -3.3404909 -10.876591 4.195610 0.7987342
## P3-L3 -2.2423641  -9.831311 5.346583 0.9577230
## P2-P1 -0.9652724  -8.551447 6.620902 0.9991352
## P3-P1  0.1328543  -7.505820 7.771529 1.0000000
## P3-P2  1.0981267  -6.540548 8.736802 0.9984407
```

```
summary( aov(Shannon ~int_and_time, data = rich_estim))
```

```
##               Df Sum Sq Mean Sq F value Pr(>F)
## int_and_time   5  0.365 0.07295   0.609  0.693
## Residuals    221 26.489 0.11986
```

```
TukeyHSD(aov(Shannon ~int_and_time, data = rich_estim), "int_and_time", ordered = F)
```

```
##   Tukey multiple comparisons of means
##     95% family-wise confidence level
##
## Fit: aov(formula = Shannon ~ int_and_time, data = rich_estim)
##
## $int_and_time
##               diff        lwr       upr     p adj
## L2-L1 -0.051748002 -0.2757278 0.1722318 0.9856108
## L3-L1 -0.093103896 -0.3185710 0.1323632 0.8426646
## P1-L1 -0.116912900 -0.3439371 0.1101113 0.6770809
## P2-L1 -0.075505738 -0.3025300 0.1515185 0.9310667
## P3-L1 -0.109155664 -0.3378120 0.1195007 0.7435059
## L3-L2 -0.041355894 -0.2682269 0.1855151 0.9951763
## P1-L2 -0.065164897 -0.2935834 0.1632536 0.9635814
## P2-L2 -0.023757736 -0.2521763 0.2046608 0.9996764
## P3-L2 -0.057407662 -0.2874484 0.1726331 0.9796786
## P1-L3 -0.023809003 -0.2536861 0.2060681 0.9996830
## P2-L3  0.017598158 -0.2122789 0.2474752 0.9999285
## P3-L3 -0.016051767 -0.2475408 0.2154373 0.9999562
## P2-P1  0.041407161 -0.1899973 0.2728117 0.9955790
## P3-P1  0.007757236 -0.2252487 0.2407632 0.9999989
## P3-P2 -0.033649926 -0.2666559 0.1993560 0.9984061
```

```
summary( aov(Simpson ~int_and_time, data = rich_estim))
```

```
##               Df Sum Sq  Mean Sq F value Pr(>F)
## int_and_time   5  0.007 0.001390   0.263  0.933
## Residuals    221  1.169 0.005289
```

```
TukeyHSD(aov(Simpson ~int_and_time, data = rich_estim), "int_and_time", ordered = F)
```

```
##   Tukey multiple comparisons of means
##     95% family-wise confidence level
##
## Fit: aov(formula = Simpson ~ int_and_time, data = rich_estim)
##
## $int_and_time
##                diff         lwr        upr     p adj
## L2-L1 -0.0004445401 -0.04749314 0.04660406 1.0000000
## L3-L1 -0.0071564860 -0.05451749 0.04020452 0.9980194
## P1-L1 -0.0132607079 -0.06094880 0.03442738 0.9673733
## P2-L1  0.0035945116 -0.04409358 0.05128260 0.9999337
## P3-L1 -0.0059845848 -0.05401551 0.04204634 0.9992189
## L3-L2 -0.0067119459 -0.05436785 0.04094396 0.9985871
## P1-L2 -0.0128161678 -0.06079714 0.03516481 0.9725935
## P2-L2  0.0040390517 -0.04394192 0.05202003 0.9998856
## P3-L2 -0.0055400447 -0.05386178 0.04278169 0.9994791
## P1-L3 -0.0061042219 -0.05439158 0.04218313 0.9991621
## P2-L3  0.0107509976 -0.03753636 0.05903835 0.9878324
## P3-L3  0.0011719011 -0.04745407 0.04979787 0.9999998
## P2-P1  0.0168552195 -0.03175298 0.06546342 0.9185671
## P3-P1  0.0072761230 -0.04166847 0.05622072 0.9981683
## P3-P2 -0.0095790964 -0.05852369 0.03936550 0.9932769
```

Figure 4 - Alpha diversity by positivity for protists

Differences are notable.

```
x_lab_expression <- expression(paste("Positivity for ", italic("Blastocystis"), " or ", italic("Dientamoeba")))

p_figure4 <- plot_richness(UNTR_G, "blasto_or_dientamoeba_pos", measures = alpha_diversity_measures) + geom_boxplot( aes(color =
NULL)) + xlab(x_lab_expression)

print(p_figure4)
```

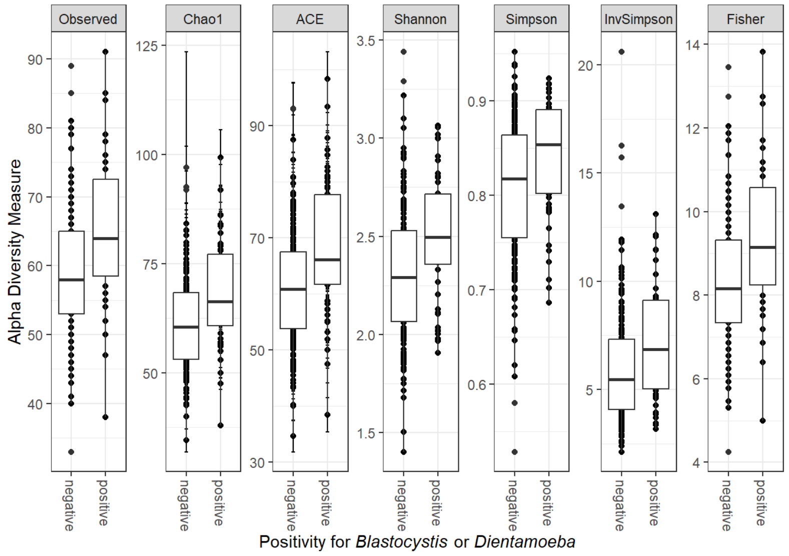


```
ggsave(filename = paste0("Figure 4 revision 2.pdf"),
       plot = p_figure4,
       #width = 8.27, height = 11.69, #A4 portrait
       width = 11.69*0.7, height = 8.27*0.7, #A4 landscape * 0.7
       device = "pdf",
       path = "./graphs_and_outputs"
       )

plot_richness(UNTR_G, "dientamoeba_posneg" ) +
  geom_boxplot( aes(color = NULL)  ) +
  labs(x = expression(paste("Positivity for ", italic("Dieantamoeba"))))
```

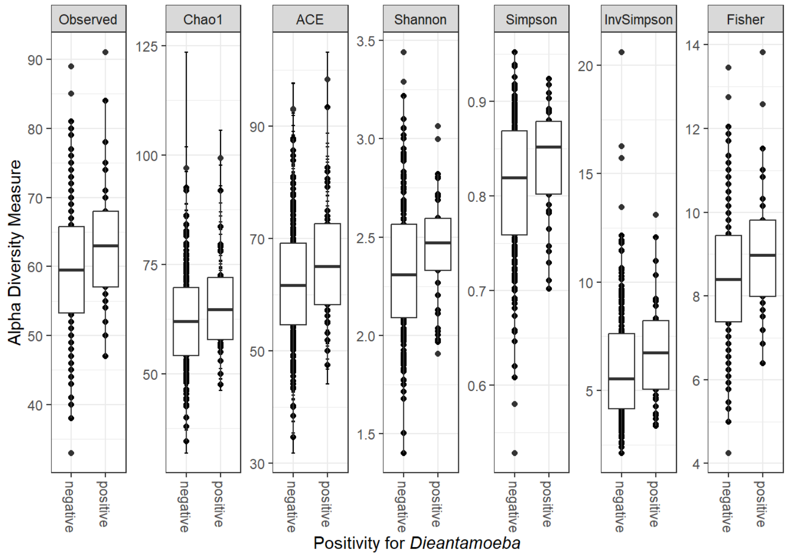


```
plot_richness(UNTR_G, "blastocystis_posneg" ) +
  geom_boxplot( aes(color = NULL)  ) +
  labs(x = expression(paste("Positivity for ", italic("Blastocystis"))))
```

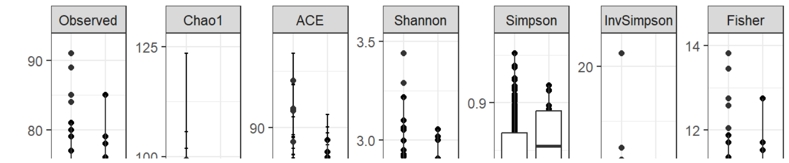

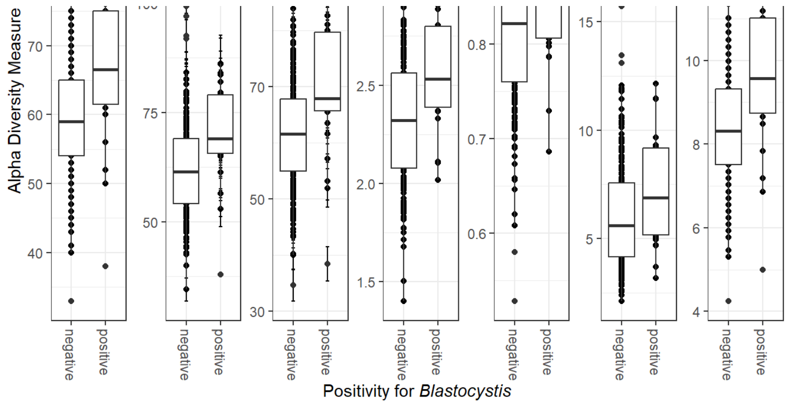

Formal testing using GEE for the association of alpha diversity with quantity or positivity of protists

the predictor = parasite quantity or positivity Parasites quantitatively predictors and alpha diversity - clear and strong association of both parasites with stool diversity. The latter p value will go into Figure 4 above.

```
for (my_measure in alpha_diversity_measures){
  print("---------------------------------")
  print(my_measure)
  print("---------------------------------")
  gee_res <- geepack::geeglm(
          formula = formula(paste0(my_measure, " ~ log_blastocystis_pcr + log_dientamoeba_pcr + on_intervention")),
          id = subject_no,
          data = rich_estim,
          family = gaussian,
          corstr = "ar1",
          contrasts = NULL
          )
  print(summary(gee_res))

  # These are the P values reported in Figure 4; the model respects the repeated sampling and is adjusted for the intervention
  gee_res2 <- geepack::geeglm(
            formula = formula(paste0(my_measure, " ~ blasto_or_dientamoeba_pos  + on_intervention")),
            id = subject_no,
            data = rich_estim,
            family = gaussian,
            corstr = "ar1",
            contrasts = NULL
            )
  print(summary(gee_res2))
}
```

```
## [1] "---------------------------------"
## [1] "Observed"
## [1] "---------------------------------"
##
## Call:
## geepack::geeglm(formula = formula(paste0(my_measure, " ~ log_blastocystis_pcr + log_dientamoeba_pcr + on_intervention")),
##     family = gaussian, data = rich_estim, contrasts = NULL, id = subject_no,
##     corstr = "ar1")
##
##  Coefficients:
##                      Estimate Std.err     Wald Pr(>|W|)
## (Intercept)           59.4122  1.0717 3073.577  < 2e-16 ***
## log_blastocystis_pcr   2.3409  0.8095    8.363  0.00383 **
## log_dientamoeba_pcr    0.2786  0.3830    0.529  0.46703
## on_intervention1       0.8848  1.1561    0.586  0.44405
## ---
## Signif. codes:  0 '***' 0.001 '**' 0.01 '*' 0.05 '.' 0.1 ' ' 1
##
## Correlation structure = ar1
## Estimated Scale Parameters:
##
##             Estimate Std.err
## (Intercept)    89.88   10.97
##   Link = identity
```

```
##
## Estimated Correlation Parameters:
##       Estimate Std.err
## alpha    0.475 0.07825
## Number of clusters:   78  Maximum cluster size: 3
##
## Call:
## geepack::geeglm(formula = formula(paste0(my_measure, " ~ blasto_or_dientamoeba_pos  + on_intervention")),
##     family = gaussian, data = rich_estim, contrasts = NULL, id = subject_no,
##     corstr = "ar1")
##
##  Coefficients:
##                                    Estimate Std.err    Wald Pr(>|W|)
## (Intercept)                          58.938   1.050 3148.73   <2e-16 ***
## blasto_or_dientamoeba_pospositive     4.952   2.089    5.62    0.018 *
## on_intervention1                      0.915   1.152    0.63    0.427
## ---
## Signif. codes:  0 '***' 0.001 '**' 0.01 '*' 0.05 '.' 0.1 ' ' 1
##
## Correlation structure = ar1
## Estimated Scale Parameters:
##
##             Estimate Std.err
## (Intercept)       89    10.5
##   Link = identity
##
## Estimated Correlation Parameters:
##       Estimate Std.err
## alpha    0.466  0.0803
## Number of clusters:   78  Maximum cluster size: 3
## [1] "----------------------------------"
## [1] "Chao1"
## [1] "----------------------------------"
##
## Call:
## geepack::geeglm(formula = formula(paste0(my_measure, " ~ log_blastocystis_pcr + log_dientamoeba_pcr + on_intervention")),
##     family = gaussian, data = rich_estim, contrasts = NULL, id = subject_no,
##     corstr = "ar1")
##
##  Coefficients:
##                      Estimate Std.err    Wald Pr(>|W|)
## (Intercept)            62.137   1.178 2783.17   <2e-16 ***
## log_blastocystis_pcr    2.487   1.007    6.10    0.014 *
## log_dientamoeba_pcr     0.309   0.460    0.45    0.501
## on_intervention1        0.496   1.356    0.13    0.715
## ---
## Signif. codes:  0 '***' 0.001 '**' 0.01 '*' 0.05 '.' 0.1 ' ' 1
##
## Correlation structure = ar1
## Estimated Scale Parameters:
##
##             Estimate Std.err
## (Intercept)      120    13.2
##   Link = identity
##
## Estimated Correlation Parameters:
##       Estimate Std.err
## alpha    0.388  0.0875
## Number of clusters:   78  Maximum cluster size: 3
##
## Call:
## geepack::geeglm(formula = formula(paste0(my_measure, " ~ blasto_or_dientamoeba_pos  + on_intervention")),
##     family = gaussian, data = rich_estim, contrasts = NULL, id = subject_no,
##     corstr = "ar1")
##
##  Coefficients:
##                                    Estimate Std.err    Wald Pr(>|W|)
```

```
## (Intercept)                         61.509   1.152 2851.76   <2e-16 ***
## blasto_or_dientamoeba_pospositive    5.727   2.303    6.18    0.013 *
## on_intervention1                     0.588   1.346    0.19    0.662
## ---
## Signif. codes:  0 '***' 0.001 '**' 0.01 '*' 0.05 '.' 0.1 ' ' 1
##
## Correlation structure = ar1
## Estimated Scale Parameters:
##
##             Estimate Std.err
## (Intercept)      119    12.6
##   Link = identity
##
## Estimated Correlation Parameters:
##       Estimate Std.err
## alpha    0.381  0.0891
## Number of clusters:   78  Maximum cluster size: 3
## [1] "-----------------------------------"
## [1] "ACE"
## [1] "-----------------------------------"
##
## Call:
## geepack::geeglm(formula = formula(paste0(my_measure, " ~ log_blastocystis_pcr + log_dientamoeba_pcr + on_intervention")),
##     family = gaussian, data = rich_estim, contrasts = NULL, id = subject_no,
##     corstr = "ar1")
##
##  Coefficients:
##                      Estimate Std.err    Wald Pr(>|W|)
## (Intercept)            62.151   1.159 2874.38   <2e-16 ***
## log_blastocystis_pcr    2.531   1.014    6.23    0.013 *
## log_dientamoeba_pcr     0.354   0.467    0.57    0.449
## on_intervention1        0.405   1.263    0.10    0.748
## ---
## Signif. codes:  0 '***' 0.001 '**' 0.01 '*' 0.05 '.' 0.1 ' ' 1
##
## Correlation structure = ar1
## Estimated Scale Parameters:
##
##             Estimate Std.err
## (Intercept)      113    12.8
##   Link = identity
##
## Estimated Correlation Parameters:
##       Estimate Std.err
## alpha    0.423  0.0788
## Number of clusters:   78  Maximum cluster size: 3
##
## Call:
## geepack::geeglm(formula = formula(paste0(my_measure, " ~ blasto_or_dientamoeba_pos  + on_intervention")),
##     family = gaussian, data = rich_estim, contrasts = NULL, id = subject_no,
##     corstr = "ar1")
##
##  Coefficients:
##                                   Estimate Std.err    Wald Pr(>|W|)
## (Intercept)                         61.585   1.140 2920.05   <2e-16 ***
## blasto_or_dientamoeba_pospositive    5.682   2.311    6.05    0.014 *
## on_intervention1                     0.471   1.259    0.14    0.708
## ---
## Signif. codes:  0 '***' 0.001 '**' 0.01 '*' 0.05 '.' 0.1 ' ' 1
##
## Correlation structure = ar1
## Estimated Scale Parameters:
##
##             Estimate Std.err
## (Intercept)      111    12.2
##   Link = identity
##
```

```
## Estimated Correlation Parameters:
##       Estimate Std.err
## alpha    0.414   0.081
## Number of clusters:   78  Maximum cluster size: 3
## [1] "---------------------------------"
## [1] "Shannon"
## [1] "---------------------------------"
##
## Call:
## geepack::geeglm(formula = formula(paste0(my_measure, " ~ log_blastocystis_pcr + log_dientamoeba_pcr + on_intervention")),
##     family = gaussian, data = rich_estim, contrasts = NULL, id = subject_no,
##     corstr = "ar1")
##
##  Coefficients:
##                      Estimate Std.err    Wald Pr(>|W|)
## (Intercept)            2.3441  0.0392 3567.61   <2e-16 ***
## log_blastocystis_pcr   0.0655  0.0283    5.36    0.021 *
## log_dientamoeba_pcr    0.0123  0.0126    0.96    0.328
## on_intervention1      -0.0324  0.0438    0.54    0.461
## ---
## Signif. codes:  0 '***' 0.001 '**' 0.01 '*' 0.05 '.' 0.1 ' ' 1
##
## Correlation structure = ar1
## Estimated Scale Parameters:
##
##             Estimate Std.err
## (Intercept)    0.113  0.0141
##   Link = identity
##
## Estimated Correlation Parameters:
##       Estimate Std.err
## alpha      0.6  0.0709
## Number of clusters:   78  Maximum cluster size: 3
##
## Call:
## geepack::geeglm(formula = formula(paste0(my_measure, " ~ blasto_or_dientamoeba_pos  + on_intervention")),
##     family = gaussian, data = rich_estim, contrasts = NULL, id = subject_no,
##     corstr = "ar1")
##
##  Coefficients:
##                                   Estimate Std.err    Wald Pr(>|W|)
## (Intercept)                         2.3374  0.0387 3653.32   <2e-16 ***
## blasto_or_dientamoeba_pospositive   0.1308  0.0575    5.17    0.023 *
## on_intervention1                   -0.0356  0.0435    0.67    0.414
## ---
## Signif. codes:  0 '***' 0.001 '**' 0.01 '*' 0.05 '.' 0.1 ' ' 1
##
## Correlation structure = ar1
## Estimated Scale Parameters:
##
##             Estimate Std.err
## (Intercept)    0.113  0.0144
##   Link = identity
##
## Estimated Correlation Parameters:
##       Estimate Std.err
## alpha    0.605  0.0706
## Number of clusters:   78  Maximum cluster size: 3
## [1] "---------------------------------"
## [1] "Simpson"
## [1] "---------------------------------"
##
## Call:
## geepack::geeglm(formula = formula(paste0(my_measure, " ~ log_blastocystis_pcr + log_dientamoeba_pcr + on_intervention")),
##     family = gaussian, data = rich_estim, contrasts = NULL, id = subject_no,
##     corstr = "ar1")
##
```

```
##  Coefficients:
##                      Estimate  Std.err    Wald Pr(>|W|)
## (Intercept)          0.810306 0.008608 8860.64   <2e-16 ***
## log_blastocystis_pcr 0.006959 0.005642    1.52    0.217
## log_dientamoeba_pcr  0.004418 0.002499    3.13    0.077 .
## on_intervention1     0.000163 0.008927    0.00    0.985
## ---
## Signif. codes:  0 '***' 0.001 '**' 0.01 '*' 0.05 '.' 0.1 ' ' 1
##
## Correlation structure = ar1
## Estimated Scale Parameters:
##
##             Estimate  Std.err
## (Intercept)  0.00504 0.000698
##   Link = identity
##
## Estimated Correlation Parameters:
##       Estimate Std.err
## alpha    0.573  0.0796
## Number of clusters:   78  Maximum cluster size: 3
##
## Call:
## geepack::geeglm(formula = formula(paste0(my_measure, " ~ blasto_or_dientamoeba_pos  + on_intervention")),
##     family = gaussian, data = rich_estim, contrasts = NULL, id = subject_no,
##     corstr = "ar1")
##
##  Coefficients:
##                                   Estimate   Std.err    Wald Pr(>|W|)
## (Intercept)                       0.808078  0.008714 8599.89   <2e-16 ***
## blasto_or_dientamoeba_pospositive  0.029443  0.010646    7.65   0.0057 **
## on_intervention1                 -0.000445  0.008891    0.00   0.9601
## ---
## Signif. codes:  0 '***' 0.001 '**' 0.01 '*' 0.05 '.' 0.1 ' ' 1
##
## Correlation structure = ar1
## Estimated Scale Parameters:
##
##             Estimate  Std.err
## (Intercept)  0.00497 0.000689
##   Link = identity
##
## Estimated Correlation Parameters:
##       Estimate Std.err
## alpha    0.574  0.0789
## Number of clusters:   78  Maximum cluster size: 3
## [1] "----------------------------------"
## [1] "InvSimpson"
## [1] "----------------------------------"
##
## Call:
## geepack::geeglm(formula = formula(paste0(my_measure, " ~ log_blastocystis_pcr + log_dientamoeba_pcr + on_intervention")),
##     family = gaussian, data = rich_estim, contrasts = NULL, id = subject_no,
##     corstr = "ar1")
##
##  Coefficients:
##                      Estimate Std.err   Wald Pr(>|W|)
## (Intercept)            6.3283  0.3248 379.55   <2e-16 ***
## log_blastocystis_pcr   0.2279  0.2923   0.61     0.44
## log_dientamoeba_pcr    0.0969  0.0999   0.94     0.33
## on_intervention1      -0.2771  0.3588   0.60     0.44
## ---
## Signif. codes:  0 '***' 0.001 '**' 0.01 '*' 0.05 '.' 0.1 ' ' 1
##
## Correlation structure = ar1
## Estimated Scale Parameters:
##
##             Estimate Std.err
```

```
## (Intercept)     7.15     1.3
##   Link = identity
##
## Estimated Correlation Parameters:
##       Estimate Std.err
## alpha    0.554  0.0617
## Number of clusters:   78  Maximum cluster size: 3
##
## Call:
## geepack::geeglm(formula = formula(paste0(my_measure, " ~ blasto_or_dientamoeba_pos  + on_intervention")),
##     family = gaussian, data = rich_estim, contrasts = NULL, id = subject_no,
##     corstr = "ar1")
##
##  Coefficients:
##                                   Estimate Std.err   Wald Pr(>|W|)
## (Intercept)                          6.205   0.330 354.56   <2e-16 ***
## blasto_or_dientamoeba_pospositive    1.013   0.454   4.97    0.026 *
## on_intervention1                    -0.286   0.354   0.65    0.419
## ---
## Signif. codes:  0 '***' 0.001 '**' 0.01 '*' 0.05 '.' 0.1 ' ' 1
##
## Correlation structure = ar1
## Estimated Scale Parameters:
##
##             Estimate Std.err
## (Intercept)     7.06    1.33
##   Link = identity
##
## Estimated Correlation Parameters:
##       Estimate Std.err
## alpha    0.557  0.0625
## Number of clusters:   78  Maximum cluster size: 3
## [1] "----------------------------------"
## [1] "Fisher"
## [1] "----------------------------------"
##
## Call:
## geepack::geeglm(formula = formula(paste0(my_measure, " ~ log_blastocystis_pcr + log_dientamoeba_pcr + on_intervention")),
##     family = gaussian, data = rich_estim, contrasts = NULL, id = subject_no,
##     corstr = "ar1")
##
##  Coefficients:
##                     Estimate Std.err    Wald Pr(>|W|)
## (Intercept)           8.4146  0.1768 2266.29   <2e-16 ***
## log_blastocystis_pcr   0.3867  0.1348    8.22   0.0041 **
## log_dientamoeba_pcr    0.0448  0.0634    0.50   0.4798
## on_intervention1       0.1373  0.1915    0.51   0.4733
## ---
## Signif. codes:  0 '***' 0.001 '**' 0.01 '*' 0.05 '.' 0.1 ' ' 1
##
## Correlation structure = ar1
## Estimated Scale Parameters:
##
##             Estimate Std.err
## (Intercept)     2.46   0.306
##   Link = identity
##
## Estimated Correlation Parameters:
##       Estimate Std.err
## alpha    0.475  0.0776
## Number of clusters:   78  Maximum cluster size: 3
##
## Call:
## geepack::geeglm(formula = formula(paste0(my_measure, " ~ blasto_or_dientamoeba_pos  + on_intervention")),
##     family = gaussian, data = rich_estim, contrasts = NULL, id = subject_no,
##     corstr = "ar1")
##
```

```
##  Coefficients:
##                                  Estimate Std.err    Wald Pr(>|W|)
## (Intercept)                         8.333   0.173 2327.58   <2e-16 ***
## blasto_or_dientamoeba_pospositive    0.829   0.346    5.73    0.017 *
## on_intervention1                    0.143   0.191    0.56    0.456
## ---
## Signif. codes:  0 '***' 0.001 '**' 0.01 '*' 0.05 '.' 0.1 ' ' 1
##
## Correlation structure = ar1
## Estimated Scale Parameters:
##
##             Estimate Std.err
## (Intercept)     2.43   0.291
##   Link = identity
##
## Estimated Correlation Parameters:
##       Estimate Std.err
## alpha    0.466  0.0799
## Number of clusters:   78  Maximum cluster size: 3
```

Answer to point 1.3.b by the reviewer

What is the distribution of alpha diversity results if three groups were compared: only Blastocystis carriers, only Dientamoeba carriers and non-protozoa carriers at different time points (i.e. baseline, 3 and 6 months)?

Prepare data

```
rich_estim_for_point_13 <- rich_estim %>%
    mutate(
        only_blastocystis_carrier = case_when(
            blastocystis_posneg=="positive" & dientamoeba_posneg == "negative"  ~ 1,
            T ~ 0
        ),
        only_dientamoeba_carrier = case_when(
            blastocystis_posneg=="negative" & dientamoeba_posneg == "positive"  ~ 1,
            T ~ 0
            ),
        more_protists_carrier = case_when(
            blastocystis_posneg=="positive" & dientamoeba_posneg == "positive" ~ 1,
            T ~ 0
            ),
        no_protist_carrier = case_when(
            blastocystis_posneg=="negative" & dientamoeba_posneg == "negative" ~ 1,
            T ~ 0
            ),
        time_point = paste0("Time point ", 3*(time_123-1), " months")
    ) %>%
    mutate(
        protist_status = case_when(
    (only_blastocystis_carrier == 1 & only_dientamoeba_carrier == 0
     & no_protist_carrier == 0 & more_protists_carrier == 0) ~ "Only Blastocystis",
    (only_blastocystis_carrier == 0 & only_dientamoeba_carrier == 1
     & no_protist_carrier == 0 & more_protists_carrier == 0) ~ "Only Dientamoeba",
    (only_blastocystis_carrier == 0 & only_dientamoeba_carrier == 0
     & no_protist_carrier == 0 & more_protists_carrier == 1) ~ "More protists",
    (only_blastocystis_carrier == 0 & only_dientamoeba_carrier == 0 &
       no_protist_carrier == 1 & more_protists_carrier == 0) ~ "No protists",
    T ~ "ERROR"
        ),

      Protists = factor(
        protist_status,
        levels = c("No protists", "Only Blastocystis", "Only Dientamoeba", "More protists")
      )

    )
#no classification index
if (nrow(rich_estim_for_point_13 %>%  filter(protist_status == "ERROR")) > 0) stop("Classification error in the protist positivity
categories")
```

Run the test for individual time points

```
#-------------- time point 1, 2 and 3
for (this_time_point in c(1,2,3)){
  for (measure in alpha_diversity_measures){
    anova_test_one_timepoint <- aov(
      formula = formula(paste0(measure, " ~ protist_status")),
      data = rich_estim_for_point_13 %>% filter(time_123 == this_time_point)
    )
    print("--------------------------------------------")
    print(paste0("measure ", measure, "; time point ", this_time_point))
    print("--------------------------------------------")
    print(summary(anova_test_one_timepoint))
    print(
      TukeyHSD(anova_test_one_timepoint, "protist_status", ordered = T)
    )
  }
}
```

```
## [1] "--------------------------------------------"
## [1] "measure Observed; time point 1"
## [1] "--------------------------------------------"
##                Df Sum Sq Mean Sq F value Pr(>F)
## protist_status  3    811     270     2.2  0.095 .
## Residuals      73   8972     123
## ---
## Signif. codes:  0 '***' 0.001 '**' 0.01 '*' 0.05 '.' 0.1 ' ' 1
##   Tukey multiple comparisons of means
##     95% family-wise confidence level
##     factor levels have been ordered
##
## Fit: aov(formula = formula(paste0(measure, " ~ protist_status")), data = rich_estim_for_point_13 %>% filter(time_123 ==
this_time_point))
##
## $protist_status
##                                    diff    lwr  upr p adj
## Only Dientamoeba-No protists        5.19  -4.81 15.2 0.526
## More protists-No protists           5.59  -8.01 19.2 0.702
## Only Blastocystis-No protists      10.39  -2.13 22.9 0.138
## More protists-Only Dientamoeba      0.40 -15.56 16.4 1.000
## Only Blastocystis-Only Dientamoeba  5.20  -9.85 20.3 0.800
## Only Blastocystis-More protists     4.80 -12.85 22.4 0.891
##
## [1] "--------------------------------------------"
## [1] "measure Chao1; time point 1"
## [1] "--------------------------------------------"
##                Df Sum Sq Mean Sq F value Pr(>F)
## protist_status  3    911     304    1.93   0.13
## Residuals      73  11509     158
##   Tukey multiple comparisons of means
##     95% family-wise confidence level
##     factor levels have been ordered
##
## Fit: aov(formula = formula(paste0(measure, " ~ protist_status")), data = rich_estim_for_point_13 %>% filter(time_123 ==
this_time_point))
##
## $protist_status
##                                    diff    lwr  upr p adj
## Only Dientamoeba-No protists        4.53  -6.80 15.9 0.720
## More protists-No protists           6.89  -8.52 22.3 0.644
## Only Blastocystis-No protists      11.17  -3.02 25.3 0.173
## More protists-Only Dientamoeba      2.36 -15.72 20.4 0.986
## Only Blastocystis-Only Dientamoeba  6.64 -10.41 23.7 0.736
## Only Blastocystis-More protists     4.28 -15.71 24.3 0.943
##
## [1] "--------------------------------------------"
## [1] "measure ACE; time point 1"
## [1] "--------------------------------------------"
##                Df Sum Sq Mean Sq F value Pr(>F)
```

```
## protist_status  3    979     326    2.12   0.11
## Residuals      73  11240     154
##   Tukey multiple comparisons of means
##     95% family-wise confidence level
##     factor levels have been ordered
##
## Fit: aov(formula = formula(paste0(measure, " ~ protist_status")), data = rich_estim_for_point_13 %>% filter(time_123 ==
this_time_point))
##
## $protist_status
##                                     diff    lwr  upr p adj
## Only Dientamoeba-No protists        5.575  -5.63 16.8 0.561
## More protists-No protists           6.449  -8.78 21.7 0.682
## Only Blastocystis-No protists      11.383  -2.63 25.4 0.152
## More protists-Only Dientamoeba      0.875 -17.00 18.7 0.999
## Only Blastocystis-Only Dientamoeba  5.808 -11.04 22.7 0.801
## Only Blastocystis-More protists     4.934 -14.82 24.7 0.913
##
## [1] "--------------------------------------------"
## [1] "measure Shannon; time point 1"
## [1] "--------------------------------------------"
##                Df Sum Sq Mean Sq F value Pr(>F)
## protist_status  3   0.68   0.228    1.81   0.15
## Residuals      73   9.21   0.126
##   Tukey multiple comparisons of means
##     95% family-wise confidence level
##     factor levels have been ordered
##
## Fit: aov(formula = formula(paste0(measure, " ~ protist_status")), data = rich_estim_for_point_13 %>% filter(time_123 ==
this_time_point))
##
## $protist_status
##                                    diff     lwr   upr p adj
## Only Dientamoeba-No protists       0.069 -0.2516 0.390 0.942
## More protists-No protists          0.204 -0.2318 0.640 0.609
## Only Blastocystis-No protists      0.316 -0.0856 0.717 0.173
## More protists-Only Dientamoeba     0.135 -0.3764 0.647 0.899
## Only Blastocystis-Only Dientamoeba 0.247 -0.2358 0.729 0.538
## Only Blastocystis-More protists    0.111 -0.4542 0.677 0.955
##
## [1] "--------------------------------------------"
## [1] "measure Simpson; time point 1"
## [1] "--------------------------------------------"
##                Df Sum Sq Mean Sq F value Pr(>F)
## protist_status  3  0.027 0.00885    1.38   0.26
## Residuals      73  0.468 0.00641
##   Tukey multiple comparisons of means
##     95% family-wise confidence level
##     factor levels have been ordered
##
## Fit: aov(formula = formula(paste0(measure, " ~ protist_status")), data = rich_estim_for_point_13 %>% filter(time_123 ==
this_time_point))
##
## $protist_status
##                                     diff     lwr    upr p adj
## Only Dientamoeba-No protists       0.0270 -0.0452 0.0993 0.759
## More protists-No protists          0.0415 -0.0567 0.1398 0.684
## Only Blastocystis-No protists      0.0567 -0.0337 0.1471 0.358
## More protists-Only Dientamoeba     0.0145 -0.1008 0.1298 0.987
## Only Blastocystis-Only Dientamoeba 0.0297 -0.0790 0.1384 0.890
## Only Blastocystis-More protists    0.0152 -0.1123 0.1426 0.989
##
## [1] "--------------------------------------------"
## [1] "measure InvSimpson; time point 1"
## [1] "--------------------------------------------"
##                Df Sum Sq Mean Sq F value Pr(>F)
## protist_status  3     19    6.40    0.72   0.54
```

```
## Residuals      73    652    8.93
##   Tukey multiple comparisons of means
##     95% family-wise confidence level
##     factor levels have been ordered
##
## Fit: aov(formula = formula(paste0(measure, " ~ protist_status")), data = rich_estim_for_point_13 %>% filter(time_123 ==
this_time_point))
##
## $protist_status
##                                    diff   lwr  upr p adj
## Only Dientamoeba-No protists       0.309 -2.39 3.01 0.990
## More protists-No protists          0.959 -2.71 4.63 0.902
## Only Blastocystis-No protists      1.732 -1.64 5.11 0.535
## More protists-Only Dientamoeba     0.650 -3.65 4.95 0.979
## Only Blastocystis-Only Dientamoeba 1.423 -2.63 5.48 0.793
## Only Blastocystis-More protists    0.773 -3.98 5.53 0.974
##
## [1] "--------------------------------------------"
## [1] "measure Fisher; time point 1"
## [1] "--------------------------------------------"
##                Df Sum Sq Mean Sq F value Pr(>F)
## protist_status  3   22.8    7.60    2.28  0.086 .
## Residuals      73  243.0    3.33
## ---
## Signif. codes:  0 '***' 0.001 '**' 0.01 '*' 0.05 '.' 0.1 ' ' 1
##   Tukey multiple comparisons of means
##     95% family-wise confidence level
##     factor levels have been ordered
##
## Fit: aov(formula = formula(paste0(measure, " ~ protist_status")), data = rich_estim_for_point_13 %>% filter(time_123 ==
this_time_point))
##
## $protist_status
##                                     diff    lwr  upr p adj
## Only Dientamoeba-No protists       0.8584 -0.788 2.51 0.522
## More protists-No protists          0.9098 -1.329 3.15 0.710
## Only Blastocystis-No protists      1.7602 -0.300 3.82 0.121
## More protists-Only Dientamoeba     0.0514 -2.576 2.68 1.000
## Only Blastocystis-Only Dientamoeba 0.9018 -1.575 3.38 0.774
## Only Blastocystis-More protists    0.8504 -2.054 3.76 0.868
##
## [1] "--------------------------------------------"
## [1] "measure Observed; time point 2"
## [1] "--------------------------------------------"
##                Df Sum Sq Mean Sq F value Pr(>F)
## protist_status  3   1034     345    4.29 0.0077 **
## Residuals      72   5793      80
## ---
## Signif. codes:  0 '***' 0.001 '**' 0.01 '*' 0.05 '.' 0.1 ' ' 1
##   Tukey multiple comparisons of means
##     95% family-wise confidence level
##     factor levels have been ordered
##
## Fit: aov(formula = formula(paste0(measure, " ~ protist_status")), data = rich_estim_for_point_13 %>% filter(time_123 ==
this_time_point))
##
## $protist_status
##                                    diff     lwr  upr p adj
## No protists-More protists           3.64 -10.330 17.6 0.903
## Only Dientamoeba-More protists      9.55  -5.821 24.9 0.366
## Only Blastocystis-More protists    17.50  -0.518 35.5 0.060
## Only Dientamoeba-No protists        5.91  -1.851 13.7 0.197
## Only Blastocystis-No protists      13.86   1.666 26.1 0.020
## Only Blastocystis-Only Dientamoeba  7.95  -5.820 21.7 0.432
##
## [1] "--------------------------------------------"
## [1] "measure Chao1; time point 2"
```

```
## [1] "--------------------------------------------"
##                Df Sum Sq Mean Sq F value Pr(>F)
## protist_status  3   1251     417    4.22 0.0083 **
## Residuals      72   7119      99
## ---
## Signif. codes:  0 '***' 0.001 '**' 0.01 '*' 0.05 '.' 0.1 ' ' 1
##   Tukey multiple comparisons of means
##     95% family-wise confidence level
##     factor levels have been ordered
##
## Fit: aov(formula = formula(paste0(measure, " ~ protist_status")), data = rich_estim_for_point_13 %>% filter(time_123 ==
this_time_point))
##
## $protist_status
##                                    diff    lwr  upr p adj
## No protists-More protists           2.55 -12.93 18.0 0.972
## Only Dientamoeba-More protists      9.43  -7.61 26.5 0.470
## Only Blastocystis-More protists    17.81  -2.17 37.8 0.098
## Only Dientamoeba-No protists        6.87  -1.73 15.5 0.162
## Only Blastocystis-No protists      15.25   1.73 28.8 0.021
## Only Blastocystis-Only Dientamoeba  8.38  -6.89 23.7 0.477
##
## [1] "--------------------------------------------"
## [1] "measure ACE; time point 2"
## [1] "--------------------------------------------"
##                Df Sum Sq Mean Sq F value Pr(>F)
## protist_status  3   1217     406    4.22 0.0084 **
## Residuals      72   6926      96
## ---
## Signif. codes:  0 '***' 0.001 '**' 0.01 '*' 0.05 '.' 0.1 ' ' 1
##   Tukey multiple comparisons of means
##     95% family-wise confidence level
##     factor levels have been ordered
##
## Fit: aov(formula = formula(paste0(measure, " ~ protist_status")), data = rich_estim_for_point_13 %>% filter(time_123 ==
this_time_point))
##
## $protist_status
##                                    diff    lwr  upr p adj
## No protists-More protists           3.64 -11.64 18.9 0.923
## Only Dientamoeba-More protists     10.21  -6.59 27.0 0.386
## Only Blastocystis-More protists    18.60  -1.10 38.3 0.071
## Only Dientamoeba-No protists        6.57  -1.91 15.1 0.184
## Only Blastocystis-No protists      14.96   1.63 28.3 0.022
## Only Blastocystis-Only Dientamoeba  8.39  -6.67 23.5 0.463
##
## [1] "--------------------------------------------"
## [1] "measure Shannon; time point 2"
## [1] "--------------------------------------------"
##                Df Sum Sq Mean Sq F value Pr(>F)
## protist_status  3   0.52   0.174    1.59    0.2
## Residuals      72   7.87   0.109
##   Tukey multiple comparisons of means
##     95% family-wise confidence level
##     factor levels have been ordered
##
## Fit: aov(formula = formula(paste0(measure, " ~ protist_status")), data = rich_estim_for_point_13 %>% filter(time_123 ==
this_time_point))
##
## $protist_status
##                                      diff    lwr   upr p adj
## More protists-No protists          0.0711 -0.444 0.586 0.983
## Only Dientamoeba-No protists       0.1593 -0.127 0.445 0.464
## Only Blastocystis-No protists      0.3002 -0.149 0.750 0.303
## Only Dientamoeba-More protists     0.0882 -0.478 0.655 0.977
## Only Blastocystis-More protists    0.2291 -0.435 0.893 0.801
## Only Blastocystis-Only Dientamoeba 0.1409 -0.367 0.649 0.885
```

```
##
## [1] "--------------------------------------------"
## [1] "measure Simpson; time point 2"
## [1] "--------------------------------------------"
##                Df Sum Sq Mean Sq F value Pr(>F)
## protist_status  3 0.0067 0.00224    0.52   0.67
## Residuals      72 0.3134 0.00435
##   Tukey multiple comparisons of means
##     95% family-wise confidence level
##     factor levels have been ordered
##
## Fit: aov(formula = formula(paste0(measure, " ~ protist_status")), data = rich_estim_for_point_13 %>% filter(time_123 ==
this_time_point))
##
## $protist_status
##                                    diff     lwr    upr p adj
## Only Blastocystis-No protists      0.0107 -0.0790 0.1004 0.989
## More protists-No protists          0.0195 -0.0833 0.1222 0.959
## Only Dientamoeba-No protists       0.0253 -0.0318 0.0823 0.651
## More protists-Only Blastocystis    0.0088 -0.1237 0.1413 0.998
## Only Dientamoeba-Only Blastocystis 0.0146 -0.0867 0.1159 0.981
## Only Dientamoeba-More protists     0.0058 -0.1072 0.1188 0.999
##
## [1] "--------------------------------------------"
## [1] "measure InvSimpson; time point 2"
## [1] "--------------------------------------------"
##                Df Sum Sq Mean Sq F value Pr(>F)
## protist_status  3     25    8.20    1.26   0.29
## Residuals      72    467    6.49
##   Tukey multiple comparisons of means
##     95% family-wise confidence level
##     factor levels have been ordered
##
## Fit: aov(formula = formula(paste0(measure, " ~ protist_status")), data = rich_estim_for_point_13 %>% filter(time_123 ==
this_time_point))
##
## $protist_status
##                                    diff    lwr  upr p adj
## More protists-No protists          0.0738 -3.893 4.04 1.000
## Only Dientamoeba-No protists       1.3667 -0.837 3.57 0.368
## Only Blastocystis-No protists      1.5983 -1.865 5.06 0.620
## Only Dientamoeba-More protists     1.2928 -3.071 5.66 0.864
## Only Blastocystis-More protists    1.5244 -3.592 6.64 0.862
## Only Blastocystis-Only Dientamoeba 0.2316 -3.680 4.14 0.999
##
## [1] "--------------------------------------------"
## [1] "measure Fisher; time point 2"
## [1] "--------------------------------------------"
##                Df Sum Sq Mean Sq F value Pr(>F)
## protist_status  3   28.7    9.56    4.33 0.0073 **
## Residuals      72  158.8    2.21
## ---
## Signif. codes:  0 '***' 0.001 '**' 0.01 '*' 0.05 '.' 0.1 ' ' 1
##   Tukey multiple comparisons of means
##     95% family-wise confidence level
##     factor levels have been ordered
##
## Fit: aov(formula = formula(paste0(measure, " ~ protist_status")), data = rich_estim_for_point_13 %>% filter(time_123 ==
this_time_point))
##
## $protist_status
##                                   diff     lwr  upr p adj
## No protists-More protists          0.604 -1.7088 2.92 0.902
## Only Dientamoeba-More protists     1.588 -0.9566 4.13 0.362
## Only Blastocystis-More protists    2.912 -0.0711 5.90 0.058
## Only Dientamoeba-No protists       0.984 -0.3009 2.27 0.192
## Only Blastocystis-No protists      2.308  0.2890 4.33 0.019
```

```
## Only Blastocystis-Only Dientamoeba 1.325 -0.9560 3.61 0.427
##
## [1] "--------------------------------------------"
## [1] "measure Observed; time point 3"
## [1] "--------------------------------------------"
##                Df Sum Sq Mean Sq F value Pr(>F)
## protist_status  3    650   216.7    3.28  0.026 *
## Residuals      70   4630    66.1
## ---
## Signif. codes:  0 '***' 0.001 '**' 0.01 '*' 0.05 '.' 0.1 ' ' 1
##   Tukey multiple comparisons of means
##     95% family-wise confidence level
##     factor levels have been ordered
##
## Fit: aov(formula = formula(paste0(measure, " ~ protist_status")), data = rich_estim_for_point_13 %>% filter(time_123 ==
this_time_point))
##
## $protist_status
##                                      diff     lwr  upr p adj
## Only Dientamoeba-No protists        2.98  -4.700 10.7 0.738
## More protists-No protists           7.09  -5.591 19.8 0.460
## Only Blastocystis-No protists      10.55   0.572 20.5 0.034
## More protists-Only Dientamoeba      4.11 -10.158 18.4 0.873
## Only Blastocystis-Only Dientamoeba  7.58  -4.360 19.5 0.347
## Only Blastocystis-More protists     3.47 -12.164 19.1 0.937
##
## [1] "--------------------------------------------"
## [1] "measure Chao1; time point 3"
## [1] "--------------------------------------------"
##                Df Sum Sq Mean Sq F value Pr(>F)
## protist_status  3    638     213    2.02   0.12
## Residuals      70   7387     106
##   Tukey multiple comparisons of means
##     95% family-wise confidence level
##     factor levels have been ordered
##
## Fit: aov(formula = formula(paste0(measure, " ~ protist_status")), data = rich_estim_for_point_13 %>% filter(time_123 ==
this_time_point))
##
## $protist_status
##                                      diff    lwr  upr p adj
## Only Dientamoeba-No protists        3.08  -6.62 12.8 0.838
## More protists-No protists           7.26  -8.75 23.3 0.633
## Only Blastocystis-No protists      10.32  -2.29 22.9 0.146
## More protists-Only Dientamoeba      4.19 -13.84 22.2 0.928
## Only Blastocystis-Only Dientamoeba  7.25  -7.83 22.3 0.588
## Only Blastocystis-More protists     3.06 -16.68 22.8 0.977
##
## [1] "--------------------------------------------"
## [1] "measure ACE; time point 3"
## [1] "--------------------------------------------"
##                Df Sum Sq Mean Sq F value Pr(>F)
## protist_status  3    745   248.4    2.82  0.045 *
## Residuals      70   6167    88.1
## ---
## Signif. codes:  0 '***' 0.001 '**' 0.01 '*' 0.05 '.' 0.1 ' ' 1
##   Tukey multiple comparisons of means
##     95% family-wise confidence level
##     factor levels have been ordered
##
## Fit: aov(formula = formula(paste0(measure, " ~ protist_status")), data = rich_estim_for_point_13 %>% filter(time_123 ==
this_time_point))
##
## $protist_status
##                                      diff     lwr  upr p adj
## Only Dientamoeba-No protists        3.76  -5.103 12.6 0.681
## More protists-No protists           8.00  -6.630 22.6 0.479
```

```
## Only Blastocystis-No protists      10.93  -0.596 22.4 0.069
## More protists-Only Dientamoeba      4.25 -12.224 20.7 0.905
## Only Blastocystis-Only Dientamoeba  7.17  -6.611 20.9 0.523
## Only Blastocystis-More protists     2.92 -15.118 21.0 0.974
##
## [1] "--------------------------------------------"
## [1] "measure Shannon; time point 3"
## [1] "--------------------------------------------"
##               Df Sum Sq Mean Sq F value Pr(>F)
## protist_status  3   0.70   0.235    2.12   0.11
## Residuals      70   7.77   0.111
##   Tukey multiple comparisons of means
##     95% family-wise confidence level
##     factor levels have been ordered
##
## Fit: aov(formula = formula(paste0(measure, " ~ protist_status")), data = rich_estim_for_point_13 %>% filter(time_123 ==
this_time_point))
##
## $protist_status
##                                      diff     lwr   upr p adj
## Only Dientamoeba-No protists       0.1601 -0.1545 0.475 0.541
## More protists-No protists          0.2448 -0.2746 0.764 0.604
## Only Blastocystis-No protists      0.3104 -0.0986 0.719 0.199
## More protists-Only Dientamoeba     0.0848 -0.4998 0.669 0.981
## Only Blastocystis-Only Dientamoeba 0.1504 -0.3387 0.639 0.850
## Only Blastocystis-More protists    0.0656 -0.5748 0.706 0.993
##
## [1] "--------------------------------------------"
## [1] "measure Simpson; time point 3"
## [1] "--------------------------------------------"
##               Df Sum Sq Mean Sq F value Pr(>F)
## protist_status  3  0.023 0.00783    1.64   0.19
## Residuals      70  0.335 0.00478
##   Tukey multiple comparisons of means
##     95% family-wise confidence level
##     factor levels have been ordered
##
## Fit: aov(formula = formula(paste0(measure, " ~ protist_status")), data = rich_estim_for_point_13 %>% filter(time_123 ==
this_time_point))
##
## $protist_status
##                                        diff     lwr   upr p adj
## Only Dientamoeba-No protists       0.039763 -0.0255 0.105 0.383
## Only Blastocystis-No protists      0.044818 -0.0400 0.130 0.510
## More protists-No protists          0.045382 -0.0624 0.153 0.686
## Only Blastocystis-Only Dientamoeba 0.005055 -0.0964 0.107 0.999
## More protists-Only Dientamoeba     0.005619 -0.1157 0.127 0.999
## More protists-Only Blastocystis    0.000564 -0.1323 0.133 1.000
##
## [1] "--------------------------------------------"
## [1] "measure InvSimpson; time point 3"
## [1] "--------------------------------------------"
##               Df Sum Sq Mean Sq F value Pr(>F)
## protist_status  3     17    5.59    0.84   0.48
## Residuals      70    465    6.65
##   Tukey multiple comparisons of means
##     95% family-wise confidence level
##     factor levels have been ordered
##
## Fit: aov(formula = formula(paste0(measure, " ~ protist_status")), data = rich_estim_for_point_13 %>% filter(time_123 ==
this_time_point))
##
## $protist_status
##                                     diff   lwr  upr p adj
## More protists-No protists          0.734 -3.28 4.75 0.963
## Only Dientamoeba-No protists       1.069 -1.37 3.50 0.657
## Only Blastocystis-No protists      1.389 -1.78 4.55 0.657
```

```
## Only Dientamoeba-More protists     0.334 -4.19 4.86 0.997
## Only Blastocystis-More protists    0.655 -4.30 5.61 0.985
## Only Blastocystis-Only Dientamoeba 0.320 -3.46 4.10 0.996
##
## [1] "--------------------------------------------"
## [1] "measure Fisher; time point 3"
## [1] "--------------------------------------------"
##                Df Sum Sq Mean Sq F value Pr(>F)
## protist_status  3   17.7    5.91    3.26  0.027 *
## Residuals      70  127.0    1.81
## ---
## Signif. codes:  0 '***' 0.001 '**' 0.01 '*' 0.05 '.' 0.1 ' ' 1
##   Tukey multiple comparisons of means
##     95% family-wise confidence level
##     factor levels have been ordered
##
## Fit: aov(formula = formula(paste0(measure, " ~ protist_status")), data = rich_estim_for_point_13 %>% filter(time_123 ==
this_time_point))
##
## $protist_status
##                                     diff     lwr  upr p adj
## Only Dientamoeba-No protists       0.482 -0.7895 1.75 0.751
## More protists-No protists          1.162 -0.9375 3.26 0.469
## Only Blastocystis-No protists      1.749  0.0957 3.40 0.034
## More protists-Only Dientamoeba     0.680 -1.6831 3.04 0.873
## Only Blastocystis-Only Dientamoeba 1.267 -0.7103 3.24 0.338
## Only Blastocystis-More protists    0.587 -2.0022 3.18 0.933
```

```
mdf = reshape2::melt(rich_estim_for_point_13, measure.vars = alpha_diversity_measures)

if (!is.null(alpha_diversity_measures)) {
    if (any(alpha_diversity_measures %in% as.character(mdf$variable))) {
        mdf <- mdf[as.character(mdf$variable) %in% alpha_diversity_measures,
            ]
    }
    else {
        warning("Argument to `measures` not supported. All alpha-diversity measures (should be) included in plot.")
    }
}


richness_map = aes_string(x = "Protists" , y = "value")
p1 = ggplot(mdf %>% filter(variable %in% alpha_diversity_measures), richness_map) + geom_point(na.rm = TRUE) +
    theme(axis.text.x = element_text(angle = -90, vjust = 0.5, hjust = 0)) +
    ylab("Alpha Diversity Measure") +
    facet_grid(variable~time_point, scales = "free") +
    geom_boxplot()

print(p1)
```

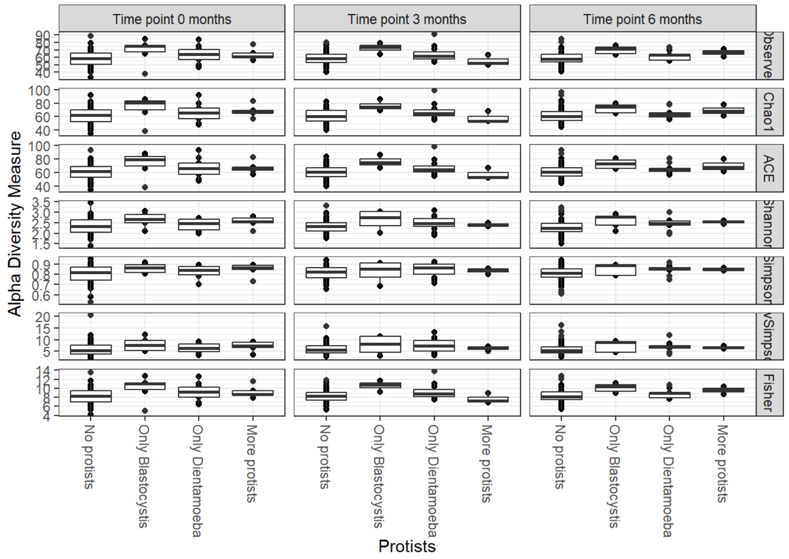

Formal testing of several exposures using GEE

predictor = intervention + allocation

Alpha diversity measures, presence of parasites, quantity of parasites (as suggested by Reviewer 1 for formal testing) in various time points of the study, and their changes with intervention.

```
dependent_names = c(
    alpha_diversity_measures,
    "Blastocystis, positivity/negativity" = "bl_10",
    "Dientamoeba, positivity/negativity"="di_10",
    "Blastocystis, decadic logarithm" = "log_blastocystis_pcr",
    "Dientamoeba, decadic logarithm"="log_dientamoeba_pcr"
    )
res_tbl <- data.frame()
vr <- 0 #line in the result table

for (dependent_name in dependent_names){
    vr <- vr + 1

    res_tbl[vr, "dependent_var"] <- dependent_name
    rich_estim[, "dependent"] <- rich_estim[ , dependent_name]

    #GEE
    gee_res<- geepack::geeglm(
        formula = formula(paste( 'dependent ~  int_group + on_intervention  ',
                                 sep = "")),
        id = subject_no,
        data = rich_estim,
        family = gaussian,
        waves = time_123,
        corstr = "ar1",
        contrasts = NULL
        )
    summary(gee_res)

    res_tbl[vr, "p_val_GEE_treatment_group"] <- summary(gee_res)$coefficients["int_group","Pr(>|W|)"]
    res_tbl[vr, "p_val_GEE_on_intervention"] <- summary(gee_res)$coefficients["on_intervention1","Pr(>|W|)"]
    res_tbl[vr, "coeff_GEE_treatment_group"] <- summary(gee_res)$coefficients["int_group","Estimate"]
    res_tbl[vr, "coeff_GEE_being_on_intervention"] <- summary(gee_res)$coefficients["on_intervention1","Estimate"]


    #GEE- yet another model, by intervention
    gee_res3<- geepack::geeglm(
        formula = formula(paste( 'dependent ~ time_123 +int_group+ time_123:int_group  ', sep = "")),
        id = subject_no,
        data = rich_estim ,
        family = gaussian,
        corstr = "ar1",
        contrasts = NULL
        )
    summary(gee_res3)

    res_tbl[vr, "p_val_gee3_time_123"] <-
        summary(gee_res3)$coefficients["time_123", "Pr(>|W|)"]
    res_tbl[vr, "p_val_gee3_int_group"] <-
        summary(gee_res3)$coefficients["int_group", "Pr(>|W|)"]
    res_tbl[vr, "p_val_gee2_time_123:int_group"] <-
        summary(gee_res3)$coefficients["time_123:int_group", "Pr(>|W|)"]
    res_tbl[vr, "coef_gee3_time_123"] <-
        summary(gee_res3)$coefficients["time_123", "Estimate"]
    res_tbl[vr, "coef_gee3_int_group"] <-
        summary(gee_res3)$coefficients["int_group", "Estimate"]
    res_tbl[vr, "coef_gee2_time_123:int_group"] <-
        summary(gee_res3)$coefficients["time_123:int_group", "Estimate"]


    # ----------------------------------------
    # draw the abundances in a spaghetti graph
```

```
# ----------------------------------------

graph_data <- rich_estim %>%
    mutate(currently_taking   = ifelse(on_intervention == 1, "Lactobacillus", "Placebo"),
           currently_taking_f = factor(currently_taking, levels = c("Lactobacillus", "Placebo")),
           treatment_group = ifelse(study_supplement == "L", "Lactobacillus", "Placebo")
        ) %>%
    group_by(subject_no) %>%
    mutate(any_positive = any(dependent>0)) %>%
    filter(any_positive) %>%
    ungroup()

y_name = dependent_name
if (y_name == "log_blastocystis_pcr") y_name <- "Blastocystis, decadic logarithm"
if (y_name == "log_dientamoeba_pcr")  y_name <- "Dientamoeba, decadic logarithm"


p1 <- ggplot(data = graph_data,
             mapping = aes_string(x = "time_123", y = "dependent")) +
    scale_color_manual(values=c( "red", "blue", "red", "blue")) +
    #geom_line(aes(group = id_enrollment, color = factor(int_group))) +
    geom_line(aes(group = int_subject), color = "grey", size = 1)+
    geom_point(aes(color = factor(currently_taking)), size = 3, ) +
    #geom_text(aes(label = int_subject),
    #          size = 3,
    #          #position_dodge(width = 1),
    #          vjust = -0.5)+
    ggtitle(label = y_name) +
    theme(plot.title = element_text(size = 20))+
    facet_grid(treatment_group ~ .) +

    #format of titles
    theme(axis.title = element_text( size = 16))+
    theme(strip.text.y  = element_text(size = 16, angle = 90))+

    #vertical axis
    #scale_y_continuous(labels = scales::percent_format(accuracy = NULL)) +
    ylab(y_name) +
    theme(axis.text.y = element_text( size = 13))+


    #horizontal axis
    scale_x_continuous(breaks = c(1,2,3), labels = c("baseline","3 mo","6 mo")) +
    xlab("Visits") +
    theme(axis.text.x = element_text( size = 13))+

    #legend labels
    labs( color = "Sample taken\nwhile on")+
    theme(legend.text = element_text(size = 13),
          legend.title = element_text(size = 16))

y_range <- ggplot_build(p1)$layout$panel_params[[2]]$y.range
#annotation of the p value: this will write the P value only on one facet
p1 <- p1 + geom_text(
             label = paste("Effect of intervention\nP =",
                sprintf("%.3f", res_tbl[vr, "p_val_GEE_on_intervention"])
                ), size = 5,
             data = data.frame(time_123 = 2.5,
                               dependent = y_range[1] + (y_range[2]-y_range[1])*0.90,
                               treatment_group = "Lactobacillus")
            )


#--- print the graph and also the table of p values
print(p1)
print(res_tbl[vr,] %>%
          select(1:3, starts_with("p_val")) %>%
```

```
            t())
    #%>%
    #          kable(format = "html", digits = 4) %>%
    #          kable_styling()

    ggsave(filename = paste0("rel_abund_figure_", dependent_name, ".pdf"),
       plot = p1,
       width = 8.27, height = 11.69, #A4 portrait
       #width = 11.69, height = 8.27, #A4 landscape
       device = "pdf",
       path = "./graphs_and_outputs/relative_abundance_figures"
       )
    ggsave(filename = paste0("rel_abund_figure_", dependent_name, ".svg"),
       plot = p1,
       width = 8.27, height = 11.69, #A4 portrait
       #width = 11.69, height = 8.27, #A4 landscape
       device = "svg",
       path = "./graphs_and_outputs/relative_abundance_figures"
    )

}
```

```
## Warning: Using `size` aesthetic for lines was deprecated in ggplot2 3.4.0.
## i Please use `linewidth` instead.
## This warning is displayed once every 8 hours.
## Call `lifecycle::last_lifecycle_warnings()` to see where this warning was
## generated.
```

```
##                               1
## dependent_var                 "Observed"
## p_val_GEE_treatment_group     "0.311"
## p_val_GEE_on_intervention     "0.982"
## p_val_gee3_time_123           "0.698"
## p_val_gee3_int_group          "0.395"
## p_val_gee2_time_123:int_group "0.777"
```

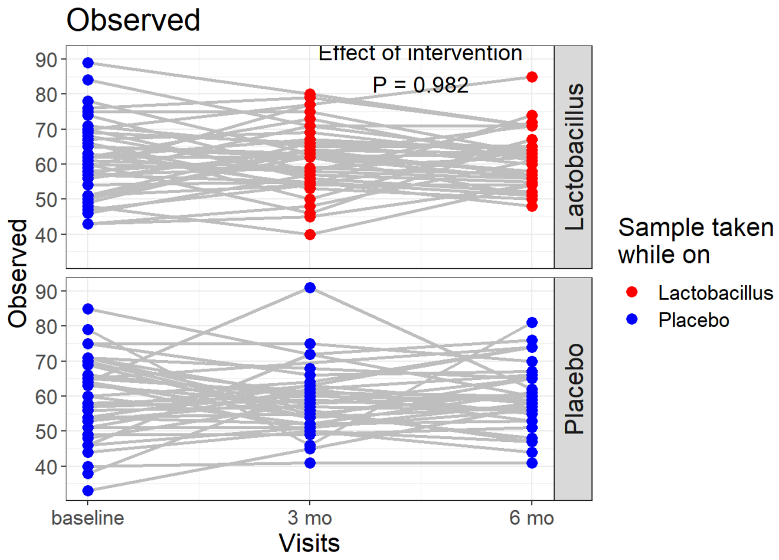


```
##                               2
## dependent_var                 "Chao1"
## p_val_GEE_treatment_group     "0.219"
## p_val_GEE_on_intervention     "0.673"
## p_val_gee3_time_123           "0.807"
## p_val_gee3_int_group          "0.402"
## p_val_gee2_time_123:int_group "0.781"
```

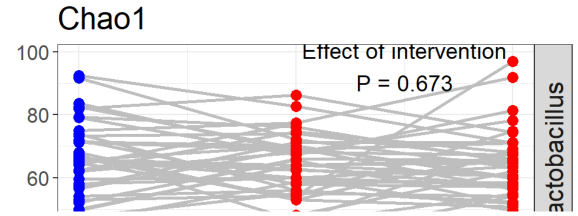

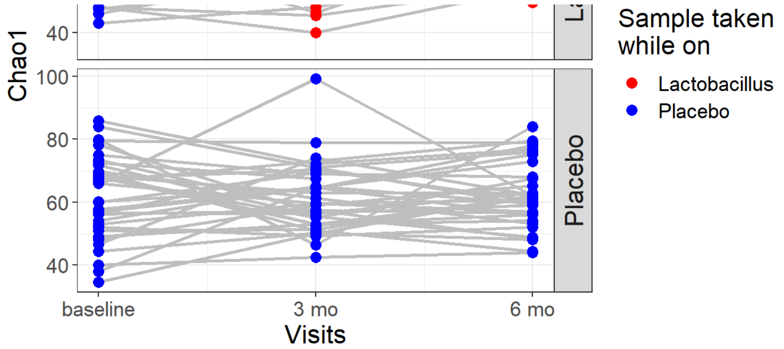


```
##                                3
## dependent_var                  "ACE"
## p_val_GEE_treatment_group      "0.306"
## p_val_GEE_on_intervention      "0.702"
## p_val_gee3_time_123            "0.692"
## p_val_gee3_int_group           "0.408"
## p_val_gee2_time_123:int_group "0.701"
```

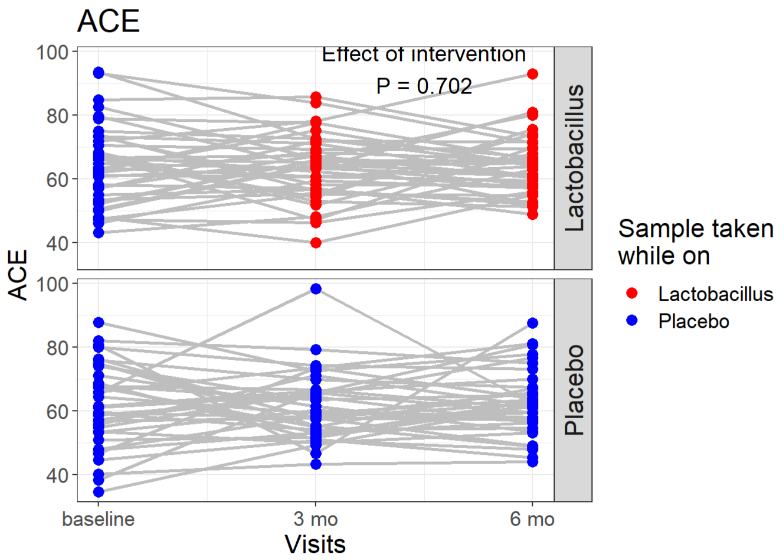


```
##                                4
## dependent_var                  "Shannon"
## p_val_GEE_treatment_group      "0.19"
## p_val_GEE_on_intervention      "0.18"
## p_val_gee3_time_123            "0.924"
## p_val_gee3_int_group           "0.173"
## p_val_gee2_time_123:int_group "0.295"
```

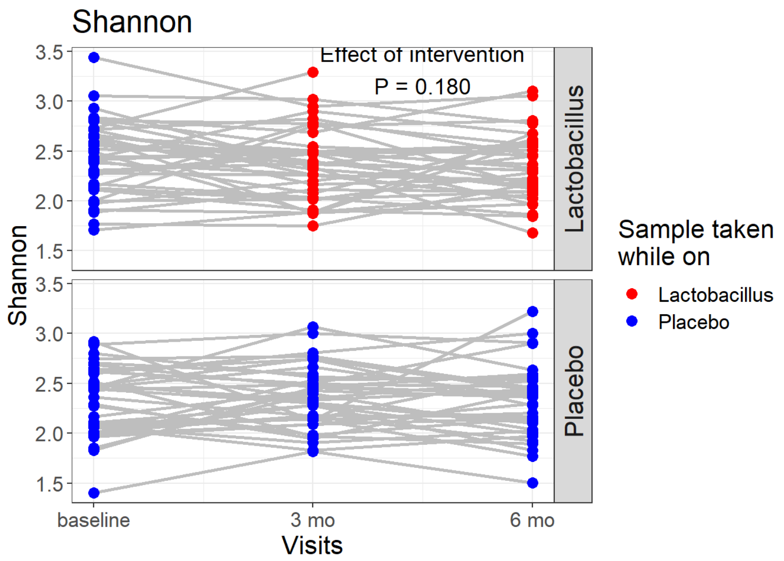


```
##                                5
## dependent_var                  "Simpson"
```

```
## p_val_GEE_treatment_group     "0.74"
## p_val_GEE_on_intervention     "0.678"
## p_val_gee3_time_123           "0.46"
## p_val_gee3_int_group          "0.55"
## p_val_gee2_time_123:int_group "0.532"
```

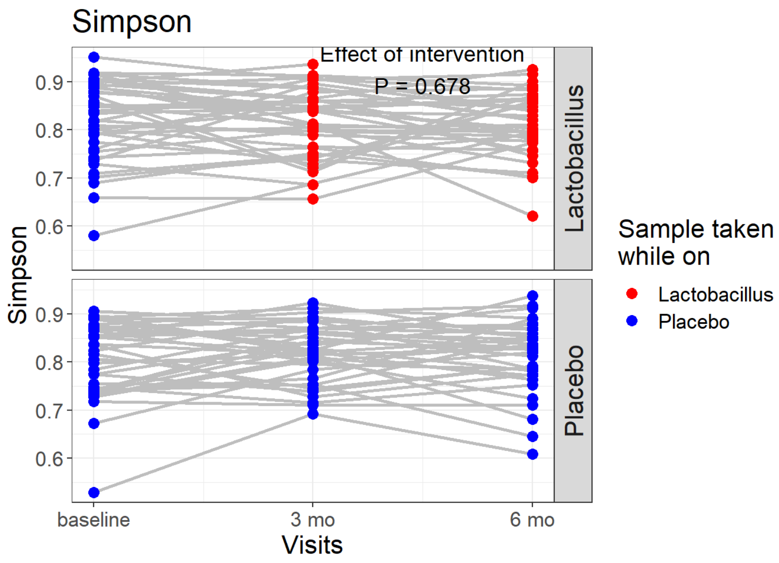


```
##                               6
## dependent_var                 "InvSimpson"
## p_val_GEE_treatment_group     "0.356"
## p_val_GEE_on_intervention     "0.268"
## p_val_gee3_time_123           "0.387"
## p_val_gee3_int_group          "0.144"
## p_val_gee2_time_123:int_group "0.131"
```

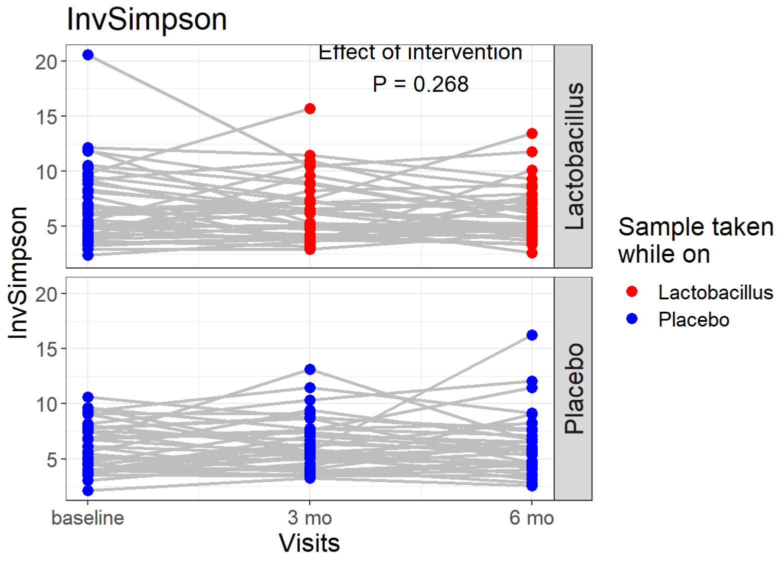


```
##                               7
## dependent_var                 "Fisher"
## p_val_GEE_treatment_group     "0.313"
## p_val_GEE_on_intervention     "0.99"
## p_val_gee3_time_123           "0.731"
## p_val_gee3_int_group          "0.4"
## p_val_gee2_time_123:int_group "0.779"
```

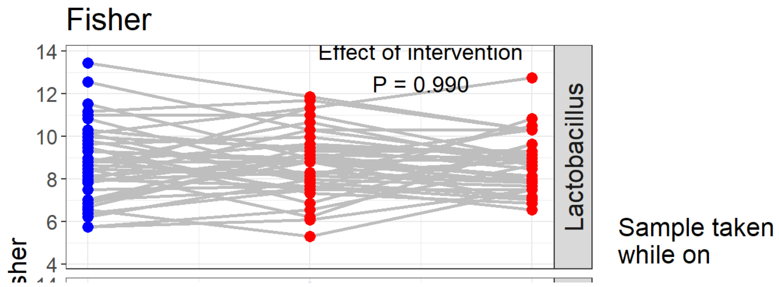

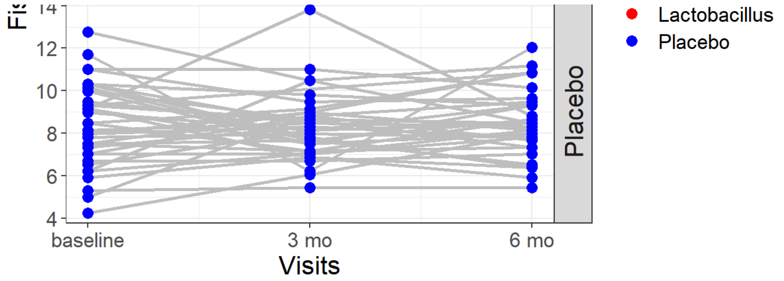


```
##                               8
## dependent_var                 "bl_10"
## p_val_GEE_treatment_group     "0.445"
## p_val_GEE_on_intervention     "0.349"
## p_val_gee3_time_123           "0.149"
## p_val_gee3_int_group          "0.958"
## p_val_gee2_time_123:int_group "0.328"
```

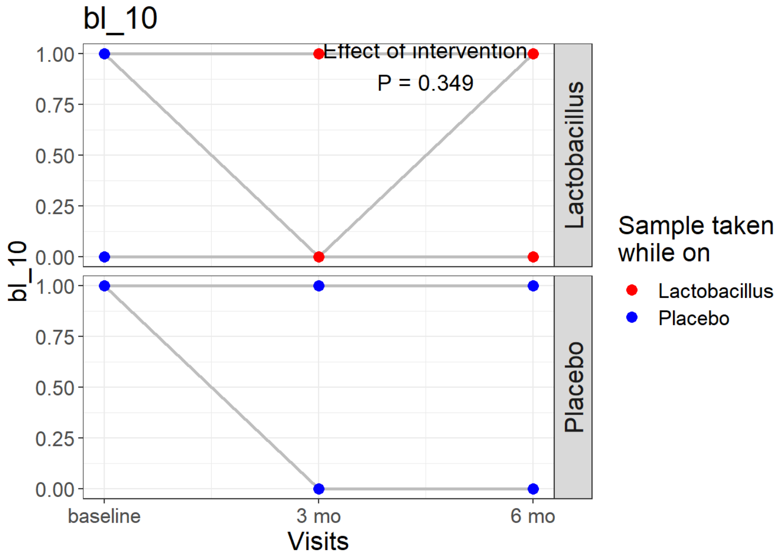


```
##                               9
## dependent_var                 "di_10"
## p_val_GEE_treatment_group     "0.605"
## p_val_GEE_on_intervention     "0.133"
## p_val_gee3_time_123           "0.61"
## p_val_gee3_int_group          "0.288"
## p_val_gee2_time_123:int_group "0.118"
```

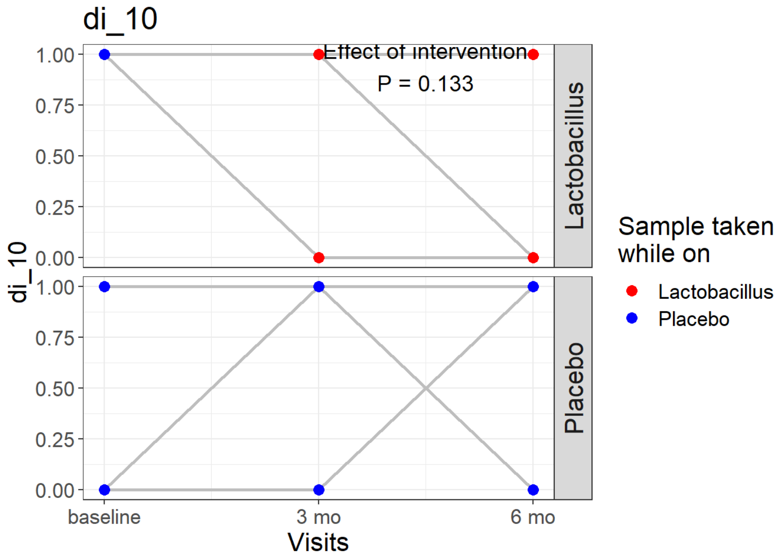


```
##                               10
## dependent_var                 "log_blastocystis_pcr"
## p_val_GEE_treatment_group     "0.106"
## p_val_GEE_on_intervention     "0.136"
## p_val_gee3_time_123           "0.206"
```

```
## p_val_gee3_int_group          "0.0765"
## p_val_gee2_time_123:int_group "0.292"
```

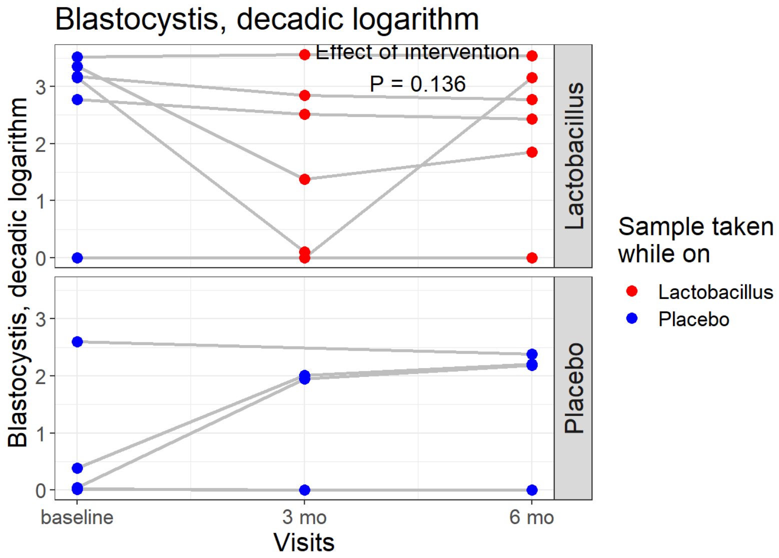


```
##                               11
## dependent_var                 "log_dientamoeba_pcr"
## p_val_GEE_treatment_group     "0.295"
## p_val_GEE_on_intervention     "0.101"
## p_val_gee3_time_123           "0.646"
## p_val_gee3_int_group          "0.164"
## p_val_gee2_time_123:int_group "0.172"
```

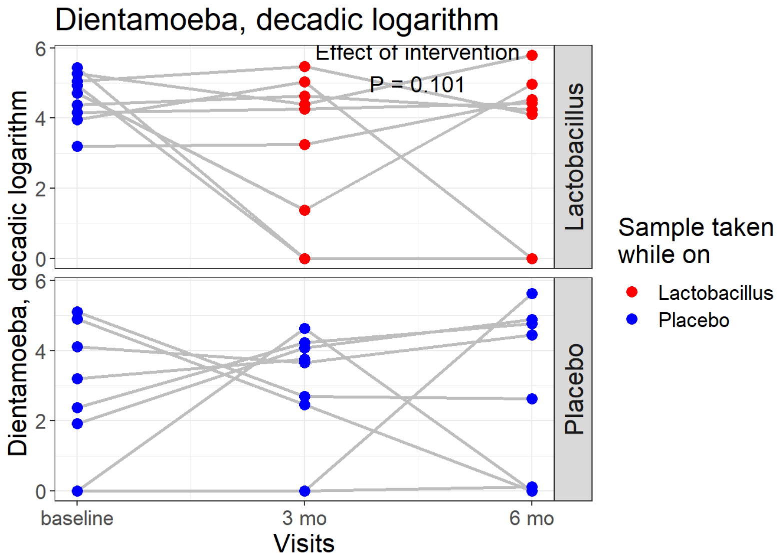


```
my_ts <- strftime(Sys.time(), "%Y%m%dT%H%M%S")
xlsx::write.xlsx(res_tbl,
                 file = file.path("./graphs_and_outputs",
                                  paste0("results_glm_and_gee_alphadiv_and_parasites_with_intervention", my_ts, ".xlsx")),
                 row.names = F)
```

save-load point

```
save.image(file = "./saved_data/cipp_intermediate02.RData")
#load(file = "./saved_data/cipp_intermediate02.RData")
```

load a library needed downstream the code

```
# # Enable the r-universe repo
# options(repos = c(
#     fawda123 = 'https://fawda123.r-universe.dev',
#     CRAN = 'https://cloud.r-project.org'))
#
# # Install ggord
# install.packages('ggord')

library(ggord)
```

PCA and RDA on the genus level

PCA

```
library(vegan)
library(BiodiversityR)
```

```
## Loading required package: tcltk
```

```
## BiodiversityR 2.14-1: Use command BiodiversityRGUI() to launch the Graphical User Interface;
## to see changes use BiodiversityRGUI(changeLog=TRUE, backward.compatibility.messages=TRUE)
```

```
otutab <- otu_table(PSMR_G)
colnames(otutab) <- tax_table(PSMR_G)[, 6]
#apply(otutab, 1, sum)

tr_hell_otutab <- decostand(otutab, "hell")

PCAsignificance(rda(tr_hell_otutab))
```

```
##                                        1       2       3       4       5
## eigenvalue                        0.0483  0.0363  0.0286  0.0219  0.0159
## percentage of variance           16.8169 12.6444  9.9425  7.6399  5.5388
## cumulative percentage of variance 16.8169 29.4613 39.4039 47.0438 52.5826
## broken-stick percentage           3.9678  3.2484  2.8887  2.6489  2.4690
## broken-stick cumulative %         3.9678  7.2162 10.1049 12.7538 15.2228
## % > bs%                           1.0000  1.0000  1.0000  1.0000  1.0000
## cum% > bs cum%                    1.0000  1.0000  1.0000  1.0000  1.0000
##                                        6       7        8
## eigenvalue                        0.0148  0.0113  0.00922
## percentage of variance            5.1449  3.9418  3.21009
## cumulative percentage of variance 57.7276 61.6694 64.87951
## broken-stick percentage           2.3251  2.2052  2.10246
## broken-stick cumulative %        17.5480 19.7532 21.85568
## % > bs%                           1.0000  1.0000  1.00000
## cum% > bs cum%                    1.0000  1.0000  1.00000
```

```
pca_res <- princomp(as.matrix((decostand(otu_table(PSMR_G), "hell"))))
#summary(pca_res)

pl_x1 <- ordiplot(pca_res, display = "sites", type = "text")
```

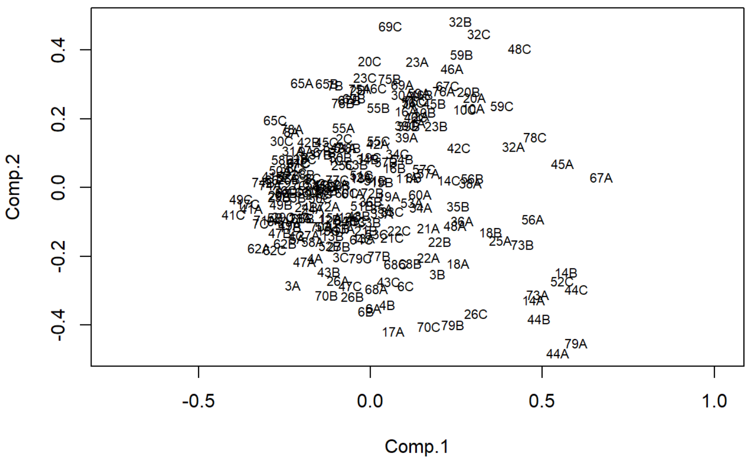


```
pl_x2 <- ordiplot(pca_res, display = "species", type = "text")
```

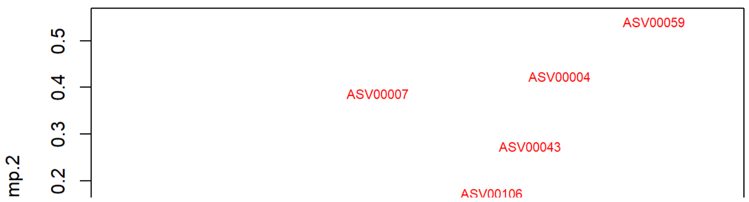

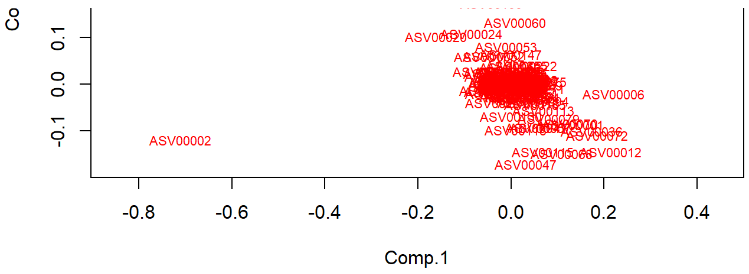

SUPPLEMENTARY FIGURE 3

RDA plot (at the genus level)

```
library(ggord)
p3 <- ggord(rda_res,
      s_data$blasto_or_dientamoeba_pos,
      cols = c("green", "orange"),
      xlims = c(-1.35, 0.5), ylims = c(-1.3, 0.5)) +
    theme(panel.grid.major = element_blank(), panel.grid.minor = element_blank())
# looking at the raw code, this is plotting the 'wa scores', the blue dots are different species

p3$guides$colour$title <- "Blastocystis\nor Dientamoeba"
p3$guides$shape$title <- "Blastocystis\nor Dientamoeba"
p3$guides$fill$title <- "Blastocystis\nor Dientamoeba"

print(p3)
```

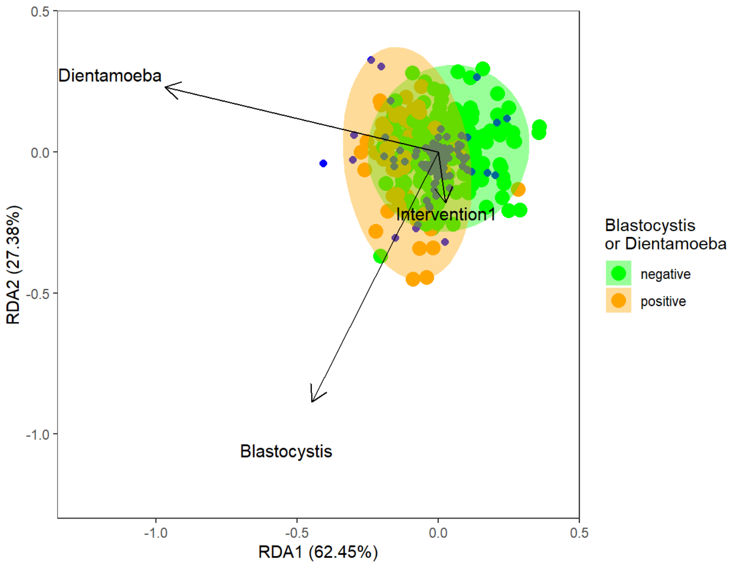


```
#coodinates of bacteria significant in the gee testing
coords_for_text <- rownames_to_column(data.frame(rda_res$CCA$v[, 1:2]), "Genus") %>%
    filter(Genus %in% c("Akkermansia", "Faecalibacterium", "Romboutsia", "Flavonifractor", "Lachnoclostridium",
                        "Ruminococcus", "Granulicatella"))

p4 <- p3 + geom_text(data = coords_for_text,
               mapping = aes(x = RDA1, y = RDA2, label = Genus),
#               position = "dodge"
                vjust = -1,      fontface = "italic"
               )

print(p4)
```

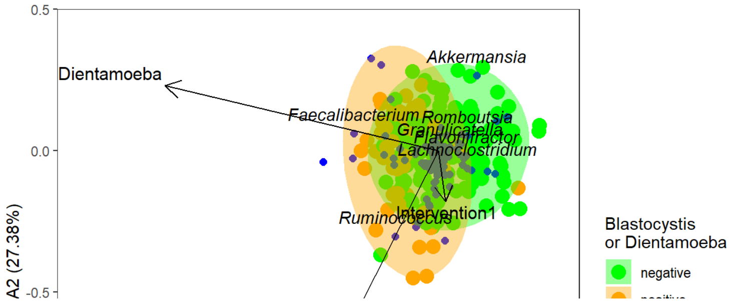

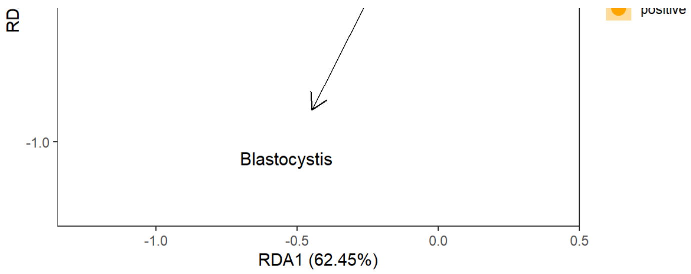

... and one more, now the ASV as the dataset - the result is the same as with genera above

```
otutab_asv <- otu_table(RAR)
tr_hell_otutab_asv <- decostand(otutab_asv, "hell")

rda_res_asv <- rda(
    tr_hell_otutab_asv ~ Blastocystis + Dientamoeba + Intervention, # + Condition(time_abc),
    data = s_data %>%
        mutate(Blastocystis = bl_10, Dientamoeba = di_10, Intervention = on_intervention ))

anova.cca(rda_res_asv, by = "margin")
```

```
## Permutation test for rda under reduced model
## Marginal effects of terms
## Permutation: free
## Number of permutations: 999
##
## Model: rda(formula = tr_hell_otutab_asv ~ Blastocystis + Dientamoeba + Intervention, data = s_data %>% mutate(Blastocystis = bl_10,
Dientamoeba = di_10, Intervention = on_intervention))
##                Df Variance    F Pr(>F)
## Blastocystis   1    0.005 1.88  0.001 ***
## Dientamoeba    1    0.010 3.45  0.001 ***
## Intervention   1    0.004 1.34  0.044 *
## Residual     223    0.629
## ---
## Signif. codes:  0 '***' 0.001 '**' 0.01 '*' 0.05 '.' 0.1 ' ' 1
```

```
p3asv <- ggord(rda_res_asv,
      s_data$intervention,
      cols = c("green", "orange"),
      xlims = c(-0.5, 1.2), ylims = c(-1.0, 0.75)
      ) +
    theme(panel.grid.major = element_blank(), panel.grid.minor = element_blank())
# looking at the raw code, this is plotting the 'wa scores', the blue dots are different species

p3asv$guides$colour$title <- "Blastocystis\nor Dientamoeba"
p3asv$guides$shape$title  <- "Blastocystis\nor Dientamoeba"
p3asv$guides$fill$title   <- "Blastocystis\nor Dientamoeba"

print(p3asv)
```

```
## Warning: Removed 1 rows containing missing values (`geom_text()`).
```

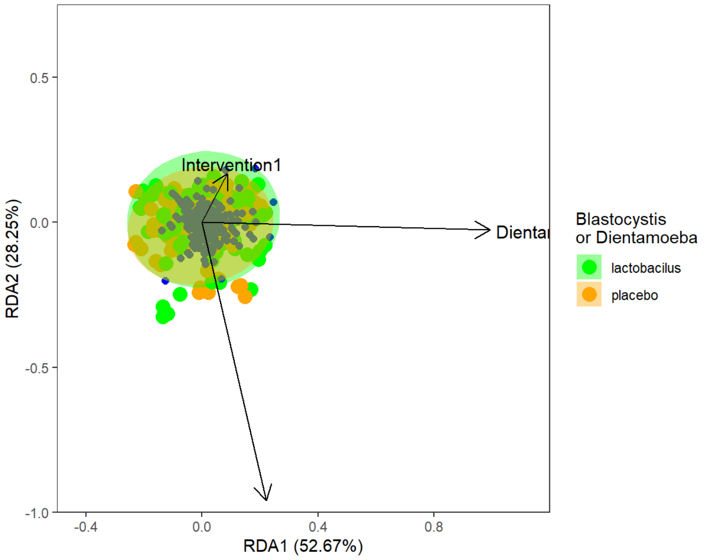

Beta diversity

Transform and calculate distances

```
tr_otutab <- decostand(otutab, "hell")
dist_bray     <- vegdist(tr_otutab, method = "bray")
dist_sorensen <- vegdist(tr_otutab, binary = T) #this is what vegdist does by default when given binary = T
```

PCoA ordination

```
pcoa <- cmdscale(dist_bray, eig = T)
px1 <- ordiplot (pcoa, display = 'sites', type = 'text')
```

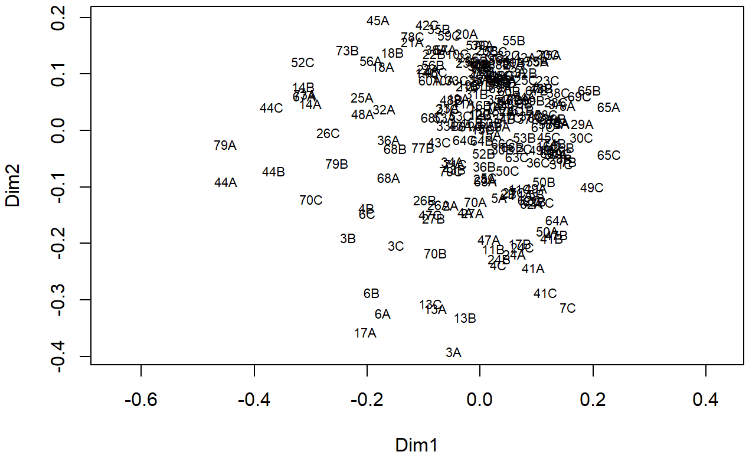

NMDS ordination

```
nmds_bray <- metaMDS(dist_bray)
```

```
## Run 0 stress 0.215
## Run 1 stress 0.215
## ... New best solution
## ... Procrustes: rmse 0.00301  max resid 0.041
## Run 2 stress 0.215
## ... New best solution
## ... Procrustes: rmse 4.26e-05  max resid 0.000506
## ... Similar to previous best
## Run 3 stress 0.215
## ... Procrustes: rmse 0.000898  max resid 0.00945
## ... Similar to previous best
## Run 4 stress 0.215
## Run 5 stress 0.217
## Run 6 stress 0.215
## ... New best solution
## ... Procrustes: rmse 0.000678  max resid 0.00941
## ... Similar to previous best
## Run 7 stress 0.215
## Run 8 stress 0.215
## ... Procrustes: rmse 0.000872  max resid 0.00917
## ... Similar to previous best
## Run 9 stress 0.215
## ... Procrustes: rmse 0.000581  max resid 0.00766
## ... Similar to previous best
## Run 10 stress 0.215
## ... Procrustes: rmse 0.000672  max resid 0.00929
## ... Similar to previous best
## Run 11 stress 0.215
## ... Procrustes: rmse 0.000157  max resid 0.00194
## ... Similar to previous best
## Run 12 stress 0.215
## ... Procrustes: rmse 0.000589  max resid 0.00766
## ... Similar to previous best
## Run 13 stress 0.215
```

```
## ... Procrustes: rmse 0.000677  max resid 0.0094
## ... Similar to previous best
## Run 14 stress 0.215
## ... Procrustes: rmse 0.000582  max resid 0.00764
## ... Similar to previous best
## Run 15 stress 0.215
## ... Procrustes: rmse 4.19e-05  max resid 0.000363
## ... Similar to previous best
## Run 16 stress 0.215
## ... Procrustes: rmse 0.000679  max resid 0.00929
## ... Similar to previous best
## Run 17 stress 0.215
## ... Procrustes: rmse 5.16e-05  max resid 0.000482
## ... Similar to previous best
## Run 18 stress 0.215
## ... New best solution
## ... Procrustes: rmse 2.98e-05  max resid 0.000305
## ... Similar to previous best
## Run 19 stress 0.215
## ... Procrustes: rmse 0.00239  max resid 0.0315
## Run 20 stress 0.215
## ... Procrustes: rmse 0.000669  max resid 0.00931
## ... Similar to previous best
## *** Solution reached
```

```
nmds_bray_points <- data.frame(nmds_bray$points, stringsAsFactors = F)
nmds_bray_points <- merge(x = nmds_bray_points,
                          y = s_data,
                          by = "row.names")

#nmds_sorensen <- metaMDS(dist_sorensen)
#nmds_sorensen_points <- data.frame(nmds_sorensen$points, stringsAsFactors = F)
#nmds_sorensen_points <- merge(x = nmds_sorensen_points, y = s_data, by = "row.names")

p3 <- ggplot(data = nmds_bray_points, mapping = aes(x = MDS1, y = MDS2))
p3 <- p3 + labs(x = "NMDS1", y = "NMDS2")
p3 <- p3 + geom_point(aes(shape = time_abc, color = blastocystis_posneg))
p3 <- p3 + theme(legend.key.height = unit(10, "mm"))
p3 <- p3 + theme(legend.key.width = unit(10, "mm"))
p3 <- p3 + theme(legend.text = element_text(size = 10))
p3 <- p3 + scale_color_manual(
  name = "Blastocystis",
  values = c(positive = "red", negative = "dodger blue" ),
  guide = guide_legend()
  )
p3 <- p3 + scale_fill_discrete(guide = "none") #this removes the legend to the fill colors
#p3 <- p3 + scale_shape_manual(name = "Blastocystis\nPCR",
#                              values = 2:3,
#                              labels = c("neg", "pos"))
p3 #copy 490 * 430
```

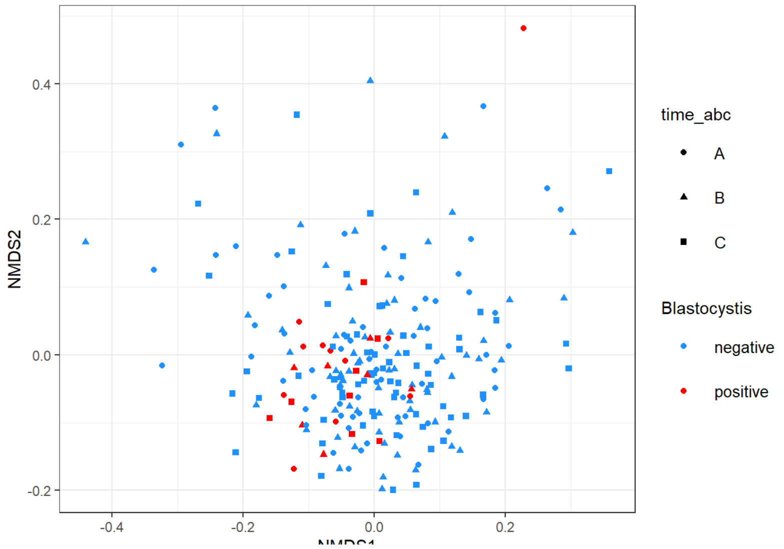

NMDS1

```
ggsave(plot = p3, filename = "NMDS.pdf", width = 11.69, height = 8.27, #A4 landscape
       device = "pdf",
       path = "./pdf_outputs"
       )
```

PERMANOVA + test of homogeneity The dispersion of blastocystis-negatives is much higher than of blastocystis-positives.

```
adonis2(dist_bray ~ blasto_or_dientamoeba_pos,
       data = s_data)
```

```
## Permutation test for adonis under reduced model
## Terms added sequentially (first to last)
## Permutation: free
## Number of permutations: 999
##
## adonis2(formula = dist_bray ~ blasto_or_dientamoeba_pos, data = s_data)
##                            Df SumOfSqs    R2    F Pr(>F)
## blasto_or_dientamoeba_pos   1     0.66 0.029 6.65  0.001 ***
## Residual                  225    22.27 0.971
## Total                     226    22.93 1.000
## ---
## Signif. codes:  0 '***' 0.001 '**' 0.01 '*' 0.05 '.' 0.1 ' ' 1
```

```
homo <- betadisper(dist_bray, s_data$blasto_or_dientamoeba_pos )
plot(homo)
```

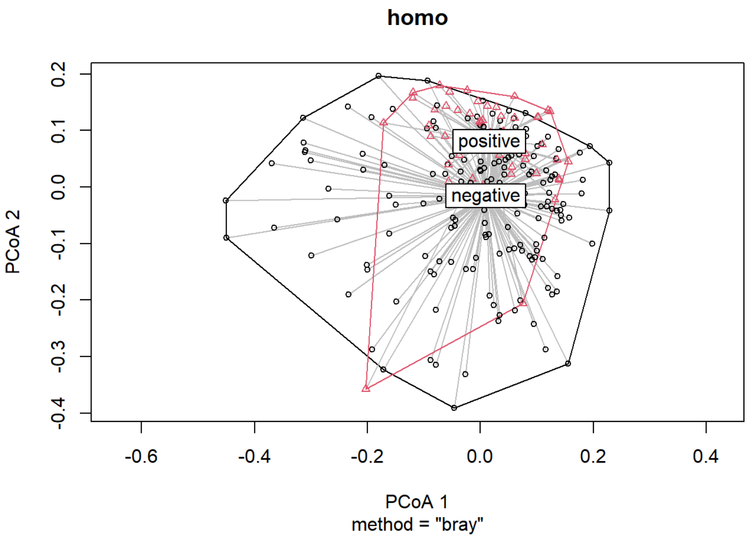


```
permutest(homo)
```

```
##
## Permutation test for homogeneity of multivariate dispersions
## Permutation: free
## Number of permutations: 999
##
## Response: Distances
##            Df Sum Sq Mean Sq    F N.Perm Pr(>F)
## Groups      1   0.11  0.1096 20.4    999  0.001 ***
## Residuals 225   1.21  0.0054
## ---
## Signif. codes:  0 '***' 0.001 '**' 0.01 '*' 0.05 '.' 0.1 ' ' 1
```

```
TukeyHSD(homo)
```

```
##   Tukey multiple comparisons of means
##     95% family-wise confidence level
##
## Fit: aov(formula = distances ~ group, data = df)
##
## $group
##                     diff     lwr     upr p adj
## positive-negative -0.051 -0.0732 -0.0287     0
```

PCoA (MDS)

```
genus_ord <- ordinate(PSMR_G, "MDS", "wunifrac")
p1 = plot_ordination(PSMR_G, genus_ord, type="samples", label = "int_subject_time", color="blastocystis_or_dientamoeba",
title="Samples by parasite positivity") + geom_point(size=3, alpha=0.7) #+ #geom_polygon( alpha = 0.1)
print(p1)
```

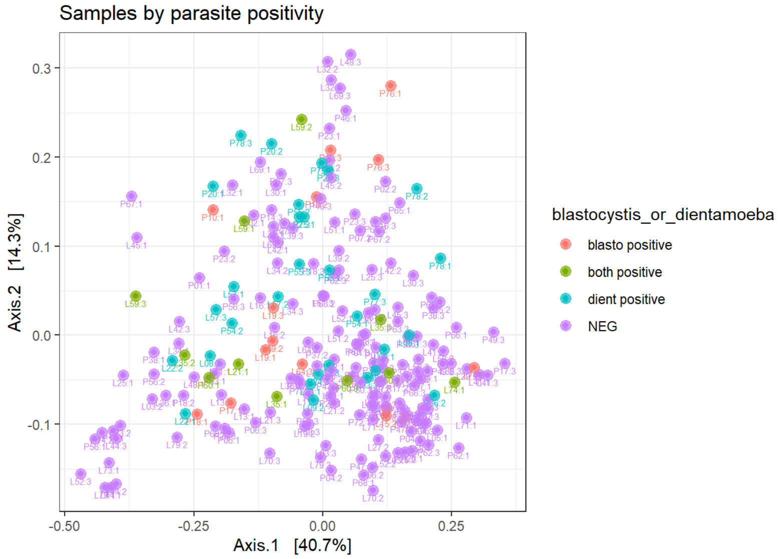

Taxa tested against parasite positivity

This chunk of code defines function used for plotting the taxa and association

```
pa_graph <- function(physeq,
                   x_cat,
                   title = "",
                   Facet = "Genus",
                   Color = "Phylum",
                   my_selection_tax_level = "Genus",
                   my_taxa = character(0)
                   ){

    #CALL: plot_abundance(PH, x_cat = "group",Facet = "Phylum" )

    #This makes one point per sample - tax gloms it...

    physeq <- tax_glom(physeq, taxrank = Facet) #this was missing from the original
    p1f <- transform_sample_counts(physeq, function(x){x / sum(x)})
    mphyseq = psmelt(p1f)
    #phyloseq has the Abundance variable (!)
    mphyseq <- mphyseq[ which(mphyseq[, my_selection_tax_level] %in% my_taxa), ]
    #mphyseq <- subset(mphyseq, Abundance > 0)
    mphyseq$Abundance = mphyseq$Abundance + 1e-5
    ggplot(data = mphyseq, mapping = aes_string(x = x_cat, y = "Abundance",
                                            color = Color, fill = Color)) +
        geom_violin(fill = NA) +
        geom_point(size = 1, alpha = 0.3,
                   position = position_jitter(width = 0.2)) +
        facet_wrap(facets = Facet) +
        scale_y_log10(
            breaks = c(1e-5,  1e-4,     1e-3,  1e-2,  0.1,   1),
            labels = c("neg", "0.01%", "0.1%", "1%", "10%", "100%")
            )+
        theme(axis.text.x = element_text(angle = 90, vjust = 0.5, hjust=1))+
        theme(legend.position="none")
}
```

A linear model, standardisation to the sample total

```
######################################################################
# Testing mixed effect model + gee: Here bacteria are the outcome,
# and the positivity for blastocystis of dientamoeba are the predictors.
#
```

```
#result table
res_parasites <- data.frame()
vr <- 0

my_taxa_levels <- (c("Kingdom", "Phylum", "Class", "Order", "Family", "Genus", "OTU"))

for (tax_level in c(2,3,4,5,6)){

    if (tax_level == 6) {
        PS <- PSMR_G
    } else if (tax_level == 5) {
        PS <- PSMR_F
    } else if (tax_level == 4) {
        PS <- PSMR_O
    } else if (tax_level == 3) {
        PS <- PSMR_C
    } else if (tax_level == 2) {
        PS <- PSMR_P
    } else if (tax_level == 7) {
        PS <- RAR
    } else {
        stop("Nonexistant phylo level")
    }

    taxrank_name <- my_taxa_levels[tax_level]
    total_reads <- max(apply(otu_table(PS), 1, sum))

    otus_for_regression <- merge(x = s_data, y = otu_table(PS), all.x = T, all.y = F, by = "row.names")
    otus_for_regression <- otus_for_regression %>%
        arrange(subject_no, time_123)

    for (i in 1:nrow(tax_table(PS))){
        vr <- vr + 1

        asv = row.names(tax_table(PS))[i]
        otus_for_regression[, "relative_signal"] = otus_for_regression[, asv] / total_reads
        taxon_name = as.character(tax_table(PS)[i, tax_level])

        print("")
        print("-------------- TESTING ------------")
        print(paste(i, asv, taxrank_name, taxon_name))
        res_parasites[vr, "asv"] <- asv
        res_parasites[vr, "taxrank_name"] <- taxrank_name
        res_parasites[vr, "taxon_name"] <- taxon_name


        #GEE
        gee_res<- geepack::geeglm(
            formula = formula(paste( asv, ' ~  blasto_or_dientamoeba_pos +time_23+  on_intervention',
                                     sep = "")),
            id = subject_no,
            data = otus_for_regression,
            family = gaussian,
            corstr = "ar1",
            contrasts = NULL
            )
        summary(gee_res)

        res_parasites[vr, "p_val_gee_blasto_dient_pos"]<- summary(gee_res)$coefficients["blasto_or_dientamoeba_pospositive",
"Pr(>|W|)"]
        res_parasites[vr, "p_val_gee_on_intervention1"]      <- summary(gee_res)$coefficients["on_intervention1", "Pr(>|W|)"]
        res_parasites[vr, "p_val_time_23"]      <- summary(gee_res)$coefficients["time_23", "Pr(>|W|)"]

        res_parasites[vr, "coef_gee_blasto_dient_pos"] <- summary(gee_res)$coefficients["blasto_or_dientamoeba_pospositive",
"Estimate"]
        res_parasites[vr, "coef_gee_on_intervention1"]       <- summary(gee_res)$coefficients["on_intervention1", "Estimate"]
        res_parasites[vr, "coef_gee_time_23"]      <- summary(gee_res)$coefficients["time_23", "Estimate"]

```

```
        #GEE blastocystis
        gee_res2<- geepack::geeglm(
            formula = formula(paste( asv, ' ~  blastocystis_posneg + time_23 +  on_intervention',
                                     sep = "")),
            id = subject_no,
            data = otus_for_regression,
            family = gaussian,
            corstr = "ar1",
            contrasts = NULL
            )
        summary(gee_res2)

        res_parasites[vr, "p_val_gee_blastocystis_positive"]<- summary(gee_res2)$coefficients["blastocystis_posnegpositive",
"Pr(>|W|)"]
        res_parasites[vr, "coef_gee_blastocystis_positive"] <- summary(gee_res2)$coefficients["blastocystis_posnegpositive",
"Estimate"]

        #GEE dientamoeba
        gee_res3<- geepack::geeglm(
            formula = formula(paste( asv, ' ~  dientamoeba_posneg + time_23 +  on_intervention',
                                     sep = "")),
            id = subject_no,
            data = otus_for_regression,
            family = gaussian,
            corstr = "ar1",
            contrasts = NULL
            )
        summary(gee_res3)

        res_parasites[vr, "p_val_gee_dientamoeba_positive"]<- summary(gee_res3)$coefficients["dientamoeba_posnegpositive", "Pr(>|W|)"]
        res_parasites[vr, "coef_gee_dientamoeba_positive"] <- summary(gee_res3)$coefficients["dientamoeba_posnegpositive", "Estimate"]


         if (res_parasites[vr, "p_val_gee_dientamoeba_positive"] < 0.001 |
            res_parasites[vr, "p_val_gee_blastocystis_positive"] < 0.001 |
            res_parasites[vr, "p_val_gee_blasto_dient_pos"] < 0.001)
            {

            px <- pa_graph(PS,
                   Facet  = taxrank_name,
                   my_selection_tax_level = taxrank_name,
                   x_cat = "blastocystis_or_dientamoeba",
                   my_taxa = taxon_name,
                   title = paste(taxrank_name, taxon_name))
            cat("p_val_gee_dientamoeba_positive", res_parasites[vr, "p_val_gee_dientamoeba_positive"], "\n")
            cat("p_val_gee_blastocystis_positive", res_parasites[vr, "p_val_gee_blastocystis_positive"], "\n")
            cat("p_val_gee_blasto_dient_pos", res_parasites[vr, "p_val_gee_blasto_dient_pos"], "\n")
            print(px)
        }


    }
}
```

```
## [1] ""
## [1] "-------------- TESTING ------------"
## [1] "1 ASV00002 Phylum Bacteroidota"
## [1] ""
## [1] "-------------- TESTING ------------"
## [1] "2 ASV01987 Phylum Fusobacteriota"
## [1] ""
## [1] "-------------- TESTING ------------"
## [1] "3 ASV00041 Phylum Proteobacteria"
## [1] ""
## [1] "-------------- TESTING ------------"
## [1] "4 ASV01371 Phylum Patescibacteria"
```

```
## [1] ""
## [1] "-------------- TESTING ------------"
## [1] "5 ASV00094 Phylum Actinobacteriota"
## [1] ""
## [1] "-------------- TESTING ------------"
## [1] "6 ASV00734 Phylum Cyanobacteria"
## [1] ""
## [1] "-------------- TESTING ------------"
## [1] "7 ASV00024 Phylum Verrucomicrobiota"
## p_val_gee_dientamoeba_positive 0.0191
## p_val_gee_blastocystis_positive 6.31e-06
## p_val_gee_blasto_dient_pos 0.00137
```

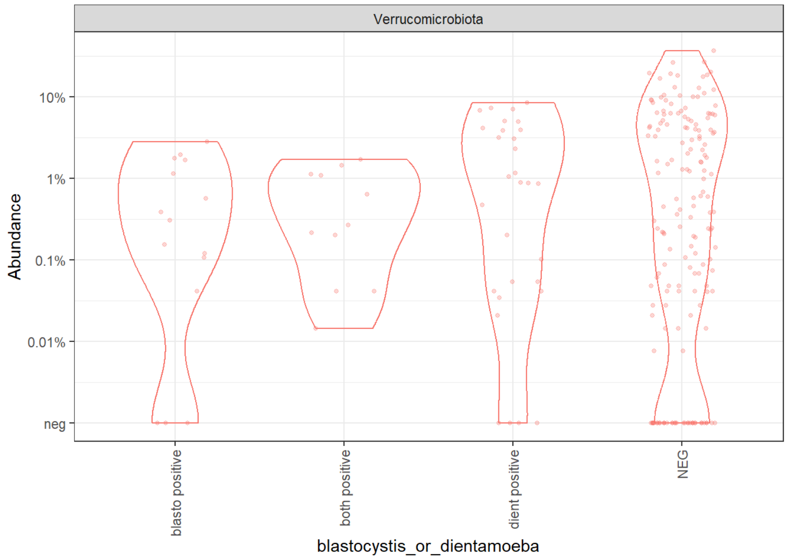


```
## [1] ""
## [1] "-------------- TESTING ------------"
## [1] "8 ASV00614 Phylum Desulfobacterota"
## [1] ""
## [1] "-------------- TESTING ------------"
## [1] "9 ASV00001 Phylum Firmicutes"
## [1] ""
## [1] "-------------- TESTING ------------"
## [1] "1 ASV00002 Class Bacteroidia"
## [1] ""
## [1] "-------------- TESTING ------------"
## [1] "2 ASV01987 Class Fusobacteriia"
## [1] ""
## [1] "-------------- TESTING ------------"
## [1] "3 ASV01698 Class Lentisphaeria"
## [1] ""
## [1] "-------------- TESTING ------------"
## [1] "4 ASV00041 Class Gammaproteobacteria"
## [1] ""
## [1] "-------------- TESTING ------------"
## [1] "5 ASV00557 Class Alphaproteobacteria"
## [1] ""
## [1] "-------------- TESTING ------------"
## [1] "6 ASV01371 Class Saccharimonadia"
## [1] ""
## [1] "-------------- TESTING ------------"
## [1] "7 ASV01337 Class Cyanobacteriia"
## [1] ""
## [1] "-------------- TESTING ------------"
## [1] "8 ASV00094 Class Actinobacteria"
## [1] ""
## [1] "-------------- TESTING ------------"
## [1] "9 ASV00283 Class Coriobacteriia"
## [1] ""
## [1] "-------------- TESTING ------------"
## [1] "10 ASV00734 Class Vampirivibrionia"
```

```
## [1] ""
## [1] "-------------- TESTING ------------"
## [1] "11 ASV00024 Class Verrucomicrobiae"
## p_val_gee_dientamoeba_positive 0.0214
## p_val_gee_blastocystis_positive 7.21e-06
## p_val_gee_blasto_dient_pos 0.00164
```

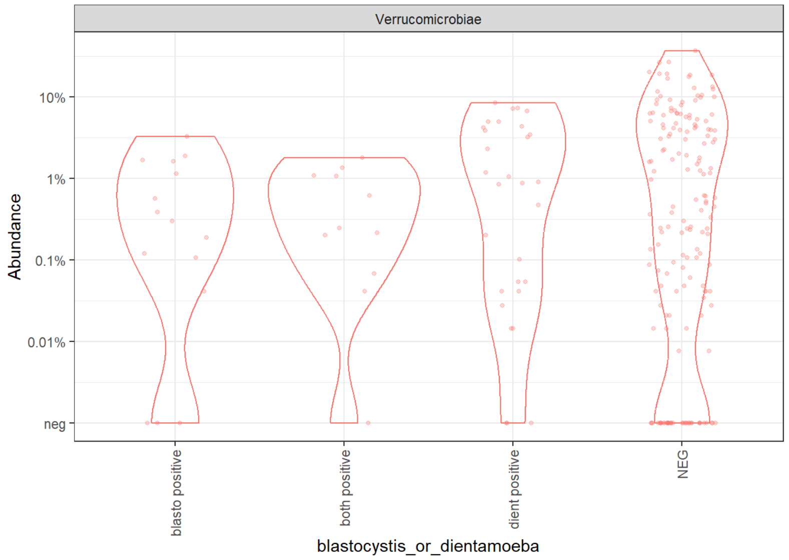


```
## [1] ""
## [1] "-------------- TESTING ------------"
## [1] "12 ASV00614 Class Desulfovibrionia"
## [1] ""
## [1] "-------------- TESTING ------------"
## [1] "13 ASV00079 Class Bacilli"
## [1] ""
## [1] "-------------- TESTING ------------"
## [1] "14 ASV00004 Class Negativicutes"
## [1] ""
## [1] "-------------- TESTING ------------"
## [1] "15 ASV01456 Class Incertae Sedis"
## [1] ""
## [1] "-------------- TESTING ------------"
## [1] "16 ASV00001 Class Clostridia"
## [1] ""
## [1] "-------------- TESTING ------------"
## [1] "1 ASV00012 Order Lachnospirales"
## [1] ""
## [1] "-------------- TESTING ------------"
## [1] "2 ASV01487 Order Flavobacteriales"
## [1] ""
## [1] "-------------- TESTING ------------"
## [1] "3 ASV00002 Order Bacteroidales"
## [1] ""
## [1] "-------------- TESTING ------------"
## [1] "4 ASV01987 Order Fusobacteriales"
## [1] ""
## [1] "-------------- TESTING ------------"
## [1] "5 ASV01698 Order Victivallales"
## [1] ""
## [1] "-------------- TESTING ------------"
## [1] "6 ASV00214 Order Burkholderiales"
## [1] ""
## [1] "-------------- TESTING ------------"
## [1] "7 ASV00041 Order Enterobacterales"
## [1] ""
## [1] "-------------- TESTING ------------"
## [1] "8 ASV00557 Order Rhodospirillales"
## [1] ""
## [1] "-------------- TESTING ------------"
## [1] "9 ASV01371 Order Saccharimonadales"
```

```
## [1] ""
## [1] "-------------- TESTING ------------"
## [1] "10 ASV01337 Order Chloroplast"
## [1] ""
## [1] "-------------- TESTING ------------"
## [1] "11 ASV01546 Order Actinomycetales"
## [1] ""
## [1] "-------------- TESTING ------------"
## [1] "12 ASV00094 Order Bifidobacteriales"
## [1] ""
## [1] "-------------- TESTING ------------"
## [1] "13 ASV00283 Order Coriobacteriales"
## [1] ""
## [1] "-------------- TESTING ------------"
## [1] "14 ASV00734 Order Gastranaerophilales"
## [1] ""
## [1] "-------------- TESTING ------------"
## [1] "15 ASV00024 Order Verrucomicrobiales"
## p_val_gee_dientamoeba_positive 0.021
## p_val_gee_blastocystis_positive 7.04e-06
## p_val_gee_blasto_dient_pos 0.00154
```

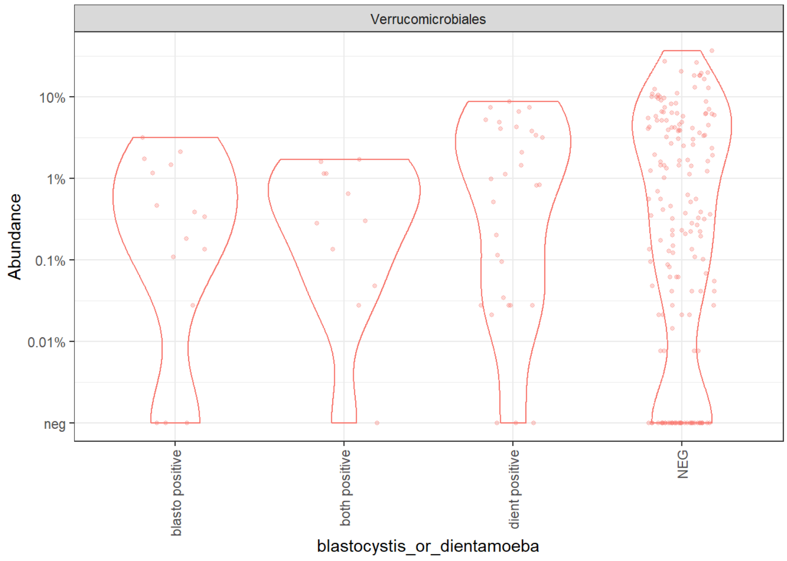


```
## [1] ""
## [1] "-------------- TESTING ------------"
## [1] "16 ASV00614 Order Desulfovibrionales"
## [1] ""
## [1] "-------------- TESTING ------------"
## [1] "17 ASV01322 Order Izemoplasmatales"
## [1] ""
## [1] "-------------- TESTING ------------"
## [1] "18 ASV00717 Order RF39"
## [1] ""
## [1] "-------------- TESTING ------------"
## [1] "19 ASV00079 Order Erysipelotrichales"
## [1] ""
## [1] "-------------- TESTING ------------"
## [1] "20 ASV00185 Order Lactobacillales"
## [1] ""
## [1] "-------------- TESTING ------------"
## [1] "21 ASV01883 Order Staphylococcales"
## [1] ""
## [1] "-------------- TESTING ------------"
## [1] "22 ASV00004 Order Veillonellales-Selenomonadales"
## [1] ""
## [1] "-------------- TESTING ------------"
## [1] "23 ASV00081 Order Acidaminococcales"
## [1] ""
## [1] "-------------- TESTING ------------"
## [1] "24 ASV00982 Order Peptococcales"
```

```
## [1] ""
## [1] "-------------- TESTING ------------"
## [1] "25 ASV02228 Order Eubacteriales"
## [1] ""
## [1] "-------------- TESTING ------------"
## [1] "26 ASV00036 Order Peptostreptococcales-Tissierellales"
## [1] ""
## [1] "-------------- TESTING ------------"
## [1] "27 ASV01456 Order DTU014"
## [1] ""
## [1] "-------------- TESTING ------------"
## [1] "28 ASV01140 Order Clostridia vadinBB60 group"
## [1] ""
## [1] "-------------- TESTING ------------"
## [1] "29 ASV00053 Order Christensenellales"
## [1] ""
## [1] "-------------- TESTING ------------"
## [1] "30 ASV00116 Order Monoglobales"
## [1] ""
## [1] "-------------- TESTING ------------"
## [1] "31 ASV00001 Order Oscillospirales"
## [1] ""
## [1] "-------------- TESTING ------------"
## [1] "32 ASV00208 Order Clostridia UCG-014"
## [1] ""
## [1] "-------------- TESTING ------------"
## [1] "33 ASV00072 Order Clostridiales"
## [1] ""
## [1] "-------------- TESTING ------------"
## [1] "1 ASV00012 Family Lachnospiraceae"
## [1] ""
## [1] "-------------- TESTING ------------"
## [1] "2 ASV00007 Family Rikenellaceae"
## [1] ""
## [1] "-------------- TESTING ------------"
## [1] "3 ASV01487 Family Flavobacteriaceae"
## [1] ""
## [1] "-------------- TESTING ------------"
## [1] "4 ASV00177 Family Marinifilaceae"
## [1] ""
## [1] "-------------- TESTING ------------"
## [1] "5 ASV01880 Family Dysgonomonadaceae"
## [1] ""
## [1] "-------------- TESTING ------------"
## [1] "6 ASV00059 Family Prevotellaceae"
## [1] ""
## [1] "-------------- TESTING ------------"
## [1] "7 ASV00106 Family Barnesiellaceae"
## [1] ""
## [1] "-------------- TESTING ------------"
## [1] "8 ASV00460 Family Muribaculaceae"
## [1] ""
## [1] "-------------- TESTING ------------"
## [1] "9 ASV00528 Family Porphyromonadaceae"
## [1] ""
## [1] "-------------- TESTING ------------"
## [1] "10 ASV00002 Family Bacteroidaceae"
## [1] ""
## [1] "-------------- TESTING ------------"
## [1] "11 ASV00020 Family Tannerellaceae"
## [1] ""
## [1] "-------------- TESTING ------------"
## [1] "12 ASV00269 Family Butyricicoccaceae"
## [1] ""
## [1] "-------------- TESTING ------------"
## [1] "13 ASV00044 Family [Eubacterium] coprostanoligenes group"
## [1] ""
```

```
## [1] "-------------- TESTING ------------"
## [1] "14 ASV01407 Family Defluviitaleaceae"
## [1] ""
## [1] "-------------- TESTING ------------"
## [1] "15 ASV01987 Family Fusobacteriaceae"
## [1] ""
## [1] "-------------- TESTING ------------"
## [1] "16 ASV01698 Family vadinBE97"
## [1] ""
## [1] "-------------- TESTING ------------"
## [1] "17 ASV00214 Family Sutterellaceae"
## [1] ""
## [1] "-------------- TESTING ------------"
## [1] "18 ASV00988 Family Oxalobacteraceae"
## [1] ""
## [1] "-------------- TESTING ------------"
## [1] "19 ASV00068 Family Pasteurellaceae"
## [1] ""
## [1] "-------------- TESTING ------------"
## [1] "20 ASV00041 Family Enterobacteriaceae"
## p_val_gee_dientamoeba_positive 0.000321
## p_val_gee_blastocystis_positive 0.00279
## p_val_gee_blasto_dient_pos 0.00066
```

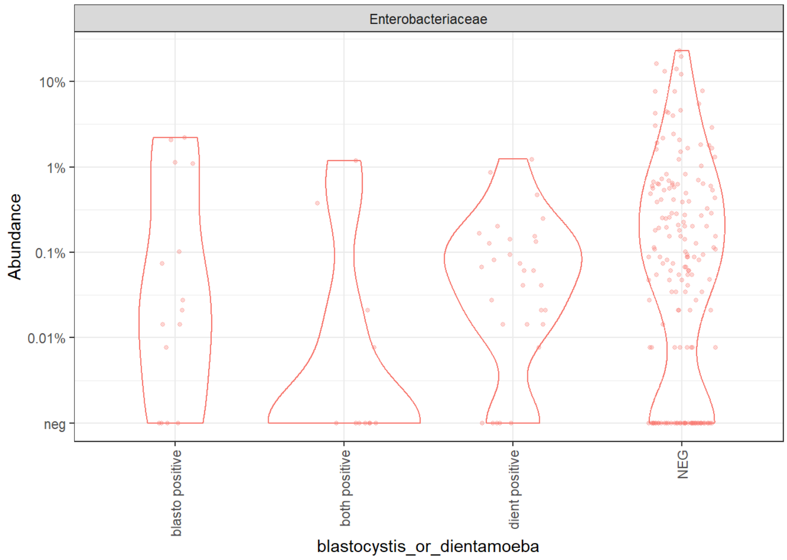


```
## [1] ""
## [1] "-------------- TESTING ------------"
## [1] "21 ASV01371 Family Saccharimonadaceae"
## [1] ""
## [1] "-------------- TESTING ------------"
## [1] "22 ASV01546 Family Actinomycetaceae"
## [1] ""
## [1] "-------------- TESTING ------------"
## [1] "23 ASV00094 Family Bifidobacteriaceae"
## [1] ""
## [1] "-------------- TESTING ------------"
## [1] "24 ASV01145 Family Eggerthellaceae"
## [1] ""
## [1] "-------------- TESTING ------------"
## [1] "25 ASV00452 Family Coriobacteriales Incertae Sedis"
## [1] ""
## [1] "-------------- TESTING ------------"
## [1] "26 ASV00283 Family Coriobacteriaceae"
## p_val_gee_dientamoeba_positive 0.000435
## p_val_gee_blastocystis_positive 0.946
## p_val_gee_blasto_dient_pos 0.122
```

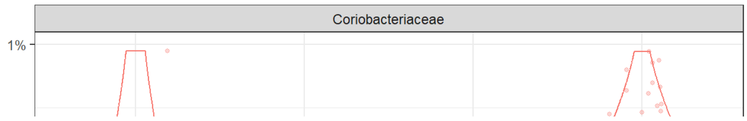

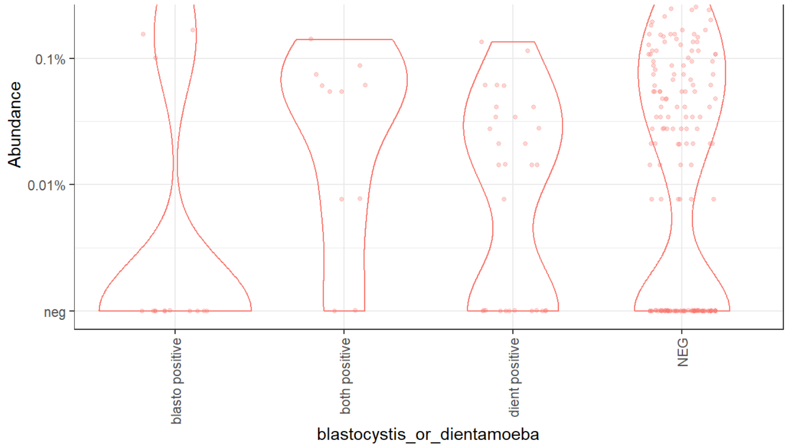


```
## [1] ""
## [1] "-------------- TESTING ------------"
## [1] "27 ASV00024 Family Akkermansiaceae"
## p_val_gee_dientamoeba_positive 0.0284
## p_val_gee_blastocystis_positive 4.7e-06
## p_val_gee_blasto_dient_pos 0.00191
```

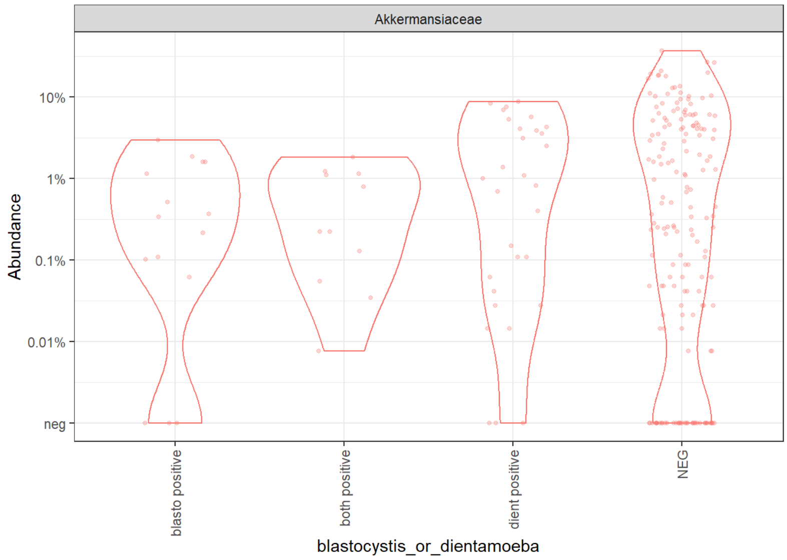


```
## [1] ""
## [1] "-------------- TESTING ------------"
## [1] "28 ASV00614 Family Desulfovibrionaceae"
## [1] ""
## [1] "-------------- TESTING ------------"
## [1] "29 ASV00113 Family Erysipelatoclostridiaceae"
## [1] ""
## [1] "-------------- TESTING ------------"
## [1] "30 ASV00079 Family Erysipelotrichaceae"
## [1] ""
## [1] "-------------- TESTING ------------"
## [1] "31 ASV00185 Family Streptococcaceae"
## [1] ""
## [1] "-------------- TESTING ------------"
## [1] "32 ASV00796 Family Lactobacillaceae"
## [1] ""
## [1] "-------------- TESTING ------------"
## [1] "33 ASV00583 Family Carnobacteriaceae"
## p_val_gee_dientamoeba_positive 0.000258
## p_val_gee_blastocystis_positive 0.00796
## p_val_gee_blasto_dient_pos 0.000486
```

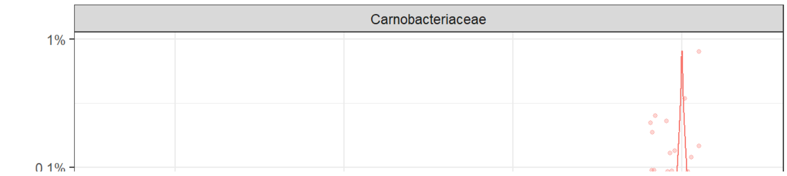

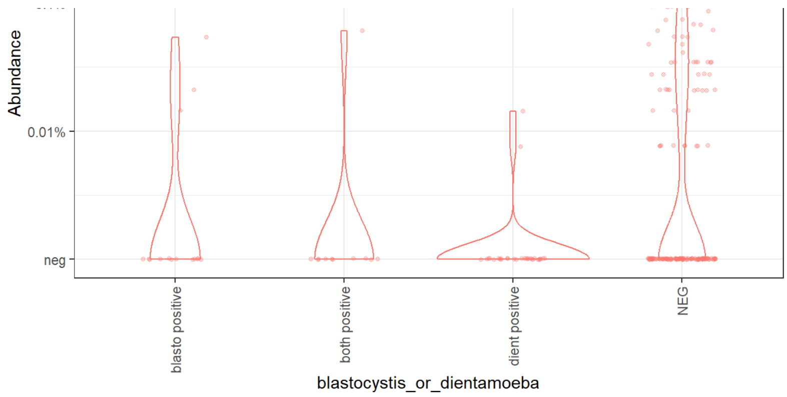


```
## [1] ""
## [1] "-------------- TESTING ------------"
## [1] "34 ASV01756 Family Aerococcaceae"
## [1] ""
## [1] "-------------- TESTING ------------"
## [1] "35 ASV01883 Family Gemellaceae"
## [1] ""
## [1] "-------------- TESTING ------------"
## [1] "36 ASV00004 Family Veillonellaceae"
## [1] ""
## [1] "-------------- TESTING ------------"
## [1] "37 ASV00744 Family Selenomonadaceae"
## [1] ""
## [1] "-------------- TESTING ------------"
## [1] "38 ASV00081 Family Acidaminococcaceae"
## [1] ""
## [1] "-------------- TESTING ------------"
## [1] "39 ASV00982 Family Peptococcaceae"
## [1] ""
## [1] "-------------- TESTING ------------"
## [1] "40 ASV02228 Family Anaerofustaceae"
## p_val_gee_dientamoeba_positive 0.000937
## p_val_gee_blastocystis_positive 0.0034
## p_val_gee_blasto_dient_pos 0.000801
```

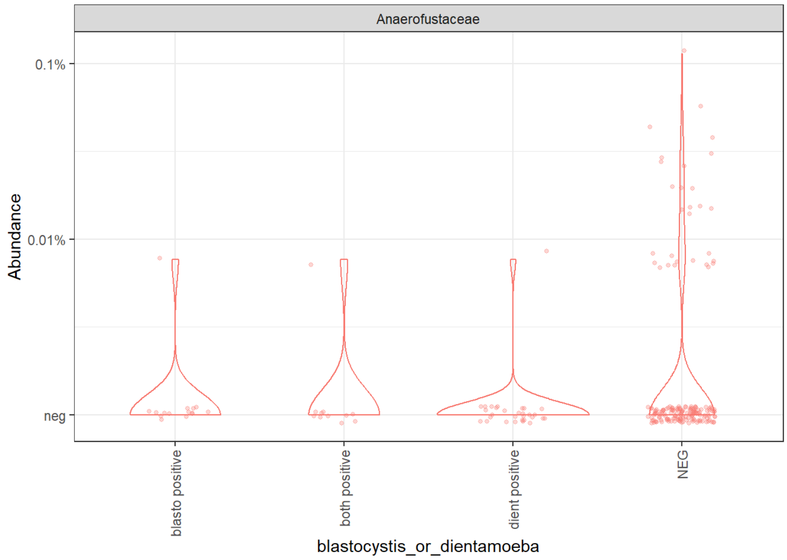


```
## [1] ""
## [1] "-------------- TESTING ------------"
## [1] "41 ASV01197 Family Family XI"
## [1] ""
## [1] "-------------- TESTING ------------"
## [1] "42 ASV00350 Family Anaerovoraceae"
## [1] ""
## [1] "-------------- TESTING ------------"
## [1] "43 ASV00036 Family Peptostreptococcaceae"
## [1] ""
## [1] "-------------- TESTING ------------"
```

```
## [1] "44 ASV00053 Family Christensenellaceae"
## [1] ""
## [1] "-------------- TESTING ------------"
## [1] "45 ASV02142 Family UCG-011"
## [1] ""
## [1] "-------------- TESTING ------------"
## [1] "46 ASV00116 Family Monoglobaceae"
## [1] ""
## [1] "-------------- TESTING ------------"
## [1] "47 ASV00043 Family Oscillospiraceae"
## [1] ""
## [1] "-------------- TESTING ------------"
## [1] "48 ASV00611 Family UCG-010"
## p_val_gee_dientamoeba_positive 0.00127
## p_val_gee_blastocystis_positive 0.0477
## p_val_gee_blasto_dient_pos 0.000701
```

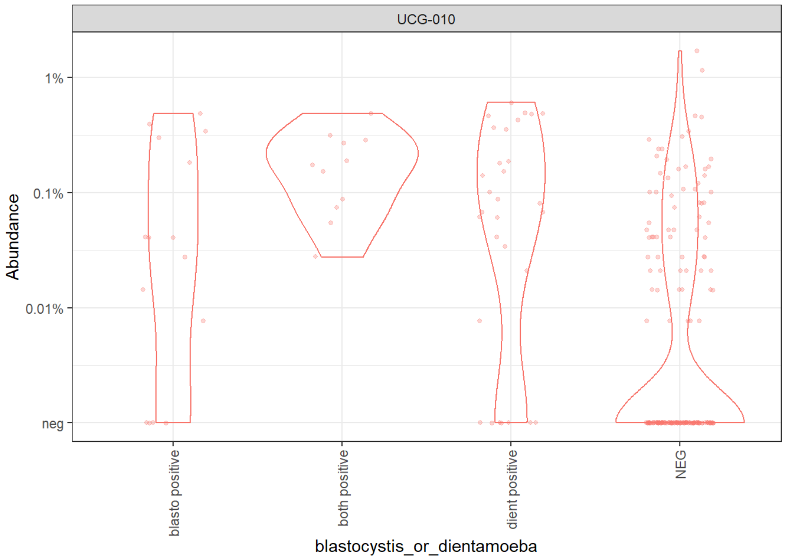


```
## [1] ""
## [1] "-------------- TESTING ------------"
## [1] "49 ASV00001 Family Ruminococcaceae"
## [1] ""
## [1] "-------------- TESTING ------------"
## [1] "50 ASV00072 Family Clostridiaceae"
## [1] ""
## [1] "-------------- TESTING ------------"
## [1] "1 ASV00642 Genus UC5-1-2E3"
## [1] ""
## [1] "-------------- TESTING ------------"
## [1] "2 ASV00295 Genus Lachnospiraceae UCG-004"
## [1] ""
## [1] "-------------- TESTING ------------"
## [1] "3 ASV00166 Genus Fusicatenibacter"
## [1] ""
## [1] "-------------- TESTING ------------"
## [1] "4 ASV00135 Genus Eisenbergiella"
## [1] ""
## [1] "-------------- TESTING ------------"
## [1] "5 ASV00047 Genus [Ruminococcus] gnavus group"
## [1] ""
## [1] "-------------- TESTING ------------"
## [1] "6 ASV00070 Genus Dorea"
## [1] ""
## [1] "-------------- TESTING ------------"
## [1] "7 ASV00459 Genus CAG-56"
## [1] ""
## [1] "-------------- TESTING ------------"
## [1] "8 ASV00559 Genus Lachnospiraceae FCS020 group"
## [1] ""
## [1] "-------------- TESTING ------------"
```

```
## [1] "9 ASV01751 Genus Marvinbryantia"
## [1] ""
## [1] "-------------- TESTING ------------"
## [1] "10 ASV00118 Genus [Ruminococcus] torques group"
## p_val_gee_dientamoeba_positive 1.65e-07
## p_val_gee_blastocystis_positive 0.115
## p_val_gee_blasto_dient_pos 0.000326
```

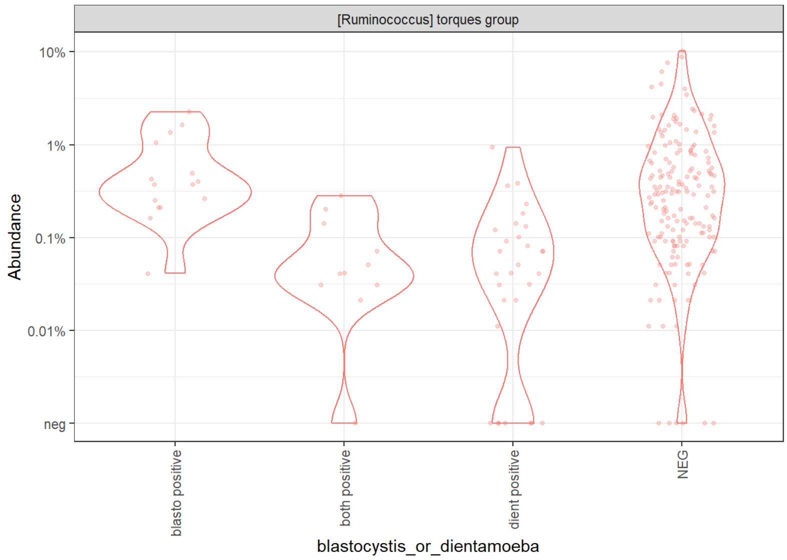


```
## [1] ""
## [1] "-------------- TESTING ------------"
## [1] "11 ASV00754 Genus Sellimonas"
## p_val_gee_dientamoeba_positive 0.000471
## p_val_gee_blastocystis_positive 0.161
## p_val_gee_blasto_dient_pos 0.776
```

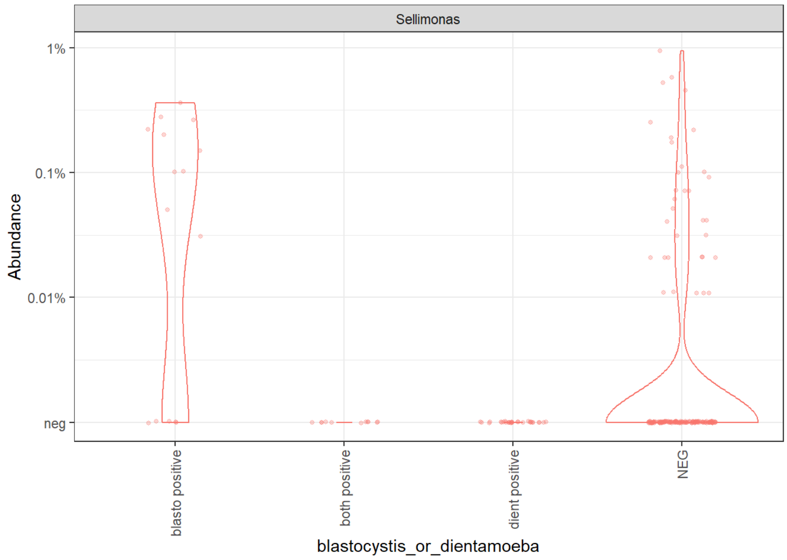


```
## [1] ""
## [1] "-------------- TESTING ------------"
## [1] "12 ASV00398 Genus [Ruminococcus] gauvreauii group"
## [1] ""
## [1] "-------------- TESTING ------------"
## [1] "13 ASV00317 Genus Lachnospiraceae UCG-001"
## [1] ""
## [1] "-------------- TESTING ------------"
## [1] "14 ASV00012 Genus Agathobacter"
## [1] ""
## [1] "-------------- TESTING ------------"
## [1] "15 ASV00057 Genus Roseburia"
## [1] ""
## [1] "-------------- TESTING ------------"
## [1] "16 ASV00975 Genus [Eubacterium] fissicatena group"
```

```
## p_val_gee_dientamoeba_positive 0.000299
## p_val_gee_blastocystis_positive 0.0032
## p_val_gee_blasto_dient_pos 0.000742
```

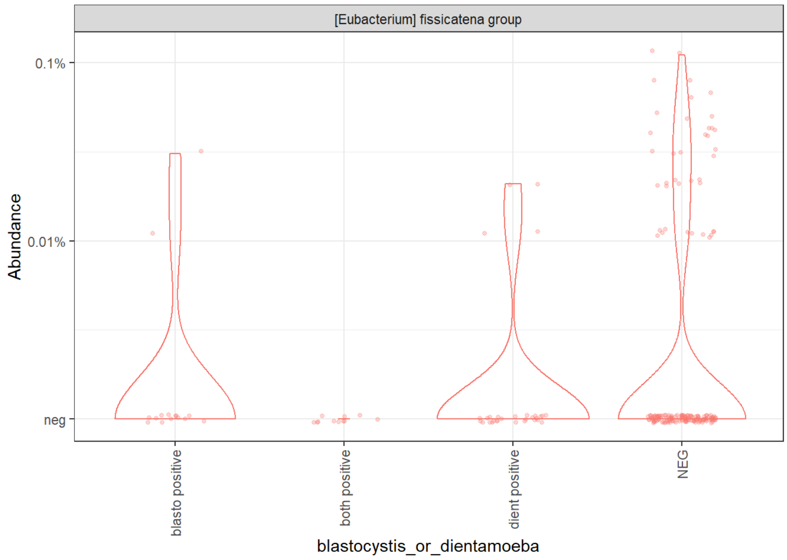


```
## [1] ""
## [1] "-------------- TESTING ------------"
## [1] "17 ASV00007 Genus Alistipes"
## [1] ""
## [1] "-------------- TESTING ------------"
## [1] "18 ASV00099 Genus Blautia"
## [1] ""
## [1] "-------------- TESTING ------------"
## [1] "19 ASV00577 Genus Lachnospiraceae UCG-003"
## [1] ""
## [1] "-------------- TESTING ------------"
## [1] "20 ASV00513 Genus [Eubacterium] hallii group"
## [1] ""
## [1] "-------------- TESTING ------------"
## [1] "21 ASV00358 Genus Rikenellaceae RC9 gut group"
## [1] ""
## [1] "-------------- TESTING ------------"
## [1] "22 ASV01122 Genus Butyricimonas"
## [1] ""
## [1] "-------------- TESTING ------------"
## [1] "23 ASV00177 Genus Odoribacter"
## [1] ""
## [1] "-------------- TESTING ------------"
## [1] "24 ASV00508 Genus Paraprevotella"
## [1] ""
## [1] "-------------- TESTING ------------"
## [1] "25 ASV00120 Genus Prevotella_7"
## [1] ""
## [1] "-------------- TESTING ------------"
## [1] "26 ASV01175 Genus Prevotella"
## [1] ""
## [1] "-------------- TESTING ------------"
## [1] "27 ASV00059 Genus Prevotella_9"
## [1] ""
## [1] "-------------- TESTING ------------"
## [1] "28 ASV01319 Genus Coprobacter"
## [1] ""
## [1] "-------------- TESTING ------------"
## [1] "29 ASV00106 Genus Barnesiella"
## [1] ""
## [1] "-------------- TESTING ------------"
## [1] "30 ASV00528 Genus Porphyromonas"
## [1] ""
## [1] "-------------- TESTING ------------"
## [1] "31 ASV00002 Genus Bacteroides"
```

```
## [1] ""
## [1] "-------------- TESTING ------------"
## [1] "32 ASV00020 Genus Parabacteroides"
## [1] ""
## [1] "-------------- TESTING ------------"
## [1] "33 ASV00056 Genus Lachnospiraceae NK4A136 group"
## [1] ""
## [1] "-------------- TESTING ------------"
## [1] "34 ASV00130 Genus Lachnoclostridium"
## p_val_gee_dientamoeba_positive 0.000208
## p_val_gee_blastocystis_positive 0.62
## p_val_gee_blasto_dient_pos 0.695
```

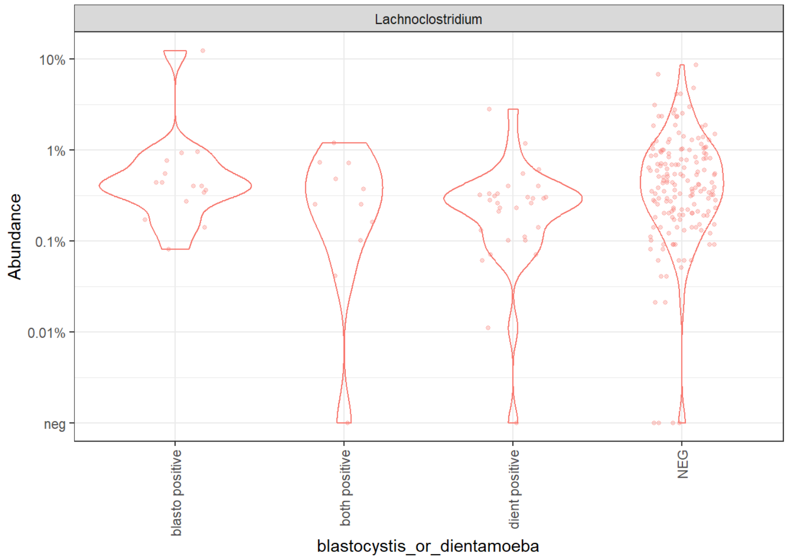


```
## [1] ""
## [1] "-------------- TESTING ------------"
## [1] "35 ASV00399 Genus Lachnospiraceae AC2044 group"
## [1] ""
## [1] "-------------- TESTING ------------"
## [1] "36 ASV00244 Genus Hungatella"
## [1] ""
## [1] "-------------- TESTING ------------"
## [1] "37 ASV01206 Genus Howardella"
## [1] ""
## [1] "-------------- TESTING ------------"
## [1] "38 ASV00124 Genus [Eubacterium] xylanophilum group"
## [1] ""
## [1] "-------------- TESTING ------------"
## [1] "39 ASV00102 Genus [Eubacterium] eligens group"
## p_val_gee_dientamoeba_positive 0.761
## p_val_gee_blastocystis_positive 0.000389
## p_val_gee_blasto_dient_pos 0.0261
```

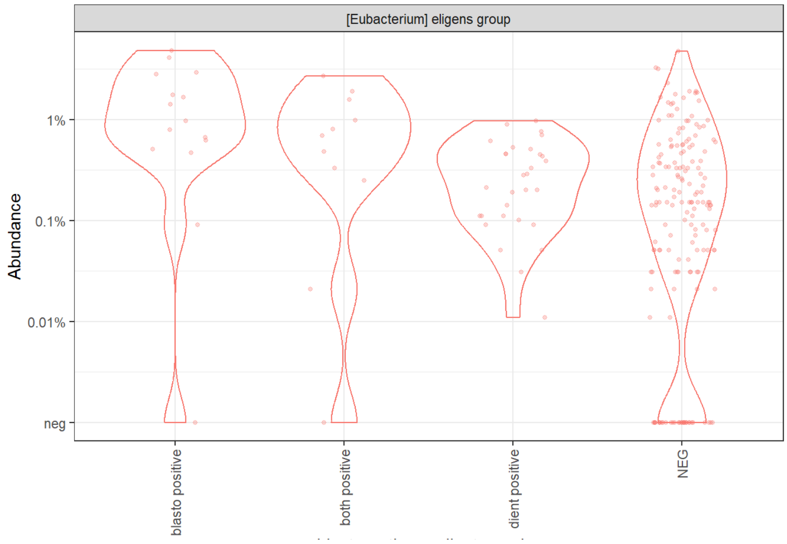

blastocystis_or_dientamoeba

```
## [1] ""
## [1] "-------------- TESTING ------------"
## [1] "40 ASV00209 Genus Lachnospira"
## [1] ""
## [1] "-------------- TESTING ------------"
## [1] "41 ASV00361 Genus Lachnospiraceae ND3007 group"
## [1] ""
## [1] "-------------- TESTING ------------"
## [1] "42 ASV00355 Genus [Eubacterium] ruminantium group"
## [1] ""
## [1] "-------------- TESTING ------------"
## [1] "43 ASV00075 Genus Coprococcus"
## [1] ""
## [1] "-------------- TESTING ------------"
## [1] "44 ASV00182 Genus Anaerostipes"
## p_val_gee_dientamoeba_positive 0.000647
## p_val_gee_blastocystis_positive 0.369
## p_val_gee_blasto_dient_pos 0.367
```

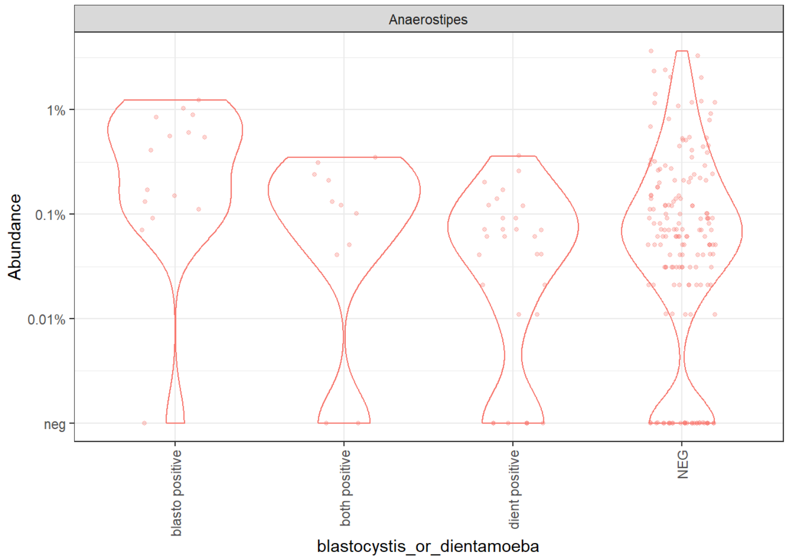


```
## [1] ""
## [1] "-------------- TESTING ------------"
## [1] "45 ASV00271 Genus [Eubacterium] ventriosum group"
## [1] ""
## [1] "-------------- TESTING ------------"
## [1] "46 ASV01264 Genus Frisingicoccus"
## [1] ""
## [1] "-------------- TESTING ------------"
## [1] "47 ASV01262 Genus GCA-900066575"
## [1] ""
## [1] "-------------- TESTING ------------"
## [1] "48 ASV00269 Genus Butyricicoccus"
## [1] ""
## [1] "-------------- TESTING ------------"
## [1] "49 ASV01407 Genus Defluviitaleaceae UCG-011"
## [1] ""
## [1] "-------------- TESTING ------------"
## [1] "50 ASV00515 Genus Lachnospiraceae UCG-010"
## [1] ""
## [1] "-------------- TESTING ------------"
## [1] "51 ASV00802 Genus Tuzzerella"
## [1] ""
## [1] "-------------- TESTING ------------"
## [1] "52 ASV01987 Genus Fusobacterium"
## [1] ""
## [1] "-------------- TESTING ------------"
## [1] "53 ASV00273 Genus Parasutterella"
## [1] ""
## [1] "-------------- TESTING ------------"
```

```
## [1] "54 ASV00214 Genus Sutterella"
## [1] ""
## [1] "-------------- TESTING ------------"
## [1] "55 ASV00988 Genus Oxalobacter"
## [1] ""
## [1] "-------------- TESTING ------------"
## [1] "56 ASV01621 Genus Aggregatibacter"
## [1] ""
## [1] "-------------- TESTING ------------"
## [1] "57 ASV00068 Genus Haemophilus"
## [1] ""
## [1] "-------------- TESTING ------------"
## [1] "58 ASV01668 Genus Actinobacillus"
## [1] ""
## [1] "-------------- TESTING ------------"
## [1] "59 ASV00769 Genus Enterobacter"
## [1] ""
## [1] "-------------- TESTING ------------"
## [1] "60 ASV00727 Genus Klebsiella"
## [1] ""
## [1] "-------------- TESTING ------------"
## [1] "61 ASV00511 Genus Citrobacter"
## [1] ""
## [1] "-------------- TESTING ------------"
## [1] "62 ASV00041 Genus Escherichia-Shigella"
## p_val_gee_dientamoeba_positive 0.00036
## p_val_gee_blastocystis_positive 0.00125
## p_val_gee_blasto_dient_pos 0.000462
```

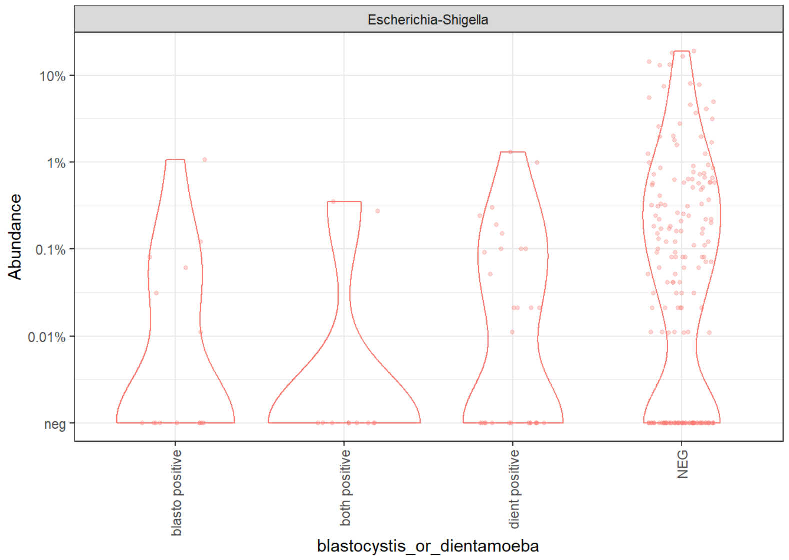


```
## [1] ""
## [1] "-------------- TESTING ------------"
## [1] "63 ASV01371 Genus TM7x"
## [1] ""
## [1] "-------------- TESTING ------------"
## [1] "64 ASV01546 Genus Actinomyces"
## [1] ""
## [1] "-------------- TESTING ------------"
## [1] "65 ASV00094 Genus Bifidobacterium"
## [1] ""
## [1] "-------------- TESTING ------------"
## [1] "66 ASV01145 Genus Eggerthella"
## [1] ""
## [1] "-------------- TESTING ------------"
## [1] "67 ASV01898 Genus Senegalimassilia"
## [1] ""
## [1] "-------------- TESTING ------------"
## [1] "68 ASV01210 Genus Slackia"
## [1] ""
## [1] "-------------- TESTING ------------"
```

```
## [1] "69 ASV00283 Genus Collinsella"
## p_val_gee_dientamoeba_positive 0.000802
## p_val_gee_blastocystis_positive 0.851
## p_val_gee_blasto_dient_pos 0.262
```

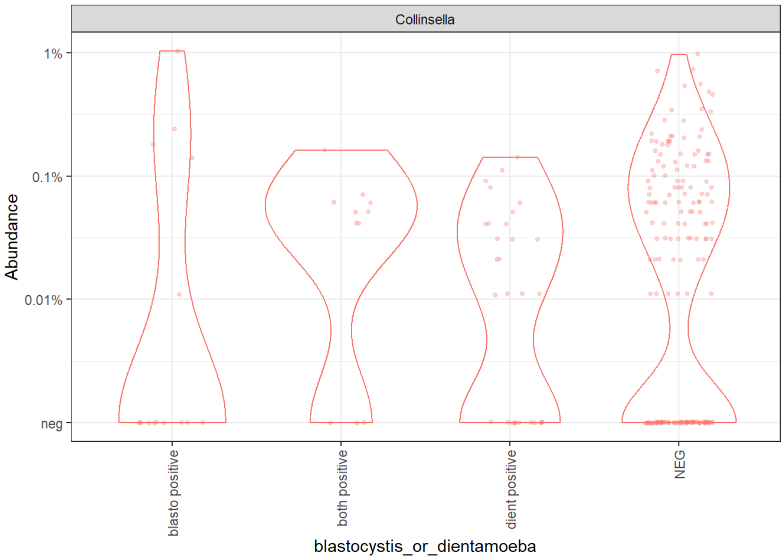


```
## [1] ""
## [1] "-------------- TESTING ------------"
## [1] "70 ASV00024 Genus Akkermansia"
## p_val_gee_dientamoeba_positive 0.0322
## p_val_gee_blastocystis_positive 9.36e-06
## p_val_gee_blasto_dient_pos 0.00245
```

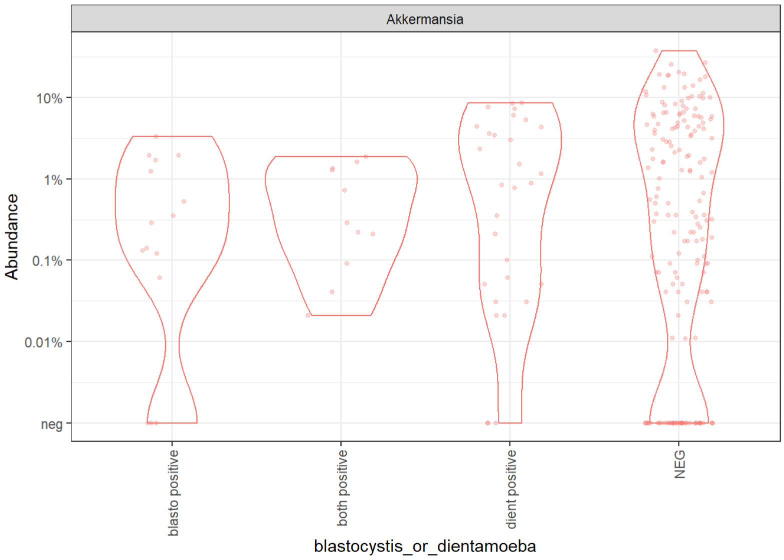


```
## [1] ""
## [1] "-------------- TESTING ------------"
## [1] "71 ASV00614 Genus Bilophila"
## [1] ""
## [1] "-------------- TESTING ------------"
## [1] "72 ASV00873 Genus Desulfovibrio"
## [1] ""
## [1] "-------------- TESTING ------------"
## [1] "73 ASV00371 Genus Holdemania"
## [1] ""
## [1] "-------------- TESTING ------------"
## [1] "74 ASV00788 Genus Dielma"
## [1] ""
## [1] "-------------- TESTING ------------"
## [1] "75 ASV01383 Genus Faecalitalea"
## p_val_gee_dientamoeba_positive 0.000779
## p_val_gee_blastocystis_positive 0.891
## p_val_gee_blasto_dient_pos 0.143
```

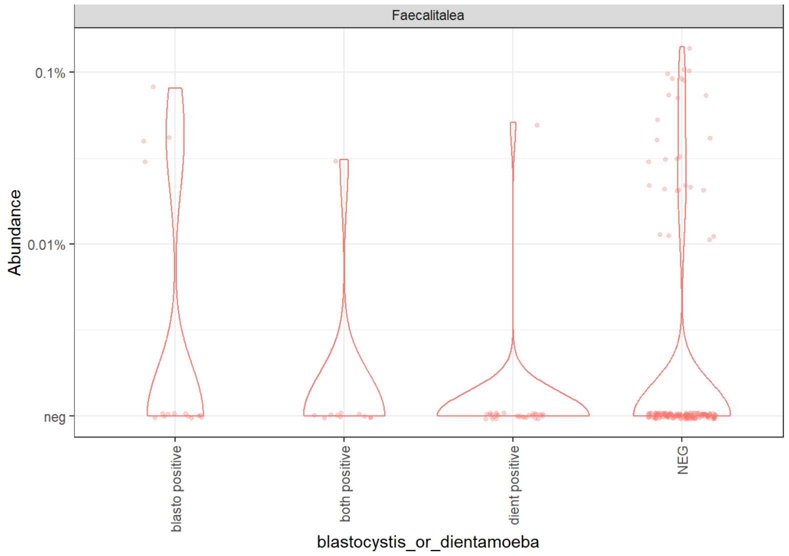


```
## [1] ""
## [1] "-------------- TESTING ------------"
## [1] "76 ASV00885 Genus [Clostridium] innocuum group"
## [1] ""
## [1] "-------------- TESTING ------------"
## [1] "77 ASV00301 Genus Holdemanella"
## [1] ""
## [1] "-------------- TESTING ------------"
## [1] "78 ASV00113 Genus Erysipelotrichaceae UCG-003"
## [1] ""
## [1] "-------------- TESTING ------------"
## [1] "79 ASV00880 Genus Coprobacillus"
## [1] ""
## [1] "-------------- TESTING ------------"
## [1] "80 ASV00473 Genus Erysipelatoclostridium"
## [1] ""
## [1] "-------------- TESTING ------------"
## [1] "81 ASV00526 Genus Catenibacterium"
## [1] ""
## [1] "-------------- TESTING ------------"
## [1] "82 ASV00079 Genus Turicibacter"
## [1] ""
## [1] "-------------- TESTING ------------"
## [1] "83 ASV00981 Genus Lactococcus"
## [1] ""
## [1] "-------------- TESTING ------------"
## [1] "84 ASV00185 Genus Streptococcus"
## [1] ""
## [1] "-------------- TESTING ------------"
## [1] "85 ASV00796 Genus Lactiplantibacillus"
## [1] ""
## [1] "-------------- TESTING ------------"
## [1] "86 ASV00855 Genus Lacticaseibacillus"
## [1] ""
## [1] "-------------- TESTING ------------"
## [1] "87 ASV01112 Genus Lactobacillus"
## [1] ""
## [1] "-------------- TESTING ------------"
## [1] "88 ASV00583 Genus Granulicatella"
## p_val_gee_dientamoeba_positive 0.000132
## p_val_gee_blastocystis_positive 0.0035
## p_val_gee_blasto_dient_pos 0.000475
```

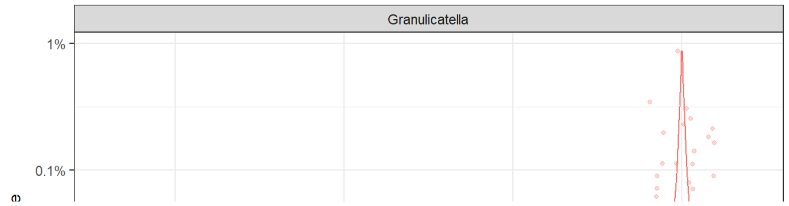

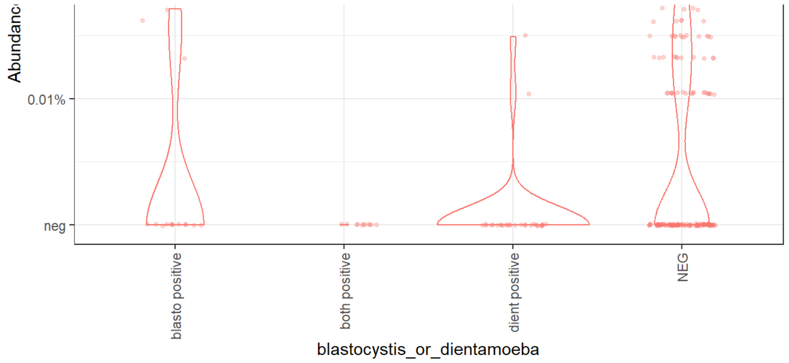


```
## [1] ""
## [1] "-------------- TESTING ------------"
## [1] "89 ASV01756 Genus Abiotrophia"
## [1] ""
## [1] "-------------- TESTING ------------"
## [1] "90 ASV01883 Genus Gemella"
## [1] ""
## [1] "-------------- TESTING ------------"
## [1] "91 ASV00115 Genus Veillonella"
## [1] ""
## [1] "-------------- TESTING ------------"
## [1] "92 ASV00004 Genus Dialister"
## [1] ""
## [1] "-------------- TESTING ------------"
## [1] "93 ASV01509 Genus Allisonella"
## [1] ""
## [1] "-------------- TESTING ------------"
## [1] "94 ASV00893 Genus Megasphaera"
## [1] ""
## [1] "-------------- TESTING ------------"
## [1] "95 ASV00744 Genus Megamonas"
## [1] ""
## [1] "-------------- TESTING ------------"
## [1] "96 ASV01065 Genus Acidaminococcus"
## [1] ""
## [1] "-------------- TESTING ------------"
## [1] "97 ASV00081 Genus Phascolarctobacterium"
## [1] ""
## [1] "-------------- TESTING ------------"
## [1] "98 ASV00197 Genus Succiniclasticum"
## [1] ""
## [1] "-------------- TESTING ------------"
## [1] "99 ASV02313 Genus Peptococcus"
## [1] ""
## [1] "-------------- TESTING ------------"
## [1] "100 ASV02228 Genus Anaerofustis"
## [1] ""
## [1] "-------------- TESTING ------------"
## [1] "101 ASV01542 Genus Ezakiella"
## [1] ""
## [1] "-------------- TESTING ------------"
## [1] "102 ASV01197 Genus Fenollaria"
## [1] ""
## [1] "-------------- TESTING ------------"
## [1] "103 ASV00503 Genus Family XIII UCG-001"
## [1] ""
## [1] "-------------- TESTING ------------"
## [1] "104 ASV00350 Genus Family XIII AD3011 group"
## [1] ""
## [1] "-------------- TESTING ------------"
## [1] "105 ASV00080 Genus Intestinibacter"
## [1] ""
## [1] "-------------- TESTING ------------"
## [1] "106 ASV00036 Genus Romboutsia"
## p_val_gee_dientamoeba_positive 0.00247
```

```
## p_val_gee_blastocystis_positive 0.000207
## p_val_gee_blasto_dient_pos 0.000398
```

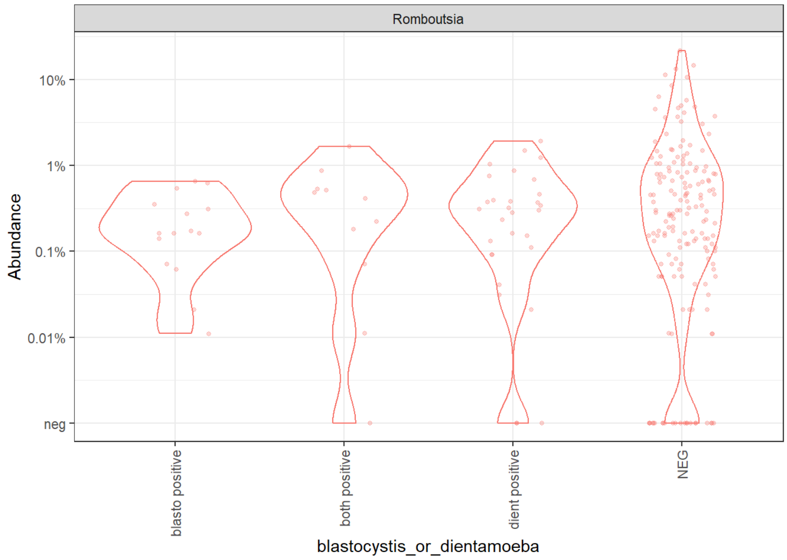


```
## [1] ""
## [1] "-------------- TESTING ------------"
## [1] "107 ASV00194 Genus Terrisporobacter"
## [1] ""
## [1] "-------------- TESTING ------------"
## [1] "108 ASV01662 Genus Catabacter"
## [1] ""
## [1] "-------------- TESTING ------------"
## [1] "109 ASV01352 Genus Christensenella"
## [1] ""
## [1] "-------------- TESTING ------------"
## [1] "110 ASV00053 Genus Christensenellaceae R-7 group"
## [1] ""
## [1] "-------------- TESTING ------------"
## [1] "111 ASV00116 Genus Monoglobus"
## [1] ""
## [1] "-------------- TESTING ------------"
## [1] "112 ASV00060 Genus UCG-005"
## [1] ""
## [1] "-------------- TESTING ------------"
## [1] "113 ASV00043 Genus UCG-002"
## p_val_gee_dientamoeba_positive 0.00148
## p_val_gee_blastocystis_positive 0.179
## p_val_gee_blasto_dient_pos 0.000663
```

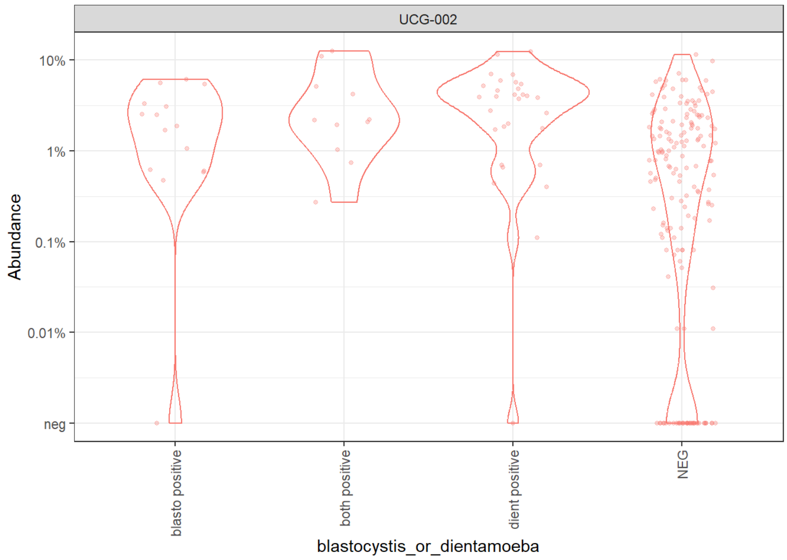


```
## [1] ""
## [1] "-------------- TESTING ------------"
```

```
## [1] "114 ASV00147 Genus UCG-003"
## [1] ""
## [1] "-------------- TESTING ------------"
## [1] "115 ASV01190 Genus Oscillospira"
## [1] ""
## [1] "-------------- TESTING ------------"
## [1] "116 ASV00125 Genus Oscillibacter"
## [1] ""
## [1] "-------------- TESTING ------------"
## [1] "117 ASV00468 Genus Colidextribacter"
## [1] ""
## [1] "-------------- TESTING ------------"
## [1] "118 ASV00231 Genus Flavonifractor"
## p_val_gee_dientamoeba_positive 3.46e-07
## p_val_gee_blastocystis_positive 0.0889
## p_val_gee_blasto_dient_pos 8.51e-05
```

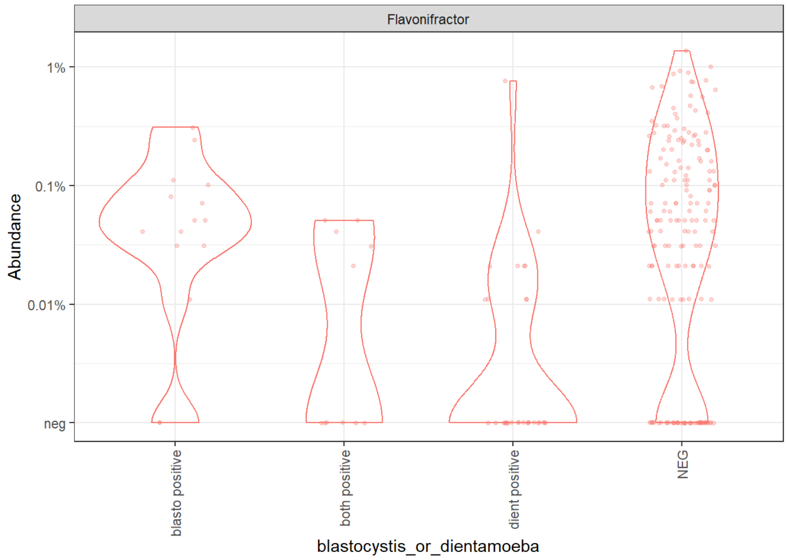


```
## [1] ""
## [1] "-------------- TESTING ------------"
## [1] "119 ASV01629 Genus Pseudoflavonifractor"
## [1] ""
## [1] "-------------- TESTING ------------"
## [1] "120 ASV00496 Genus Intestinimonas"
## [1] ""
## [1] "-------------- TESTING ------------"
## [1] "121 ASV00222 Genus NK4A214 group"
## p_val_gee_dientamoeba_positive 0.000964
## p_val_gee_blastocystis_positive 0.718
## p_val_gee_blasto_dient_pos 0.00406
```

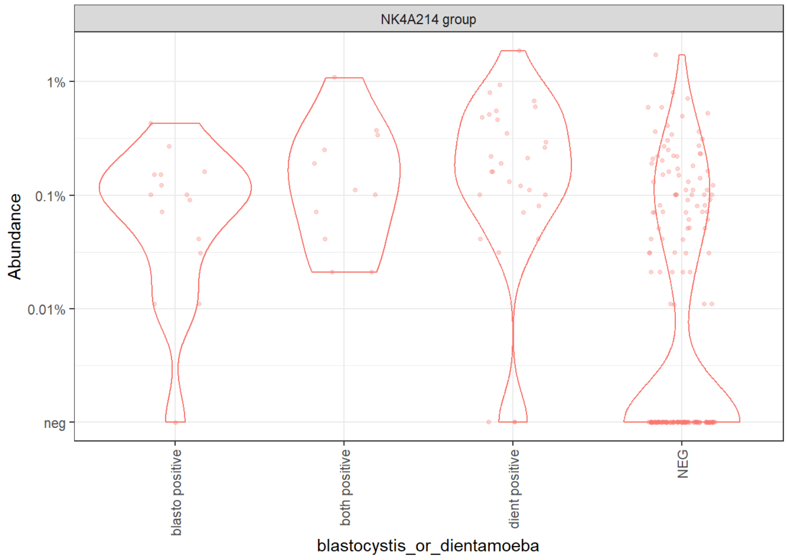

```
## [1] ""
## [1] "-------------- TESTING ------------"
## [1] "122 ASV00151 Genus Incertae Sedis"
## [1] ""
## [1] "-------------- TESTING ------------"
## [1] "123 ASV00524 Genus DTU089"
## [1] ""
## [1] "-------------- TESTING ------------"
## [1] "124 ASV00123 Genus Ruminococcus"
## [1] ""
## [1] "-------------- TESTING ------------"
## [1] "125 ASV00052 Genus CAG-352"
## [1] ""
## [1] "-------------- TESTING ------------"
## [1] "126 ASV00876 Genus Paludicola"
## [1] ""
## [1] "-------------- TESTING ------------"
## [1] "127 ASV00006 Genus Subdoligranulum"
## [1] ""
## [1] "-------------- TESTING ------------"
## [1] "128 ASV00148 Genus UBA1819"
## p_val_gee_dientamoeba_positive 6.32e-05
## p_val_gee_blastocystis_positive 2.04e-05
## p_val_gee_blasto_dient_pos 2.12e-05
```

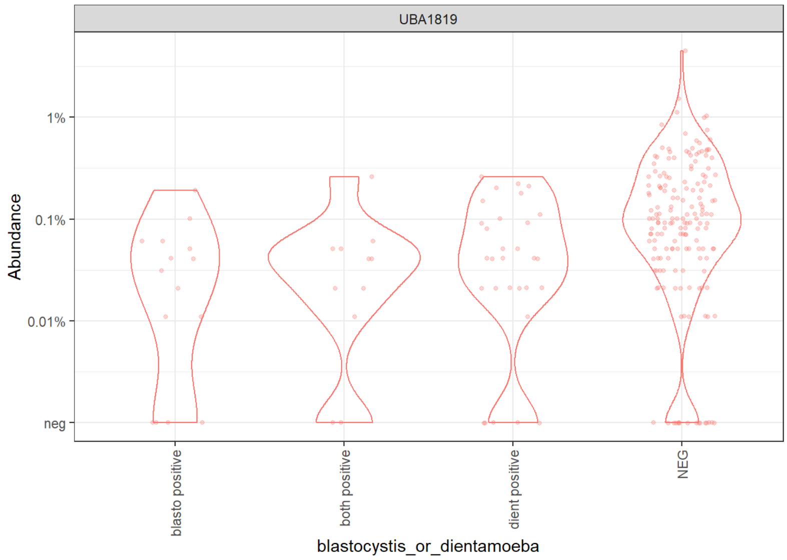


```
## [1] ""
## [1] "-------------- TESTING ------------"
## [1] "129 ASV01984 Genus Fournierella"
## [1] ""
## [1] "-------------- TESTING ------------"
## [1] "130 ASV01591 Genus Anaerofilum"
## [1] ""
## [1] "-------------- TESTING ------------"
## [1] "131 ASV00001 Genus Faecalibacterium"
## [1] ""
## [1] "-------------- TESTING ------------"
## [1] "132 ASV01513 Genus Candidatus Soleaferrea"
## [1] ""
## [1] "-------------- TESTING ------------"
## [1] "133 ASV02446 Genus Negativibacillus"
## [1] ""
## [1] "-------------- TESTING ------------"
## [1] "134 ASV00866 Genus Anaerotruncus"
## [1] ""
## [1] "-------------- TESTING ------------"
## [1] "135 ASV00211 Genus [Eubacterium] siraeum group"
## [1] ""
## [1] "-------------- TESTING ------------"
## [1] "136 ASV00268 Genus Sarcina"
```

```
## [1] ""
## [1] "-------------- TESTING ------------"
## [1] "137 ASV00072 Genus Clostridium sensu stricto 1"
## [1] ""
## [1] "-------------- TESTING ------------"
## [1] "138 ASV00618 Genus Tyzzerella"
## [1] ""
## [1] "-------------- TESTING ------------"
## [1] "139 ASV01527 Genus GCA-900066755"
```

```
res_parasites_adj <- res_parasites %>%
    group_by(taxrank_name) %>%
    mutate(
        tested_entities = n(),
        padj_gee_blasto_dient_pos = p.adjust(p_val_gee_blasto_dient_pos, method = "bonferroni"),
        padj_gee_blasto_pos = p.adjust(p_val_gee_blastocystis_positive, method = "bonferroni"),
        padj_gee_dient_pos = p.adjust(p_val_gee_dientamoeba_positive, method = "bonferroni"),
        )

my_ts <- strftime(Sys.time(), "%Y%m%dT%H%M%S")
xlsx::write.xlsx(data.frame(res_parasites_adj),
                 file = file.path("./graphs_and_outputs",
                                  paste0("results_gee_of_all_levels_against_parasites", my_ts, ".xlsx")),
                 row.names = F)



write_rds(
    x = res_parasites_adj,
    file = file.path("./saved_data", paste0("results_gee_LINEAR_RAREFIED_against_parasites", my_ts, ".rds"))
)
```

This is yet another graph showing those that are significant

```
pa2 <- function(physeq, x_cat, title = "", Facet = "Genus", Color = "Phylum", my_selection_tax_level = "Genus", my_taxa =
character(0)){

    #CALL: plot_abundance(PH, x_cat = "group",Facet = "Phylum" )

    #This makes one point per sample - tax gloms it...

    physeq <- tax_glom(physeq, taxrank = Facet) #this was missing from the original
    p1f <- transform_sample_counts(physeq, function(x){x / sum(x)})
    mphyseq = psmelt(p1f)
    #phyloseq has the Abundance variable (!)
    mphyseq <- mphyseq[ which(mphyseq[, my_selection_tax_level] %in% my_taxa), ]
    mphyseq <- subset(mphyseq, Abundance > 0)
    ggplot(data = mphyseq, mapping = aes_string(x = x_cat, y = "Abundance",
                                                color = Color, fill = Color)) +
        geom_violin(fill = NA) +
        geom_point(size = 1, alpha = 0.3,
                   position = position_jitter(width = 0.3)) +
        facet_wrap(facets = Facet) +
        scale_y_log10(
            breaks = c(1e-4,1e-3,1e-2,0.1,1),
            labels = c("0.01%", "0.1%", "1%", "10%", "100%")
            )+
        theme(axis.text.x = element_text(angle = 90, vjust = 0.5, hjust=1))+
        theme(legend.position="none")
}

pa2(PSMR_G, Facet  = "Genus", x_cat = "blastocystis_or_dientamoeba", my_selection_tax_level = "Genus", my_taxa = c("Akkermansia",
"UBA1819", "Blautia", "Flavonifractor", "Anaerostipes", "Granulicatella"), title  = "Significant - normalized to total, linear GEE") +
labs(x = "Category of sample by parasite findings")
```

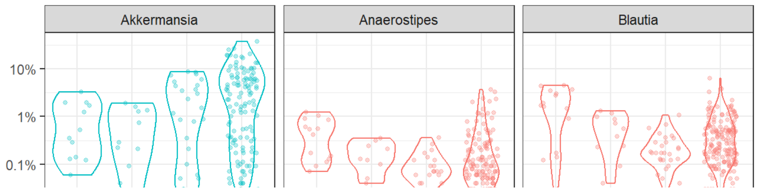

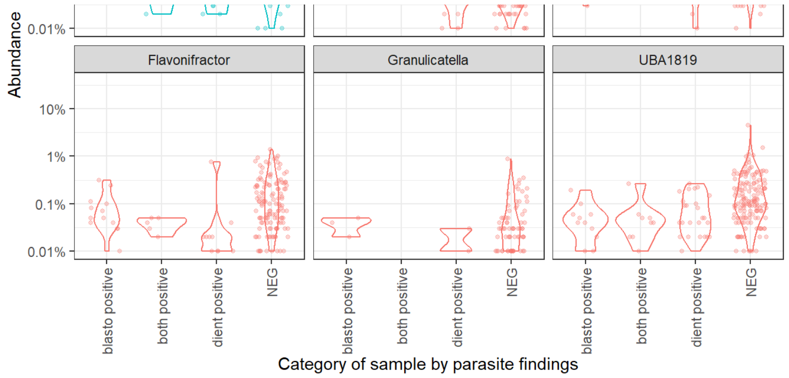

save-load point 3

```
save.image(file = "./saved_data/cipp_intermediate03.RData")
#load(file = "./saved_data/cipp_intermediate03.RData")
```

Taxa tested against parasite positivity - this time analyses are done using the CLR-transformed dataset

This is an additional unplanned analysis requested by added into the revision

```
###################################################################
# Testing mixed effect model + gee with bacteria as outcome.
#

#result table
res_parasites <- data.frame()
vr <- 0

my_taxa_levels <- (c("Kingdom", "Phylum", "Class", "Order", "Family", "Genus", "OTU"))

for (tax_level in c(2,3,4,5,6)){

    if (tax_level == 6) {
        PS <- PSMR_G
    } else if (tax_level == 5) {
        PS <- PSMR_F
    } else if (tax_level == 4) {
        PS <- PSMR_O
    } else if (tax_level == 3) {
        PS <- PSMR_C
    } else if (tax_level == 2) {
        PS <- PSMR_P
    } else if (tax_level == 7) {
        PS <- RAR
    } else {
        stop("Nonexistant phylo level")
    }

    taxrank_name <- my_taxa_levels[tax_level]
    total_reads <- max(apply(otu_table(PS), 1, sum))

    otus_for_regression <- merge(x = s_data, y = otu_table(PS), all.x = T, all.y = F, by = "row.names")
    otus_for_regression <- otus_for_regression %>%
        arrange(subject_no, time_123)

    PS_FOR_TRANSFORM <- PS
    otu_table(PS_FOR_TRANSFORM) <- otu_table(PS_FOR_TRANSFORM) + 5
    PS_CLR <- microbiome::transform(PS_FOR_TRANSFORM, "clr")
    # CLR transform applies a pseudocount of min(relative abundance)/2 to exact zero relative abundance entries
    # in OTU table before taking logs...
    # this is exactly what we want, we want to go a bit higher with the pseudocount; that will eliminate false effects of the lowest
quantities.
    taxa_sums(PS_CLR)
    sample_sums(PS_CLR)

    clr_otus_for_regression <- merge(
        x = s_data,
```

```
        y = otu_table(PS_CLR),
        all.x = T,
        all.y = F,
        by = "row.names"
    )
    clr_otus_for_regression <- clr_otus_for_regression %>%
        arrange(subject_no, time_123)



    for (i in 1:nrow(tax_table(PS))){
        vr <- vr + 1

        asv = row.names(tax_table(PS))[i]
        otus_for_regression[, "relative_signal"] = otus_for_regression[, asv] / total_reads
        taxon_name = as.character(tax_table(PS)[i, tax_level])

        print("")
        print("-------------- TESTING ------------")
        print(paste(i, asv, taxrank_name, taxon_name))
        res_parasites[vr, "asv"] <- asv
        res_parasites[vr, "taxrank_name"] <- taxrank_name
        res_parasites[vr, "taxon_name"] <- taxon_name

        #GEE either Blasto or Dientam.
        gee_res<- geepack::geeglm(
            formula = formula(paste( asv, ' ~  blasto_or_dientamoeba_pos +time_23+  on_intervention',
                                     sep = "")),
            id = subject_no,
            data = clr_otus_for_regression,
            family = gaussian,
            corstr = "ar1",
            contrasts = NULL
            )
        summary(gee_res)

        res_parasites[vr, "p_val_gee_blasto_dient_pos"]<- summary(gee_res)$coefficients["blasto_or_dientamoeba_pospositive",
"Pr(>|W|)"]
        res_parasites[vr, "p_val_gee_on_intervention1"]      <- summary(gee_res)$coefficients["on_intervention1", "Pr(>|W|)"]
        res_parasites[vr, "p_val_time_23"]      <- summary(gee_res)$coefficients["time_23", "Pr(>|W|)"]

        res_parasites[vr, "coef_gee_blasto_dient_pos"] <- summary(gee_res)$coefficients["blasto_or_dientamoeba_pospositive",
"Estimate"]
        res_parasites[vr, "coef_gee_on_intervention1"]       <- summary(gee_res)$coefficients["on_intervention1", "Estimate"]
        res_parasites[vr, "coef_gee_time_23"]      <- summary(gee_res)$coefficients["time_23", "Estimate"]


        #GEE blastocystis
        gee_res2<- geepack::geeglm(
            formula = formula(paste( asv, ' ~  blastocystis_posneg + time_23 +  on_intervention',
                                     sep = "")),
            id = subject_no,
            data = clr_otus_for_regression,
            family = gaussian,
            corstr = "ar1",
            contrasts = NULL
            )
        summary(gee_res2)

        res_parasites[vr, "p_val_gee_blastocystis_positive"]<- summary(gee_res2)$coefficients["blastocystis_posnegpositive",
"Pr(>|W|)"]
        res_parasites[vr, "coef_gee_blastocystis_positive"] <- summary(gee_res2)$coefficients["blastocystis_posnegpositive",
"Estimate"]

        #GEE dientamoeba
        gee_res3<- geepack::geeglm(
            formula = formula(paste( asv, ' ~  dientamoeba_posneg + time_23 +  on_intervention',
```

```
                                    sep = "")),
            id = subject_no,
            data = clr_otus_for_regression,
            family = gaussian,
            corstr = "ar1",
            contrasts = NULL
            )
        summary(gee_res3)

        res_parasites[vr, "p_val_gee_dientamoeba_positive"]<- summary(gee_res3)$coefficients["dientamoeba_posnegpositive", "Pr(>|W|)"]
        res_parasites[vr, "coef_gee_dientamoeba_positive"] <- summary(gee_res3)$coefficients["dientamoeba_posnegpositive", "Estimate"]



        if (res_parasites[vr, "p_val_gee_dientamoeba_positive"] < 0.001 |
            res_parasites[vr, "p_val_gee_blastocystis_positive"] < 0.001 |
            res_parasites[vr, "p_val_gee_blasto_dient_pos"] < 0.001)
            {

            px <- pa_graph(PS,
                   Facet  = taxrank_name,
                   my_selection_tax_level = taxrank_name,
                   x_cat = "blastocystis_or_dientamoeba",
                   my_taxa = taxon_name,
                   title = paste(taxrank_name, taxon_name))
            cat("p_val_gee_dientamoeba_positive", res_parasites[vr, "p_val_gee_dientamoeba_positive"], "\n")
            cat("p_val_gee_blastocystis_positive", res_parasites[vr, "p_val_gee_blastocystis_positive"], "\n")
            cat("p_val_gee_blasto_dient_pos", res_parasites[vr, "p_val_gee_blasto_dient_pos"], "\n")
            print(px)
        }
    }
}
```

```
## [1] ""
## [1] "-------------- TESTING ------------"
## [1] "1 ASV00002 Phylum Bacteroidota"
## [1] ""
## [1] "-------------- TESTING ------------"
## [1] "2 ASV01987 Phylum Fusobacteriota"
## [1] ""
## [1] "-------------- TESTING ------------"
## [1] "3 ASV00041 Phylum Proteobacteria"
## [1] ""
## [1] "-------------- TESTING ------------"
## [1] "4 ASV01371 Phylum Patescibacteria"
## [1] ""
## [1] "-------------- TESTING ------------"
## [1] "5 ASV00094 Phylum Actinobacteriota"
## [1] ""
## [1] "-------------- TESTING ------------"
## [1] "6 ASV00734 Phylum Cyanobacteria"
## [1] ""
## [1] "-------------- TESTING ------------"
## [1] "7 ASV00024 Phylum Verrucomicrobiota"
## [1] ""
## [1] "-------------- TESTING ------------"
## [1] "8 ASV00614 Phylum Desulfobacterota"
## [1] ""
## [1] "-------------- TESTING ------------"
## [1] "9 ASV00001 Phylum Firmicutes"
## [1] ""
## [1] "-------------- TESTING ------------"
## [1] "1 ASV00002 Class Bacteroidia"
## [1] ""
## [1] "-------------- TESTING ------------"
## [1] "2 ASV01987 Class Fusobacteriia"
## p_val_gee_dientamoeba_positive 0.602
## p_val_gee_blastocystis_positive 0.000393
```

```
## p_val_gee_blasto_dient_pos 0.0502
```

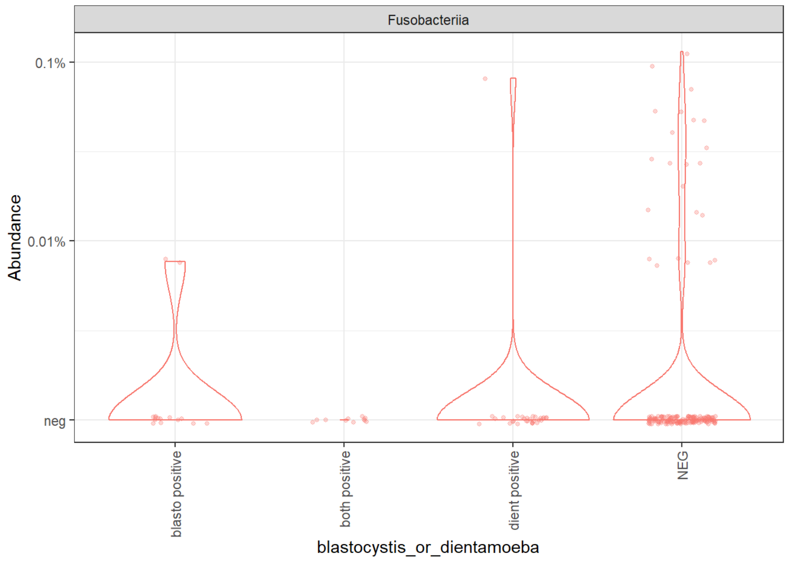


```
## [1] ""
## [1] "-------------- TESTING ------------"
## [1] "3 ASV01698 Class Lentisphaeria"
## [1] ""
## [1] "-------------- TESTING ------------"
## [1] "4 ASV00041 Class Gammaproteobacteria"
## [1] ""
## [1] "-------------- TESTING ------------"
## [1] "5 ASV00557 Class Alphaproteobacteria"
## [1] ""
## [1] "-------------- TESTING ------------"
## [1] "6 ASV01371 Class Saccharimonadia"
## [1] ""
## [1] "-------------- TESTING ------------"
## [1] "7 ASV01337 Class Cyanobacteriia"
## [1] ""
## [1] "-------------- TESTING ------------"
## [1] "8 ASV00094 Class Actinobacteria"
## [1] ""
## [1] "-------------- TESTING ------------"
## [1] "9 ASV00283 Class Coriobacteriia"
## [1] ""
## [1] "-------------- TESTING ------------"
## [1] "10 ASV00734 Class Vampirivibrionia"
## [1] ""
## [1] "-------------- TESTING ------------"
## [1] "11 ASV00024 Class Verrucomicrobiae"
## [1] ""
## [1] "-------------- TESTING ------------"
## [1] "12 ASV00614 Class Desulfovibrionia"
## [1] ""
## [1] "-------------- TESTING ------------"
## [1] "13 ASV00079 Class Bacilli"
## [1] ""
## [1] "-------------- TESTING ------------"
## [1] "14 ASV00004 Class Negativicutes"
## [1] ""
## [1] "-------------- TESTING ------------"
## [1] "15 ASV01456 Class Incertae Sedis"
## [1] ""
## [1] "-------------- TESTING ------------"
## [1] "16 ASV00001 Class Clostridia"
## [1] ""
## [1] "-------------- TESTING ------------"
## [1] "1 ASV00012 Order Lachnospirales"
## [1] ""
## [1] "-------------- TESTING ------------"
```

```
## [1] "2 ASV01487 Order Flavobacteriales"
## p_val_gee_dientamoeba_positive 0.559
## p_val_gee_blastocystis_positive 0.000455
## p_val_gee_blasto_dient_pos 0.893
```

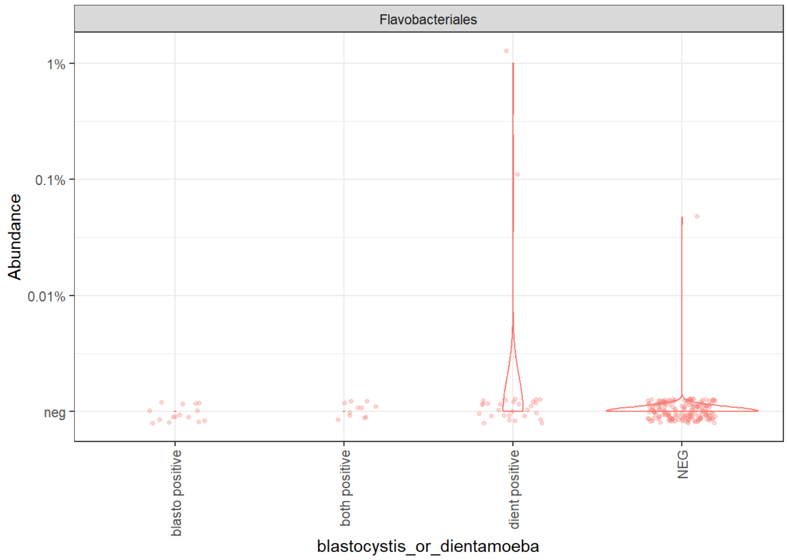


```
## [1] ""
## [1] "-------------- TESTING ------------"
## [1] "3 ASV00002 Order Bacteroidales"
## [1] ""
## [1] "-------------- TESTING ------------"
## [1] "4 ASV01987 Order Fusobacteriales"
## p_val_gee_dientamoeba_positive 0.00759
## p_val_gee_blastocystis_positive 2.73e-06
## p_val_gee_blasto_dient_pos 0.00011
```

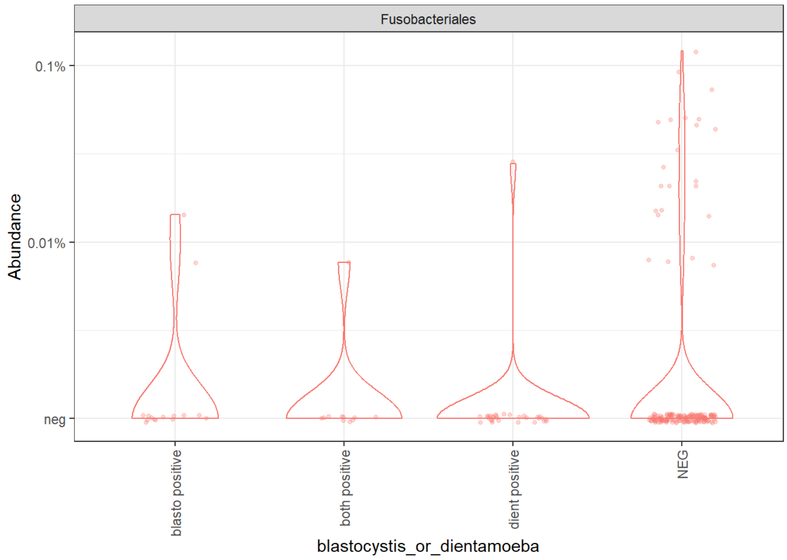


```
## [1] ""
## [1] "-------------- TESTING ------------"
## [1] "5 ASV01698 Order Victivallales"
## [1] ""
## [1] "-------------- TESTING ------------"
## [1] "6 ASV00214 Order Burkholderiales"
## [1] ""
## [1] "-------------- TESTING ------------"
## [1] "7 ASV00041 Order Enterobacterales"
## [1] ""
## [1] "-------------- TESTING ------------"
## [1] "8 ASV00557 Order Rhodospirillales"
## [1] ""
## [1] "-------------- TESTING ------------"
## [1] "9 ASV01371 Order Saccharimonadales"
```

```
## [1] ""
## [1] "-------------- TESTING ------------"
## [1] "10 ASV01337 Order Chloroplast"
## p_val_gee_dientamoeba_positive 0.011
## p_val_gee_blastocystis_positive 8.58e-05
## p_val_gee_blasto_dient_pos 0.000183
```

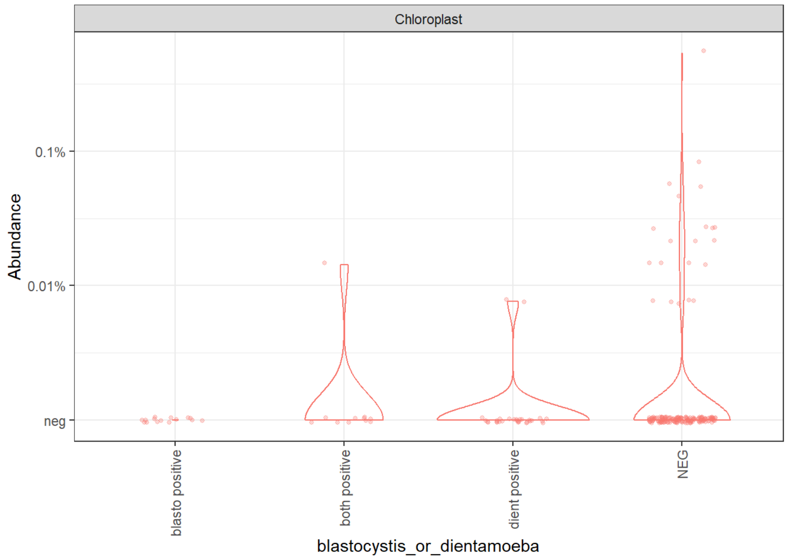


```
## [1] ""
## [1] "-------------- TESTING ------------"
## [1] "11 ASV01546 Order Actinomycetales"
## p_val_gee_dientamoeba_positive 0.00141
## p_val_gee_blastocystis_positive 0.000269
## p_val_gee_blasto_dient_pos 1.22e-05
```

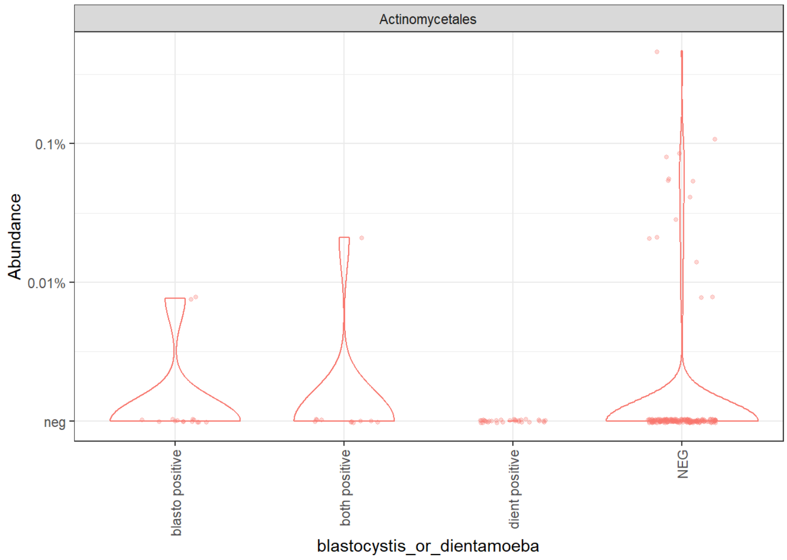


```
## [1] ""
## [1] "-------------- TESTING ------------"
## [1] "12 ASV00094 Order Bifidobacteriales"
## [1] ""
## [1] "-------------- TESTING ------------"
## [1] "13 ASV00283 Order Coriobacteriales"
## [1] ""
## [1] "-------------- TESTING ------------"
## [1] "14 ASV00734 Order Gastranaerophilales"
## [1] ""
## [1] "-------------- TESTING ------------"
## [1] "15 ASV00024 Order Verrucomicrobiales"
## [1] ""
## [1] "-------------- TESTING ------------"
## [1] "16 ASV00614 Order Desulfovibrionales"
## [1] ""
```

```
## [1] "-------------- TESTING ------------"
## [1] "17 ASV01322 Order Izemoplasmatales"
## [1] ""
## [1] "-------------- TESTING ------------"
## [1] "18 ASV00717 Order RF39"
## [1] ""
## [1] "-------------- TESTING ------------"
## [1] "19 ASV00079 Order Erysipelotrichales"
## [1] ""
## [1] "-------------- TESTING ------------"
## [1] "20 ASV00185 Order Lactobacillales"
## [1] ""
## [1] "-------------- TESTING ------------"
## [1] "21 ASV01883 Order Staphylococcales"
## p_val_gee_dientamoeba_positive 0.000709
## p_val_gee_blastocystis_positive 0.000518
## p_val_gee_blasto_dient_pos 2.09e-05
```

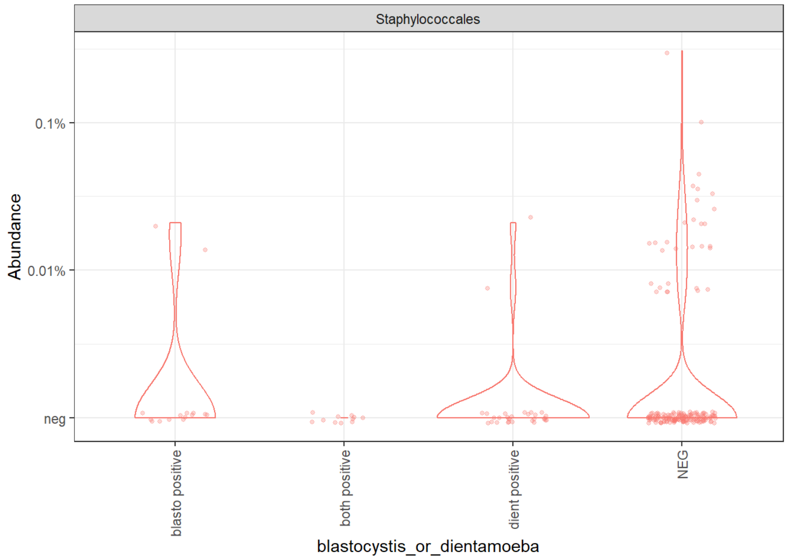


```
## [1] ""
## [1] "-------------- TESTING ------------"
## [1] "22 ASV00004 Order Veillonellales-Selenomonadales"
## [1] ""
## [1] "-------------- TESTING ------------"
## [1] "23 ASV00081 Order Acidaminococcales"
## [1] ""
## [1] "-------------- TESTING ------------"
## [1] "24 ASV00982 Order Peptococcales"
## [1] ""
## [1] "-------------- TESTING ------------"
## [1] "25 ASV02228 Order Eubacteriales"
## p_val_gee_dientamoeba_positive 0.000487
## p_val_gee_blastocystis_positive 2.74e-07
## p_val_gee_blasto_dient_pos 9.67e-07
```

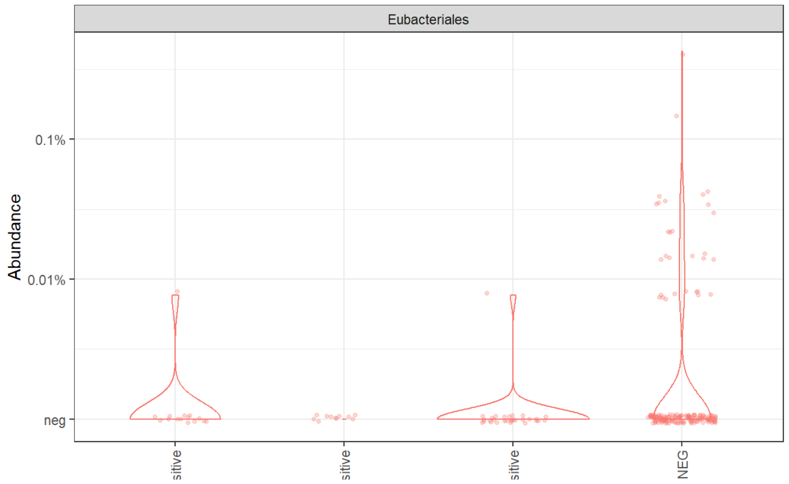

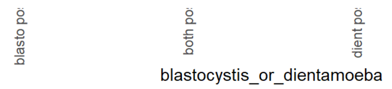


```
## [1] ""
## [1] "-------------- TESTING ------------"
## [1] "26 ASV00036 Order Peptostreptococcales-Tissierellales"
## [1] ""
## [1] "-------------- TESTING ------------"
## [1] "27 ASV01456 Order DTU014"
## [1] ""
## [1] "-------------- TESTING ------------"
## [1] "28 ASV01140 Order Clostridia vadinBB60 group"
## [1] ""
## [1] "-------------- TESTING ------------"
## [1] "29 ASV00053 Order Christensenellales"
## [1] ""
## [1] "-------------- TESTING ------------"
## [1] "30 ASV00116 Order Monoglobales"
## [1] ""
## [1] "-------------- TESTING ------------"
## [1] "31 ASV00001 Order Oscillospirales"
## [1] ""
## [1] "-------------- TESTING ------------"
## [1] "32 ASV00208 Order Clostridia UCG-014"
## [1] ""
## [1] "-------------- TESTING ------------"
## [1] "33 ASV00072 Order Clostridiales"
## [1] ""
## [1] "-------------- TESTING ------------"
## [1] "1 ASV00012 Family Lachnospiraceae"
## [1] ""
## [1] "-------------- TESTING ------------"
## [1] "2 ASV00007 Family Rikenellaceae"
## [1] ""
## [1] "-------------- TESTING ------------"
## [1] "3 ASV01487 Family Flavobacteriaceae"
## [1] ""
## [1] "-------------- TESTING ------------"
## [1] "4 ASV00177 Family Marinifilaceae"
## [1] ""
## [1] "-------------- TESTING ------------"
## [1] "5 ASV01880 Family Dysgonomonadaceae"
## [1] ""
## [1] "-------------- TESTING ------------"
## [1] "6 ASV00059 Family Prevotellaceae"
## [1] ""
## [1] "-------------- TESTING ------------"
## [1] "7 ASV00106 Family Barnesiellaceae"
## p_val_gee_dientamoeba_positive 1.44e-05
## p_val_gee_blastocystis_positive 0.365
## p_val_gee_blasto_dient_pos 0.0399
```

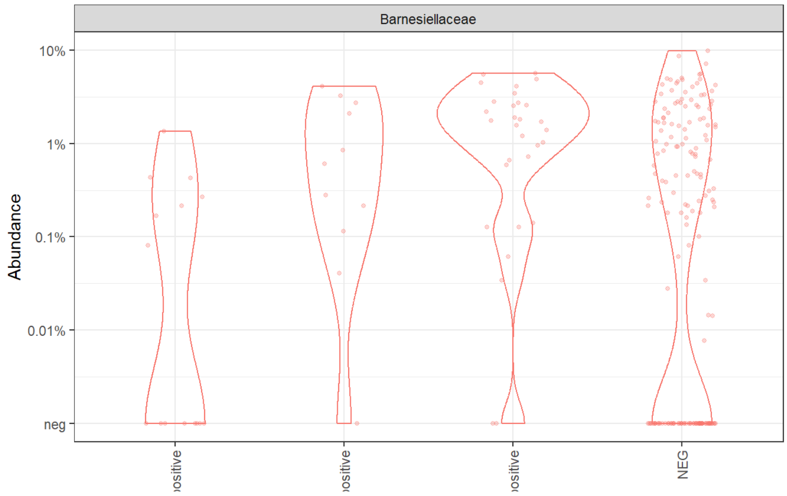

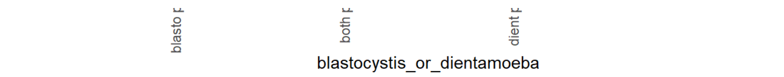


```
## [1] ""
## [1] "-------------- TESTING ------------"
## [1] "8 ASV00460 Family Muribaculaceae"
## [1] ""
## [1] "-------------- TESTING ------------"
## [1] "9 ASV00528 Family Porphyromonadaceae"
## [1] ""
## [1] "-------------- TESTING ------------"
## [1] "10 ASV00002 Family Bacteroidaceae"
## [1] ""
## [1] "-------------- TESTING ------------"
## [1] "11 ASV00020 Family Tannerellaceae"
## [1] ""
## [1] "-------------- TESTING ------------"
## [1] "12 ASV00269 Family Butyricicoccaceae"
## [1] ""
## [1] "-------------- TESTING ------------"
## [1] "13 ASV00044 Family [Eubacterium] coprostanoligenes group"
## p_val_gee_dientamoeba_positive 0.000108
## p_val_gee_blastocystis_positive 0.309
## p_val_gee_blasto_dient_pos 0.0118
```

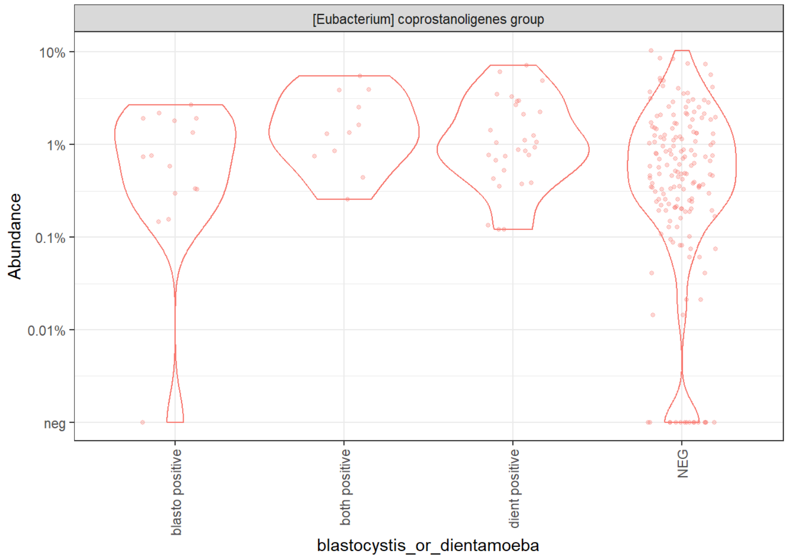


```
## [1] ""
## [1] "-------------- TESTING ------------"
## [1] "14 ASV01407 Family Defluviitaleaceae"
## [1] ""
## [1] "-------------- TESTING ------------"
## [1] "15 ASV01987 Family Fusobacteriaceae"
## p_val_gee_dientamoeba_positive 0.0116
## p_val_gee_blastocystis_positive 1.09e-05
## p_val_gee_blasto_dient_pos 0.000427
```

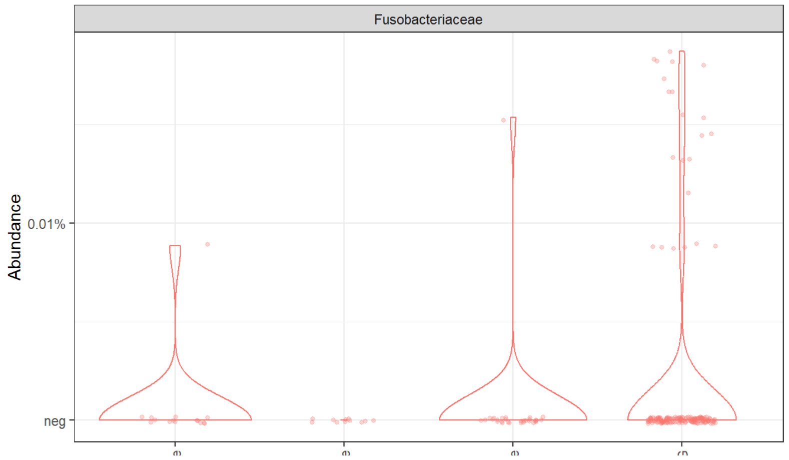

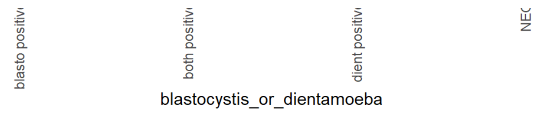


```
## [1] ""
## [1] "-------------- TESTING ------------"
## [1] "16 ASV01698 Family vadinBE97"
## [1] ""
## [1] "-------------- TESTING ------------"
## [1] "17 ASV00214 Family Sutterellaceae"
## [1] ""
## [1] "-------------- TESTING ------------"
## [1] "18 ASV00988 Family Oxalobacteraceae"
## [1] ""
## [1] "-------------- TESTING ------------"
## [1] "19 ASV00068 Family Pasteurellaceae"
## [1] ""
## [1] "-------------- TESTING ------------"
## [1] "20 ASV00041 Family Enterobacteriaceae"
## p_val_gee_dientamoeba_positive 0.000289
## p_val_gee_blastocystis_positive 0.000675
## p_val_gee_blasto_dient_pos 3.43e-05
```

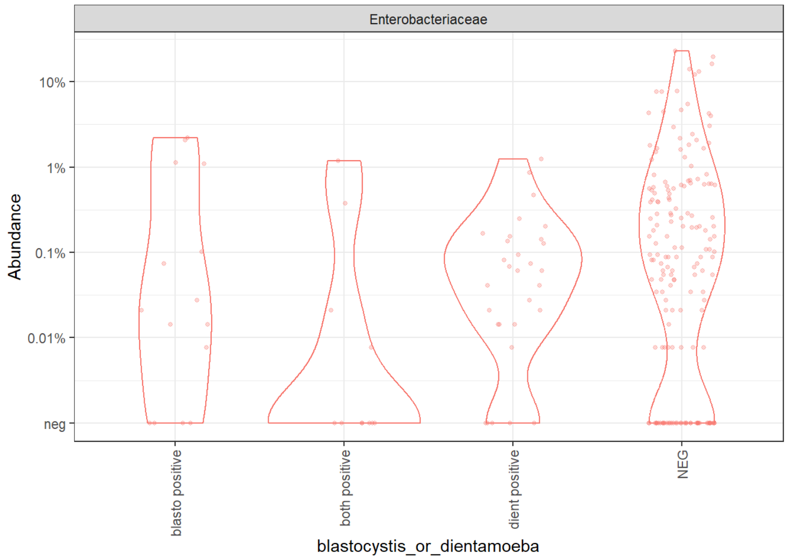


```
## [1] ""
## [1] "-------------- TESTING ------------"
## [1] "21 ASV01371 Family Saccharimonadaceae"
## [1] ""
## [1] "-------------- TESTING ------------"
## [1] "22 ASV01546 Family Actinomycetaceae"
## p_val_gee_dientamoeba_positive 0.000632
## p_val_gee_blastocystis_positive 0.00251
## p_val_gee_blasto_dient_pos 0.000141
```

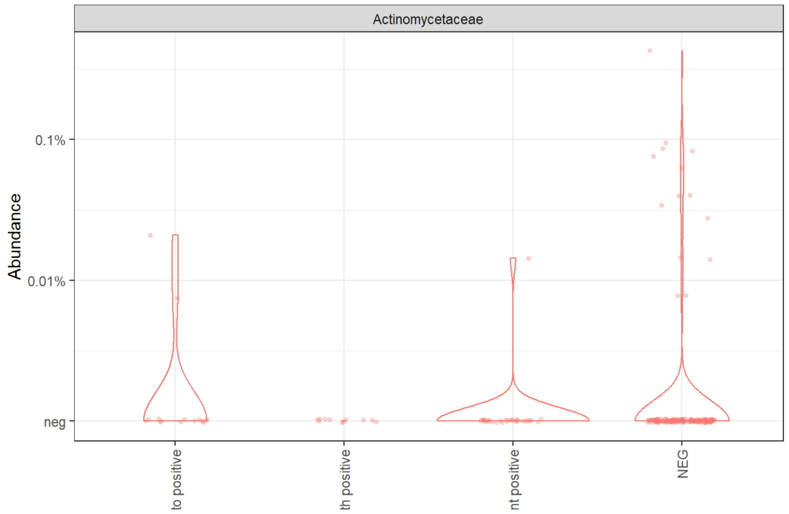

blas bo die
blastocystis_or_dientamoeba

```
## [1] ""
## [1] "-------------- TESTING ------------"
## [1] "23 ASV00094 Family Bifidobacteriaceae"
## [1] ""
## [1] "-------------- TESTING ------------"
## [1] "24 ASV01145 Family Eggerthellaceae"
## [1] ""
## [1] "-------------- TESTING ------------"
## [1] "25 ASV00452 Family Coriobacteriales Incertae Sedis"
## [1] ""
## [1] "-------------- TESTING ------------"
## [1] "26 ASV00283 Family Coriobacteriaceae"
## p_val_gee_dientamoeba_positive 0.000705
## p_val_gee_blastocystis_positive 0.349
## p_val_gee_blasto_dient_pos 0.00388
```

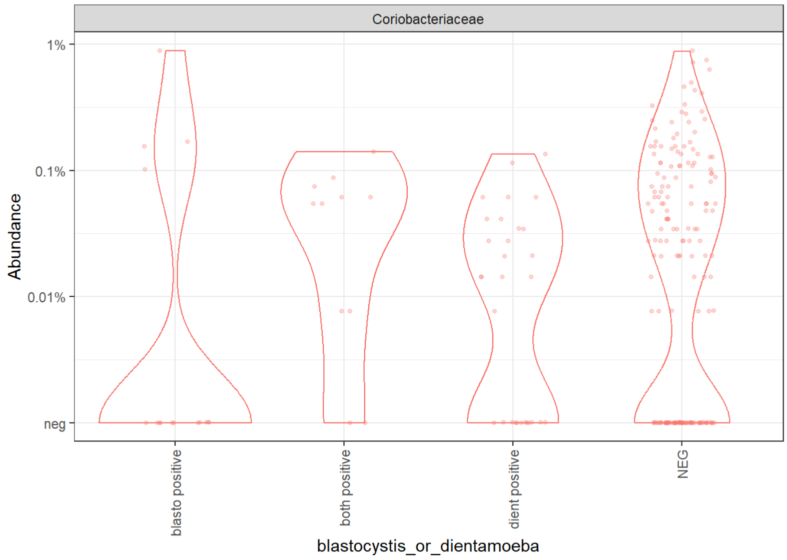


```
## [1] ""
## [1] "-------------- TESTING ------------"
## [1] "27 ASV00024 Family Akkermansiaceae"
## [1] ""
## [1] "-------------- TESTING ------------"
## [1] "28 ASV00614 Family Desulfovibrionaceae"
## [1] ""
## [1] "-------------- TESTING ------------"
## [1] "29 ASV00113 Family Erysipelatoclostridiaceae"
## p_val_gee_dientamoeba_positive 5.14e-05
## p_val_gee_blastocystis_positive 0.346
## p_val_gee_blasto_dient_pos 0.000307
```

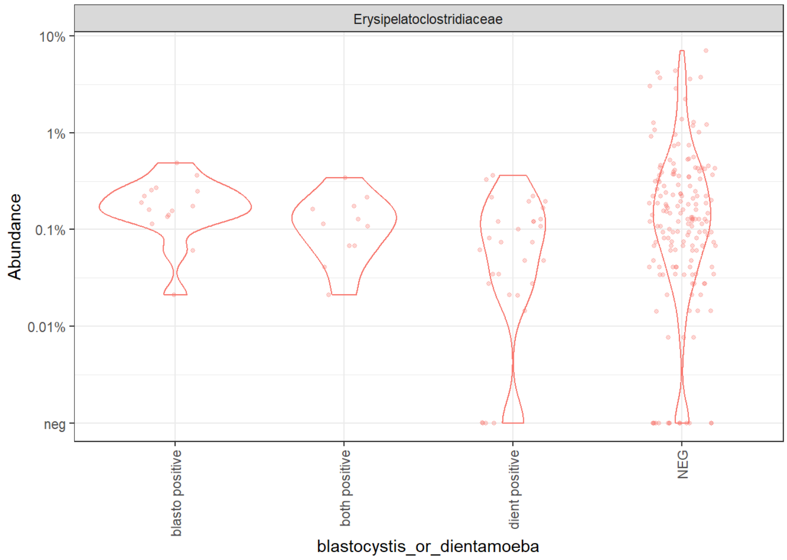

```
## [1] ""
## [1] "-------------- TESTING ------------"
## [1] "30 ASV00079 Family Erysipelotrichaceae"
## [1] ""
## [1] "-------------- TESTING ------------"
## [1] "31 ASV00185 Family Streptococcaceae"
## [1] ""
## [1] "-------------- TESTING ------------"
## [1] "32 ASV00796 Family Lactobacillaceae"
## [1] ""
## [1] "-------------- TESTING ------------"
## [1] "33 ASV00583 Family Carnobacteriaceae"
## p_val_gee_dientamoeba_positive 2.82e-05
## p_val_gee_blastocystis_positive 0.123
## p_val_gee_blasto_dient_pos 0.00086
```

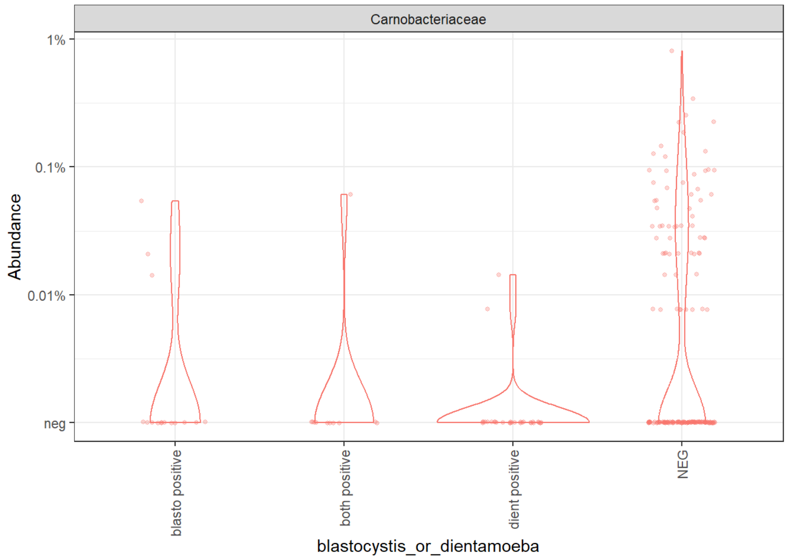


```
## [1] ""
## [1] "-------------- TESTING ------------"
## [1] "34 ASV01756 Family Aerococcaceae"
## [1] ""
## [1] "-------------- TESTING ------------"
## [1] "35 ASV01883 Family Gemellaceae"
## [1] ""
## [1] "-------------- TESTING ------------"
## [1] "36 ASV00004 Family Veillonellaceae"
## [1] ""
## [1] "-------------- TESTING ------------"
## [1] "37 ASV00744 Family Selenomonadaceae"
## [1] ""
## [1] "-------------- TESTING ------------"
## [1] "38 ASV00081 Family Acidaminococcaceae"
## [1] ""
## [1] "-------------- TESTING ------------"
## [1] "39 ASV00982 Family Peptococcaceae"
## [1] ""
## [1] "-------------- TESTING ------------"
## [1] "40 ASV02228 Family Anaerofustaceae"
## p_val_gee_dientamoeba_positive 0.00603
## p_val_gee_blastocystis_positive 0.00139
## p_val_gee_blasto_dient_pos 0.000495
```

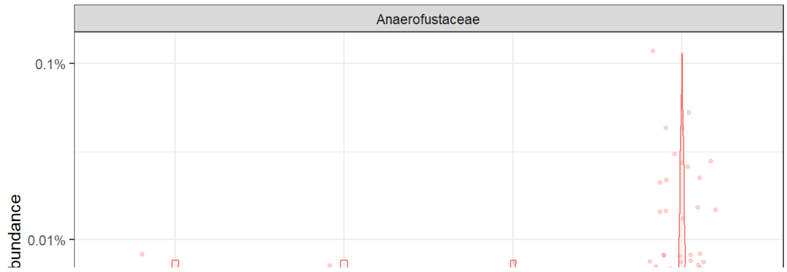

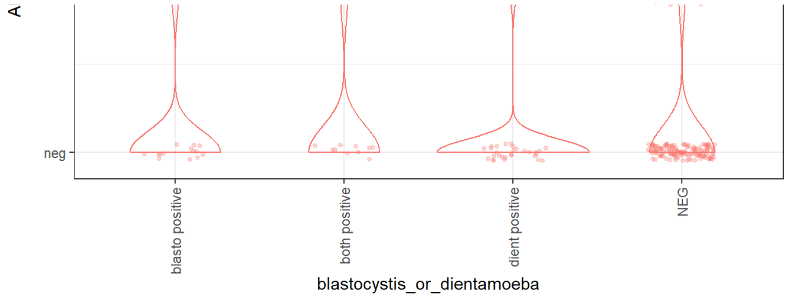


```
## [1] ""
## [1] "-------------- TESTING ------------"
## [1] "41 ASV01197 Family Family XI"
## [1] ""
## [1] "-------------- TESTING ------------"
## [1] "42 ASV00350 Family Anaerovoracaceae"
## [1] ""
## [1] "-------------- TESTING ------------"
## [1] "43 ASV00036 Family Peptostreptococcaceae"
## [1] ""
## [1] "-------------- TESTING ------------"
## [1] "44 ASV00053 Family Christensenellaceae"
## [1] ""
## [1] "-------------- TESTING ------------"
## [1] "45 ASV02142 Family UCG-011"
## [1] ""
## [1] "-------------- TESTING ------------"
## [1] "46 ASV00116 Family Monoglobaceae"
## [1] ""
## [1] "-------------- TESTING ------------"
## [1] "47 ASV00043 Family Oscillospiraceae"
## [1] ""
## [1] "-------------- TESTING ------------"
## [1] "48 ASV00611 Family UCG-010"
## p_val_gee_dientamoeba_positive 2.11e-05
## p_val_gee_blastocystis_positive 0.0672
## p_val_gee_blasto_dient_pos 0.000376
```

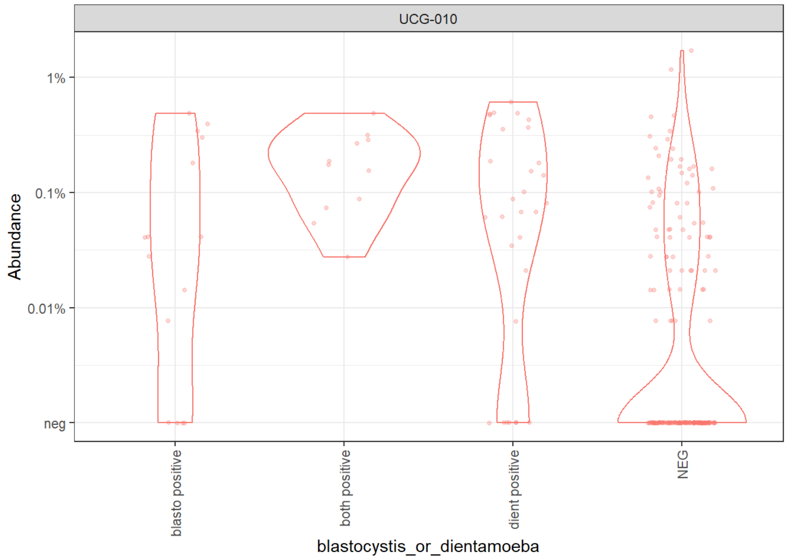


```
## [1] ""
## [1] "-------------- TESTING ------------"
## [1] "49 ASV00001 Family Ruminococcaceae"
## [1] ""
## [1] "-------------- TESTING ------------"
## [1] "50 ASV00072 Family Clostridiaceae"
## [1] ""
## [1] "-------------- TESTING ------------"
## [1] "1 ASV00642 Genus UC5-1-2E3"
## [1] ""
## [1] "-------------- TESTING ------------"
```

```
## [1] "2 ASV00295 Genus Lachnospiraceae UCG-004"
## [1] ""
## [1] "-------------- TESTING ------------"
## [1] "3 ASV00166 Genus Fusicatenibacter"
## p_val_gee_dientamoeba_positive 0.581
## p_val_gee_blastocystis_positive 0.000123
## p_val_gee_blasto_dient_pos 0.0523
```

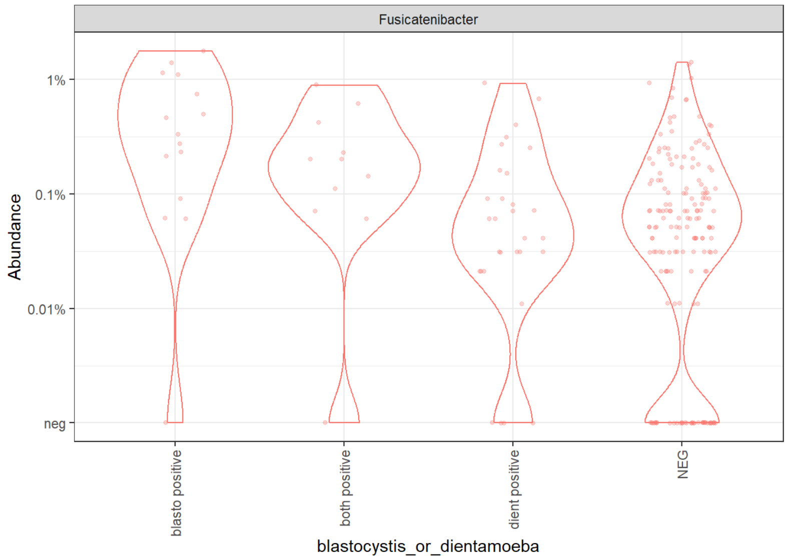


```
## [1] ""
## [1] "-------------- TESTING ------------"
## [1] "4 ASV00135 Genus Eisenbergiella"
## [1] ""
## [1] "-------------- TESTING ------------"
## [1] "5 ASV00047 Genus [Ruminococcus] gnavus group"
## p_val_gee_dientamoeba_positive 1.04e-06
## p_val_gee_blastocystis_positive 0.329
## p_val_gee_blasto_dient_pos 0.0157
```

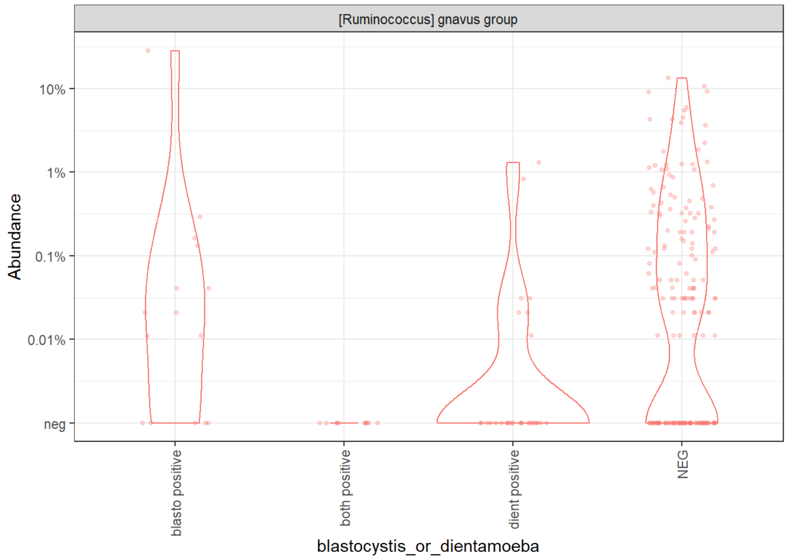


```
## [1] ""
## [1] "-------------- TESTING ------------"
## [1] "6 ASV00070 Genus Dorea"
## [1] ""
## [1] "-------------- TESTING ------------"
## [1] "7 ASV00459 Genus CAG-56"
## p_val_gee_dientamoeba_positive 7.89e-06
## p_val_gee_blastocystis_positive 0.17
## p_val_gee_blasto_dient_pos 5.5e-05
```

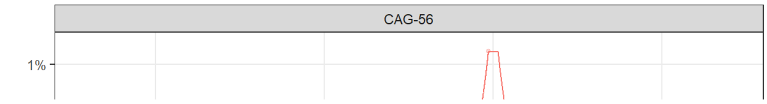

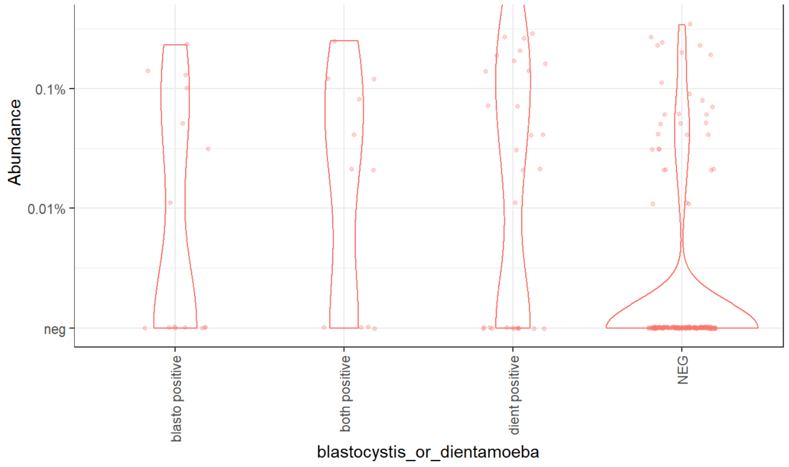


```
## [1] ""
## [1] "-------------- TESTING ------------"
## [1] "8 ASV00559 Genus Lachnospiraceae FCS020 group"
## [1] ""
## [1] "-------------- TESTING ------------"
## [1] "9 ASV01751 Genus Marvinbryantia"
## [1] ""
## [1] "-------------- TESTING ------------"
## [1] "10 ASV00118 Genus [Ruminococcus] torques group"
## p_val_gee_dientamoeba_positive 7.43e-11
## p_val_gee_blastocystis_positive 0.431
## p_val_gee_blasto_dient_pos 0.000151
```

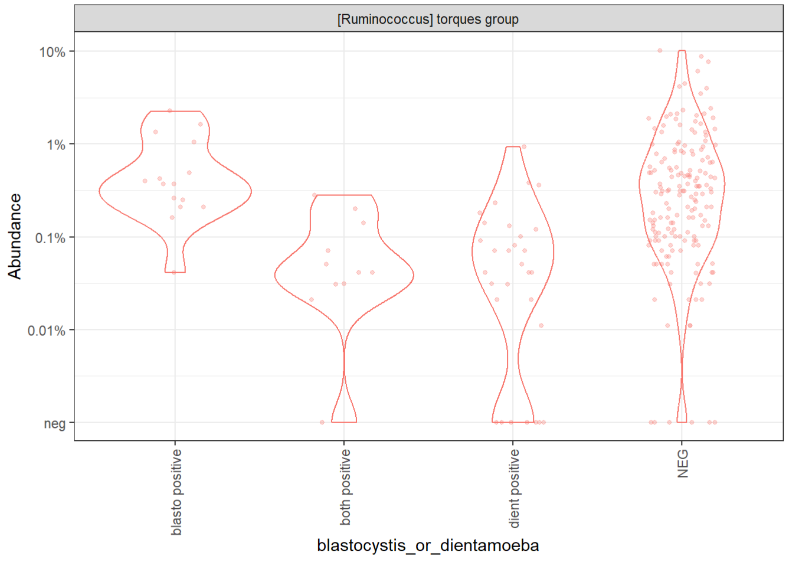


```
## [1] ""
## [1] "-------------- TESTING ------------"
## [1] "11 ASV00754 Genus Sellimonas"
## p_val_gee_dientamoeba_positive 0.000172
## p_val_gee_blastocystis_positive 0.221
## p_val_gee_blasto_dient_pos 0.831
```

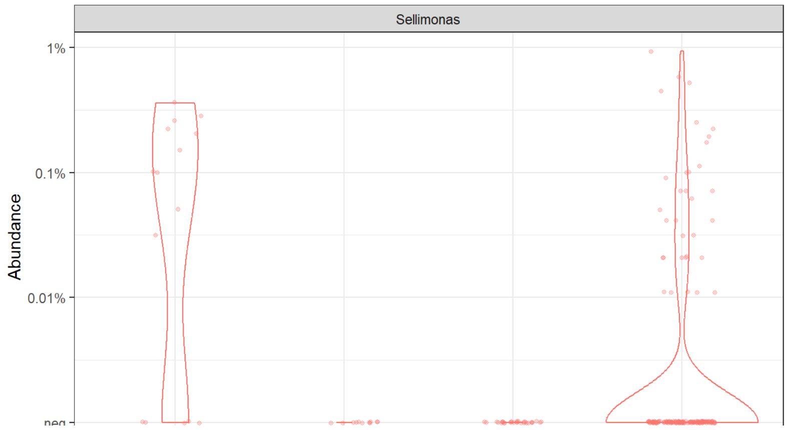

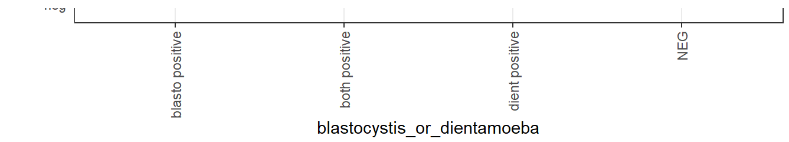


```
## [1] ""
## [1] "-------------- TESTING ------------"
## [1] "12 ASV00398 Genus [Ruminococcus] gauvreauii group"
## [1] ""
## [1] "-------------- TESTING ------------"
## [1] "13 ASV00317 Genus Lachnospiraceae UCG-001"
## [1] ""
## [1] "-------------- TESTING ------------"
## [1] "14 ASV00012 Genus Agathobacter"
## [1] ""
## [1] "-------------- TESTING ------------"
## [1] "15 ASV00057 Genus Roseburia"
## [1] ""
## [1] "-------------- TESTING ------------"
## [1] "16 ASV00975 Genus [Eubacterium] fissicatena group"
## p_val_gee_dientamoeba_positive 0.0103
## p_val_gee_blastocystis_positive 7.46e-06
## p_val_gee_blasto_dient_pos 0.000145
```

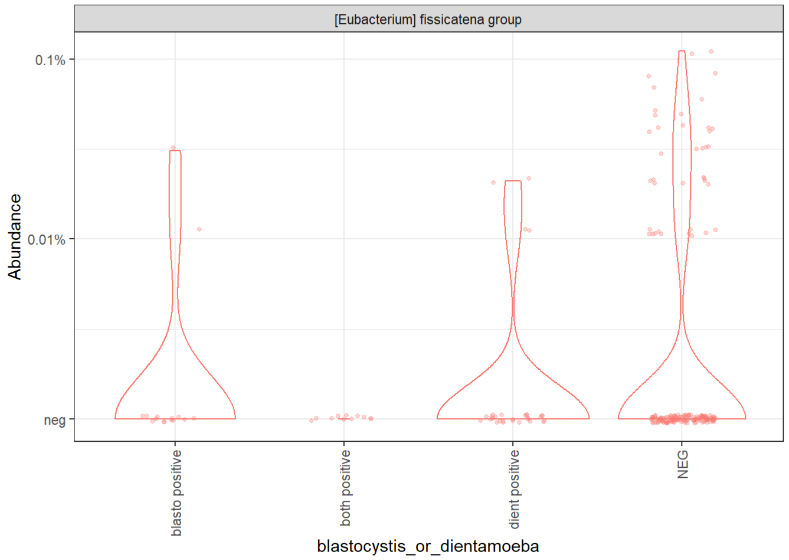


```
## [1] ""
## [1] "-------------- TESTING ------------"
## [1] "17 ASV00007 Genus Alistipes"
## [1] ""
## [1] "-------------- TESTING ------------"
## [1] "18 ASV00099 Genus Blautia"
## [1] ""
## [1] "-------------- TESTING ------------"
## [1] "19 ASV00577 Genus Lachnospiraceae UCG-003"
## [1] ""
## [1] "-------------- TESTING ------------"
## [1] "20 ASV00513 Genus [Eubacterium] hallii group"
## p_val_gee_dientamoeba_positive 0.234
## p_val_gee_blastocystis_positive 0.000505
## p_val_gee_blasto_dient_pos 0.0999
```

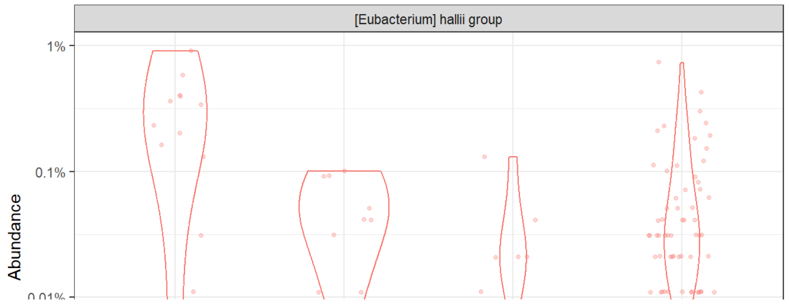

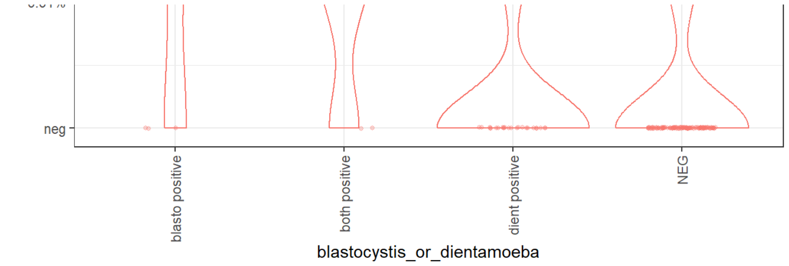


```
## [1] ""
## [1] "-------------- TESTING ------------"
## [1] "21 ASV00358 Genus Rikenellaceae RC9 gut group"
## [1] ""
## [1] "-------------- TESTING ------------"
## [1] "22 ASV01122 Genus Butyricimonas"
## [1] ""
## [1] "-------------- TESTING ------------"
## [1] "23 ASV00177 Genus Odoribacter"
## [1] ""
## [1] "-------------- TESTING ------------"
## [1] "24 ASV00508 Genus Paraprevotella"
## [1] ""
## [1] "-------------- TESTING ------------"
## [1] "25 ASV00120 Genus Prevotella_7"
## [1] ""
## [1] "-------------- TESTING ------------"
## [1] "26 ASV01175 Genus Prevotella"
## [1] ""
## [1] "-------------- TESTING ------------"
## [1] "27 ASV00059 Genus Prevotella_9"
## [1] ""
## [1] "-------------- TESTING ------------"
## [1] "28 ASV01319 Genus Coprobacter"
## [1] ""
## [1] "-------------- TESTING ------------"
## [1] "29 ASV00106 Genus Barnesiella"
## p_val_gee_dientamoeba_positive 4.95e-05
## p_val_gee_blastocystis_positive 0.396
## p_val_gee_blasto_dient_pos 0.05
```

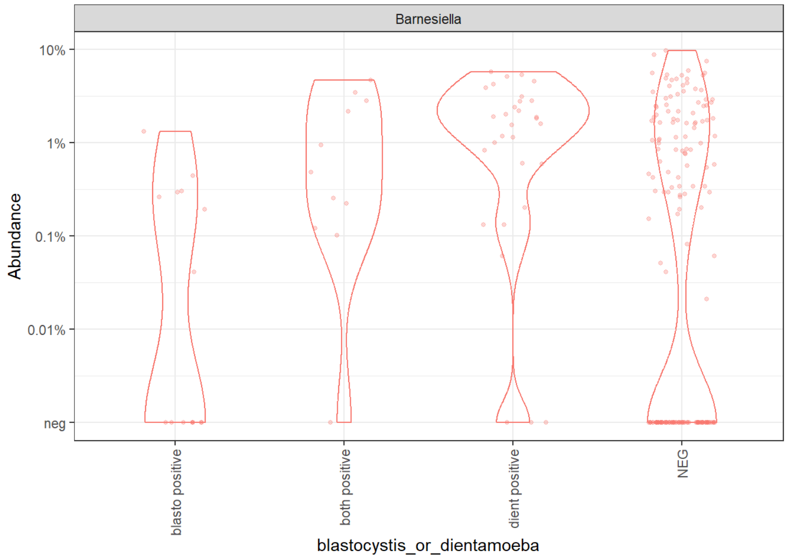


```
## [1] ""
## [1] "-------------- TESTING ------------"
## [1] "30 ASV00528 Genus Porphyromonas"
## [1] ""
## [1] "-------------- TESTING ------------"
## [1] "31 ASV00002 Genus Bacteroides"
## [1] ""
## [1] "-------------- TESTING ------------"
## [1] "32 ASV00020 Genus Parabacteroides"
```

```
## [1] ""
## [1] "-------------- TESTING ------------"
## [1] "33 ASV00056 Genus Lachnospiraceae NK4A136 group"
## p_val_gee_dientamoeba_positive 0.000268
## p_val_gee_blastocystis_positive 0.606
## p_val_gee_blasto_dient_pos 0.00765
```

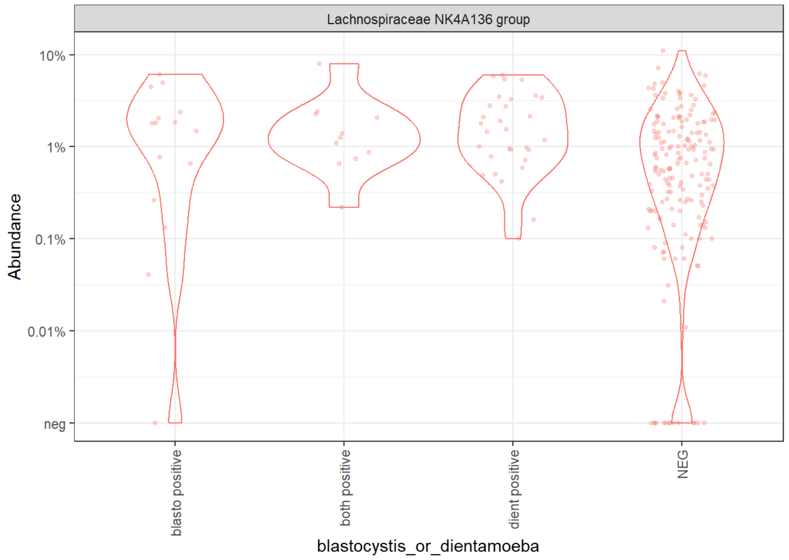


```
## [1] ""
## [1] "-------------- TESTING ------------"
## [1] "34 ASV00130 Genus Lachnoclostridium"
## [1] ""
## [1] "-------------- TESTING ------------"
## [1] "35 ASV00399 Genus Lachnospiraceae AC2044 group"
## [1] ""
## [1] "-------------- TESTING ------------"
## [1] "36 ASV00244 Genus Hungatella"
## p_val_gee_dientamoeba_positive 2.68e-05
## p_val_gee_blastocystis_positive 0.0531
## p_val_gee_blasto_dient_pos 0.000812
```

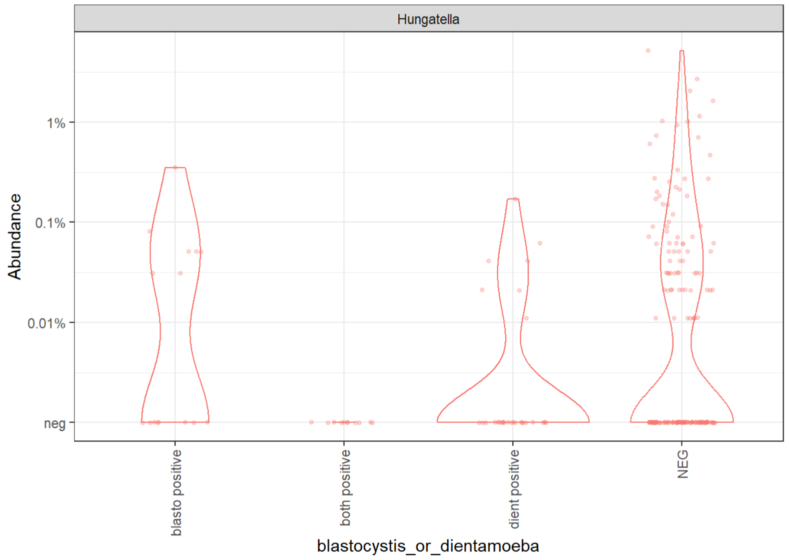


```
## [1] ""
## [1] "-------------- TESTING ------------"
## [1] "37 ASV01206 Genus Howardella"
## [1] ""
## [1] "-------------- TESTING ------------"
## [1] "38 ASV00124 Genus [Eubacterium] xylanophilum group"
## [1] ""
## [1] "-------------- TESTING ------------"
## [1] "39 ASV00102 Genus [Eubacterium] eligens group"
## [1] ""
```

```
## [1] "-------------- TESTING ------------"
## [1] "40 ASV00209 Genus Lachnospira"
## p_val_gee_dientamoeba_positive 0.486
## p_val_gee_blastocystis_positive 0.000957
## p_val_gee_blasto_dient_pos 0.0591
```

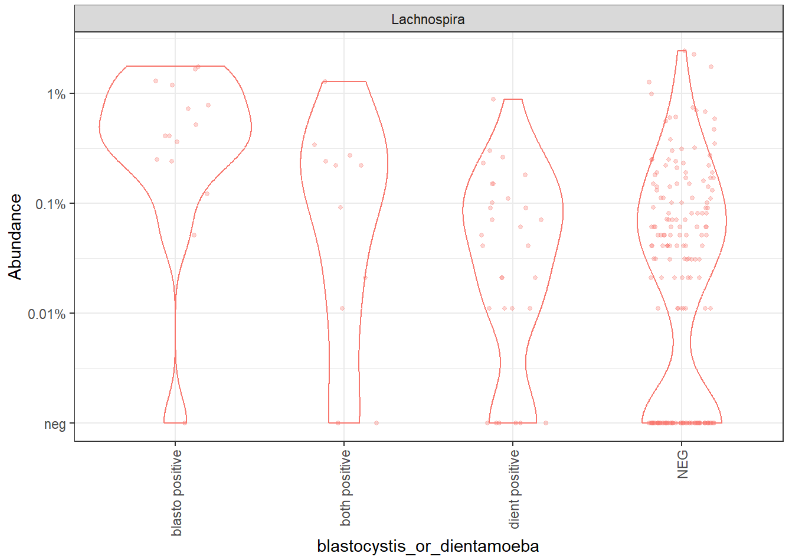


```
## [1] ""
## [1] "-------------- TESTING ------------"
## [1] "41 ASV00361 Genus Lachnospiraceae ND3007 group"
## p_val_gee_dientamoeba_positive 0.989
## p_val_gee_blastocystis_positive 6.64e-05
## p_val_gee_blasto_dient_pos 0.0418
```

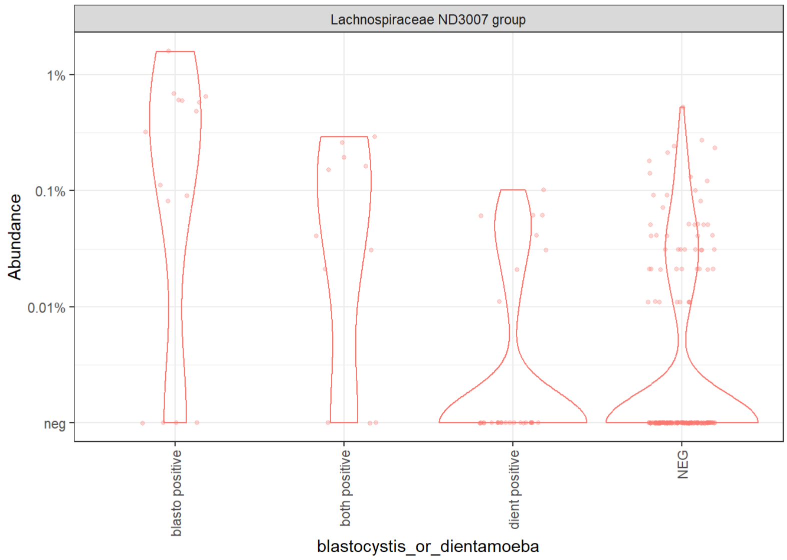


```
## [1] ""
## [1] "-------------- TESTING ------------"
## [1] "42 ASV00355 Genus [Eubacterium] ruminantium group"
## [1] ""
## [1] "-------------- TESTING ------------"
## [1] "43 ASV00075 Genus Coprococcus"
## [1] ""
## [1] "-------------- TESTING ------------"
## [1] "44 ASV00182 Genus Anaerostipes"
## [1] ""
## [1] "-------------- TESTING ------------"
## [1] "45 ASV00271 Genus [Eubacterium] ventriosum group"
## [1] ""
## [1] "-------------- TESTING ------------"
## [1] "46 ASV01264 Genus Frisingicoccus"
## [1] ""
## [1] "-------------- TESTING ------------"
```

```
## [1] "47 ASV01262 Genus GCA-900066575"
## [1] ""
## [1] "-------------- TESTING ------------"
## [1] "48 ASV00269 Genus Butyricicoccus"
## [1] ""
## [1] "-------------- TESTING ------------"
## [1] "49 ASV01407 Genus Defluviitaleaceae UCG-011"
## [1] ""
## [1] "-------------- TESTING ------------"
## [1] "50 ASV00515 Genus Lachnospiraceae UCG-010"
## [1] ""
## [1] "-------------- TESTING ------------"
## [1] "51 ASV00802 Genus Tuzzerella"
## [1] ""
## [1] "-------------- TESTING ------------"
## [1] "52 ASV01987 Genus Fusobacterium"
## p_val_gee_dientamoeba_positive 0.0447
## p_val_gee_blastocystis_positive 0.00012
## p_val_gee_blasto_dient_pos 0.000722
```

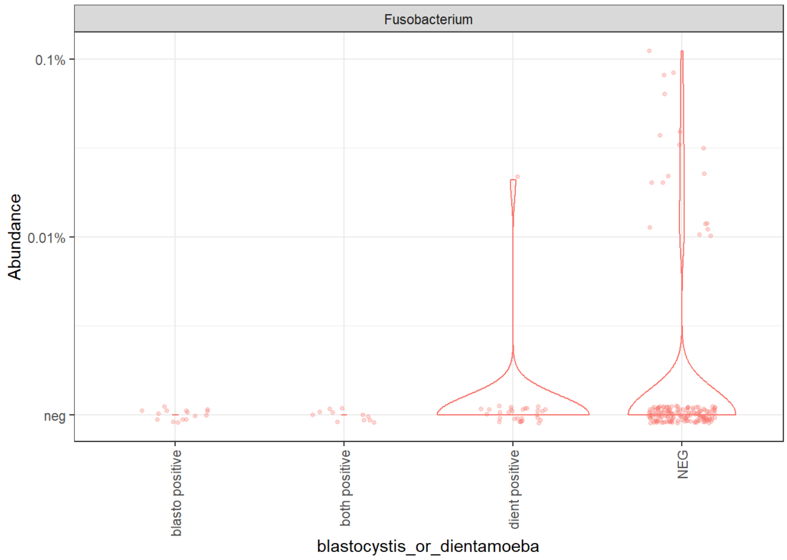


```
## [1] ""
## [1] "-------------- TESTING ------------"
## [1] "53 ASV00273 Genus Parasutterella"
## [1] ""
## [1] "-------------- TESTING ------------"
## [1] "54 ASV00214 Genus Sutterella"
## [1] ""
## [1] "-------------- TESTING ------------"
## [1] "55 ASV00988 Genus Oxalobacter"
## [1] ""
## [1] "-------------- TESTING ------------"
## [1] "56 ASV01621 Genus Aggregatibacter"
## [1] ""
## [1] "-------------- TESTING ------------"
## [1] "57 ASV00068 Genus Haemophilus"
## [1] ""
## [1] "-------------- TESTING ------------"
## [1] "58 ASV01668 Genus Actinobacillus"
## [1] ""
## [1] "-------------- TESTING ------------"
## [1] "59 ASV00769 Genus Enterobacter"
## [1] ""
## [1] "-------------- TESTING ------------"
## [1] "60 ASV00727 Genus Klebsiella"
## p_val_gee_dientamoeba_positive 0.0388
## p_val_gee_blastocystis_positive 0.000434
## p_val_gee_blasto_dient_pos 0.00295
```

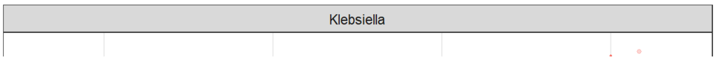

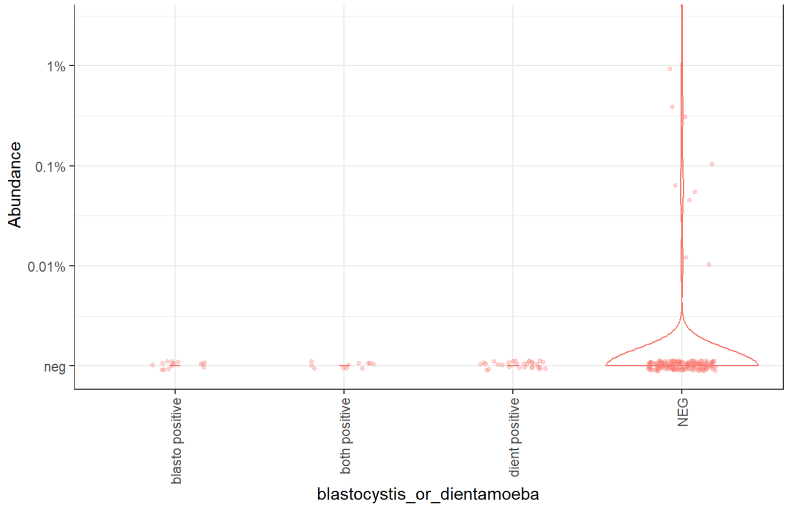


```
## [1] ""
## [1] "-------------- TESTING ------------"
## [1] "61 ASV00511 Genus Citrobacter"
## [1] ""
## [1] "-------------- TESTING ------------"
## [1] "62 ASV00041 Genus Escherichia-Shigella"
## p_val_gee_dientamoeba_positive 9.57e-05
## p_val_gee_blastocystis_positive 8.49e-09
## p_val_gee_blasto_dient_pos 1.34e-07
```

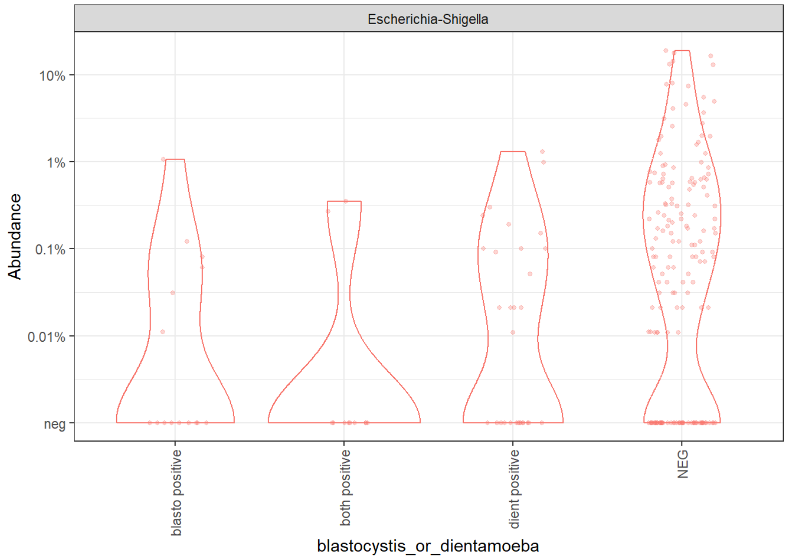


```
## [1] ""
## [1] "-------------- TESTING ------------"
## [1] "63 ASV01371 Genus TM7x"
## [1] ""
## [1] "-------------- TESTING ------------"
## [1] "64 ASV01546 Genus Actinomyces"
## p_val_gee_dientamoeba_positive 0.0115
## p_val_gee_blastocystis_positive 0.000211
## p_val_gee_blasto_dient_pos 7.92e-05
```

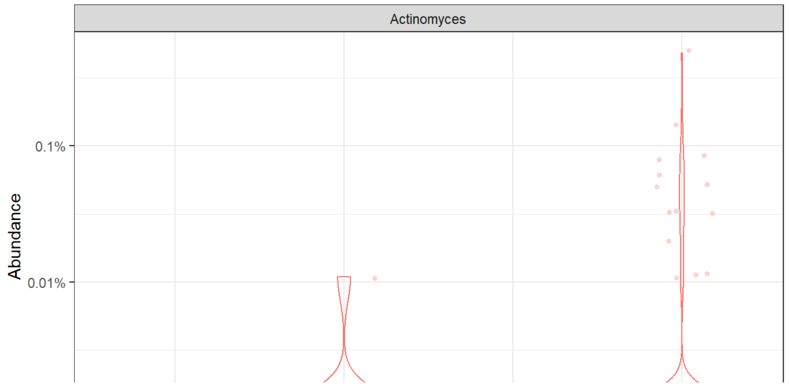

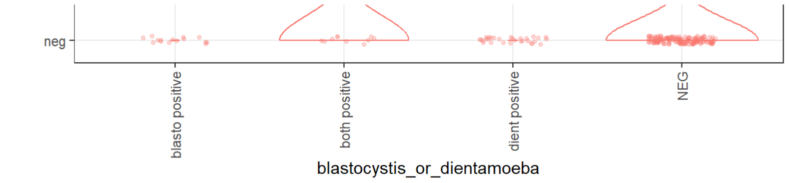


```
## [1] ""
## [1] "-------------- TESTING ------------"
## [1] "65 ASV00094 Genus Bifidobacterium"
## [1] ""
## [1] "-------------- TESTING ------------"
## [1] "66 ASV01145 Genus Eggerthella"
## [1] ""
## [1] "-------------- TESTING ------------"
## [1] "67 ASV01898 Genus Senegalimassilia"
## [1] ""
## [1] "-------------- TESTING ------------"
## [1] "68 ASV01210 Genus Slackia"
## [1] ""
## [1] "-------------- TESTING ------------"
## [1] "69 ASV00283 Genus Collinsella"
## [1] ""
## [1] "-------------- TESTING ------------"
## [1] "70 ASV00024 Genus Akkermansia"
## [1] ""
## [1] "-------------- TESTING ------------"
## [1] "71 ASV00614 Genus Bilophila"
## [1] ""
## [1] "-------------- TESTING ------------"
## [1] "72 ASV00873 Genus Desulfovibrio"
## [1] ""
## [1] "-------------- TESTING ------------"
## [1] "73 ASV00371 Genus Holdemania"
## p_val_gee_dientamoeba_positive 0.0645
## p_val_gee_blastocystis_positive 0.000161
## p_val_gee_blasto_dient_pos 0.00353
```

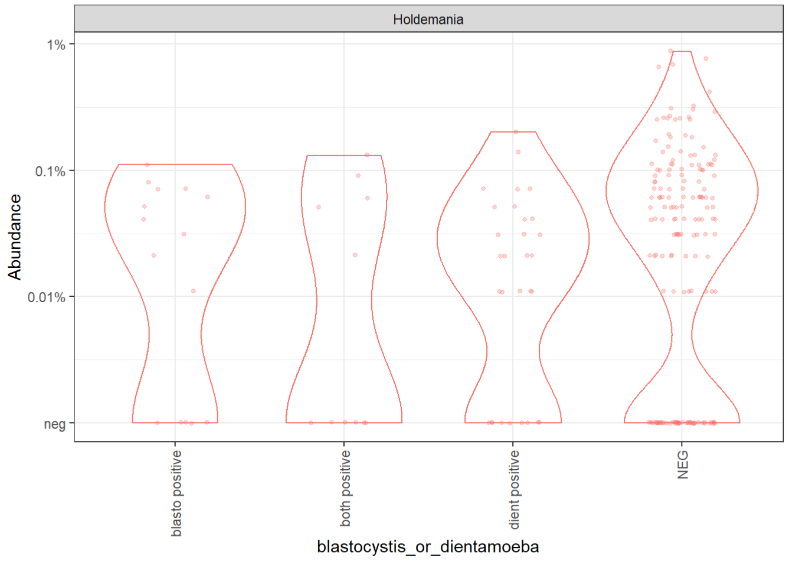


```
## [1] ""
## [1] "-------------- TESTING ------------"
## [1] "74 ASV00788 Genus Dielma"
## p_val_gee_dientamoeba_positive 0.0379
## p_val_gee_blastocystis_positive 0.000123
## p_val_gee_blasto_dient_pos 0.000934
```

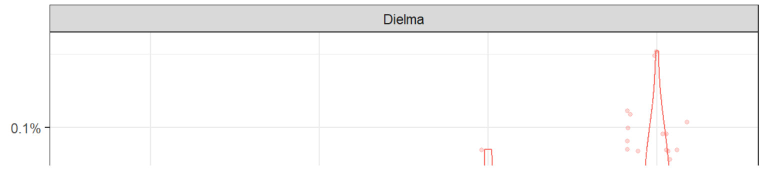

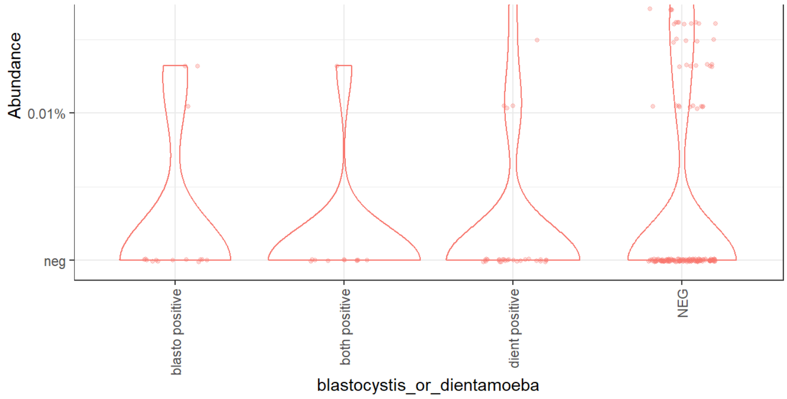


```
## [1] ""
## [1] "-------------- TESTING ------------"
## [1] "75 ASV01383 Genus Faecalitalea"
## [1] ""
## [1] "-------------- TESTING ------------"
## [1] "76 ASV00885 Genus [Clostridium] innocuum group"
## [1] ""
## [1] "-------------- TESTING ------------"
## [1] "77 ASV00301 Genus Holdemanella"
## [1] ""
## [1] "-------------- TESTING ------------"
## [1] "78 ASV00113 Genus Erysipelotrichaceae UCG-003"
## [1] ""
## [1] "-------------- TESTING ------------"
## [1] "79 ASV00880 Genus Coprobacillus"
## p_val_gee_dientamoeba_positive 0.0129
## p_val_gee_blastocystis_positive 4.27e-05
## p_val_gee_blasto_dient_pos 0.000535
```

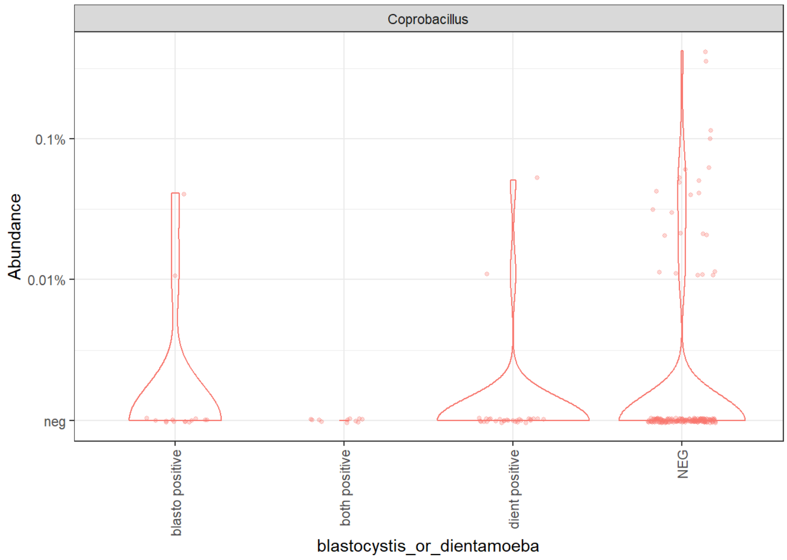


```
## [1] ""
## [1] "-------------- TESTING ------------"
## [1] "80 ASV00473 Genus Erysipelatoclostridium"
## [1] ""
## [1] "-------------- TESTING ------------"
## [1] "81 ASV00526 Genus Catenibacterium"
## [1] ""
## [1] "-------------- TESTING ------------"
## [1] "82 ASV00079 Genus Turicibacter"
## [1] ""
## [1] "-------------- TESTING ------------"
## [1] "83 ASV00981 Genus Lactococcus"
## [1] ""
## [1] "-------------- TESTING ------------"
## [1] "84 ASV00185 Genus Streptococcus"
## [1] ""
```

```
## [1] "-------------- TESTING ------------"
## [1] "85 ASV00796 Genus Lactiplantibacillus"
## p_val_gee_dientamoeba_positive 0.000666
## p_val_gee_blastocystis_positive 0.819
## p_val_gee_blasto_dient_pos 0.148
```

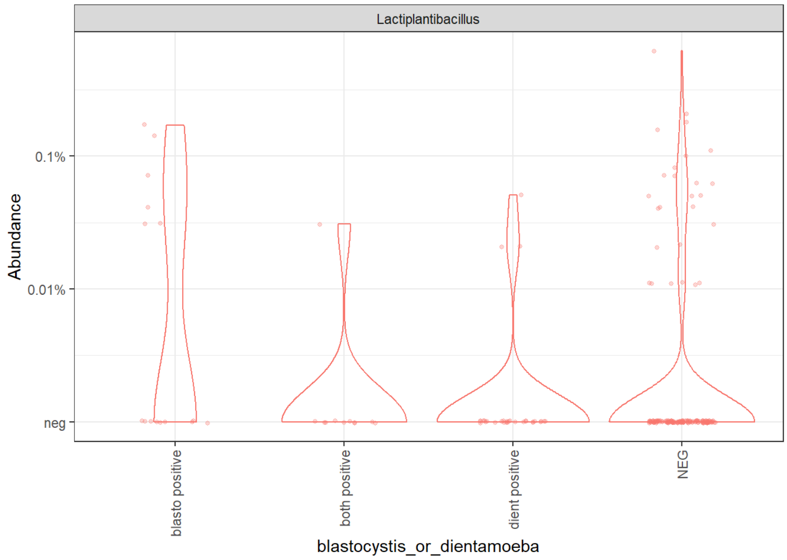


```
## [1] ""
## [1] "-------------- TESTING ------------"
## [1] "86 ASV00855 Genus Lacticaseibacillus"
## [1] ""
## [1] "-------------- TESTING ------------"
## [1] "87 ASV01112 Genus Lactobacillus"
## [1] ""
## [1] "-------------- TESTING ------------"
## [1] "88 ASV00583 Genus Granulicatella"
## p_val_gee_dientamoeba_positive 3.4e-06
## p_val_gee_blastocystis_positive 0.01
## p_val_gee_blasto_dient_pos 4.73e-05
```

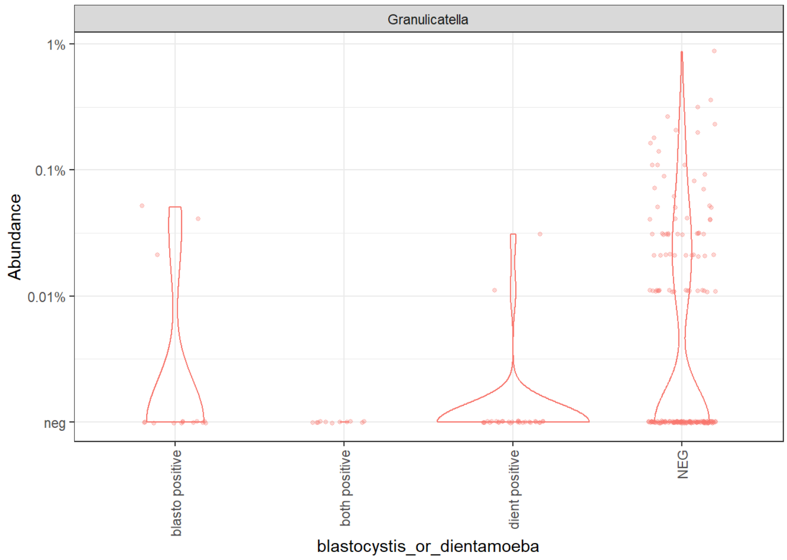


```
## [1] ""
## [1] "-------------- TESTING ------------"
## [1] "89 ASV01756 Genus Abiotrophia"
## [1] ""
## [1] "-------------- TESTING ------------"
## [1] "90 ASV01883 Genus Gemella"
## p_val_gee_dientamoeba_positive 0.00198
## p_val_gee_blastocystis_positive 0.000119
## p_val_gee_blasto_dient_pos 2.03e-05
```

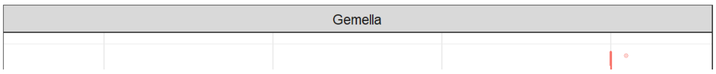

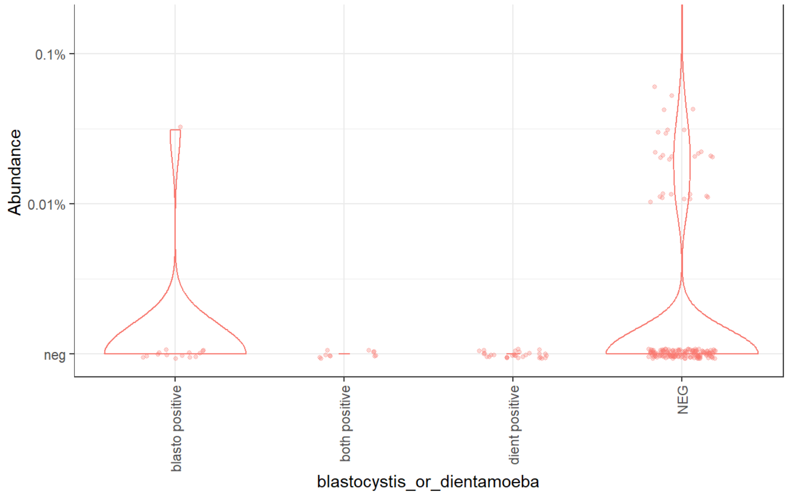


```
## [1] ""
## [1] "-------------- TESTING ------------"
## [1] "91 ASV00115 Genus Veillonella"
## [1] ""
## [1] "-------------- TESTING ------------"
## [1] "92 ASV00004 Genus Dialister"
## [1] ""
## [1] "-------------- TESTING ------------"
## [1] "93 ASV01509 Genus Allisonella"
## [1] ""
## [1] "-------------- TESTING ------------"
## [1] "94 ASV00893 Genus Megasphaera"
## [1] ""
## [1] "-------------- TESTING ------------"
## [1] "95 ASV00744 Genus Megamonas"
## [1] ""
## [1] "-------------- TESTING ------------"
## [1] "96 ASV01065 Genus Acidaminococcus"
## [1] ""
## [1] "-------------- TESTING ------------"
## [1] "97 ASV00081 Genus Phascolarctobacterium"
## [1] ""
## [1] "-------------- TESTING ------------"
## [1] "98 ASV00197 Genus Succiniclasticum"
## [1] ""
## [1] "-------------- TESTING ------------"
## [1] "99 ASV02313 Genus Peptococcus"
## [1] ""
## [1] "-------------- TESTING ------------"
## [1] "100 ASV02228 Genus Anaerofustis"
## p_val_gee_dientamoeba_positive 0.0213
## p_val_gee_blastocystis_positive 0.000182
## p_val_gee_blasto_dient_pos 0.000174
```

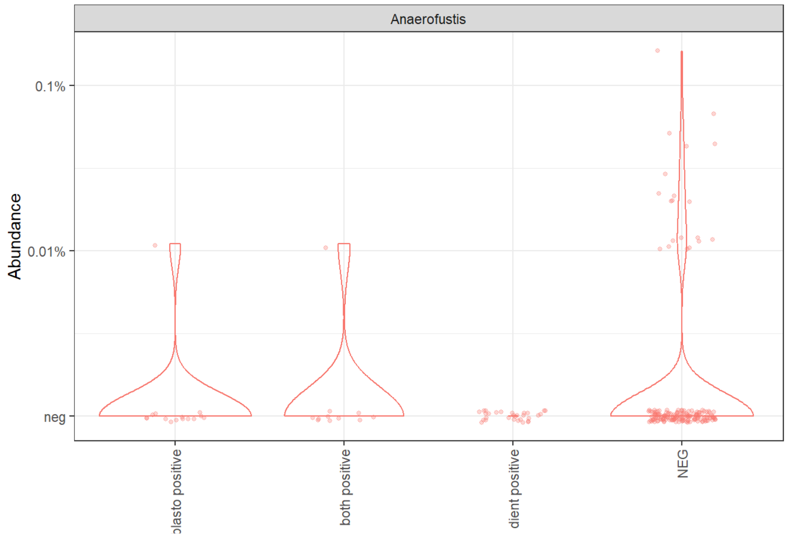

blastocystis_or_dientamoeba

```
## [1] ""
## [1] "-------------- TESTING ------------"
## [1] "101 ASV01542 Genus Ezakiella"
## [1] ""
## [1] "-------------- TESTING ------------"
## [1] "102 ASV01197 Genus Fenollaria"
## [1] ""
## [1] "-------------- TESTING ------------"
## [1] "103 ASV00503 Genus Family XIII UCG-001"
## [1] ""
## [1] "-------------- TESTING ------------"
## [1] "104 ASV00350 Genus Family XIII AD3011 group"
## [1] ""
## [1] "-------------- TESTING ------------"
## [1] "105 ASV00080 Genus Intestinibacter"
## [1] ""
## [1] "-------------- TESTING ------------"
## [1] "106 ASV00036 Genus Romboutsia"
## [1] ""
## [1] "-------------- TESTING ------------"
## [1] "107 ASV00194 Genus Terrisporobacter"
## [1] ""
## [1] "-------------- TESTING ------------"
## [1] "108 ASV01662 Genus Catabacter"
## [1] ""
## [1] "-------------- TESTING ------------"
## [1] "109 ASV01352 Genus Christensenella"
## [1] ""
## [1] "-------------- TESTING ------------"
## [1] "110 ASV00053 Genus Christensenellaceae R-7 group"
## [1] ""
## [1] "-------------- TESTING ------------"
## [1] "111 ASV00116 Genus Monoglobus"
## [1] ""
## [1] "-------------- TESTING ------------"
## [1] "112 ASV00060 Genus UCG-005"
## [1] ""
## [1] "-------------- TESTING ------------"
## [1] "113 ASV00043 Genus UCG-002"
## [1] ""
## [1] "-------------- TESTING ------------"
## [1] "114 ASV00147 Genus UCG-003"
## [1] ""
## [1] "-------------- TESTING ------------"
## [1] "115 ASV01190 Genus Oscillospira"
## [1] ""
## [1] "-------------- TESTING ------------"
## [1] "116 ASV00125 Genus Oscillibacter"
## p_val_gee_dientamoeba_positive 0.000877
## p_val_gee_blastocystis_positive 0.218
## p_val_gee_blasto_dient_pos 0.00887
```

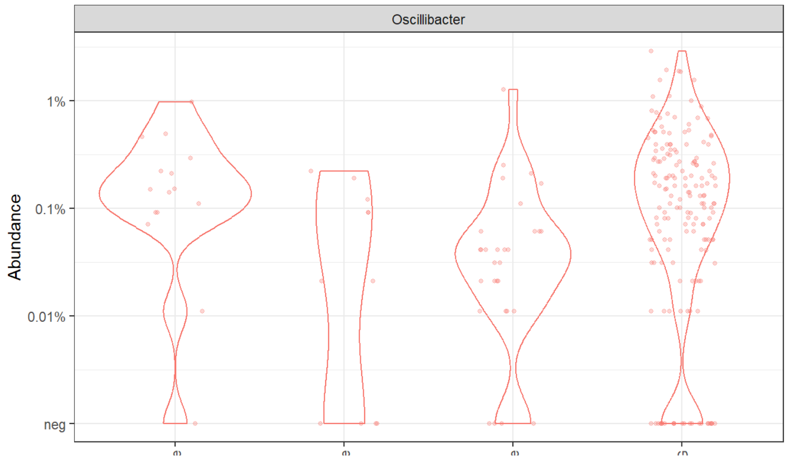

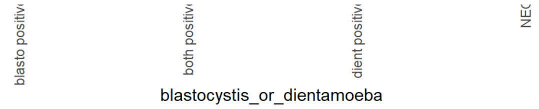


```
## [1] ""
## [1] "-------------- TESTING ------------"
## [1] "117 ASV00468 Genus Colidextribacter"
## [1] ""
## [1] "-------------- TESTING ------------"
## [1] "118 ASV00231 Genus Flavonifractor"
## p_val_gee_dientamoeba_positive 4.82e-08
## p_val_gee_blastocystis_positive 0.145
## p_val_gee_blasto_dient_pos 5.78e-05
```

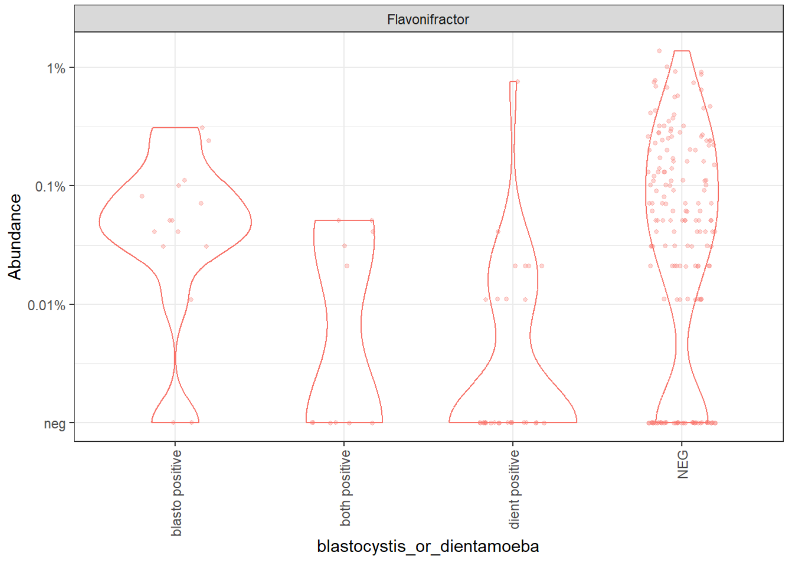


```
## [1] ""
## [1] "-------------- TESTING ------------"
## [1] "119 ASV01629 Genus Pseudoflavonifractor"
## [1] ""
## [1] "-------------- TESTING ------------"
## [1] "120 ASV00496 Genus Intestinimonas"
## [1] ""
## [1] "-------------- TESTING ------------"
## [1] "121 ASV00222 Genus NK4A214 group"
## p_val_gee_dientamoeba_positive 3.77e-05
## p_val_gee_blastocystis_positive 0.317
## p_val_gee_blasto_dient_pos 0.000107
```

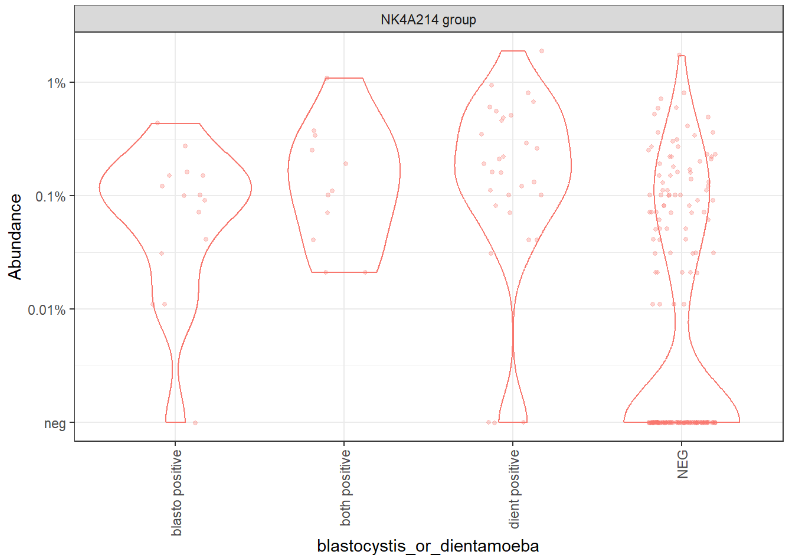


```
## [1] ""
## [1] "-------------- TESTING ------------"
## [1] "122 ASV00151 Genus Incertae Sedis"
```

```
## [1] ""
## [1] "-------------- TESTING ------------"
## [1] "123 ASV00524 Genus DTU089"
## p_val_gee_dientamoeba_positive 0.162
## p_val_gee_blastocystis_positive 6.53e-05
## p_val_gee_blasto_dient_pos 0.00461
```

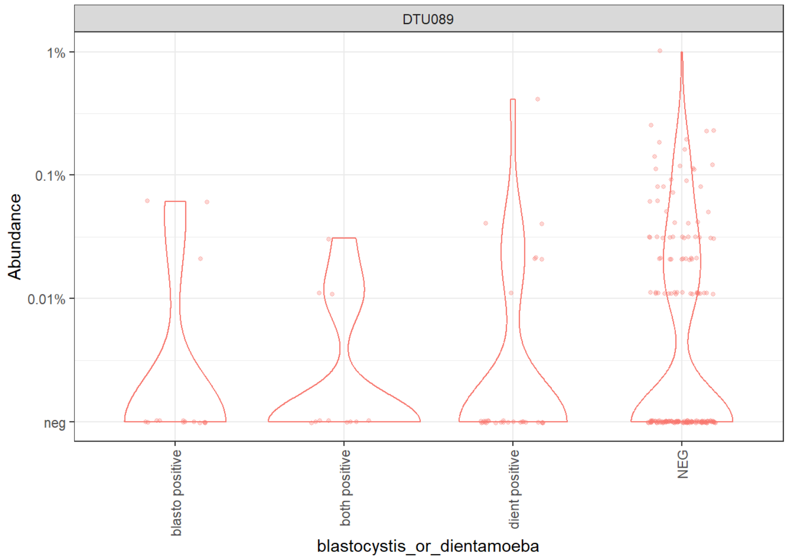


```
## [1] ""
## [1] "-------------- TESTING ------------"
## [1] "124 ASV00123 Genus Ruminococcus"
## [1] ""
## [1] "-------------- TESTING ------------"
## [1] "125 ASV00052 Genus CAG-352"
## [1] ""
## [1] "-------------- TESTING ------------"
## [1] "126 ASV00876 Genus Paludicola"
## p_val_gee_dientamoeba_positive 0.604
## p_val_gee_blastocystis_positive 3.39e-05
## p_val_gee_blasto_dient_pos 0.249
```

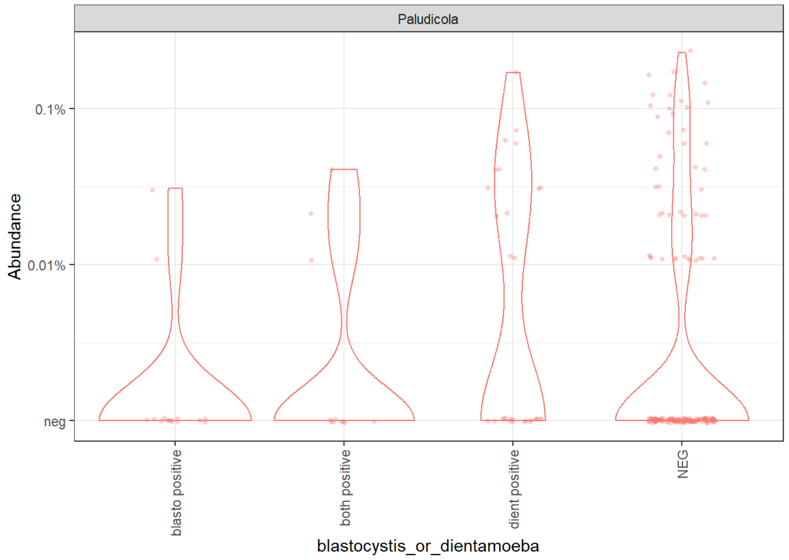


```
## [1] ""
## [1] "-------------- TESTING ------------"
## [1] "127 ASV00006 Genus Subdoligranulum"
## [1] ""
## [1] "-------------- TESTING ------------"
## [1] "128 ASV00148 Genus UBA1819"
## p_val_gee_dientamoeba_positive 4.38e-05
## p_val_gee_blastocystis_positive 2.01e-10
## p_val_gee_blasto_dient_pos 6.06e-09
```

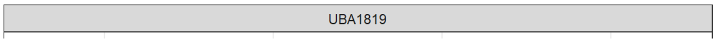

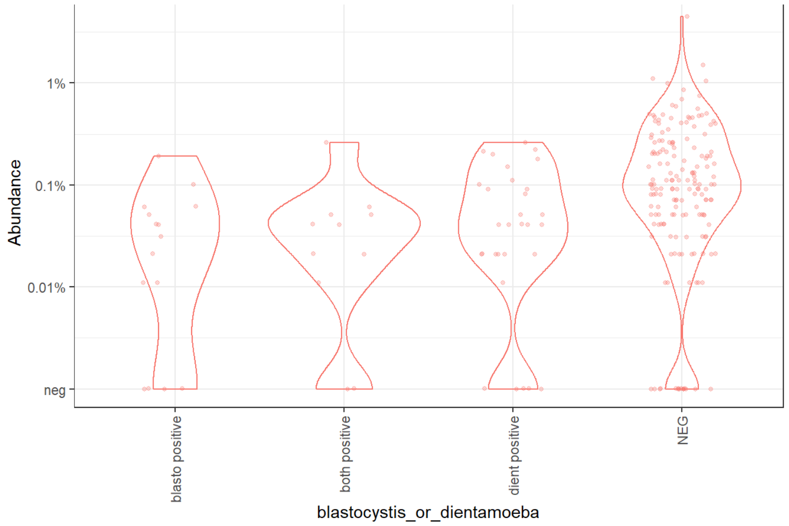


```
## [1] ""
## [1] "-------------- TESTING ------------"
## [1] "129 ASV01984 Genus Fournierella"
## [1] ""
## [1] "-------------- TESTING ------------"
## [1] "130 ASV01591 Genus Anaerofilum"
## [1] ""
## [1] "-------------- TESTING ------------"
## [1] "131 ASV00001 Genus Faecalibacterium"
## [1] ""
## [1] "-------------- TESTING ------------"
## [1] "132 ASV01513 Genus Candidatus Soleaferrea"
## p_val_gee_dientamoeba_positive 0.023
## p_val_gee_blastocystis_positive 0.000167
## p_val_gee_blasto_dient_pos 0.00037
```

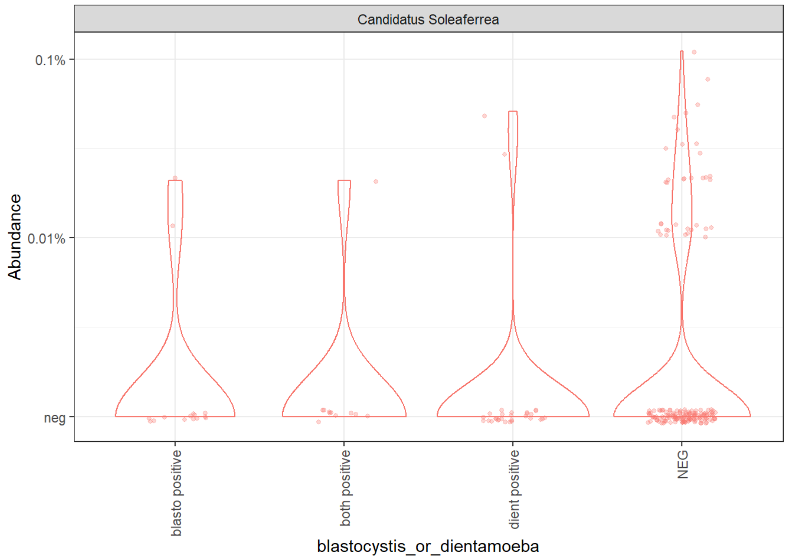


```
## [1] ""
## [1] "-------------- TESTING ------------"
## [1] "133 ASV02446 Genus Negativibacillus"
## [1] ""
## [1] "-------------- TESTING ------------"
## [1] "134 ASV00866 Genus Anaerotruncus"
## [1] ""
## [1] "-------------- TESTING ------------"
## [1] "135 ASV00211 Genus [Eubacterium] siraeum group"
## [1] ""
## [1] "-------------- TESTING ------------"
## [1] "136 ASV00268 Genus Sarcina"
## [1] ""
## [1] "-------------- TESTING ------------"
## [1] "137 ASV00072 Genus Clostridium sensu stricto 1"
```

```
## [1] ""
## [1] "-------------- TESTING ------------"
## [1] "138 ASV00618 Genus Tyzzerella"
## [1] ""
## [1] "-------------- TESTING ------------"
## [1] "139 ASV01527 Genus GCA-900066755"
```

```
res_parasites_adj <- res_parasites %>%
    group_by(taxrank_name) %>%
    mutate(
        tested_entities = n(),
        padj_gee_blasto_dient_pos = p.adjust(p_val_gee_blasto_dient_pos, method = "bonferroni"),
        padj_gee_blasto_pos = p.adjust(p_val_gee_blastocystis_positive, method = "bonferroni"),
        padj_gee_dient_pos = p.adjust(p_val_gee_dientamoeba_positive, method = "bonferroni"),
        )


my_ts <- strftime(Sys.time(), "%Y%m%dT%H%M%S")
xlsx::write.xlsx(data.frame(res_parasites_adj),
                 file = file.path("./graphs_and_outputs",
paste0("results_gee_CLR_STANDARDISED_REVISION_of_all_levels_against_parasites", my_ts, ".xlsx")),
                 row.names = F)


write_rds(
    x = res_parasites,
    file = file.path("./saved_data", paste0("results_gee_CLR_STANDARDISED_REVISION_against_parasites", my_ts, ".rds"))
)
```

Unify the two tables: the linear and CLR model tables merged

```
res_gee_linear <- read_rds(
    file = file.path("./saved_data", "results_gee_LINEAR_RAREFIED_against_parasites20230829T120921.rds")
)

colnames(res_gee_linear)[4:ncol(res_gee_linear)] <- paste0(colnames(res_gee_linear)[4:ncol(res_gee_linear)], ".lin")
res_gee_clr <- res_parasites_adj
colnames(res_gee_clr)[4:ncol(res_gee_clr)] <- paste0(colnames(res_gee_clr)[4:ncol(res_gee_clr)], ".clr")

results <- left_join(
    res_gee_clr, res_gee_linear, by = c("asv", "taxrank_name", "taxon_name")
) %>% mutate(note = " ") %>%
    select(
        asv, taxrank_name, taxon_name,
        note,
        coef_gee_blastocystis_positive.clr,
        coef_gee_dientamoeba_positive.clr,
        padj_gee_blasto_pos.clr,
        padj_gee_dient_pos.clr,
        coef_gee_blastocystis_positive.lin,
        coef_gee_dientamoeba_positive.lin,
        padj_gee_blasto_pos.lin,
        padj_gee_dient_pos.lin,
        everything()
    )


my_ts <- strftime(Sys.time(), "%Y%m%dT%H%M%S")
xlsx::write.xlsx(data.frame(results),
                 file = file.path("./graphs_and_outputs", paste0("results_gee_COMPARED_TWO_METHODS", my_ts, ".xlsx")),
                 row.names = F)

write_rds(
    x = results,
    file = file.path("./saved_data", paste0("results_gee_COMPARED_TWO_METHODS", my_ts, ".rds"))
)
```

Comparison of the two result sets as shown in Results

```
#lacti
```

```
print(t(results %>% filter(str_starts(taxon_name, "Lacti"))))
```

```
##                                      [,1]                   [,2]
## asv                                  "ASV00796"             "ASV00855"
## taxrank_name                         "Genus"                "Genus"
## taxon_name                           "Lactiplantibacillus" "Lacticaseibacillus"
## note                                 " "                    " "
## coef_gee_blastocystis_positive.clr   "-0.0162"              "-0.0760"
## coef_gee_dientamoeba_positive.clr    "-0.1071"              "-0.0744"
## padj_gee_blasto_pos.clr              "1"                    "1"
## padj_gee_dient_pos.clr               "0.0926"               "1.0000"
## coef_gee_blastocystis_positive.lin   "0.762"                "1.175"
## coef_gee_dientamoeba_positive.lin    "-0.989"               "-0.761"
## padj_gee_blasto_pos.lin              "1"                    "1"
## padj_gee_dient_pos.lin               "1"                    "1"
## p_val_gee_blasto_dient_pos.clr       "0.148"                "0.325"
## p_val_gee_on_intervention1.clr       "6.04e-06"             "1.05e-06"
## p_val_time_23.clr                    "0.721"                "0.536"
## coef_gee_blasto_dient_pos.clr        "-0.0613"              "-0.0600"
## coef_gee_on_intervention1.clr        "0.266"                "0.297"
## coef_gee_time_23.clr                 "0.00916"              "0.01388"
## p_val_gee_blastocystis_positive.clr "0.819"                 "0.479"
## p_val_gee_dientamoeba_positive.clr   "0.000666"             "0.100820"
## tested_entities.clr                  "139"                  "139"
## padj_gee_blasto_dient_pos.clr        "1"                    "1"
## p_val_gee_blasto_dient_pos.lin       "0.852"                "0.728"
## p_val_gee_on_intervention1.lin       "0.001722"             "0.000478"
## p_val_time_23.lin                    "0.181"                "0.336"
## coef_gee_blasto_dient_pos.lin        "-0.119"               " 0.347"
## coef_gee_on_intervention1.lin        "3.05"                 "3.38"
## coef_gee_time_23.lin                 "-0.227"               "-0.141"
## p_val_gee_blastocystis_positive.lin "0.452"                 "0.549"
## p_val_gee_dientamoeba_positive.lin   "0.0238"               "0.1439"
## tested_entities.lin                  "139"                  "139"
## padj_gee_blasto_dient_pos.lin        "1"                    "1"
```

```
results_sign_blasto <- results %>%
    filter(padj_gee_blasto_pos.lin < 0.05 & padj_gee_blasto_pos.clr < 0.05)
t(results_sign_blasto)
```

```
##                                      [,1]
## asv                                  "ASV00148"
## taxrank_name                         "Genus"
## taxon_name                           "UBA1819"
## note                                 " "
## coef_gee_blastocystis_positive.clr   "-0.692"
## coef_gee_dientamoeba_positive.clr    "-0.449"
## padj_gee_blasto_pos.clr              "2.79e-08"
## padj_gee_dient_pos.clr               "0.00608"
## coef_gee_blastocystis_positive.lin   "-14.9"
## coef_gee_dientamoeba_positive.lin    "-12.7"
## padj_gee_blasto_pos.lin              "0.00283"
## padj_gee_dient_pos.lin               "0.00879"
## p_val_gee_blasto_dient_pos.clr       "6.06e-09"
## p_val_gee_on_intervention1.clr       "0.535"
## p_val_time_23.clr                    "0.183"
## coef_gee_blasto_dient_pos.clr        "-0.613"
## coef_gee_on_intervention1.clr        "0.0794"
## coef_gee_time_23.clr                 "-0.153"
## p_val_gee_blastocystis_positive.clr "2.01e-10"
## p_val_gee_dientamoeba_positive.clr   "4.38e-05"
## tested_entities.clr                  "139"
## padj_gee_blasto_dient_pos.clr        "8.42e-07"
## p_val_gee_blasto_dient_pos.lin       "2.12e-05"
## p_val_gee_on_intervention1.lin       "0.344"
## p_val_time_23.lin                    "0.149"
## coef_gee_blasto_dient_pos.lin        "-15"
## coef_gee_on_intervention1.lin        "5.99"
## coef_gee_time_23.lin                 "-4.58"
```

```
## p_val_gee_blastocystis_positive.lin "2.04e-05"
## p_val_gee_dientamoeba_positive.lin  "6.32e-05"
## tested_entities.lin                 "139"
## padj_gee_blasto_dient_pos.lin       "0.00295"
```

```
results_sign_dient <- results %>%
    filter(padj_gee_dient_pos.lin < 0.05 & padj_gee_dient_pos.clr < 0.05)
t(results_sign_dient)
```

```
##                                     [,1]                 [,2]
## asv                                 "ASV00041"           "ASV00283"
## taxrank_name                        "Family"             "Family"
## taxon_name                          "Enterobacteriaceae" "Coriobacteriaceae"
## note                                " "                  " "
## coef_gee_blastocystis_positive.clr  "-0.907"             "-0.198"
## coef_gee_dientamoeba_positive.clr   "-0.837"             "-0.322"
## padj_gee_blasto_pos.clr             "3.37e-02"           "1.00e+00"
## padj_gee_dient_pos.clr              "1.44e-02"           "3.53e-02"
## coef_gee_blastocystis_positive.lin  "-131.995"           "  -0.406"
## coef_gee_dientamoeba_positive.lin   "-135.58"            "  -7.35"
## padj_gee_blasto_pos.lin             "0.13938"            "1.00000"
## padj_gee_dient_pos.lin              "1.60e-02"           "2.17e-02"
## p_val_gee_blasto_dient_pos.clr      "3.43e-05"           "3.88e-03"
## p_val_gee_on_intervention1.clr      "0.00385"            "0.38113"
## p_val_time_23.clr                   "0.0438"             "0.6837"
## coef_gee_blasto_dient_pos.clr       "-0.866"             "-0.349"
## coef_gee_on_intervention1.clr       " 0.7255"            "-0.0947"
## coef_gee_time_23.clr                "-0.5139"            "-0.0418"
## p_val_gee_blastocystis_positive.clr "6.75e-04"           "3.49e-01"
## p_val_gee_dientamoeba_positive.clr  "2.89e-04"           "7.05e-04"
## tested_entities.clr                 " 50"                " 50"
## padj_gee_blasto_dient_pos.clr       "1.72e-03"           "1.94e-01"
## p_val_gee_blasto_dient_pos.lin      "6.60e-04"           "1.22e-01"
## p_val_gee_on_intervention1.lin      "0.572"              "0.824"
## p_val_time_23.lin                   "0.2978"             "0.2650"
## coef_gee_blasto_dient_pos.lin       "-147.06"            "  -5.14"
## coef_gee_on_intervention1.lin       " 39.029"            " -0.593"
## coef_gee_time_23.lin                "-61.77"             " -3.43"
## p_val_gee_blastocystis_positive.lin "2.79e-03"           "9.46e-01"
## p_val_gee_dientamoeba_positive.lin  "3.21e-04"           "4.35e-04"
## tested_entities.lin                 " 50"                " 50"
## padj_gee_blasto_dient_pos.lin       "0.03302"            "1.00000"
##                                     [,3]
## asv                                 "ASV00583"
## taxrank_name                        "Family"
## taxon_name                          "Carnobacteriaceae"
## note                                " "
## coef_gee_blastocystis_positive.clr  "-0.203"
## coef_gee_dientamoeba_positive.clr   "-0.311"
## padj_gee_blasto_pos.clr             "1.00e+00"
## padj_gee_dient_pos.clr              "1.41e-03"
## coef_gee_blastocystis_positive.lin  "  -2.812"
## coef_gee_dientamoeba_positive.lin   "  -3.46"
## padj_gee_blasto_pos.lin             "0.39788"
## padj_gee_dient_pos.lin              "1.29e-02"
## p_val_gee_blasto_dient_pos.clr      "8.60e-04"
## p_val_gee_on_intervention1.clr      "0.62203"
## p_val_time_23.clr                   "0.1552"
## coef_gee_blasto_dient_pos.clr       "-0.291"
## coef_gee_on_intervention1.clr       "-0.0459"
## coef_gee_time_23.clr                "-0.1230"
## p_val_gee_blastocystis_positive.clr "1.23e-01"
## p_val_gee_dientamoeba_positive.clr  "2.82e-05"
## tested_entities.clr                 " 50"
## padj_gee_blasto_dient_pos.clr       "4.30e-02"
## p_val_gee_blasto_dient_pos.lin      "4.86e-04"
## p_val_gee_on_intervention1.lin      "0.603"
## p_val_time_23.lin                   "0.0845"
## coef_gee_blasto_dient_pos.lin       "  -3.60"
```

```
## coef_gee_on_intervention1.lin       "  0.541"
## coef_gee_time_23.lin               " -3.13"
## p_val_gee_blastocystis_positive.lin "7.96e-03"
## p_val_gee_dientamoeba_positive.lin  "2.58e-04"
## tested_entities.lin                " 50"
## padj_gee_blasto_dient_pos.lin       "0.02429"
##                                     [,4]
## asv                                 "ASV00118"
## taxrank_name                        "Genus"
## taxon_name                          "[Ruminococcus] torques group"
## note                                " "
## coef_gee_blastocystis_positive.clr  "-0.219"
## coef_gee_dientamoeba_positive.clr   "-1.126"
## padj_gee_blasto_pos.clr             "1.00e+00"
## padj_gee_dient_pos.clr              "1.03e-08"
## coef_gee_blastocystis_positive.lin  " -25.721"
## coef_gee_dientamoeba_positive.lin   " -63.01"
## padj_gee_blasto_pos.lin             "1.00000"
## padj_gee_dient_pos.lin              "2.30e-05"
## p_val_gee_blasto_dient_pos.clr      "1.51e-04"
## p_val_gee_on_intervention1.clr      "0.42855"
## p_val_time_23.clr                   "0.6613"
## coef_gee_blasto_dient_pos.clr       "-0.733"
## coef_gee_on_intervention1.clr       "-0.1434"
## coef_gee_time_23.clr                "-0.0739"
## p_val_gee_blastocystis_positive.clr "4.31e-01"
## p_val_gee_dientamoeba_positive.clr  "7.43e-11"
## tested_entities.clr                 "139"
## padj_gee_blasto_dient_pos.clr       "2.10e-02"
## p_val_gee_blasto_dient_pos.lin      "3.26e-04"
## p_val_gee_on_intervention1.lin      "0.585"
## p_val_time_23.lin                   "0.7835"
## coef_gee_blasto_dient_pos.lin       " -49.98"
## coef_gee_on_intervention1.lin       "-12.380"
## coef_gee_time_23.lin                " -6.03"
## p_val_gee_blastocystis_positive.lin "1.15e-01"
## p_val_gee_dientamoeba_positive.lin  "1.65e-07"
## tested_entities.lin                 "139"
## padj_gee_blasto_dient_pos.lin       "0.04535"
##                                     [,5]            [,6]
## asv                                 "ASV00583"      "ASV00231"
## taxrank_name                        "Genus"         "Genus"
## taxon_name                          "Granulicatella" "Flavonifractor"
## note                                " "             " "
## coef_gee_blastocystis_positive.clr  "-0.233"        "-0.199"
## coef_gee_dientamoeba_positive.clr   "-0.237"        "-0.618"
## padj_gee_blasto_pos.clr             "1.00e+00"      "1.00e+00"
## padj_gee_dient_pos.clr              "4.72e-04"      "6.70e-06"
## coef_gee_blastocystis_positive.lin  "  -2.177"      "  -4.300"
## coef_gee_dientamoeba_positive.lin   "  -2.62"       " -12.27"
## padj_gee_blasto_pos.lin             "0.48715"       "1.00000"
## padj_gee_dient_pos.lin              "1.83e-02"      "4.81e-05"
## p_val_gee_blasto_dient_pos.clr      "4.73e-05"      "5.78e-05"
## p_val_gee_on_intervention1.clr      "0.65664"       "0.54040"
## p_val_time_23.clr                   "0.1976"        "0.1096"
## coef_gee_blasto_dient_pos.clr       "-0.254"        "-0.486"
## coef_gee_on_intervention1.clr       "-0.0293"       " 0.0750"
## coef_gee_time_23.clr                "-0.0831"       "-0.1557"
## p_val_gee_blastocystis_positive.clr "1.00e-02"      "1.45e-01"
## p_val_gee_dientamoeba_positive.clr  "3.40e-06"      "4.82e-08"
## tested_entities.clr                 "139"           "139"
## padj_gee_blasto_dient_pos.clr       "6.57e-03"      "8.04e-03"
## p_val_gee_blasto_dient_pos.lin      "4.75e-04"      "8.51e-05"
## p_val_gee_on_intervention1.lin      "0.819"         "0.790"
## p_val_time_23.lin                   "0.1226"        "0.3869"
## coef_gee_blasto_dient_pos.lin       "  -2.63"       "  -9.97"
## coef_gee_on_intervention1.lin       "  0.179"       " -0.825"
```

```
## coef_gee_time_23.lin               " -1.95"          " -2.00"
## p_val_gee_blastocystis_positive.lin "3.50e-03"       "8.89e-02"
## p_val_gee_dientamoeba_positive.lin "1.32e-04"        "3.46e-07"
## tested_entities.lin                "139"             "139"
## padj_gee_blasto_dient_pos.lin      "0.06609"         "0.01184"
##                                    [,7]
## asv                                "ASV00148"
## taxrank_name                       "Genus"
## taxon_name                         "UBA1819"
## note                               " "
## coef_gee_blastocystis_positive.clr  "-0.692"
## coef_gee_dientamoeba_positive.clr   "-0.449"
## padj_gee_blasto_pos.clr            "2.79e-08"
## padj_gee_dient_pos.clr             "6.08e-03"
## coef_gee_blastocystis_positive.lin  " -14.850"
## coef_gee_dientamoeba_positive.lin   " -12.72"
## padj_gee_blasto_pos.lin            "0.00283"
## padj_gee_dient_pos.lin             "8.79e-03"
## p_val_gee_blasto_dient_pos.clr     "6.06e-09"
## p_val_gee_on_intervention1.clr     "0.53480"
## p_val_time_23.clr                  "0.1827"
## coef_gee_blasto_dient_pos.clr      "-0.613"
## coef_gee_on_intervention1.clr      " 0.0794"
## coef_gee_time_23.clr               "-0.1533"
## p_val_gee_blastocystis_positive.clr "2.01e-10"
## p_val_gee_dientamoeba_positive.clr "4.38e-05"
## tested_entities.clr                "139"
## padj_gee_blasto_dient_pos.clr      "8.42e-07"
## p_val_gee_blasto_dient_pos.lin     "2.12e-05"
## p_val_gee_on_intervention1.lin     "0.344"
## p_val_time_23.lin                  "0.1493"
## coef_gee_blasto_dient_pos.lin      " -15.00"
## coef_gee_on_intervention1.lin      "  5.986"
## coef_gee_time_23.lin               " -4.58"
## p_val_gee_blastocystis_positive.lin "2.04e-05"
## p_val_gee_dientamoeba_positive.lin "6.32e-05"
## tested_entities.lin                "139"
## padj_gee_blasto_dient_pos.lin      "0.00295"
```

```
results_sign_blasto_clr <- results %>%
    filter( padj_gee_blasto_pos.clr < 0.05)
t(results_sign_blasto_clr)
```

```
##                                    [,1]            [,2]
## asv                                "ASV01987"      "ASV01487"
## taxrank_name                       "Class"         "Order"
## taxon_name                         "Fusobacteriia" "Flavobacteriales"
## note                               " "             " "
## coef_gee_blastocystis_positive.clr  "-0.178"        "-0.148"
## coef_gee_dientamoeba_positive.clr   "-0.02500"      " 0.08595"
## padj_gee_blasto_pos.clr            "6.29e-03"      "1.50e-02"
## padj_gee_dient_pos.clr             "1.00000"       "1.00000"
## coef_gee_blastocystis_positive.lin  "  -0.664"      "  -0.479"
## coef_gee_dientamoeba_positive.lin   "  -0.344"      "   4.201"
## padj_gee_blasto_pos.lin            "0.08703"       "1.00000"
## padj_gee_dient_pos.lin             "1.00000"       "1.00000"
## p_val_gee_blasto_dient_pos.clr     "5.02e-02"      "8.93e-01"
## p_val_gee_on_intervention1.clr     "0.47643"       "0.06639"
## p_val_time_23.clr                  "0.86911"       "0.00212"
## coef_gee_blasto_dient_pos.clr      "-0.0912"       " 0.0154"
## coef_gee_on_intervention1.clr      "-0.03647"      "-0.08811"
## coef_gee_time_23.clr               "-0.00911"      " 0.11321"
## p_val_gee_blastocystis_positive.clr "3.93e-04"      "4.55e-04"
## p_val_gee_dientamoeba_positive.clr "6.02e-01"      "5.59e-01"
## tested_entities.clr                " 16"           " 33"
## padj_gee_blasto_dient_pos.clr      "8.04e-01"      "1.00e+00"
## p_val_gee_blasto_dient_pos.lin     "1.19e-01"      "3.15e-01"
## p_val_gee_on_intervention1.lin     "0.7015"        "0.3308"
## p_val_time_23.lin                  "0.0858"        "0.3118"
```

```
## coef_gee_blasto_dient_pos.lin       "  -0.461"      "   3.199"
## coef_gee_on_intervention1.lin       "-0.10520"      "-2.06849"
## coef_gee_time_23.lin                " -0.6405"      "  2.1908"
## p_val_gee_blastocystis_positive.lin "5.44e-03"      "3.19e-01"
## p_val_gee_dientamoeba_positive.lin  "3.06e-01"      "3.11e-01"
## tested_entities.lin                 " 16"           " 33"
## padj_gee_blasto_dient_pos.lin       "1.00000"       "1.00000"
##                                     [,3]              [,4]
## asv                                 "ASV01987"        "ASV01337"
## taxrank_name                        "Order"           "Order"
## taxon_name                          "Fusobacteriales" "Chloroplast"
## note                                " "               " "
## coef_gee_blastocystis_positive.clr  "-0.211"          "-0.178"
## coef_gee_dientamoeba_positive.clr   "-0.12159"        "-0.11186"
## padj_gee_blasto_pos.clr             "9.02e-05"        "2.83e-03"
## padj_gee_dient_pos.clr              "0.25031"         "0.36332"
## coef_gee_blastocystis_positive.lin  "  -0.550"        "  -0.530"
## coef_gee_dientamoeba_positive.lin   "  -0.506"        "  -0.714"
## padj_gee_blasto_pos.lin             "0.78520"         "1.00000"
## padj_gee_dient_pos.lin              "0.57518"         "1.00000"
## p_val_gee_blasto_dient_pos.clr      "1.10e-04"        "1.83e-04"
## p_val_gee_on_intervention1.clr      "0.14375"         "0.14618"
## p_val_time_23.clr                   "0.63082"         "0.00767"
## coef_gee_blasto_dient_pos.clr       "-0.1701"         "-0.1562"
## coef_gee_on_intervention1.clr       "-0.07504"        "-0.07157"
## coef_gee_time_23.clr                " 0.02037"        " 0.10232"
## p_val_gee_blastocystis_positive.clr "2.73e-06"        "8.58e-05"
## p_val_gee_dientamoeba_positive.clr  "7.59e-03"        "1.10e-02"
## tested_entities.clr                 " 33"             " 33"
## padj_gee_blasto_dient_pos.clr       "3.64e-03"        "6.05e-03"
## p_val_gee_blasto_dient_pos.lin      "1.56e-02"        "9.74e-02"
## p_val_gee_on_intervention1.lin      "0.6013"          "0.3678"
## p_val_time_23.lin                   "0.3801"          "0.2616"
## coef_gee_blasto_dient_pos.lin       "  -0.573"        "  -0.766"
## coef_gee_on_intervention1.lin       "-0.19904"        "-1.00662"
## coef_gee_time_23.lin                " -0.2577"        "  1.1718"
## p_val_gee_blastocystis_positive.lin "2.38e-02"        "9.32e-02"
## p_val_gee_dientamoeba_positive.lin  "1.74e-02"        "1.30e-01"
## tested_entities.lin                 " 33"             " 33"
## padj_gee_blasto_dient_pos.lin       "0.51431"         "1.00000"
##                                     [,5]              [,6]
## asv                                 "ASV01546"        "ASV01883"
## taxrank_name                        "Order"           "Order"
## taxon_name                          "Actinomycetales" "Staphylococcales"
## note                                " "               " "
## coef_gee_blastocystis_positive.clr  "-0.172"          "-0.185"
## coef_gee_dientamoeba_positive.clr   "-0.13687"        "-0.14088"
## padj_gee_blasto_pos.clr             "8.89e-03"        "1.71e-02"
## padj_gee_dient_pos.clr              "0.04649"         "0.02339"
## coef_gee_blastocystis_positive.lin  "  -0.701"        "  -0.544"
## coef_gee_dientamoeba_positive.lin   "  -0.806"        "  -0.648"
## padj_gee_blasto_pos.lin             "1.00000"         "1.00000"
## padj_gee_dient_pos.lin              "1.00000"         "1.00000"
## p_val_gee_blasto_dient_pos.clr      "1.22e-05"        "2.09e-05"
## p_val_gee_on_intervention1.clr      "0.12398"         "0.09237"
## p_val_time_23.clr                   "0.66016"         "0.07936"
## coef_gee_blasto_dient_pos.clr       "-0.1779"         "-0.1719"
## coef_gee_on_intervention1.clr       "-0.07033"        "-0.07408"
## coef_gee_time_23.clr                " 0.02176"        " 0.06329"
## p_val_gee_blastocystis_positive.clr "2.69e-04"        "5.18e-04"
## p_val_gee_dientamoeba_positive.clr  "1.41e-03"        "7.09e-04"
## tested_entities.clr                 " 33"             " 33"
## padj_gee_blasto_dient_pos.clr       "4.02e-04"        "6.91e-04"
## p_val_gee_blasto_dient_pos.lin      "6.55e-02"        "6.47e-02"
## p_val_gee_on_intervention1.lin      "0.5825"          "0.6247"
## p_val_time_23.lin                   "0.2187"          "0.3789"
## coef_gee_blasto_dient_pos.lin       "  -0.875"        "  -0.633"
```

```
## coef_gee_on_intervention1.lin       " 0.16184"        " 0.12445"
## coef_gee_time_23.lin                " -1.1939"        " -0.5340"
## p_val_gee_blastocystis_positive.lin "1.40e-01"        "1.32e-01"
## p_val_gee_dientamoeba_positive.lin  "6.12e-02"        "3.58e-02"
## tested_entities.lin                 " 33"             " 33"
## padj_gee_blasto_dient_pos.lin       "1.00000"         "1.00000"
##                                     [,7]            [,8]
## asv                                 "ASV02228"      "ASV01987"
## taxrank_name                        "Order"         "Family"
## taxon_name                          "Eubacteriales" "Fusobacteriaceae"
## note                                " "             " "
## coef_gee_blastocystis_positive.clr  "-0.208"        "-0.220"
## coef_gee_dientamoeba_positive.clr   "-0.14501"      "-0.12677"
## padj_gee_blasto_pos.clr             "9.05e-06"      "5.43e-04"
## padj_gee_dient_pos.clr              "0.01607"       "0.58073"
## coef_gee_blastocystis_positive.lin  "  -0.830"      "  -0.569"
## coef_gee_dientamoeba_positive.lin   "  -0.796"      "  -0.482"
## padj_gee_blasto_pos.lin             "1.00000"       "0.07228"
## padj_gee_dient_pos.lin              "0.74928"       "0.80985"
## p_val_gee_blasto_dient_pos.clr      "9.67e-07"      "4.27e-04"
## p_val_gee_on_intervention1.clr      "0.72074"       "0.34297"
## p_val_time_23.clr                   "0.46812"       "0.38901"
## coef_gee_blasto_dient_pos.clr       "-0.1877"       "-0.1667"
## coef_gee_on_intervention1.clr       "-0.01867"      "-0.04056"
## coef_gee_time_23.clr                " 0.02957"      "-0.04101"
## p_val_gee_blastocystis_positive.clr "2.74e-07"      "1.09e-05"
## p_val_gee_dientamoeba_positive.clr  "4.87e-04"      "1.16e-02"
## tested_entities.clr                 " 33"           " 50"
## padj_gee_blasto_dient_pos.clr       "3.19e-05"      "2.14e-02"
## p_val_gee_blasto_dient_pos.lin      "2.69e-02"      "6.38e-03"
## p_val_gee_on_intervention1.lin      "0.2530"        "0.0753"
## p_val_time_23.lin                   "0.1263"        "0.2053"
## coef_gee_blasto_dient_pos.lin       "  -0.890"      "  -0.548"
## coef_gee_on_intervention1.lin       " 0.94574"      "-0.39823"
## coef_gee_time_23.lin                " -0.4806"      " -0.4022"
## p_val_gee_blastocystis_positive.lin "3.63e-02"      "1.45e-03"
## p_val_gee_dientamoeba_positive.lin  "2.27e-02"      "1.62e-02"
## tested_entities.lin                 " 33"           " 50"
## padj_gee_blasto_dient_pos.lin       "0.88882"       "0.31906"
##                                     [,9]                 [,10]
## asv                                 "ASV00041"           "ASV00166"
## taxrank_name                        "Family"             "Genus"
## taxon_name                          "Enterobacteriaceae" "Fusicatenibacter"
## note                                " "                  " "
## coef_gee_blastocystis_positive.clr  "-0.907"             " 0.761"
## coef_gee_dientamoeba_positive.clr   "-0.83739"           " 0.08481"
## padj_gee_blasto_pos.clr             "3.37e-02"           "1.70e-02"
## padj_gee_dient_pos.clr              "0.01444"            "1.00000"
## coef_gee_blastocystis_positive.lin  "-131.995"           "  25.282"
## coef_gee_dientamoeba_positive.lin   "-135.582"           "   1.103"
## padj_gee_blasto_pos.lin             "0.13938"            "1.00000"
## padj_gee_dient_pos.lin              "0.01604"            "1.00000"
## p_val_gee_blasto_dient_pos.clr      "3.43e-05"           "5.23e-02"
## p_val_gee_on_intervention1.clr      "0.00385"            "0.39760"
## p_val_time_23.clr                   "0.04376"            "0.74523"
## coef_gee_blasto_dient_pos.clr       "-0.8656"            " 0.3078"
## coef_gee_on_intervention1.clr       " 0.72553"           " 0.10691"
## coef_gee_time_23.clr                "-0.51394"           "-0.03498"
## p_val_gee_blastocystis_positive.clr "6.75e-04"           "1.23e-04"
## p_val_gee_dientamoeba_positive.clr  "2.89e-04"           "5.81e-01"
## tested_entities.clr                 " 50"                "139"
## padj_gee_blasto_dient_pos.clr       "1.72e-03"           "1.00e+00"
## p_val_gee_blasto_dient_pos.lin      "6.60e-04"           "7.50e-02"
## p_val_gee_on_intervention1.lin      "0.5716"             "0.9462"
## p_val_time_23.lin                   "0.2978"             "0.6388"
## coef_gee_blasto_dient_pos.lin       "-147.065"           "   9.885"
## coef_gee_on_intervention1.lin       "39.02855"           " 0.34242"
```

```
## coef_gee_time_23.lin                "-61.7678"           "  1.9044"
## p_val_gee_blastocystis_positive.lin "2.79e-03"           "9.09e-03"
## p_val_gee_dientamoeba_positive.lin  "3.21e-04"           "7.82e-01"
## tested_entities.lin                 " 50"                "139"
## padj_gee_blasto_dient_pos.lin       "0.03302"            "1.00000"
##                                     [,11]
## asv                                 "ASV00975"
## taxrank_name                        "Genus"
## taxon_name                          "[Eubacterium] fissicatena group"
## note                                " "
## coef_gee_blastocystis_positive.clr  "-0.195"
## coef_gee_dientamoeba_positive.clr   "-0.09032"
## padj_gee_blasto_pos.clr             "1.04e-03"
## padj_gee_dient_pos.clr              "1.00000"
## coef_gee_blastocystis_positive.lin  "  -0.553"
## coef_gee_dientamoeba_positive.lin   "  -0.568"
## padj_gee_blasto_pos.lin             "0.44518"
## padj_gee_dient_pos.lin              "0.04153"
## p_val_gee_blasto_dient_pos.clr      "1.45e-04"
## p_val_gee_on_intervention1.clr      "0.80381"
## p_val_time_23.clr                   "0.12771"
## coef_gee_blasto_dient_pos.clr       "-0.1401"
## coef_gee_on_intervention1.clr       " 0.01143"
## coef_gee_time_23.clr                " 0.05157"
## p_val_gee_blastocystis_positive.clr "7.46e-06"
## p_val_gee_dientamoeba_positive.clr  "1.03e-02"
## tested_entities.clr                 "139"
## padj_gee_blasto_dient_pos.clr       "2.01e-02"
## p_val_gee_blasto_dient_pos.lin      "7.42e-04"
## p_val_gee_on_intervention1.lin      "0.7420"
## p_val_time_23.lin                   "0.4705"
## coef_gee_blasto_dient_pos.lin       "  -0.591"
## coef_gee_on_intervention1.lin       " 0.10229"
## coef_gee_time_23.lin                "  0.1768"
## p_val_gee_blastocystis_positive.lin "3.20e-03"
## p_val_gee_dientamoeba_positive.lin  "2.99e-04"
## tested_entities.lin                 "139"
## padj_gee_blasto_dient_pos.lin       "0.10317"
##                                     [,12]
## asv                                 "ASV00361"
## taxrank_name                        "Genus"
## taxon_name                          "Lachnospiraceae ND3007 group"
## note                                " "
## coef_gee_blastocystis_positive.clr  " 0.886"
## coef_gee_dientamoeba_positive.clr   "-0.00175"
## padj_gee_blasto_pos.clr             "9.23e-03"
## padj_gee_dient_pos.clr              "1.00000"
## coef_gee_blastocystis_positive.lin  "  20.303"
## coef_gee_dientamoeba_positive.lin   "  -1.467"
## padj_gee_blasto_pos.lin             "1.00000"
## padj_gee_dient_pos.lin              "1.00000"
## p_val_gee_blasto_dient_pos.clr      "4.18e-02"
## p_val_gee_on_intervention1.clr      "0.59290"
## p_val_time_23.clr                   "0.19800"
## coef_gee_blasto_dient_pos.clr       " 0.2929"
## coef_gee_on_intervention1.clr       "-0.04765"
## coef_gee_time_23.clr                "-0.10650"
## p_val_gee_blastocystis_positive.clr "6.64e-05"
## p_val_gee_dientamoeba_positive.clr  "9.89e-01"
## tested_entities.clr                 "139"
## padj_gee_blasto_dient_pos.clr       "1.00e+00"
## p_val_gee_blasto_dient_pos.lin      "8.58e-02"
## p_val_gee_on_intervention1.lin      "0.2199"
## p_val_time_23.lin                   "0.2429"
## coef_gee_blasto_dient_pos.lin       "   6.501"
## coef_gee_on_intervention1.lin       "-2.33835"
## coef_gee_time_23.lin                " -2.1004"
```

```
## p_val_gee_blastocystis_positive.lin "1.45e-02"
## p_val_gee_dientamoeba_positive.lin  "4.52e-01"
## tested_entities.lin                 "139"
## padj_gee_blasto_dient_pos.lin       "1.00000"
##                                     [,13]           [,14]
## asv                                 "ASV01987"      "ASV00041"
## taxrank_name                        "Genus"         "Genus"
## taxon_name                          "Fusobacterium" "Escherichia-Shigella"
## note                                " "             " "
## coef_gee_blastocystis_positive.clr  "-0.155"        "-1.160"
## coef_gee_dientamoeba_positive.clr   "-0.05688"      "-0.83202"
## padj_gee_blasto_pos.clr             "1.67e-02"      "1.18e-06"
## padj_gee_dient_pos.clr              "1.00000"       "0.01330"
## coef_gee_blastocystis_positive.lin  "  -0.315"      "-106.939"
## coef_gee_dientamoeba_positive.lin   "  -0.294"      " -86.524"
## padj_gee_blasto_pos.lin             "0.30059"       "0.17385"
## padj_gee_dient_pos.lin              "1.00000"       "0.05006"
## p_val_gee_blasto_dient_pos.clr      "7.22e-04"      "1.34e-07"
## p_val_gee_on_intervention1.clr      "0.15432"       "0.03070"
## p_val_time_23.clr                   "0.69708"       "0.09584"
## coef_gee_blasto_dient_pos.clr       "-0.1043"       "-1.0421"
## coef_gee_on_intervention1.clr       "-0.03906"      " 0.53002"
## coef_gee_time_23.clr                "-0.01085"      "-0.40974"
## p_val_gee_blastocystis_positive.clr "1.20e-04"      "8.49e-09"
## p_val_gee_dientamoeba_positive.clr  "4.47e-02"      "9.57e-05"
## tested_entities.clr                 "139"           "139"
## padj_gee_blasto_dient_pos.clr       "1.00e-01"      "1.87e-05"
## p_val_gee_blasto_dient_pos.lin      "5.76e-03"      "4.62e-04"
## p_val_gee_on_intervention1.lin      "0.0773"        "0.8891"
## p_val_time_23.lin                   "0.4986"        "0.4347"
## coef_gee_blasto_dient_pos.lin       "  -0.329"      "-104.976"
## coef_gee_on_intervention1.lin       "-0.30289"      " 6.07656"
## coef_gee_time_23.lin                " -0.1480"      "-32.2783"
## p_val_gee_blastocystis_positive.lin "2.16e-03"      "1.25e-03"
## p_val_gee_dientamoeba_positive.lin  "1.26e-02"      "3.60e-04"
## tested_entities.lin                 "139"           "139"
## padj_gee_blasto_dient_pos.lin       "0.80101"       "0.06424"
##                                     [,15]         [,16]        [,17]
## asv                                 "ASV01546"    "ASV00371"   "ASV00788"
## taxrank_name                        "Genus"       "Genus"      "Genus"
## taxon_name                          "Actinomyces" "Holdemania" "Dielma"
## note                                " "           " "          " "
## coef_gee_blastocystis_positive.clr  "-0.164"      "-0.399"     "-0.270"
## coef_gee_dientamoeba_positive.clr   "-0.07389"    "-0.23680"   "-0.09642"
## padj_gee_blasto_pos.clr             "2.93e-02"    "2.23e-02"   "1.71e-02"
## padj_gee_dient_pos.clr              "1.00000"     "1.00000"    "1.00000"
## coef_gee_blastocystis_positive.lin  "  -0.578"    "  -4.777"   "  -1.274"
## coef_gee_dientamoeba_positive.lin   "  -0.583"    "  -3.863"   "  -1.012"
## padj_gee_blasto_pos.lin             "1.00000"     "0.50481"    "0.26528"
## padj_gee_dient_pos.lin              "1.00000"     "1.00000"    "1.00000"
## p_val_gee_blasto_dient_pos.clr      "7.92e-05"    "3.53e-03"   "9.34e-04"
## p_val_gee_on_intervention1.clr      "0.65048"     "0.60154"    "0.85579"
## p_val_time_23.clr                   "0.89028"     "0.15886"    "0.82247"
## coef_gee_blasto_dient_pos.clr       "-0.1300"     "-0.3327"    "-0.1812"
## coef_gee_on_intervention1.clr       "-0.01557"    " 0.04962"   " 0.01146"
## coef_gee_time_23.clr                "-0.00576"    "-0.13211"   " 0.01396"
## p_val_gee_blastocystis_positive.clr "2.11e-04"    "1.61e-04"   "1.23e-04"
## p_val_gee_dientamoeba_positive.clr  "1.15e-02"    "6.45e-02"   "3.79e-02"
## tested_entities.clr                 "139"         "139"        "139"
## padj_gee_blasto_dient_pos.clr       "1.10e-02"    "4.91e-01"   "1.30e-01"
## p_val_gee_blasto_dient_pos.lin      "4.48e-02"    "1.03e-02"   "2.47e-03"
## p_val_gee_on_intervention1.lin      "0.6231"      "0.9170"     "0.7016"
## p_val_time_23.lin                   "0.2963"      "0.0559"     "0.9139"
## coef_gee_blasto_dient_pos.lin       "  -0.655"    "  -4.671"   "  -1.213"
## coef_gee_on_intervention1.lin       " 0.12132"    " 0.12988"   "-0.26049"
## coef_gee_time_23.lin                " -0.6946"    " -2.5687"   "  0.0845"
## p_val_gee_blastocystis_positive.lin "7.07e-02"    "3.63e-03"   "1.91e-03"
```

```
## p_val_gee_dientamoeba_positive.lin  "4.74e-02"    "5.71e-02"   "7.55e-03"
## tested_entities.lin                 "139"         "139"        "139"
## padj_gee_blasto_dient_pos.lin       "1.00000"     "1.00000"    "0.34378"
##                                     [,18]           [,19]      [,20]
## asv                                 "ASV00880"      "ASV01883" "ASV02228"
## taxrank_name                        "Genus"         "Genus"    "Genus"
## taxon_name                          "Coprobacillus" "Gemella"  "Anaerofustis"
## note                                " "             " "        " "
## coef_gee_blastocystis_positive.clr  "-0.162"        "-0.161"   "-0.144"
## coef_gee_dientamoeba_positive.clr   "-0.08697"      "-0.08427" "-0.05874"
## padj_gee_blasto_pos.clr             "5.93e-03"      "1.66e-02" "2.54e-02"
## padj_gee_dient_pos.clr              "1.00000"       "0.27580"  "1.00000"
## coef_gee_blastocystis_positive.lin  "  -0.642"      "  -0.383" "  -0.269"
## coef_gee_dientamoeba_positive.lin   "  -0.747"      "  -0.505" "  -0.305"
## padj_gee_blasto_pos.lin             "1.00000"       "1.00000"  "1.00000"
## padj_gee_dient_pos.lin              "1.00000"       "0.85478"  "0.73681"
## p_val_gee_blasto_dient_pos.clr      "5.35e-04"      "2.03e-05" "1.74e-04"
## p_val_gee_on_intervention1.clr      "0.88981"       "0.62252"  "0.68869"
## p_val_time_23.clr                   "0.93610"       "0.69745"  "0.11900"
## coef_gee_blasto_dient_pos.clr       "-0.1249"       "-0.1290"  "-0.1056"
## coef_gee_on_intervention1.clr       " 0.00656"      "-0.01454" " 0.01038"
## coef_gee_time_23.clr                "-0.00381"      "-0.01338" "-0.04160"
## p_val_gee_blastocystis_positive.clr "4.27e-05"      "1.19e-04" "1.82e-04"
## p_val_gee_dientamoeba_positive.clr  "1.29e-02"      "1.98e-03" "2.13e-02"
## tested_entities.clr                 "139"           "139"      "139"
## padj_gee_blasto_dient_pos.clr       "7.44e-02"      "2.82e-03" "2.41e-02"
## p_val_gee_blasto_dient_pos.lin      "5.15e-02"      "1.94e-02" "6.52e-03"
## p_val_gee_on_intervention1.lin      "0.8570"        "0.9700"   "0.0709"
## p_val_time_23.lin                   "0.7159"        "0.2169"   "0.0319"
## coef_gee_blasto_dient_pos.lin       "  -0.742"      "  -0.489" "  -0.328"
## coef_gee_on_intervention1.lin       "-0.09780"      " 0.00479" " 0.16616"
## coef_gee_time_23.lin                " -0.2639"      " -0.4666" " -0.5236"
## p_val_gee_blastocystis_positive.lin "7.63e-02"      "8.51e-02" "3.09e-02"
## p_val_gee_dientamoeba_positive.lin  "3.72e-02"      "6.15e-03" "5.30e-03"
## tested_entities.lin                 "139"           "139"      "139"
## padj_gee_blasto_dient_pos.lin       "1.00000"       "1.00000"  "0.90585"
##                                     [,21]      [,22]        [,23]
## asv                                 "ASV00524" "ASV00876"   "ASV00148"
## taxrank_name                        "Genus"    "Genus"      "Genus"
## taxon_name                          "DTU089"   "Paludicola" "UBA1819"
## note                                " "        " "          " "
## coef_gee_blastocystis_positive.clr  "-0.238"   "-0.228"     "-0.692"
## coef_gee_dientamoeba_positive.clr   "-0.08960" " 0.03209"   "-0.44891"
## padj_gee_blasto_pos.clr             "9.08e-03" "4.72e-03"   "2.79e-08"
## padj_gee_dient_pos.clr              "1.00000"  "1.00000"    "0.00608"
## coef_gee_blastocystis_positive.lin  "  -1.786" "  -1.142"   " -14.850"
## coef_gee_dientamoeba_positive.lin   "  -0.744" "   0.225"   " -12.725"
## padj_gee_blasto_pos.lin             "1.00000"  "0.15064"    "0.00283"
## padj_gee_dient_pos.lin              "1.00000"  "1.00000"    "0.00879"
## p_val_gee_blasto_dient_pos.clr      "4.61e-03" "2.49e-01"   "6.06e-09"
## p_val_gee_on_intervention1.clr      "0.58618"  "0.47898"    "0.53480"
## p_val_time_23.clr                   "0.83985"  "0.90509"    "0.18272"
## coef_gee_blasto_dient_pos.clr       "-0.1637"  "-0.0681"    "-0.6129"
## coef_gee_on_intervention1.clr       "-0.04415" " 0.04451"   " 0.07945"
## coef_gee_time_23.clr                " 0.01280" "-0.00639"   "-0.15330"
## p_val_gee_blastocystis_positive.clr "6.53e-05" "3.39e-05"   "2.01e-10"
## p_val_gee_dientamoeba_positive.clr  "1.62e-01" "6.04e-01"   "4.38e-05"
## tested_entities.clr                 "139"      "139"        "139"
## padj_gee_blasto_dient_pos.clr       "6.40e-01" "1.00e+00"   "8.42e-07"
## p_val_gee_blasto_dient_pos.lin      "2.36e-01" "6.16e-01"   "2.12e-05"
## p_val_gee_on_intervention1.lin      "0.7133"   "0.4328"     "0.3442"
## p_val_time_23.lin                   "0.6286"   "0.6236"     "0.1493"
## coef_gee_blasto_dient_pos.lin       "  -1.138" "  -0.252"   " -15.003"
## coef_gee_on_intervention1.lin       " 0.61644" " 0.45836"   " 5.98621"
## coef_gee_time_23.lin                " -0.3945" " -0.2780"   " -4.5839"
## p_val_gee_blastocystis_positive.lin "2.25e-02" "1.08e-03"   "2.04e-05"
## p_val_gee_dientamoeba_positive.lin  "5.03e-01" "7.08e-01"   "6.32e-05"
```

```
## tested_entities.lin                 "139"      "139"        "139"
## padj_gee_blasto_dient_pos.lin       "1.00000"  "1.00000"    "0.00295"
##                                     [,24]
## asv                                 "ASV01513"
## taxrank_name                        "Genus"
## taxon_name                          "Candidatus Soleaferrea"
## note                                " "
## coef_gee_blastocystis_positive.clr  "-0.158"
## coef_gee_dientamoeba_positive.clr   "-0.06059"
## padj_gee_blasto_pos.clr             "2.32e-02"
## padj_gee_dient_pos.clr              "1.00000"
## coef_gee_blastocystis_positive.lin  "  -0.326"
## coef_gee_dientamoeba_positive.lin   "  -0.240"
## padj_gee_blasto_pos.lin             "1.00000"
## padj_gee_dient_pos.lin              "1.00000"
## p_val_gee_blasto_dient_pos.clr      "3.70e-04"
## p_val_gee_on_intervention1.clr      "0.34073"
## p_val_time_23.clr                   "0.23099"
## coef_gee_blasto_dient_pos.clr       "-0.1081"
## coef_gee_on_intervention1.clr       " 0.02920"
## coef_gee_time_23.clr                "-0.04097"
## p_val_gee_blastocystis_positive.clr "1.67e-04"
## p_val_gee_dientamoeba_positive.clr  "2.30e-02"
## tested_entities.clr                 "139"
## padj_gee_blasto_dient_pos.clr       "5.14e-02"
## p_val_gee_blasto_dient_pos.lin      "6.66e-02"
## p_val_gee_on_intervention1.lin      "0.1380"
## p_val_time_23.lin                   "0.0533"
## coef_gee_blasto_dient_pos.lin       "  -0.287"
## coef_gee_on_intervention1.lin       " 0.23970"
## coef_gee_time_23.lin                " -0.4293"
## p_val_gee_blastocystis_positive.lin "3.04e-02"
## p_val_gee_dientamoeba_positive.lin  "1.60e-01"
## tested_entities.lin                 "139"
## padj_gee_blasto_dient_pos.lin       "1.00000"
```

```
pa_graph(PSMR_O, Facet  = "Order", x_cat = "blastocystis_or_dientamoeba", my_selection_tax_level = "Order",
       my_taxa = c("Flavobacteriales", "Eubacteriales", "Verrucomicrobiales")
       )
```

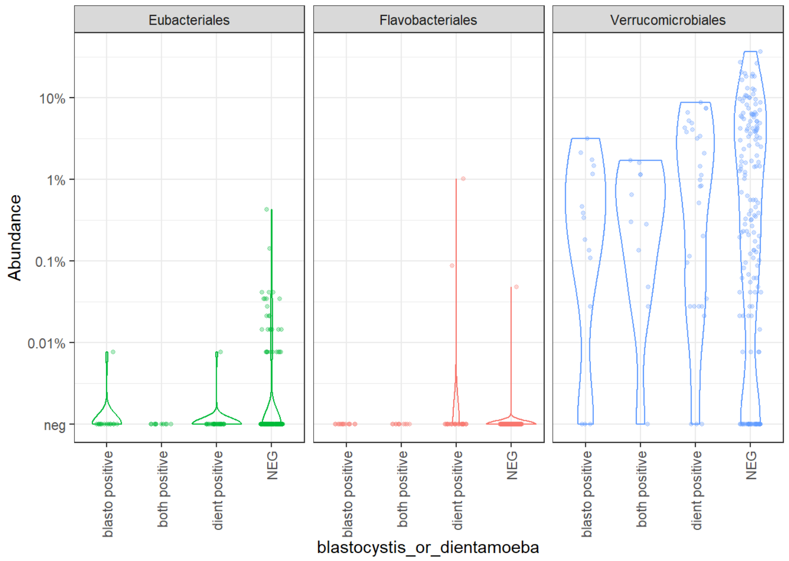


```
otu_table(PSMR_O)[,which(tax_table(PSMR_O)[, "Order"]%in% c("Flavobacteriales", "Eubacteriales", "Verrucomicrobiales"))]
```

```
## OTU Table:          [3 taxa and 227 samples]
##                      taxa are columns
##     ASV01487 ASV00024 ASV02228
## 10A        0       16        0
## 10B        0       50        0
## 10C        0        4        0
## 11A        0      631        6
## 11B        0     3057       64
```

```
## 11C       0    250      0
## 12A       0      0      0
## 12B       0      0      0
## 12C       0      0      0
## 13A       0    919      0
## 13B       0   1488      2
## 13C       0   5485      0
## 14A       0     33      2
## 14B       0    103      0
## 14C       0    727      2
## 15A       0      0      0
## 15B       0      0      0
## 15C       0      0      0
## 16A       0    897      0
## 16B       0    973      0
## 16C       0      1      0
## 17A       0     69      0
## 17B       0    955      0
## 17C       0      7      0
## 18A       0     20      0
## 18B       0      0      0
## 18C       0    242      0
## 19A       0    219      1
## 19B       0    315      0
## 19C       0    257      0
## 1A        0   1437      1
## 1B        0    628      0
## 1C        0    582      0
## 20A       0      4      0
## 20B       0     14      0
## 20C       0    124      0
## 21A       0      4      0
## 21B       0      4      0
## 21C       0     18      0
## 22A       0     17      0
## 22B       0    564      0
## 22C       0    167      0
## 23A       0     34      0
## 23B       0     49      1
## 23C       0      9      0
## 24A       0      0      0
## 24B       0      0      3
## 24C       0      4      1
## 25A       0      1      0
## 25B       0      0      0
## 25C       0     15      0
## 26A       0      0      0
## 26B       0      0      0
## 26C       0      0      0
## 27A       0    865      0
## 27B       0    211      2
## 27C       0   1946      0
## 28A       0      0      5
## 28B       0    611      0
## 28C       0    494      0
## 29A       0    215      0
## 29B       0    215      0
## 29C       0      0      0
## 2A        0     47      6
## 2B        0    169      0
## 2C        0     30      0
## 30A       0    247      0
## 30B       0      3      0
## 30C       0    819      0
## 31A       0   3941      0
## 31B       0     68      0
## 31C       0      0      0
```

```
## 32A       0      0      0
## 32B       7      3      0
## 32C       0      0      0
## 33A       0      0      0
## 33B       0      0      0
## 33C       0      0      0
## 34A       0   2927      0
## 34B       0   1235      0
## 34C       0     62      0
## 35A       0    170      0
## 35B       0     42      0
## 35C       0     96      0
## 36A       0      3      0
## 36B       0      6      1
## 36C       0      4      0
## 37A       0    681      0
## 37B       0   4048      0
## 37C       0   1052      0
## 38A       0    199      0
## 38B       0    673      0
## 38C       0    868      0
## 39A       0    575      0
## 39B       0    290      2
## 39C       0    459      0
## 3A        0    987      0
## 3B        0   2701      5
## 3C        0   2736      0
## 41A       0      0      0
## 41B       0      0      0
## 41C       0      0      1
## 42A       0    768      0
## 42B       0   1412      0
## 42C       0   1503      1
## 43A       0      0      0
## 43B       0      0      0
## 43C       0      0      0
## 44A       0     56      1
## 44B       0     48      0
## 44C       0     42      5
## 45A       0      0     21
## 45B       0    217      0
## 45C       0   1926      0
## 46A       0    387      1
## 46B       0     76      0
## 46C       0     19      3
## 47A       0    182      0
## 47B       0    919      0
## 47C       0     14      0
## 48A       0      8      0
## 48B       0     34      0
## 48C       0      2      0
## 49A       0    613      0
## 49B       0      0      0
## 49C       0     54      2
## 4A        0    397      0
## 4B        0      0      0
## 4C        0      0      0
## 50A       0   2465      1
## 50B       0   1504      0
## 50C       0    929      0
## 51A       0      6      3
## 51B       0      1      0
## 51C       0     22      0
## 52A       0    537      0
## 52B       0     29      0
## 52C       0     83      1
## 53A       0    286      0
```

```
## 53B        0     505        0
## 53C        0       1        0
## 54A       13     146        0
## 54B      153     780        0
## 54C        0     939        0
## 55A        0     986        0
## 55B        0     472        0
## 55C        0     611        0
## 56A        0    1309        0
## 56B        0    1858        0
## 56C        0       0        0
## 57A        0       3        0
## 57B        0    1099        0
## 57C        0       4        0
## 58A        0      20        0
## 58B        0      94        0
## 58C        0      20        0
## 59A        0     239        0
## 59B        0       0        0
## 59C        0       7        0
## 5A         0       0        0
## 5B         0      58        0
## 5C         0     347        0
## 60A        0     171        0
## 60B        0      45        0
## 60C        0      20        0
## 61A        0      83        0
## 61B        0      10        0
## 61C        0      12        0
## 62A        0       0        0
## 62B        0       0        0
## 62C        0       6        0
## 63A        0    1609        0
## 63B        0     769        0
## 63C        0     448        0
## 64A        0     238        0
## 64B        0     187        0
## 64C        0      58        0
## 65A        0     768        0
## 65B        0      83        0
## 65C        0    1638        0
## 66A        0       6        0
## 66B        0       5        0
## 66C        0      40        0
## 67A        0       0        0
## 67B        0     151        5
## 67C        0      31        0
## 68A        0     477        2
## 68B        0    1582        0
## 68C        0    1348        0
## 69A        0    2734        0
## 69B        0    2956        0
## 69C        0    1106        0
## 6A         0      26        0
## 6B         0       0        0
## 6C         0       9        0
## 70A        0     640        0
## 70B        0      16        0
## 70C        0       0        0
## 71A        0       9        1
## 71C        0       0        0
## 72A        0      13        0
## 72B        0      33        0
## 73A        0       0        4
## 73B        0     563        6
## 74A        0     254        0
## 74B        0     472        0
```

```
## 75A       0      507       0
## 75B       0     1097       0
## 76A       0      173       0
## 76C       0       27       0
## 77B       0        3       0
## 77C       0        4       0
## 78A       0       76       0
## 78B       0      311       0
## 78C       0       30       0
## 79A       0        0       0
## 79B       0       52       0
## 79C       0        0       0
## 7A        0        0       0
## 7B        0      372       0
## 7C        0     1226       0
## 8A        0      732       0
## 8B        0     1314       0
## 8C        0      122       0
## 9A        0      635       0
## 9B        0      214       0
## 9C        0      589       0
```

Session info

```
sessionInfo()
```

```
## R version 4.1.2 (2021-11-01)
## Platform: x86_64-w64-mingw32/x64 (64-bit)
## Running under: Windows 10 x64 (build 22621)
##
## Matrix products: default
##
## locale:
## [1] LC_COLLATE=English_World.1252  LC_CTYPE=English_World.1252
## [3] LC_MONETARY=English_World.1252 LC_NUMERIC=C
## [5] LC_TIME=English_World.1252
##
## attached base packages:
##  [1] tcltk     parallel  grid      stats4    stats     graphics  grDevices
##  [8] utils     datasets  methods   base
##
## other attached packages:
##  [1] BiodiversityR_2.14-1        ggord_1.1.7
##  [3] xlsx_0.6.5                  forcats_0.5.1
##  [5] dplyr_1.1.2                 purrr_1.0.1
##  [7] readr_2.1.2                 tidyr_1.3.0
##  [9] tibble_3.2.1                tidyverse_1.3.1
## [11] biomformat_1.22.0           foreach_1.5.2
## [13] waffle_0.7.0                stringr_1.5.0
## [15] ROCR_1.0-11                 LiblineaR_2.10-12
## [17] gplots_3.1.1                ggvegan_0.1-0
## [19] vegan_2.5-7                 permute_0.9-7
## [21] xtable_1.8-4                RColorBrewer_1.1-3
## [23] gridExtra_2.3               Polychrome_1.5.1
## [25] pals_1.8                    pheatmap_1.0.12
## [27] psych_2.1.9                 lattice_0.20-45
## [29] cowplot_1.1.1               data.table_1.14.2
## [31] beeswarm_0.4.0              ggsci_3.0.0
## [33] ape_5.6-1                   DESeq2_1.34.0
## [35] SummarizedExperiment_1.24.0 Biobase_2.54.0
## [37] MatrixGenerics_1.6.0        matrixStats_0.61.0
## [39] GenomicRanges_1.46.1        scales_1.2.1
## [41] ggplot2_3.4.0               RDPutils_1.4.1
## [43] Biostrings_2.62.0           GenomeInfoDb_1.30.1
## [45] XVector_0.34.0              IRanges_2.28.0
## [47] S4Vectors_0.32.3            BiocGenerics_0.40.0
## [49] reshape2_1.4.4              phyloseq_1.38.0
##
## loaded via a namespace (and not attached):
```

```
##   [1] utf8_1.2.2              lme4_1.1-28             tidyselect_1.2.0
##   [4] htmlwidgets_1.5.4       RSQLite_2.2.10          AnnotationDbi_1.56.2
##   [7] BiocParallel_1.28.3     Rtsne_0.16              munsell_0.5.0
##  [10] codetools_0.2-18        withr_2.5.0             colorspace_2.0-2
##  [13] highr_0.9               knitr_1.37              rstudioapi_0.13
##  [16] rJava_1.0-6             Rttf2pt1_1.3.10         labeling_0.4.2
##  [19] GenomeInfoDbData_1.2.7 mnormt_2.0.2            bit64_4.0.5
##  [22] farver_2.1.0            rhdf5_2.38.0            vctrs_0.6.1
##  [25] generics_0.1.3          xfun_0.29               geepack_1.3.3
##  [28] R6_2.5.1                locfit_1.5-9.4          microbiome_1.16.0
##  [31] bitops_1.0-7            rhdf5filters_1.6.0      cachem_1.0.6
##  [34] DelayedArray_0.20.0     assertthat_0.2.1        nnet_7.3-16
##  [37] gtable_0.3.1            sandwich_3.0-1          rlang_1.1.0
##  [40] genefilter_1.76.0       systemfonts_1.0.4       scatterplot3d_0.3-41
##  [43] splines_4.1.2           extrafontdb_1.0         dichromat_2.0-0.1
##  [46] checkmate_2.0.0         broom_1.0.1             abind_1.4-5
##  [49] yaml_2.2.2              modelr_0.1.8            backports_1.4.1
##  [52] Hmisc_4.6-0             extrafont_0.17          tools_4.1.2
##  [55] Rcmdr_2.7-2             ellipsis_0.3.2          jquerylib_0.1.4
##  [58] proxy_0.4-26            Rcpp_1.0.9              plyr_1.8.8
##  [61] base64enc_0.1-3         zlibbioc_1.40.0         RCurl_1.98-1.6
##  [64] rpart_4.1-15            zoo_1.8-9               haven_2.4.3
##  [67] ggrepel_0.9.2           cluster_2.1.2           survey_4.1-1
##  [70] fs_1.5.2                magrittr_2.0.2          reprex_2.0.1
##  [73] effects_4.2-1           tmvnsim_1.0-2           hms_1.1.1
##  [76] xlsxjars_0.6.1          RcmdrMisc_2.7-2         evaluate_0.15
##  [79] XML_3.99-0.8            jpeg_0.1-9              readxl_1.3.1
##  [82] compiler_4.1.2          maps_3.4.1              KernSmooth_2.23-20
##  [85] crayon_1.5.0            minqa_1.2.4             htmltools_0.5.2
##  [88] mgcv_1.8-38             tzdb_0.2.0              Formula_1.2-4
##  [91] geneplotter_1.72.0      lubridate_1.8.0         DBI_1.1.2
##  [94] dbplyr_2.1.1            MASS_7.3-54             relimp_1.0-5
##  [97] boot_1.3-28             car_3.1-1               Matrix_1.5-4
## [100] ade4_1.7-18             mitools_2.4             cli_3.6.1
## [103] insight_0.16.0          igraph_1.2.11           pkgconfig_2.0.3
## [106] foreign_0.8-81          xml2_1.3.3              svglite_2.1.0
## [109] annotate_1.72.0         bslib_0.4.1             multtest_2.50.0
## [112] rvest_1.0.2             digest_0.6.29           rmarkdown_2.11
## [115] cellranger_1.1.0        htmlTable_2.4.0         nortest_1.0-4
## [118] gtools_3.9.2            nloptr_2.0.0            lifecycle_1.0.3
## [121] nlme_3.1-153            jsonlite_1.7.3          Rhdf5lib_1.16.0
## [124] carData_3.0-5           mapproj_1.2.11          fansi_1.0.2
## [127] pillar_1.9.0            KEGGREST_1.34.0         fastmap_1.1.0
## [130] httr_1.4.2              survival_3.2-13         glue_1.6.1
## [133] png_0.1-7               iterators_1.0.14        tcltk2_1.2-11
## [136] bit_4.0.4               class_7.3-19            stringi_1.7.6
## [139] sass_0.4.0              blob_1.2.2              latticeExtra_0.6-29
## [142] caTools_1.18.2          memoise_2.0.1           e1071_1.7-9
```
