# Supplementary material for: Effects of Lactiplantibacillus plantarum and Lacticaseibacillus paracasei supplementation on the single-cell fecal parasitome in children with celiac disease autoimmunity: a randomized, double-blind placebo-controlled clinical trial
Source: Parasit Vectors. 2023 Nov 9;16:411. doi: 10.1186/s13071-023-06027-1 (PMC10636941; doi:10.1186/s13071-023-06027-1)
Supplement: Supplementary file 2 — Additional file 2: Figure S1. The detection primers and hydrolysis probe annealing to the consensus of several sequences of Entamoeba sp. Figure S2. Positivity of Blastocystis sp., Dieantamoeba fragilis and Entamoeba sp. in individual study samples. Figure S3. Constrained ordination (redundancy analysis) of the bacteriome community composition by Blastocystis sp. positivity, Dieantamoeba fragilis positivity and intervention with lactobacilli. Figure S4. The dispersion of samples negative for Blastocystis sp. or Dieantamoeba fragilis is significantly higher than that of their positive counterparts (P < 0.001), so testing by Permutational Multivariate Analysis of Variance would not be meaningful—its significant result may reflect not only the significant difference in centroid position but also the difference in spread. Table S1. Specific primers and probes for Entamoeba sp. quantitative PCR. [file 13071_2023_6027_MOESM2_ESM.docx]

### Additional files

**Figure S1.** The detection primers and hydrolysis probe annealing to the consensus of several sequences of *Entamoeba* sp.

**
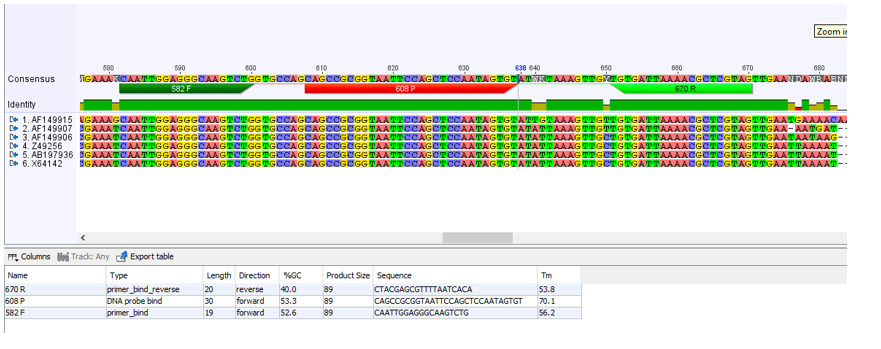
**


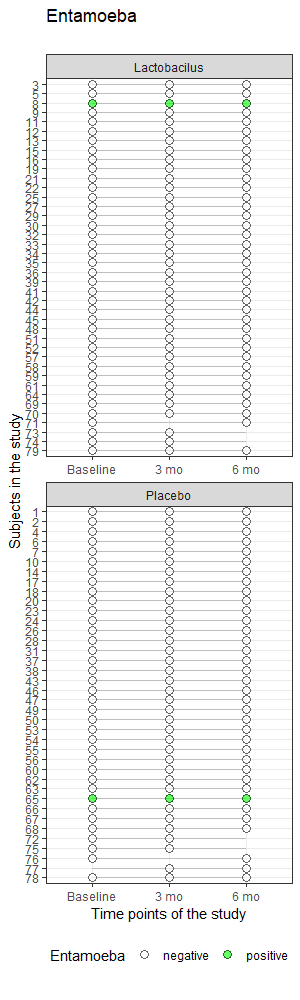

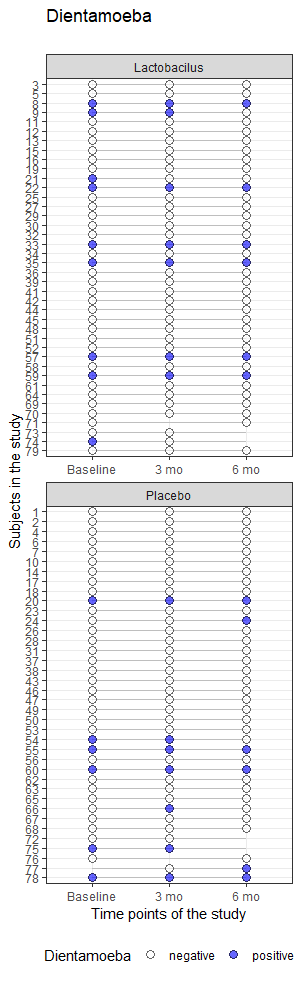

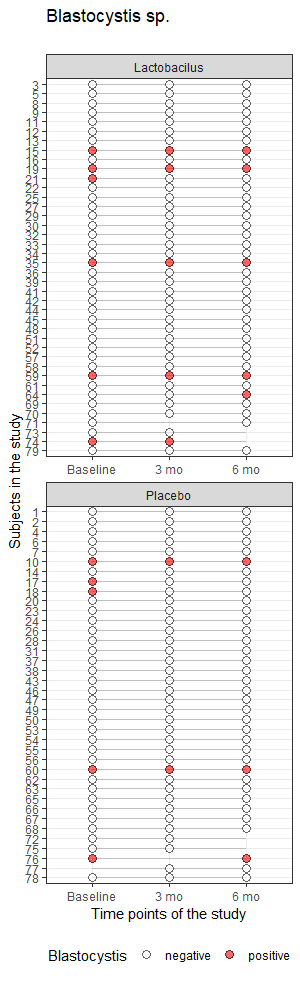
**Figure S2**. Positivity of *Blastocystis* sp., *Dieantamoeba fragilis* and *Entamoeba* sp. in individual study samples.

**Figure S3.** Constrained ordination (redundancy analysis) of the bacteriome community composition by *Blastocystis* sp. positivity, *D.fragilis* positivity and intervention with lactobacilli.


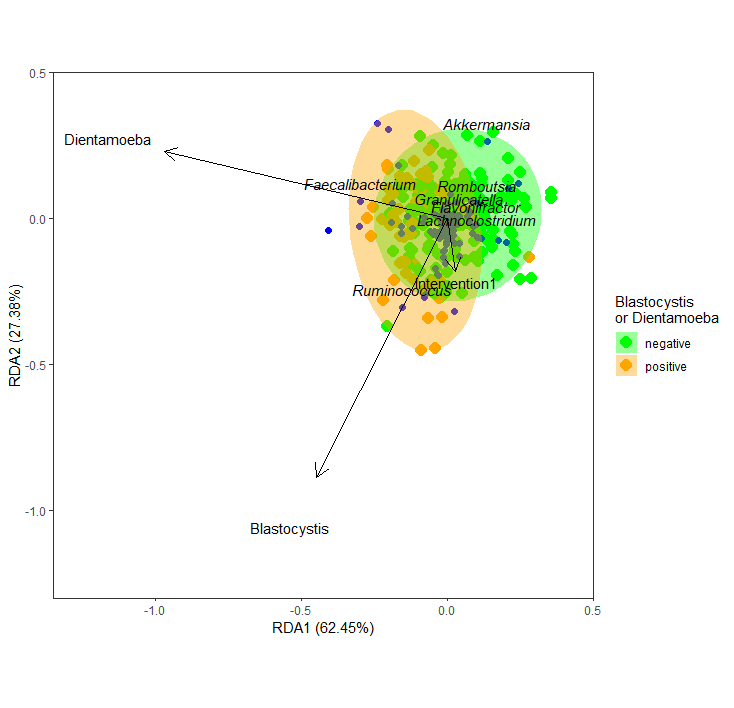


The bacteriome data are aggregated at the taxonomic level of genus. Effect of the study intervention are minor ("Intervention1"), with no significant explanation of the overall community variance at the genus level (P=0.591).

Associations of *Blastocystis* sp. (P=0.003) and *D. fragilis* (P=0.001) with the bacteriome community composition were both highly significant, but the explained proportion of overall community variance was very low (1.0% for *Blastocystis* sp. and 1.95% for *D. fragilis*).

Notably, the effects of the two protozoa were nearly orthogonal, i.e. the associations with individual microbes of the bacteriome differed.

Blue dots are bacterial taxa, orange and green dots are samples. The genera that are associated with positivity for either of the parasites (Table 2) are labelled.

**Figure S4**. The dispersion of samples negative for *Blastocystis* sp. or *D. fragilis* is significantly higher than that of their positive counterparts (P<0.001), so testing by Permutational Multivariate Analysis of Variance would not be meaningful - its significant result may reflect not only the significant difference in centroid position but also the difference in spread.


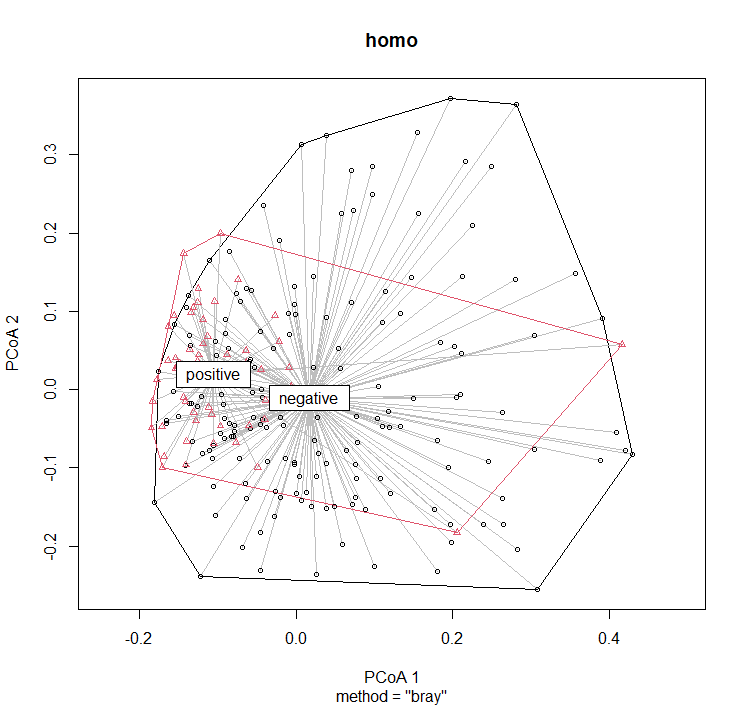


**Table S1**. Specific primers and probes for *Entamoeba* sp. quantitative PCR

| Primers and probes | Sequences |
| --- | --- |
| forward primer (**entamoeba_F**) | 5´-CAATTGGAGGGCAAGTCTG-3´ |
| reverse primer (**entamoeba_R**) | 5´-CTACGAGCGTTTTAATCACA-3´ |
| hydrolysis probe (**entamoeba_P**) | 5´FAM- CAGCCGCGGTAATTCCAGCTCCAATAGTGT -TAMRA 3´ |
